# Mechanochemistry Drives Alkene Difunctionalization via Radical Ligand Transfer and Electron Catalysis

**DOI:** 10.1002/advs.202402970

**Published:** 2024-06-03

**Authors:** Subrata Patra, Bhargav N. Nandasana, Vasiliki Valsamidou, Dmitry Katayev

**Affiliations:** ^1^ Department of Chemistry Biochemistry, and Pharmaceutical Sciences University of Bern Freiestrasse 3 Bern 3012 Switzerland

**Keywords:** electron catalysis, mechanochemistry, nitration, olefin difunctionalization, radical ligand transfer (RLT)

## Abstract

A general and modular protocol is reported for olefin difunctionalization through mechanochemistry, facilitated by cooperative radical ligand transfer (RLT) and electron catalysis. Utilizing mechanochemical force and catalytic amounts of 2,2,6,6‐tetramethylpiperidinyloxyl (TEMPO), ferric nitrate can leverage nitryl radicals, transfer nitrooxy‐functional group via RLT, and mediate an electron catalysis cycle under room temperature. A diverse range of activated and unactivated alkenes exhibited chemo‐ and regioselective 1,2‐nitronitrooxylation under solvent‐free or solvent‐less conditions, showcasing excellent functional group tolerance. Mechanistic studies indicated a significant impact of mechanochemistry and highlighted the radical nature of this nitrative difunctionalization process.

## Graphical abstract

Mechanochemistry: Herein, a modular protocol is reported for olefin difunctionalization through mechanochemistry, facilitated by cooperative radical ligand transfer (RLT) and electron catalysis. A broad spectrum of activated and unactivated alkenes demonstrated chemo‐ and regioselective 1,2‐nitronitrooxylation employing ferric nitrate as a dual‐source of nitro and nitrooxy‐functional groups, conducted under solvent‐free conditions.

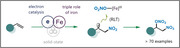

## 1 Introduction

**Figure 1:**
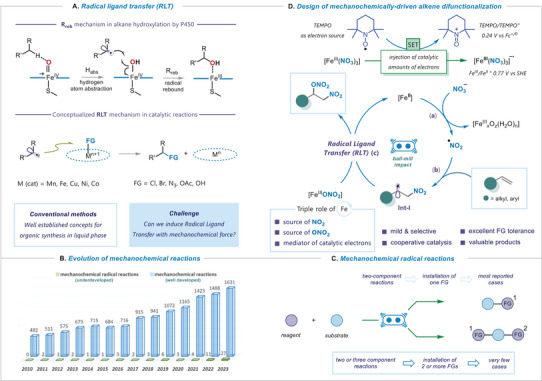
A) Radical ligand transfer concept. B) Evaluation of mechanochemical transformations since 2010 (blue), progress in mechanochemical radical reactions (green). C) Two and three‐component mechanochemical radical reactions. D) This work: design of mechanochemically‐driven alkene difunctionalization.

The increasing interest in the selective difunctionalization of alkenes emphasizes the potential of this strategy to generate molecular complexity from readily available organic feedstock and in a high atom‐ and step‐economy fashion.^[^ ^1^ ^]^ The interplay between intermolecular two‐ or three‐component radical difunctionalization processes offers a fresh avenue for this purpose.^[^ ^2^ ^]^ Single electron transfer (SET), homolytic scission (HS), hydrogen (HAT), and halogen atom transfer (XAT), are few catalytic concepts that facilitate the access to radical intermediates, while atom transfer radical addition (ATRA) and radical polar crossover (RPC) stand as fundamental methods to drive selectivity in radical‐based olefin difunctionalization.^[^ ^2^, ^3^ ^]^ Despite significant advances of these radical toolboxes, the incorporation of two distinct functionalities from orthogonal reagents, especially across unactivated olefin molecules, remains challenging in the field of synthetic radical chemistry. The mechanistic feature of the sequential events of hydrogen atom abstraction (Habs) and radical rebound (Rreb), identified in metalloenzyme catalyzed C(sp3)−H hydroxylation, has been recently conceptualized into a general synthetic paradigm – radical ligand transfer (RLT) (**Figure** **1**A).^[^ ^4^ ^]^ A noteworthy advantage of this innovative manifold is its ability to be integrated with various radical‐based elementary steps, functionalizing alkyl radicals in catalytic and selective manner. For example, when coupled with electrochemical or photochemical approaches, RLT has demonstrated substantial potential in the seamless attachment of two different functional groups across an unsaturated bond of both activated and unactivated alkenes.^[^ ^5^ ^]^ Mechanistically, it entails the outer‐sphere shuttle of a ligand from a redox‐active metal center to a transient alkyl radical intermediate, forming a new carbon–ligand bond, while the metal undergoes reduction through SET. Sequential reoxidation of the metal center occurs in the presence of a stoichiometric anionic ligand, regenerating the active species for the RLT step and enabling catalysis. However, the use of harmful organic solvents, complex reaction conditions, elevated temperatures, and extended reaction times need to be addressed if RLT process is intended for use in sustainable and fine‐chemical synthesis.[Supplementary-material advs8552-supitem-0001]

In this context, solvent‐less or solvent‐free mechanochemical transformations using ball milling have emerged as notable alternatives for researchers across various disciplines (Figure 1B).^[^ ^6^ ^]^ This straightforward and environmentally friendly approach has been widely adopted in material science,^[^ ^7^ ^]^ polymer chemistry,^[^ ^8^ ^]^ and (in)organic synthesis,^[^ ^9^ ^]^ providing cleaner and more sustainable methodologies. In addition to their environmental benefits, mechanochemistry offers the potential to explore new chemical space with unique reactivities and selectivity, contrasting with conventional liquid‐phase reactions.^[^ ^10^ ^]^ Despite rapid progress in mechanochemical two‐electron transformations, catalytic radical reactions that proceed effectively under solid‐state conditions remain relatively scarce in the literature (Figure 1B). Significant advances in this field have been demonstrated with the exploration of mechanochemical redox chemistry using piezoelectric materials such as tetragonal BaTiO3, which effectively mimic the well‐established oxidative quenching cycle of a photoredox catalysis.^[^ ^11^ ^]^ Notably, mechanochemistry has recently been enhanced with other energy sources, offering new opportunities for conducting SET transformations with minimal or even in the absence of solvents.^[^ ^12^ ^]^ In the realm of successfully developed mechanochemical radical transformations, the predominant focus has been on establishing single chemical bonds, whereas the installation of two distinct functionalities across the π‐bond of an olefin molecule via two‐ or three‐component catalytic mechanochemical processes has almost no documentation in the literature (Figure 1C).

## 2 Results and Discussion

### 2.1 Reaction Optimization

**Figure 2:**
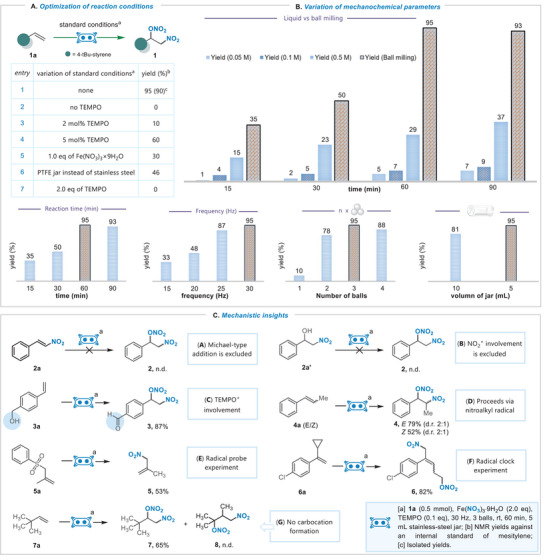
Reaction development. A) Optimization of reaction conditions. B) Variation of mechanochemical parameters. C) Mechanistic insights.

Given our focus on difunctionalization reactions using RPC and RLT liquid‐phase catalysis,^[^ ^13^ ^]^ with an emphasis on nitro compounds synthesis, we set out to develop a modular catalytic platform for the radical difunctionalization of alkenes that operates under mechanochemical conditions. We propose the general design plan for mechanoredox alkene difunctionalization, as detailed in Figure 1D. In light of our recent development in the electron‐driven nitration of unsaturated hydrocarbons using ferric nitrate,^[^ ^14^ ^]^ we hypothesized that injecting catalytic amounts of electrons (from TEMPO) could reduce Fe(NO_3_)3×9H_2_O under mechanochemical force, sustaining the continuous presence of **·**NO_2_ in the reaction medium. The generation of nitryl radicals from ferric nitrate via SET process was previously studied using pulse radiolysis.^[^ ^14^ ^]^ Pulse radiolysis is a robust technique for investigating short‐living and highly reactive intermediates and radicals. The study suggested the possibility of a single‐electron reduction of NO_3_− to NO_3_ **·**2−, which, upon reaction with H^+^, delivers **·**NO_2_. A Giese‐type addition^[^ ^15^ ^]^ of N‐centered radicals to an olefin molecule will occur, leading to the formation of transient nitroalkyl intermediate Int‐I. The oxidation of these species with an external oxidant to perform RPC may pose a challenge to the redox balance of the system. Therefore, we anticipated, that the radical ligand transfer with ferric nitrate, if operational under mechanochemical force, could facilitate the selective transfer of a second functional group (ONO_2_), delivering a difunctionalized product and restoring an electron to complete the catalytic cycle. Herein, we report that the mechanochemical approach using ball milling allows for a highly selective, robust, and modular method for the radical difunctionalization of unsaturated hydrocarbons. Carrying out the reaction in the presence of ferric nitrate and catalytic amounts of TEMPO, rapid nitronitrooxylation^[^ ^16^ ^]^ of alkenes takes place, delivering unique adducts with high levels of chemo‐ and regioselectivity. Importantly, the success of this protocol relies on the cooperative processes of radical ligand transfer (RLT) and electron e) catalysis,^[^ ^17^ ^]^ which can be effectively mediated under mechanochemical force. A significant deviation of the reaction outcome can be observed between liquid and solid‐state phases. The modularity of this mechanochemically driven catalytic platform is further demonstrated by its ability to selectively introduce other functional groups. With our group's keen interest in developing novel tools to access nitro‐derived molecules,^[^ ^18^ ^]^ we initiated an examination of the proposed mechanistic scenario in Figure 1D using 4‐tert‐butylstyrene as the model substrate, ferric nitrate as the source of nitro‐ and nitrooxy‐functional groups and TEMPO as the electron‐donating agent. Mechanochemical optimization was carried out using a Retsch MM400 mixer mill in a 5 mL stainless‐steel milling jar with 5 mm‐diameter stainless‐steel balls. The best outcome was found during the ball milling of 1a in the presence of 2 eq. Fe(NO^3^)3×9H^2^O and 10 mol% of TEMPO at 30 Hz frequency for 60 min, using 3 stainless‐steel balls, leading to a quantitative formation of disubstituted product 1 with an isolated yield of 95% (**Figure** **2**A, entry 1). Reducing the loading of TEMPO or iron nitrate had a notable impact on the product formation, as shown in entries 2–5. Other electron donors including phthalimide‐N‐oxyl (PINO) and 9‐azabicyclo[3.3.1]nonane N‐oxyl (ABNO) can also be used in this transformation, yielding a comparable amount of product 1. Additionally, we compared reactivity using a PTFE jar under the optimized conditions. The results closely aligned with the initial estimates, yielding 46% of the desired product 1. The yield is lower than the yield obtained with stainless steel jar, possibly due to the difference in hardness of the material (Figure 2A. entry 6). It's noteworthy that catalytic TEMPO is essential for this transformation (Figure 2A, entry 2). However, increasing the TEMPO loading to 2 equivalents led to the complete cessation of the reaction, as anticipated for a radical process (Figure 2A, entry 7). The effects of grinding time and ball milling frequency were also tested, and 60 min and 30 Hz were found optimal in terms of 1a conversion and product formation (Figure 2B). To maximize ball milling yield, balls must move freely within the jar during vibration to ensure proper mechanical impact. Our study found that using 3 balls in a 5 mL jar delivers optimal impact/contact on the reaction mixture, yielding the highest results, while more balls or larger jar volumes decreased yield (Figure 2B). Having optimized the reaction under solvent‐free conditions, we were curious about the performance of this transformation in the liquid‐phase. As illustrated in Figure 2B, in a liquid‐phase reaction with a concentration of 0.05 m (dimethyl carbonate), only a small amount of product 1 could be obtained. By elevating the concentration to 0.5 m and extending the reaction time to 90 min, a modest improvement in yield up to 37% was attained. The presented results emphasize the significant influence of mechanochemical force on both the rate and efficiency of this catalytic transformation.^[^ ^19^ ^]^

### 2.2 Investigation of Substrate Scope

**Figure 3:**
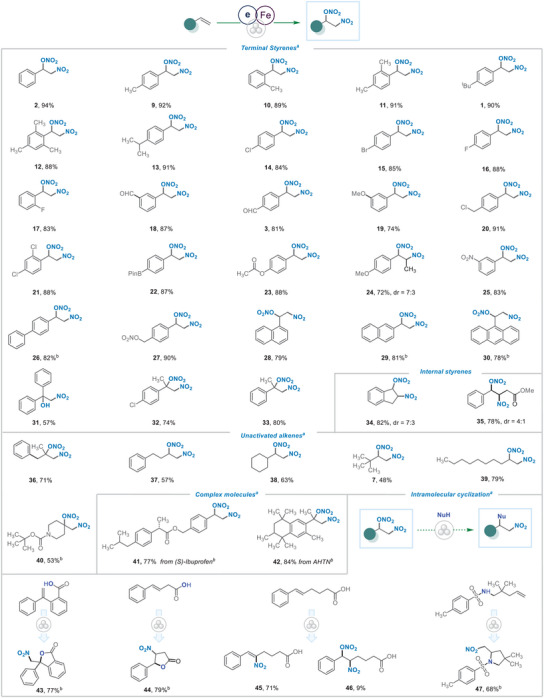
Substrate scope. a) Standard conditions: alkene (0.5 mmol), Fe(NO_3_)3×9H_2_O (2.0 eq), TEMPO (0.1 eq), 30 Hz, rt, 60 min. b) ‘Green’ solvent Me_2_CO_3_ (0.1 mL mg^−1^) was added for solid alkenes.

Next, we directed our efforts toward investigating the mechanistic aspects of the reaction through an experimental approach, specifically focusing on its radical nature and the involvement of a radical ligand transfer paradigm. When ball milling the nitro‐styrene 2a using standard reaction conditions, product 2 was not detected, indicating that the reaction does not proceed through Michael‐type addition (Figure 2A,C). Similarly, the potential involvement of nitronium ions in the reaction mixture was ruled out through the attempted nitration under standard conditions of the alcohol moiety (Figure 2B,C). Remarkably, the evidence for the formation of TEMPO+ was confirmed through iron(III) nitrate/TEMPO‐catalyzed alcohol oxidation under ball milling conditions with substrate 3a (Figure 2C).^[^ ^20^ ^]^ The generation of nitroalkyl radical species was also supported by the difunctionalization reaction of (Z)‐ and (E)‐b‐methylstyrene, yielding the corresponding product 4 in 52% and 79% yield respectively with similar d.r. values. Radical probe experiments with substrate 5a and radical clock reaction with 6a were also successfully conducted under ball milling conditions, thereby reducing the likelihood of the potential oxidation of an in situ generated nitroalkyl radical intermediate. In addition, alkene 7a also underwent nitronitrooxylation to produce 7 in 65%, providing further support for the exclusion of the RPC pathway and preference of RLT. With the optimized conditions in hand, we proceeded to investigate the substrate scope (**Figure** **3**). Initially, aryl‐substituted olefins containing both electron‐donating and withdrawing common functionalities at ortho‐, meta‐ and para‐positions were examined, revealing a high level of chemo‐ and regioselectivity, with product yields ranging from 74% to 95%. It is noteworthy that halogen substituents (14‐17, 20, 21, 32), aldehydes (3, 18), esters, and nitrate esters (22, 23, 27) remained completely untouched under ball milling conditions. Alkene building blocks including di‐ and tri‐substituted aryl substrates (12, 21) and polycyclic aromatics (28‐30) all gave the desired products in excellent yields. Substitution patterns on the alkene fragment (24, 31, 32–35)^[^ ^21^ ^]^ were also well tolerated. Despite the chemical inertness of unactivated alkenes, they demonstrated valuable reactivity under mechanochemically‐ driven catalytic conditions. Terminal alkenes with alkyl linear chains (36, 37, 39) or cyclic systems (38) with amide functional group (40) all yielded the corresponding 1,2‐disubstituted adducts. Encouraged by the excellent reactivity of alkenes and functional group tolerance, we next showcased the successful application of this protocol in the late‐stage functionalization of ibuprofen and AHTN derivatives (41, 42). Encouragingly, our protocol successfully cyclized carboxylic acids and sulfamide functional groups, yielding the cyclized products (43, 44, 47) in good yield. We hypothesize that the presence of free acid and sulfamide facilitated the intramolecular cyclization of the corresponding nitronitrooxylation, leading to the formation of 5‐membered cyclic nitrative derivatives. However, (E)−6‐phenylhex‐5‐enoic acid yielded product 46, which was unstable during the purification and converted to 45. In order to extend the scope of the protocol to alkynes, phenylacetylene, and 1‐octyne were examined under standard reaction conditions. However, after several attempts, only a complex reaction mixture was observed.

### 2.3 Concept Extension

**Figure 4:**
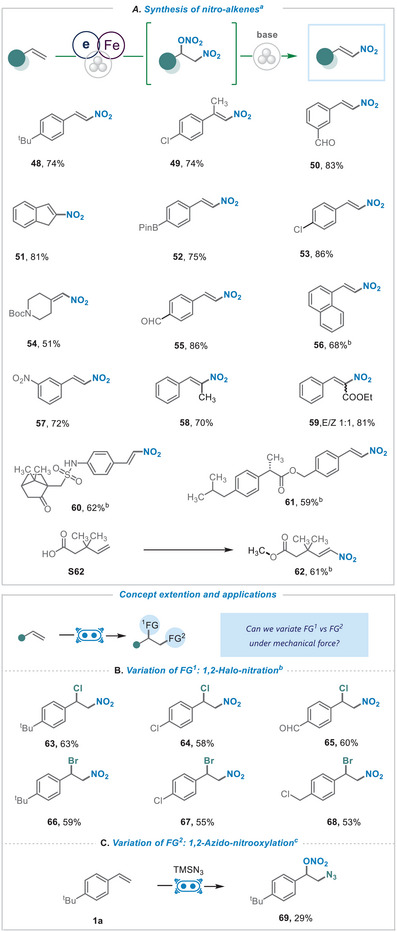
A) Synthesis of nitro‐alkenes. a) Standard conditions and NEt_3_ (1.5 eq) were added in the end of reaction and ball milling for an additional 60 min. Concept extension and applications. (B & C) Variation of functional groups. b) Standard conditions with the addition of Me2CO3 (0.1 µL mg^−1^). c) Standard conditions with the addition of Me2CO3 (0.1 µL mg^−1^) and TMSN3 (1.0 eq).

The ball milling strategy proved highly effective for the in situ conversion of 1,2‐nitronitrooxy adducts to the corresponding nitro alkenes under solvent free reaction conditions, facilitated by the addition of triethylamine. As demonstrated in **Figure** **4**A, activated (48‐53, 55–59), unactivated (54, 62), and complex nitro alkenes (60‐61) can effectively be generated in good to excellent chemical yields. Interestingly, 3,3‐dimethylpent‐4‐enoic acid under standard conditions yielded nitroalkene 62 in good yield. Expanding on our mechanistic insights, we speculated that radical ligand transfer, under mechanochemical force, could shuttle other functional groups FG1, as rapid ligand exchange with nucleophiles may occur at the metal center. Indeed, adding stoichiometric amounts of LiCl or NH_4_Br led to the selective alkene halonitration, delivering the corresponding 1‐chloro‐ (63‐65) and 1‐bromo‐2‐nitroalkanes (66‐68) in up to 63% isolated yield (Figure 4B). These results indicate the potential for a ligand exchange step in a solid‐state or solvent‐less environment under mechanochemical conditions. The FG2 can also be altered in the presence of competitive radical sources. For example, the addition of TMSN3 allowed to generate azidyl radicals (via TEMPO‐mediated azide oxidation)^[^ ^22^ ^]^ and the corresponding 1,2‐azidonitrooxy derivative 69 was isolated in 29% (Figure 4C).

### 2.4 Applications

**Figure 5:**
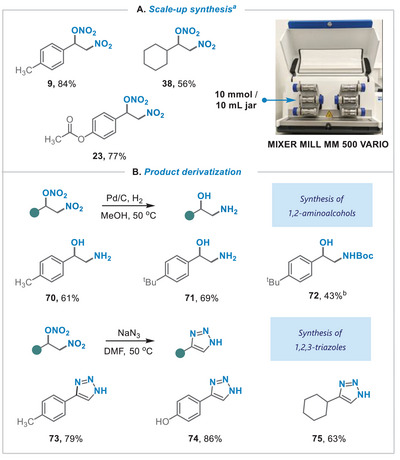
A) Scale‐up synthesis. B) Product derivatization. a) Standard solvent‐free conditions using a 10 mL jar.

To further highlight the operational simplicity and scalability of this protocol, the process was applied to 10 mmol scale in a 10 mL stainless‐steel ball‐milling jar using 10 mm‐diameter balls (**Figure** **5**A). The corresponding 1,2‐nitronitrooxy‐derivatives (9, 23, 38) were obtained with yields almost unaffected by the scale‐up. The 1,2‐nitronitrooxy‐alkanes serve as compelling synthetic building blocks and can undergo various modifications. Conducting hydrogenation in the presence of Pd/C allows for the facile synthesis of an important class of 1,2‐aminoalcohols (70‐72), while performing nucleophilic substitution with NaN_3_ in DMF led to the preparation of diverse triazole derivatives (73‐75) (Figure 5B).

## 3 Conclusion

In conclusion, we developed a mechanochemical process for the difunctionalization of alkenes that proceeds via synergistic radical ligand transfer (RLT) and electron catalysis. In this system, ferric nitrate can be effectively activated in a solid‐state at room temperature in the presence of catalytic amounts of TEMPO, acting as a donor of nitro‐ and nitrooxy‐functional groups as well as an electron mediator to promote the catalytic cycle. A broad array of unique 1,2‐nitronitrooxyalkanes can be synthesized from activated and unactivated olefines using this catalytic platform in a chemo‐ and regioselective fashion, while tolerating a great number of functionalities. The performed mechanistic studies strongly suggested that the process is accelerated by mechanochemistry and proceeds via radical pathway. We anticipate that this robust and straightforward protocol will serve as a driving force for developing solid‐state alkene difunctionalization processes.

## Conflict of Interest

The authors declare no conflict of interest.

## Supporting information

Supporting Information

## Data Availability

The data that support the findings of this study are available from the corresponding author upon reasonable request.
