# Supplementary material for: Mechanochemistry Drives Alkene Difunctionalization via Radical Ligand Transfer and Electron Catalysis
Source: Adv Sci (Weinh). 2024 Jun 3;11(29):2402970. doi: 10.1002/advs.202402970 (PMC11304296; doi:10.1002/advs.202402970)
Supplement: Supplementary file 1 — Supporting Information [file ADVS-11-2402970-s001.pdf]

ADVANCED SCIENCE
Open Access

Supporting Information

for *Adv. Sci.*, DOI 10.1002/advs.202402970

Mechanochemistry Drives Alkene Difunctionalization via Radical Ligand Transfer and Electron Catalysis

*Subrata Patra, Bhargav N. Nandasana, Vasiliki Valsamidou and Dmitry Katayev**

Supporting Information

Mechanochemistry Drives Alkene Difunctionalization via Radical Ligand Transfer and Electron Catalysis

Subrata Patra, Bhargav N. Nandasana, Vasiliki Valsamidou and Dmitry Katayev*

Correspondence to: dmitry.katayev@unibe.ch

Contribution from:

Department of Chemistry, Biochemistry and Pharmaceutical Sciences, University of Bern, Freistrasse 3, 3012 Bern, Switzerland

Table of Contents

1. General Information S3
2. Development of the Reaction Conditions S4
    2.1. Optimisation of reaction time S4
    2.2. Catalyst loading S4
    2.3. Impact of the number of stainless-steel balls S4
    2.4. Effect of ferric nitrate loading S5
    2.5. Effect of frequency S5
3. Availability of Starting Materials S6
    3.1. Commercially available starting materials S6
    3.2. Synthesis of starting materials S7
4. General Procedures S9
    4.1. General procedure for 1,2-nitronitroxylation S9
    4.2. General procedure for nitro-alkene synthesis S10
    4.3. General procedure for 1,2-chloro-nitration S11
    4.4. General procedure for 1,2-bromo-nitration S12
    4.5. General procedure for 1,2-azido-nitroxilation S13
    4.6. General procedure for 1,2-aminoalcohol synthesis S13
    4.7. General procedure for triazole synthesis S13
    4.7. Scale-Up Synthesis S13
5. Mechanistic Investigations S15
6. NMR Data S18
7. NMR Spectra of Isolated Compounds S36
8. References S169

1. General Information

1.1. Material and methods

• Starting materials were commercially available from Thermoscientific – Acros, Sigma Aldrich, Apollo Scientific, Fluorochem, and TCI, unless otherwise specified.

• All ball milling reactions were performed using a Mixer Mill (MM 400 Retsch Gmbh, Hann, Germany) equipped with 5 mL stainless steel grinding vessels and stainless steel balls, unless otherwise specified. Scale up synthesis was performed using Mixer Mill MM 500 Vario equipped with 10 mL stainless steel grinding vessels.

• Reaction progress was monitored by analytical Thin Layer Chromatography (TLC) on Merck silica gel 60 F254 TLC glass plates and visualized with 254 nm light or potassium permanganate staining solutions followed by heating for detection.

• Reaction product purification was performed by flash chromatography using Brunschwig silica 32-63, 60Å under 0.3-0.5 bar overpressure. Medium pressure liquid chromatography (MPLC) was carried out on a CombiFlash Rf 200 System from Teledyne ISCO with a built-in UV-detector and fraction collector, or manually using silica gel SilicaFlash P60, 40-63 µm. Teledyne ISCO RediSep Rf flash columns had particle sizes of 0.035–0.070 mm and 230–400 mesh. Normal phase preparatory HPLC purification was conducted on a Teledyne Isco CombiFlash EZ Prep system using a Macherey-Nagel VP 250/21 Nucleosil 50-5 column.

• ^{1}H- and ^{13}C-NMR spectra were recorded on a Bruker Ultrashield 300 (operating at 300.0 MHz and 75.0 MHz, respectively). Chemical shifts are reported in parts per million (ppm) and coupling constants (J) in Hertz (Hz). ^{1}H-NMR spectra are referenced to the solvent resonance unless noted otherwise ($CDCl_3$ at 7.26 ppm). Peaks are designated as (s = singlet, d = doublet, t = triplet, q = quartet, m = multiplet or unresolved) with coupling constant(s) in Hz and integration. ^{13}C-NMR spectra were recorded with ^{1}H-decoupling and referenced to the solvent resonance unless noted otherwise ($CDCl_3$ at 77.16 ppm). ^{19}F-NMR spectra were recorded with ^{1}H-decoupling unless noted otherwise. Infrared spectra were recorded on a Bruker Tensor III spectrometer equipped with a golden gate.

• Melting points were measured using the OptiMelt Automated Melting Point System Type K/°C. HR-MS (ESI+) mass spectra were measured on a Bruker FTMS 4.7T BioAPEX II and Thermo Scientific LTQ Orbitrap XL equipped with a static nanospray ion source. Mass spectrometry service was operated on VG-TRIBRID for electron impact ionization (EI) or Varian IonSpec Spectrometer for electrospray ionization (ESI), and mass spectra are reported as (m/z). Electron impact ionization mass spectra (EI-MS) were obtained using an Agilent 8890 series GC system and Aglient 5977B GC/MSD.

2. Development of the Reaction Conditions

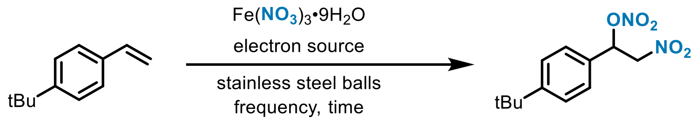

1a **1**

Without any precautions to exclude air or moisture, a Retsch stainless steel vessel (5 mL) equipped with steel balls was charged with a nitrating reagent, reductant, followed by alkene substrate. The reaction vessel cap was locked and placed in the mixer mill. After completion of the reaction, the crude product was dissolved in a solvent, a reference (mesitylene as a standard) was added, and the sample was analyzed by ^{1}H-NMR to determine the corresponding yield.

2.1. Optimisation of reaction time

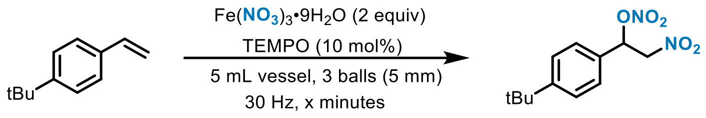

1a **1**

Entry[a]	*Time (mins)*	*Yield (%)*[b]
1	30	50
2	60	95
3	90	93

Table S1. **a.** Reaction conditions: 4-*tert*-butylstyrene **1a** (0.5 mmol, 1.0 equiv), $Fe(NO_3)_3{\times}9H_2O$ (2.0 equiv), TEMPO (0.1 equiv), 30 Hz, 3 stainless steel balls (5 mm), 30-90 minutes. **b.** Yield of **1** determined by ^{1}H-NMR against mesitylene.

2.2. Catalyst loading

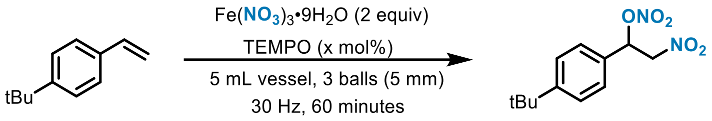

1a **1**

Entry[a]	*TEMPO (equiv)*	*Yield (%)*[b]
1	0.02	10
2	0.05	60
3	0.10	95
4	0.20	88

Table S2. **a.** Reaction conditions: 4-*tert*-butylstyrene **1a** (0.5 mmol, 1.0 equiv), $Fe(NO_3)_3{\times}9H_2O$ (2.0 equiv), TEMPO (0.02-0.20 equiv), 30 Hz, 3 stainless steel balls (5 mm), 60 mins. **b.** Yield of **1** determined by ^{1}H-NMR against mesitylene.

2.3. Impact of the number of stainless-steel balls

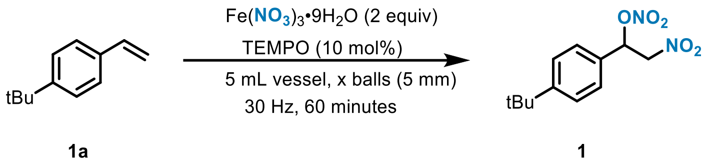

Entry[a]	*Number of balls*	*Yield (%)*[b]
1	1	10
2	2	78
3	3	95
4	4	88

Table S3. a. Reaction conditions: 4-*tert*-butylstyrene **1a** (0.5 mmol, 1.0 equiv), $Fe(NO_3)_3 \times 9H_2O$ (2.0 equiv), TEMPO (0.1 equiv), 30 Hz, (1-4) stainless steel balls (5 mm), 60 mins. **b.** Yield of **1** determined by ^{1}H-NMR against mesitylene.

2.4. Effect of ferric nitrate loading

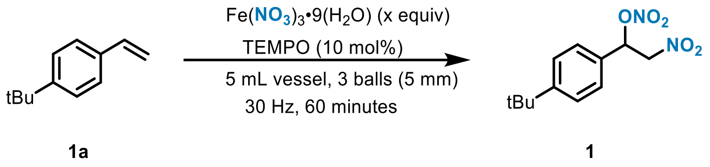

Entry[a]	*Fe(NO3)3•9H2O (equiv)*	*Yield (%)*[b]
1	1	30
2	1.5	67
3	2	95

Table S4. a. Reaction conditions: 4-*tert*-butylstyrene **1a** (0.5 mmol, 1.0 equiv), $Fe(NO_3)_3 \times 9H_2O$ (1-2 equiv), TEMPO (0.1 equiv), 30 Hz, 3 stainless steel balls (5 mm), 60 mins. **b.** Yield of **1** determined by ^{1}H-NMR against mesitylene.

2.5. Effect of frequency

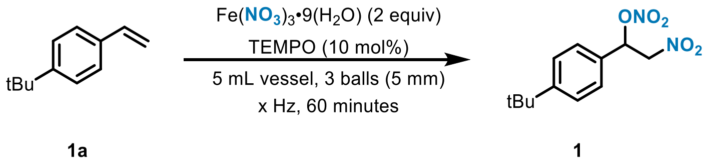

Entry[a]	*Frequency [Hz]*	*Yield (%)*[b]
1	15	33
2	20	48
3	25	87
4	30	95

Table S5. a. Reaction conditions: 4-*tert*-butylstyrene **1a** (0.5 mmol, 1.0 equiv), $Fe(NO_3)_3 \times 9H_2O$ (2.0 equiv), TEMPO (0.1 equiv), (15-30) Hz, 3 stainless steel balls (5 mm), 60 mins. **b.** Yield of **1** determined by ^{1}H-NMR against mesitylene.

3. Availability of Starting Materials

3.1. Commercially available starting materials

Commercially available starting materials were purchased from Thermoscientific – Acros, Sigma Aldrich, Apollo Scientific, Fluorochem and TCI companies.

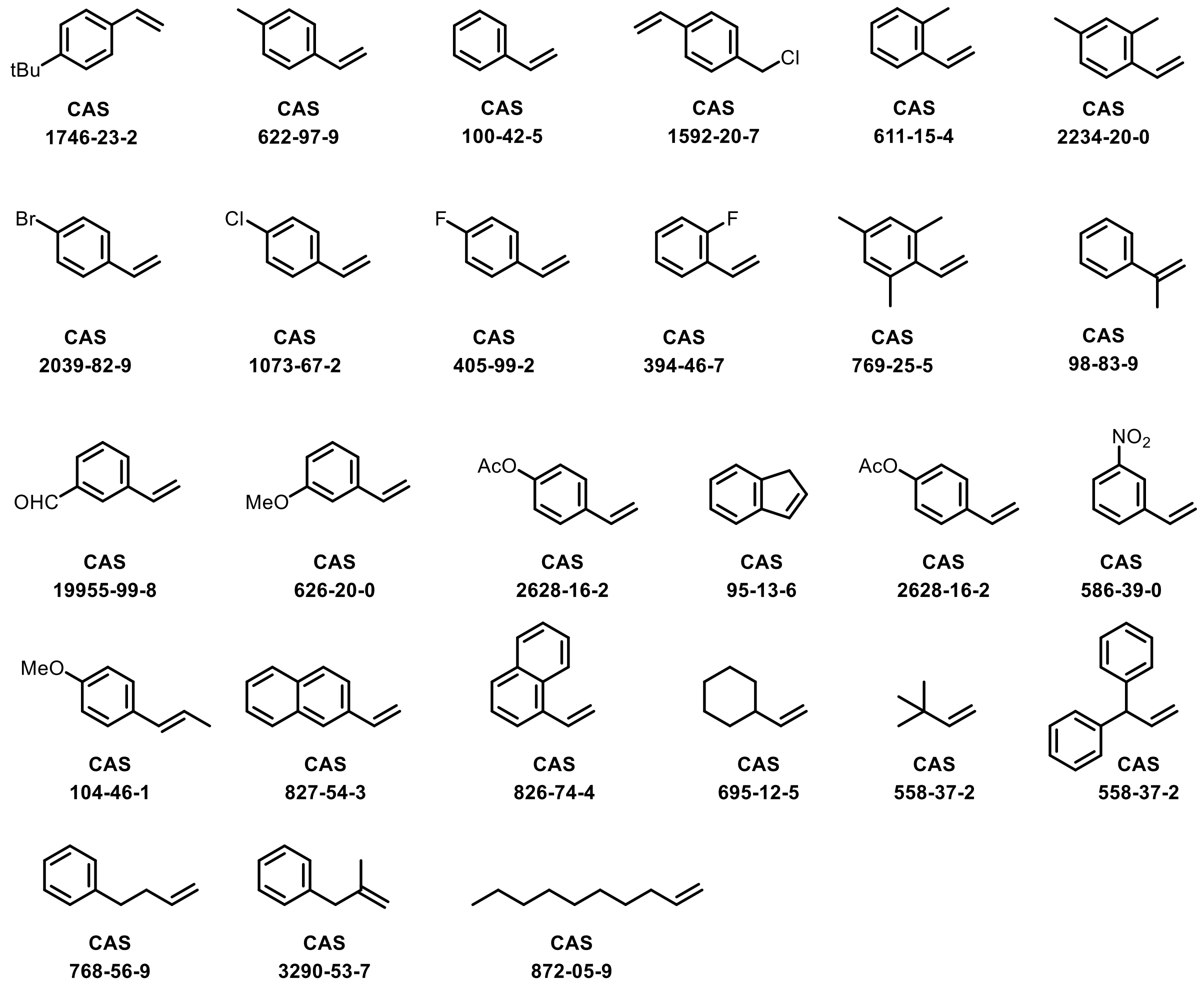

Figure S1. Commercially available starting materials

3.2. Synthesis of starting materials

(E)-(2-nitrovinyl)benzene (S-1)

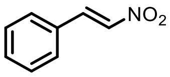

The titled compound was prepared according to our previous reported procedure.[1]

^{1}H-NMR (400 MHz, $CDCl_3$): δ 8.01 (d, *J* = 13.7 Hz, 1H), 7.62 – 7.54 (m, 3H), 7.53 – 7.43 (m, 3H).

2-nitro-1-phenylethan-1-ol (S-2)

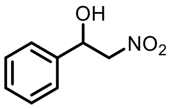

The titled compound was prepared according to our previous reported procedure.[2]

^{1}H-NMR (300 MHz, $CDCl_3$): δ 7.44 – 7.33 (m, 5H), 5.46 (dd, *J* = 9.4, 3.2 Hz, 1H), 4.67 – 4.46 (m, 2H), 2.84 (s, 1H).

(2-methylallyl)sulfonyl)benzene (S-3)

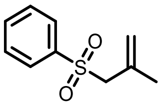

The titled compound was prepared according to reported procedure.[3]

^{1}H-NMR (400 MHz, $CDCl_3$): δ 7.91 – 7.84 (m, 2H), 7.68 – 7.60 (m, 1H), 7.58 – 7.50 (m, 2H), 5.05 – 4.99 (m, 1H), 4.71 – 4.66 (m, 1H), 3.76 (d, *J* = 0.9 Hz, 2H), 1.86 (dd, *J* = 1.6, 1.0 Hz, 3H).

1-chloro-4-(1-cyclopropylvinyl)benzene (S-4)

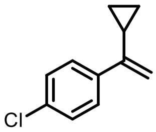

The titled compound was prepared according to reported procedure.[1] A 250 mL three-necked round-bottom flask was flame-dried and then cooled to room temperature under a nitrogen atmosphere. Methyltriphenylphosphonium bromide (1.2 equiv.) and NaH (1.3 equiv., 60% dispersion in mineral oil) were added to the flask along with THF (0.5 M). The mixture was refluxed for 1 hour and then cooled to 0°C. A solution of the corresponding ketone in THF (20 mL) was added dropwise, and the reflux was continued for 12 hours. The reaction was quenched with saturated NH_4Cl (10 mL) until the starting material disappeared, monitored by TLC. The reaction mixture was concentrated under vacuum and filtered on silica gel. The combined filtrates were concentrated under vacuum and further purified by flash chromatography.

^{1}H-NMR (300 MHz, $CDCl_3$): δ 7.53 (d, *J* = 8.6 Hz, 2H), 7.30 (d, *J* = 8.6 Hz, 2H), 5.30 – 5.23 (m, 1H), 4.96 (t, *J* = 1.1 Hz, 1H), 1.61 (ttd, *J* = 8.3, 5.2, 1.2 Hz, 1H), 0.89 – 0.81 (m, 2H), 0.63 – 0.53 (m, 2H).

^{13}C-NMR (75 MHz, $CDCl_3$): δ 148.4, 140.2, 133.4, 128.4, 128.4, 127.5, 109.7, 15.7, 6.8.

2,4-dichloro-1-vinylbenzene (S-5)[4]

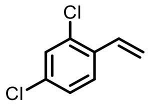

A mixture of methyltriphenylphosphonium bromide (11.0 mmol, 1.1 equiv) and K_2CO_3 (16.0 mmol, 1.6 equiv) was stirred in 150 mL of 1,4-dioxane under a nitrogen atmosphere at room temperature for 4 h in a 250 mL 2-necked round bottom flask. Corresponding benzaldehyde (10 mmol, 1.0 equiv) was added dropwise to the reaction mixture and refluxed for 12 h. Then flask was cooled to room temperature, reaction mixture was filtered, and solvents were removed under vacuum. The products were purified by silica gel chromatography using n-hexane : EtOAc (10:1) as the eluent. The title compound was obtained as a colorless oil in 83% isolated yield.

^{1}H-NMR (300 MHz, $CDCl_3$): δ 7.49 (d, *J* = 8.4 Hz, 1H), 7.37 (d, *J* = 2.1 Hz, 1H), 7.21 (ddd, *J* = 8.4, 2.1, 0.6 Hz, 1H), 7.04 (dd, *J* = 17.5, 11.0 Hz, 1H), 5.73 (dd, *J* = 17.5, 1.0 Hz, 1H), 5.41 (dd, *J* = 11.0, 0.9 Hz, 1H).

^{13}C-NMR (75 MHz, $CDCl_3$): δ 134.5, 134.0, 133.8, 132.3, 129.5, 127.5, 127.4, 117.2.

4,4,5,5-tetramethyl-2-(4-vinylphenyl)-1,3,2-dioxaborolane (S-6)[5]

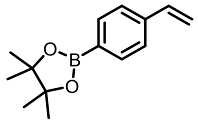

Pinacol (5.5 mmol, 1.1 equiv) was added in one portion to a solution of 4-vinylphenylboronic acid (5.0 mmol, 1.0 equiv) and $MgSO_4$ (10 mol%) in THF (15.0 mL). After stirring the resulting mixture for 2 hours at room temperature, it was filtered and concentrated under vacuum. The crude product was then purified by column chromatography on silica gel using Hexanes: EtOAc 95 : 5 (v/v). The title compound **S-6** was obtained as a colorless oil in 95%.

^{1}H-NMR (300 MHz, $CDCl_3$): δ 7.79 (d, *J* = 8.1 Hz, 2H), 7.42 (d, *J* = 8.0 Hz, 2H), 6.74 (dd, *J* = 17.6, 10.9 Hz, 1H), 5.82 (dd, *J* = 17.6, 1.0 Hz, 1H), 5.30 (dd, *J* = 10.8, 0.9 Hz, 1H), 1.36 (s, 12H).

^{13}C-NMR (75 MHz, $CDCl_3$): δ 140.3, 137.0, 135.2, 125.6, 115.0, 83.9, 25.0.

4-vinylbenzyl nitrate (S-7)

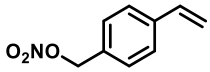

A suspension of $AgNO_3$ (10.0 mmol, 5.0 equiv) in CH_3CN (2 mL) was combined with a solution of 1a (2.0 mmol, 1.0 equiv) in CH_3CN (15 mL), and the resulting mixture was stirred at 60°C for 4 hours under light-shielding. Afterward, the mixture was filtered through Celite to remove insoluble materials, and the filtrate was concentrated. The titled compound was purified on a silica gel column using EtOAc: n-hexane (9:1) as the eluent, yielding **S-7** in 90% as colorless oil.

^{1}H-NMR (300 MHz, $CDCl_3$): δ 77.44 (d, *J* = 8.3 Hz, 2H), 7.36 (d, *J* = 8.3 Hz, 2H), 6.72 (dd, *J* = 17.6, 10.9 Hz, 1H), 5.79 (dd, *J* = 17.6, 0.8 Hz, 1H), 5.41 (s, 2H), 5.32 (dd, *J* = 10.9, 0.8 Hz, 1H).

^{13}C-NMR (101 MHz, $CDCl_3$): δ 138.9, 136.2, 129.5, 129.0, 126.8, 115.3, 74.7.

4. General procedures

4.1. General procedures for 1,2-nitronitroxylation

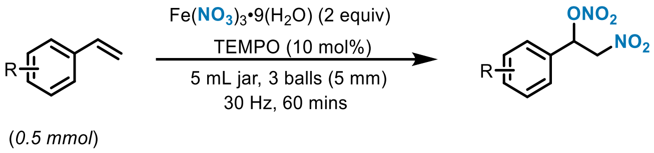

GP1: An alkene (0.50 mmol, 1.0 equiv), $Fe(NO_3)_3 \times 9H_2O$ (1.0 mmol, 2.0 equiv) and TEMPO (10 mol%) were placed in a stainless-steel vessel (5 mL) equipped with 3 stainless-steel balls (5 mm) under air. Next, the ball milling vessel was closed and placed in the mixer mill (Retch MM400) for 60 mins at a frequency of 30 Hz. After the reaction was completed, the contents were removed from the vessel and either purified directly by column chromatography on silica gel using EtOAc/n-hexane (1/10) as the eluent or collected without further purification to obtain the desired product.

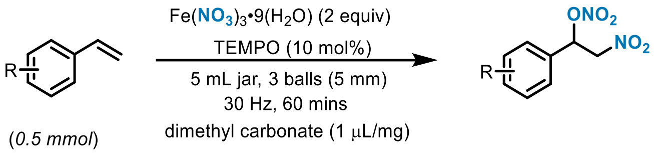

GP2: An alkene (0.50 mmol, 1.0 equiv), $Fe(NO_3)_3 \times 9H_2O$ (1.0 mmol, 2.0 equiv), dimethyl carbonate (1 µL/mg of alkene) and TEMPO (10 mol%) were placed in a stainless-steel vessel (5 mL) with 3 stainless-steel balls (5 mm). Next, the ball milling vessel was sealed and placed in the mixer mill (Retch MM400) for 60 minutes at a frequency of 30 Hz. After the reaction was finished, the contents were scraped off the vessel and purified directly by column chromatography on silica gel using EtOAc/n-hexane(1/10) as the eluent or collected without further purification to obtain the desired product.

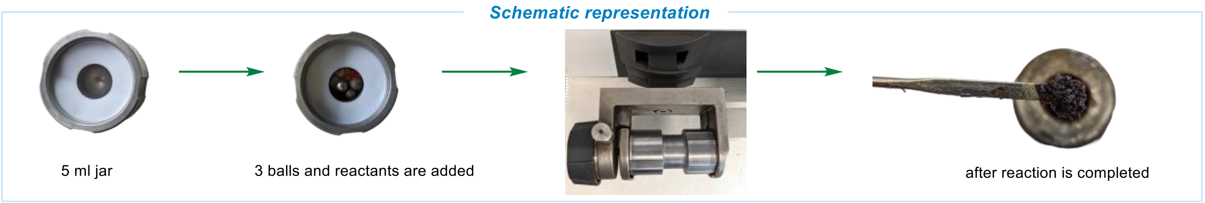

Figure S2. Graphical presentation of the reaction set-up

4.2. General procedure for nitro-alkene synthesis

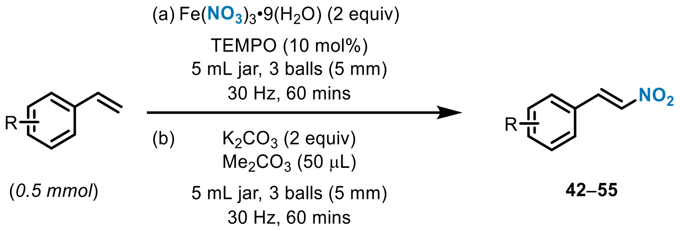

GP3: An alkene (0.50 mmol, 1.0 equiv), $Fe(NO_3)_3 \times 9H_2O$ (1.0 mmol, 2.0 equiv) and TEMPO (10 mol%) were placed in a stainless-steel vessel (5 mL) equipped with 3 stainless-steel balls (5 mm). Next, the ball milling vessel was closed and placed in the mixer mill (Retch MM400) for 60 mins at a frequency of 30 Hz. After the reaction was completed, K_2CO_3 (2.0 equiv), and dimethyl carbonate (50 µL) were added to the reaction vessel. After that the reaction mixture was placed again in the mixer mill (Retch MM400) for additional 60 mins at a frequency of 30 Hz. The contents were scraped off the vessel and subsequently purified by column chromatography on silica gel using a mixture of EtOAc/n-hexane (1/10) as the eluent.

4.2. Screening of bases

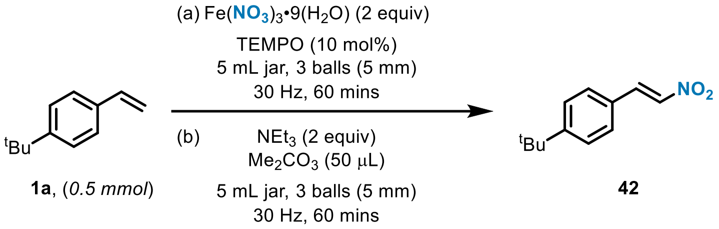

Entry	*Base (2 equiv)*	*Yield (%)*[a]
1	$NaHCO_3$	16
2	$LiOH{\cdot}H_2O$	59
3	Na_2CO_3	24
4	K_2CO_3	68
5	NEt_3	88

Table S6. a. Yield of **42** determined by ^{1}H-NMR against mesitylene.

4.3. General procedure for 1,2-chloro-nitration

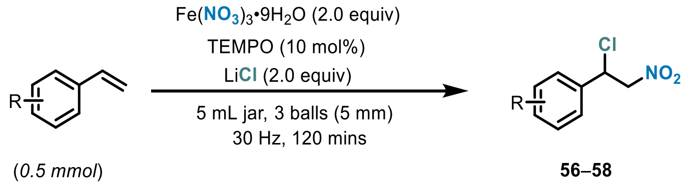

GP4: An alkene (0.50 mmol, 1.0 equiv), $Fe(NO_3)_3{\times}9H_2O$ (1.0 mmol, 2.0 equiv), TEMPO (10 mol%), LiCl (1.0 mmol, 2.0 equiv) and dimethyl carbonate (1 μL/mg) were placed in a stainless-steel vessel (5 mL) equipped with 3 stainless-steel balls (5 mm). Next, the ball milling vessel was closed and placed in the mixer mill (Retch MM400) for 120 mins at a frequency of 30 Hz. After the reaction was finished, the contents were scraped off the vessel and purified directly on silica gel using EtOAc/n-hexane (1/10) as the eluent.

4.3. Screening of chloride salts

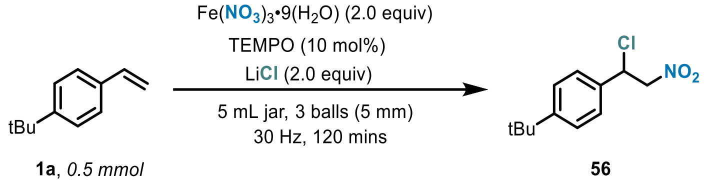

Entry	*Additive (2 equiv)*	*Yield (%)[a]*
1	KCl	12
2	NH_4Cl	46
3	NaCl	31
4	LiCl	65

Table S7. a. Yield of **56** determined by ^{1}H-NMR against mesitylene.

4.4. General procedure for 1,2-bromo-nitration

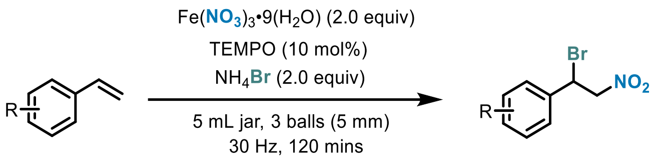

GP5: An alkene (0.50 mmol, 1.0 equiv), $Fe(NO_3)_3{\times}9H_2O$ (1.0 mmol, 2.0 equiv), TEMPO (10 mol%), NH_4Br (1.0 mmol, 2.0 equiv) and dimethyl carbonate (1 µL/mg) were placed in a stainless-steel vessel (5 mL) with 3 stainless-steel balls (5 mm). Next, the ball milling vessel was closed and placed in the mixer mill (Retch MM400) for 120 mins at a frequency of 30 Hz. After the reaction was finished, the content was scraped off the vessel and purified directly on silica gel using EtOAc/n-hexane (1/10) as the eluent.

4.4. Screening of bromide salts

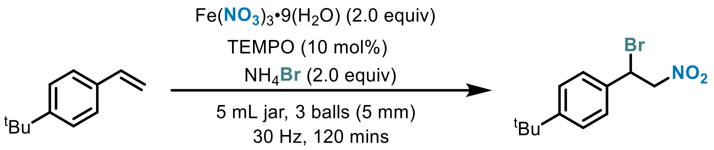

Entry	*Additive (X eq)*	*Yield (%)[a]*
1	KBr	5
2	NH_4Br	64
3	NaBr	3
4	LiBr	29

Table S8. a. Yield of **59** determined by ^{1}H-NMR against mesitylene.

4.5. General procedure for 1,2-azido-nitroxilation

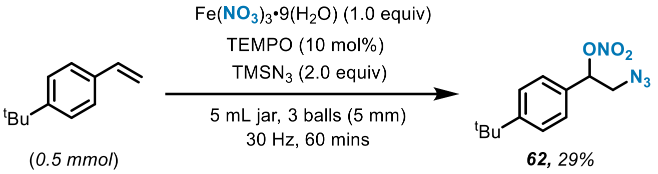

GP6: 4-Tertbutyl styrene (0.50 mmol, 1.0 equiv), $Fe(NO_3)_3 \times 9H_2O$ (0.5 mmol, 1.0 equiv), TEMPO (10 mol%), and $TMSN_3$ (1.0 mmol, 2.0 equiv) were placed in a stainless-steel vessel (5 mL) additionally equipped with 3 stainless-steel balls (5 mm). Next, the ball milling vessel was closed and placed in the mixer mill (Retch MM400) for 60 mins at frequency of 30 Hz. After the reaction was finished, the contents were scratched off the vessel and purified directly by column chromatography on silica gel using EtOAc/n-hexane (1/10) as the eluent.

4.6. General procedure for 1,2-aminoalcohol synthesis

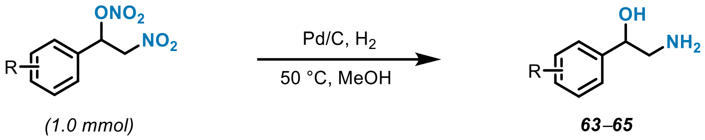

GP7: Nitronitrooxylated compound (1.0 mmol) was placed in a crimp vial and dissolved in MeOH (2 mL). Pd/C (5 wt%, 0.40 equiv) was added and the vial was closed with a crimp cap. The ballon of H_2 was placed over the vial. The reaction mixture was stirred for 12 h at 50°C. After completion, the crude mixture was purified by column chromatography on silica gel (DCM/MeOH/TEA). Note: The amino alcohol (e.g., **64**) can also be protected prior to purification by adding anhydrous $NaHCO_3$ (2.0 equiv) and Boc_2O (1.2 equiv) to the crude mixture and stirring for 12 h at rt. The protected compound was then purified by column chromatography on silica gel using EtOAc/n-hexane (4/6) as the eluent.

4.7. General procedure for triazole synthesis

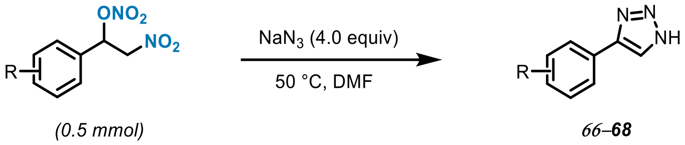

GP8: Nitronitrooxylated compound (0.5 mmol) was dissolved in DMF (2 mL) and sodium azide (2.0 mmol, 4 equiv) was added. The mixture was stirred for 12 h at 50°C and was then washed with a saturated Na_2CO_3 solution (2 mL) and extracted with diethyl ether (3 x 5 mL). The organic layer was washed with water (5 x 10 mL) and brine and then dried over magnesium sulfate, filtered, and concentrated under vacuum. The crude mixture was purified with column chromatography on silica gel using EtOAc/n-hexane (3/7) as the eluent.

4.8. Scale-Up Synthesis

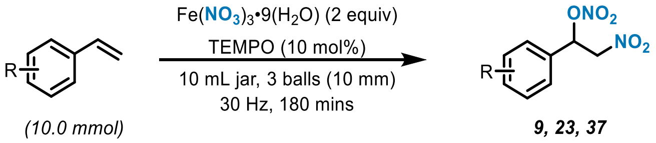

A 10 mL ball milling vessel equipped with 3 balls (10 mm) was charged with alkene (10.0 mmol, 1.0 equiv), $Fe(NO_3)_3 \times 9H_2O$ (2.0 equiv), and TEMPO (10 mol%). The jar was then closed and placed into the ball milling machine and reaction was carried out at 30 Hz frequency for 180 minutes. The crude

product was purified by flash column chromatography on silica gel using EtOAc/n-hexane (1/10) as the eluent to get the desired product.

5. Mechanistic insights

5.1. Experiment 1:

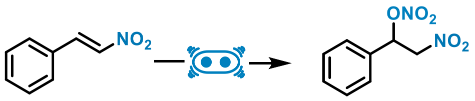

2a **2**, n.d.

A 5 mL ball milling vessel equipped with 3 balls (5 mm) was charged under air with (E)-(2-nitrovinyl)benzene (0.5 mmol, 1.0 equiv), $Fe(NO_3)_3 \times 9H_2O$ (2.0 eq), and TEMPO (10 mol%). The jar was then closed and placed into the ball milling machine and the reaction was carried out at 30 Hz frequency for 60 minutes. The crude product was shortly purified via silica gel and analyzed by ^{1}H-NMR.

5.2. Experiment 2:

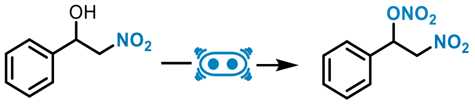

2a' **2**, n.d.

2-Nitro-1-phenylethane-1-ol (0.5 mmol, 1.0 equiv) was added to a 5 mL ball milling vessel equipped with 3 balls (5 mm). After that $Fe(NO_3)_3 \times 9H_2O$ (2.0 eq), and TEMPO (10 mol%) were introduced sequentially under air. The jar was then closed and placed into the ball milling machine, and the reaction was carried out at 30 Hz frequency for 60 minutes. The crude product was shortly purified via silica gel and analyzed by ^{1}H-NMR.

5.3. Experiment 3:

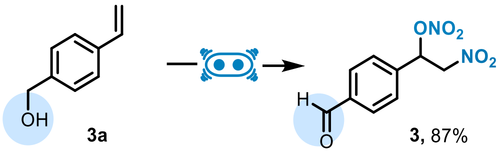

3a **3**, 87%

A 5 mL ball milling vessel equipped with 3 balls (5 mm) was charged with (4-vinylphenyl)methanol (0.5 mmol, 1.0 equiv) was added into the jar. After that $Fe(NO_3)_3 \times 9H_2O$ (2.0 eq), and TEMPO (10 mol%) were introduced sequentially. The jar was then closed and placed into the ball milling machine and the reaction was carried out at 30 Hz frequency for 60 minutes. The crude product was shortly purified via silica gel and analyzed by ^{1}H-NMR.

5.4. Experiment 4:

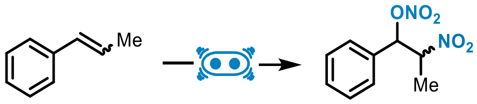

4a (E/Z) **4**, *from E* 79% (d.r. 2:1)
from Z 52% (d.r. 2:1)

A 10 mL ball milling jar equipped with 3 balls (10 mm) was taken and then either (*E*)-prop-1-en-1-ylbenxene or (*Z*)-prop-1-en-1-ylbenxene (0.5 mmol, 1.0 eq) was added into the jar. After that $Fe(NO_3)_3 \times 9H_2O$ (2.0 eq), and TEMPO (10 mol%) were added. The jar was then placed into the ball

milling machine and reaction was carried out at 30 Hz frequency for 60 minutes. The crude product was shortly purified via silica gel and analyzed by ^{1}H-NMR.

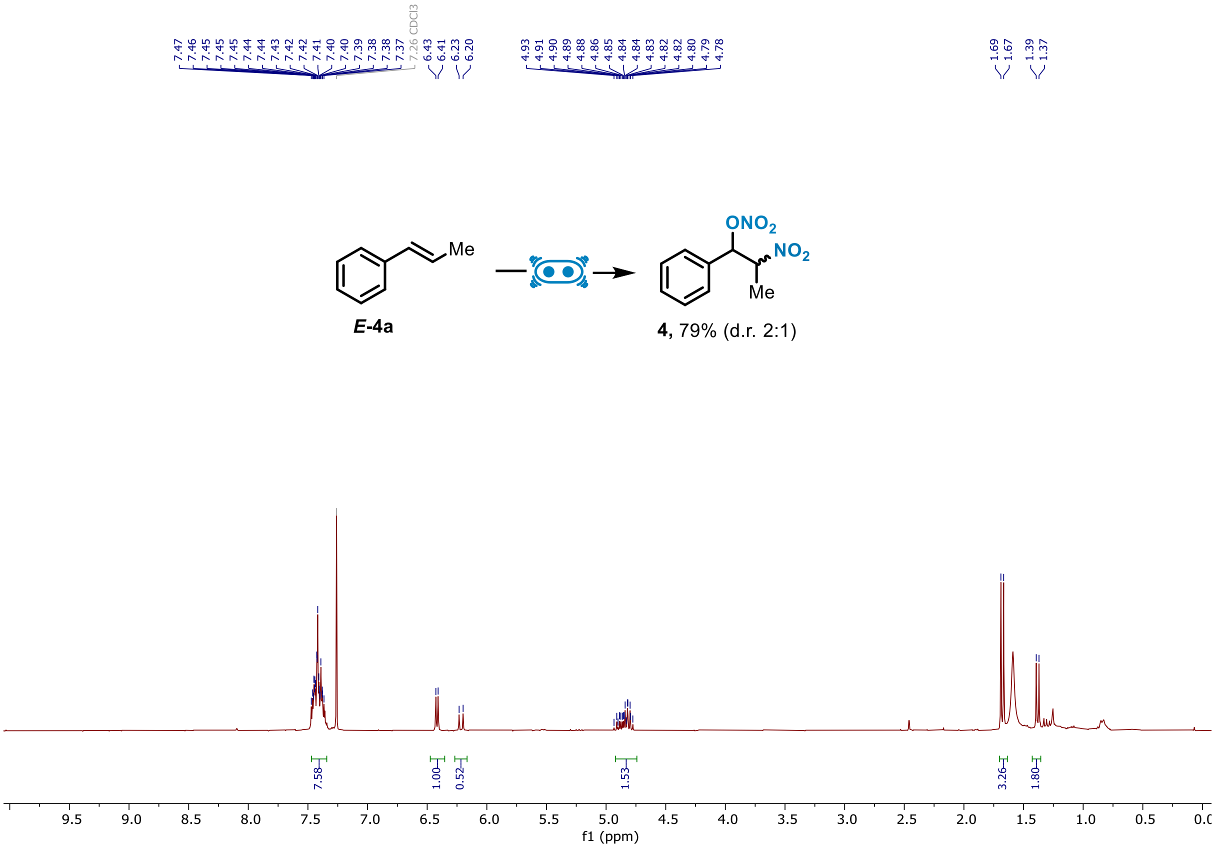

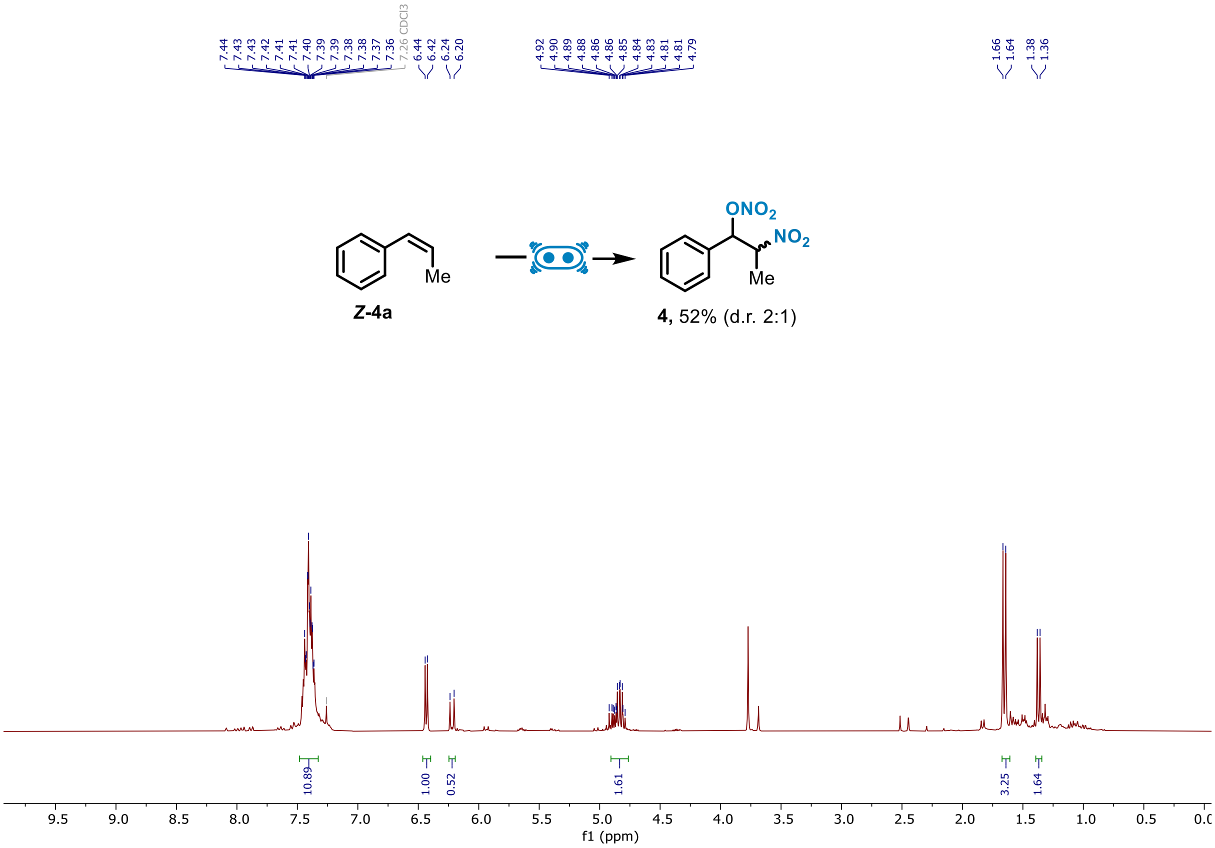

5.5. Experiment 5:

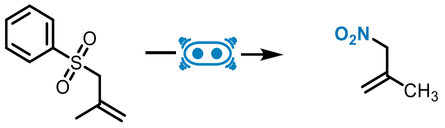

5a **5**, 53%

A 5 mL ball milling vessel equipped with 3 balls (5 mm) was charged with (2-methylallyl)sulfonyl)benzene (0.5 mmol, 1.0 equiv). After that $Fe(NO_3)_3 \times 9H_2O$ (2.0 eq), and TEMPO (10 mol%) were introduced sequentially. The jar was then closed and placed into the ball milling machine and reaction was carried out at 30 Hz frequency for 60 minutes. The crude product was shortly purified via silica gel and analyzed by ^{1}H-NMR. For analytical data see page S18.

5.6. Experiment 6:

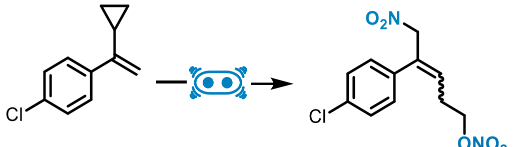

6a **6**, 82%

A 5 mL ball milling vessel equipped with 3 balls (5 mm) was charged with 1-chloro-4-(1-cyclopropylvinyl)benzene (0.5 mmol, 1.0 equiv). After that $Fe(NO_3)_3 \times 9H_2O$ (2.0 eq), and TEMPO (10 mol%) were introduced sequentially. The jar was then closed and placed into the ball milling machine and reaction was carried out at 30 Hz frequency for 60 minutes. The crude product was shortly purified via silica gel and analyzed by ^{1}H-NMR.

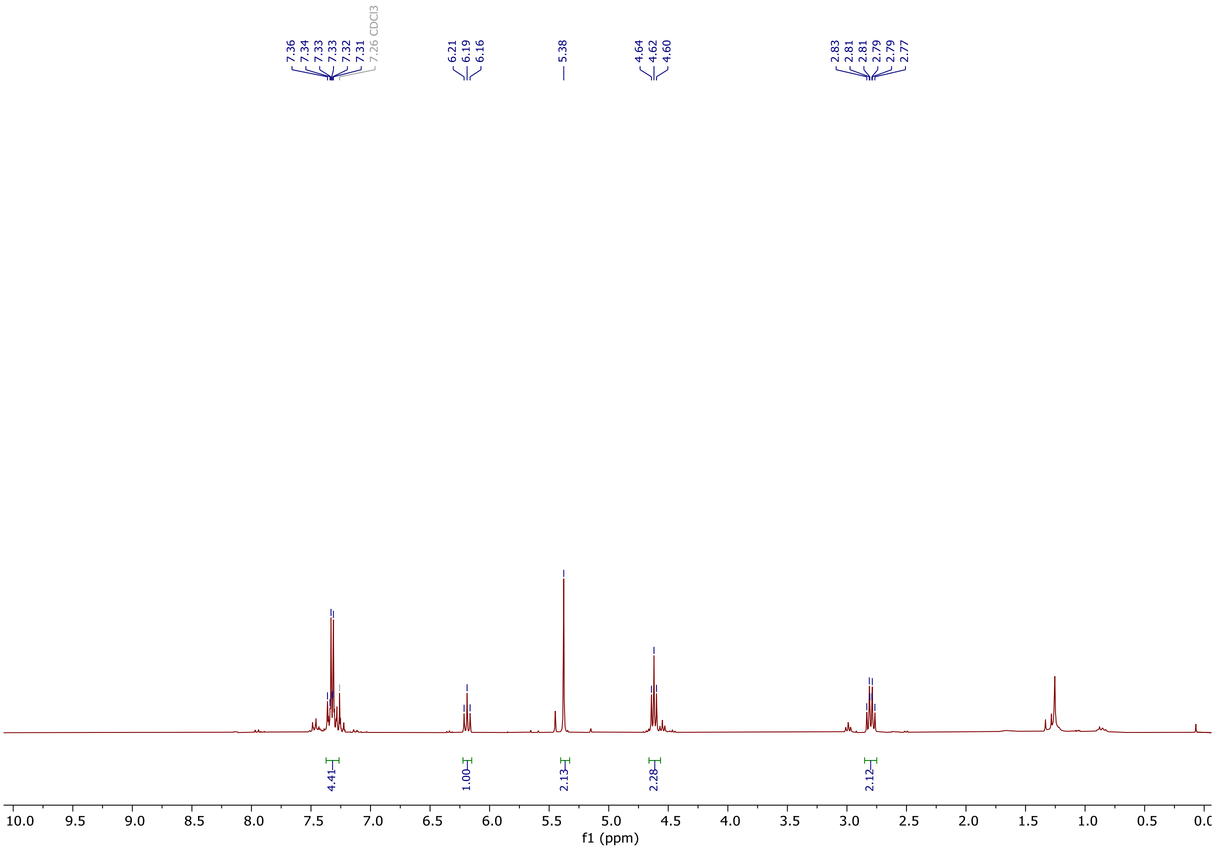

6. NMR Data

1-(4-(tert-butyl)phenyl)-2-nitroethyl nitrate (1)[1]

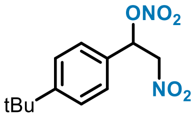

Compound **1** was obtained according to general procedure **GP1** from 1-(tert-butyl)-4-vinylbenzene (0.5 mmol, 1.0 equiv). Isolated as a yellow liquid (90% yield).

^{1}H-NMR (300 MHz, $CDCl_3$): δ 7.48 (d, *J* = 8.5 Hz, 2H), 7.36 (d, *J* = 8.4 Hz, 2H), 6.58 (dd, *J* = 10.2, 3.5 Hz, 1H), 4.86 (dd, *J* = 14.6, 10.2 Hz, 1H), 4.62 (dd, *J* = 14.6, 3.5 Hz, 1H), 1.33 (s, 9H).

^{13}C-NMR (126 MHz, $CDCl_3$): δ 154.0, 129.4, 126.7, 126.6, 79.8, 75.6, 35.0, 31.3.

2-nitro-1-phenylethyl nitrate (2)

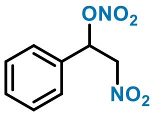

Compound **2** was obtained according to general procedure **GP1** from styrene (0.5 mmol, 1.0 equiv). Isolated as a yellow liquid (94% yield).

^{1}H-NMR (300 MHz, $CDCl_3$): δ 7.49 – 7.39 (m, 5H), 6.58 (dd, *J* = 10.1, 3.5 Hz, 1H), 4.84 (dd, *J* = 14.6, 10.1 Hz, 1H), 4.62 (dd, *J* = 14.6, 3.5 Hz, 1H).

^{13}C-NMR (75 MHz, $CDCl_3$): δ 132.6, 130.7, 130.6, 129.8, 129.7, 129.4, 129.2, 126.9, 79.8, 75.6.

IR (ATR, neat): 3060, 2971, 1662, 1371, 1280, 1214, 1163.

HRMS (ESI) *m/z*, calcd for $C_8H_8N_2O_5$–HNO_3: 149.0477; found 149.0475.

1-(4-formylphenyl)-2-nitroethyl nitrate (3)

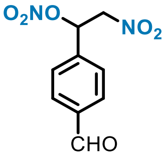

Compound **3** was obtained according to general procedure **GP1** from 4-vinylbenzaldehyde (0.5 mmol, 1.0 equiv). Isolated as a colourless liquid (81% yield).

^{1}H-NMR (300 MHz, $CDCl_3$): δ 10.05 (s, 1H), 7.98 (d, *J* = 7.8 Hz, 2H), 7.62 (d, *J* = 7.8 Hz, 2H), 6.64 (dd, *J* = 9.8, 3.6 Hz, 1H), 4.86 (dd, *J* = 14.6, 9.8 Hz, 1H), 4.65 (dd, *J* = 14.6, 3.6 Hz, 1H).

^{13}C-NMR (75 MHz, $CDCl_3$): δ 191.2, 138.7, 137.8, 130.8, 127.5, 78.9, 75.3.

IR (ATR, neat): 3012, 2853, 1738, 1647, 1616, 1547, 1464, 1233, 1056.

HRMS (ESI) *m/z,* calcd for $C_9H_8N_2O_6$–HNO_3: 177.0426; found 177.0424.

2-methyl-3-nitroprop-1-ene (5)[6]

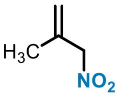

Compound **5** was obtained according to general procedure **GP1** from ((2-methylallyl)sulfonyl)benzene (0.5 mmol, 1.0 equiv). Isolated as a colourless liquid (53% yield).

^{1}H-NMR (300 MHz, $CDCl_3$): δ 5.27 – 5.15 (m, 2H), 4.89 (d, J = 1.1 Hz, 2H), 1.90 – 1.87 (m, 3H).

3,3-dimethyl-1-nitrobutan-2-yl nitrate (7)

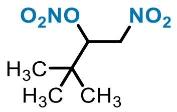

Compound **7** was obtained according to general procedure **GP1** from 3,3-dimethylbut-1-ene (0.5 mmol, 1.0 equiv). Isolated as a colourless liquid (65% yield).

^{1}H-NMR (300 MHz, $CDCl_3$): δ 5.23 (dd, J = 15.7, 10.7 Hz, 1H), 5.00 (dd, J = 10.7, 2.0 Hz, 1H), 4.68 (dd, J = 15.7, 2.0 Hz, 1H), 1.11 (s, 9H).

^{13}C-NMR (75 MHz, $CDCl_3$): δ 91.7, 72.9, 34.6, 26.8.

IR (ATR, neat): 2843, 1637, 1536, 1326, 1216, 1186.

HRMS (ESI) *m/z*, calcd for $C_6H_{12}N_2O_5$–HNO_3: 129.0790; found 129.0788.

2-nitro-1-(p-tolyl)ethyl nitrate (9)

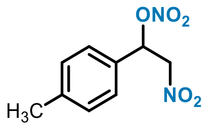

Compound **9** was obtained according to general procedure **GP1** from 1-methyl-4-vinylbenzene (0.5 mmol, 1.0 equiv). Isolated as yellow solid (92% yield).

^{1}H-NMR (300 MHz, $CDCl_3$): δ 7.28 (q, J = 8.1 Hz, 4H), 6.53 (dd, J = 10.1, 3.6 Hz, 1H), 4.83 (dd, J = 14.5, 10.1 Hz, 1H), 4.58 (dd, J = 14.5, 3.7 Hz, 1H), 2.37 (s, 3H).

^{13}C-NMR (75 MHz, $CDCl_3$): δ 140.9, 130.3, 129.6, 126.9, 79.8, 77.2, 75.7, 21.4.

IR (ATR, neat): 3014, 2983, 1626, 1566, 1347, 1237, 1147.

HRMS (ESI) *m/z*, calcd for $C_9H_{10}N_2O_5$–HNO_3: 163.0633; found 163.0631.

2-nitro-1-(o-tolyl)ethyl nitrate (10)

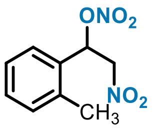

Compound **10** was obtained according to general procedure **GP1** from 1-methyl-2-vinylbenzene (0.5 mmol, 1.0 equiv). Isolated as a light-yellow liquid (89% yield).

^{1}H-NMR (300 MHz, $CDCl_3$): δ 7.32 – 7.15 (m, 4H), 6.74 (dd, J = 10.2, 3.2 Hz, 1H), 4.70 (dd, J = 14.7, 10.2 Hz, 1H), 4.45 (dd, J = 14.7, 3.2 Hz, 1H), 2.43 (s, 3H).

^{13}C-NMR (75 MHz, $CDCl_3$): δ 135.7, 131.5, 131.0, 130.2, 127.3, 125.8, 77.0, 75.0, 19.0.

IR (ATR, neat): 3003, 2973, 2868, 1626, 1552, 1237, 1136.

HRMS (ESI) *m/z*, calcd for $C_9H_{10}N_2O_5$–HNO_3: 163.0633; found 163.0631.

1-(2,4-dimethylphenyl)-2-nitroethyl nitrate (11)

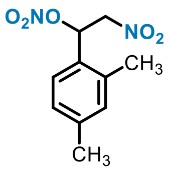

Compound **11** was obtained according to general procedure **GP1** from 2,4-dimethyl-1-vinylbenzene (0.5 mmol, 1.0 equiv). Isolated as a light-yellow liquid (91% yield).

^{1}H-NMR (300 MHz, $CDCl_3$): δ 7.25 (d, *J* = 7.8 Hz, 1H), 7.09 (d, *J* = 8.6 Hz, 2H), 6.78 (dd, *J* = 10.1, 3.3 Hz, 1H), 4.78 (dd, *J* = 14.6, 10.2 Hz, 1H), 4.51 (dd, *J* = 14.6, 3.3 Hz, 1H), 2.47 (s, 3H), 2.33 (s, 3H).

^{13}C-NMR (75 MHz, $CDCl_3$): δ 140.4, 135.6, 132.2, 128.0, 125.9, 77.2, 77.1, 75.2, 21.2, 18.9.

IR (ATR, neat): 3012, 2822, 1636, 1466, 1383, 1282, 1153, 741.

HRMS (ESI) *m/z*, calcd for $C_{10}H_{12}N_2O_5$–HNO_3: 177.0790; found 177.0788.

1-mesityl-2-nitroethyl nitrate (12)

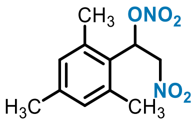

Compound **12** was obtained according to general procedure **GP1** from 1,3,5-trimethyl-2-vinylbenzene (0.5 mmol, 1.0 equiv). Isolated as a yellow liquid (88% yield).

^{1}H-NMR (300 MHz, $CDCl_3$): δ 7.07 (dd, *J* = 10.4, 3.3 Hz, 1H), 6.90 (s, 2H), 5.08 (dd, *J* = 14.7, 10.4 Hz, 1H), 4.54 (dd, *J* = 14.7, 3.3 Hz, 1H), 2.47 (s, 6H), 2.28 (s, 3H).

^{13}C-NMR (75 MHz, $CDCl_3$): δ 140.1, 137.1, 130.9, 125.8, 77.5, 74.3, 21.0, 20.4.

IR (ATR, neat): 3030, 2937, 1634, 1465, 1353, 1147,1047.

HRMS (ESI) *m/z*, calcd for $C_{11}H_{14}N_2O_5$–HNO_3: 191.0941; found 191.0943.

1-(4-isopropylphenyl)-2-nitroethyl nitrate (13)

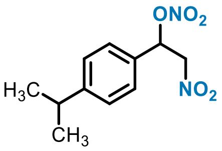

Compound **13** was obtained according to general procedure **GP1** from 1-isopropyl-4-vinylbenzene (0.5 mmol, 1.0 equiv). Isolated as a light-yellow liquid (91% yield).

^{1}H-NMR (400 MHz, $CDCl_3$): δ 7.34 (d, *J* = 8.6 Hz, 2H), 7.30 (d, *J* = 8.6 Hz, 2H), 6.56 (dd, *J* = 10.2, 3.5 Hz, 1H), 4.84 (dd, *J* = 14.6, 10.2 Hz, 1H), 4.60 (dd, *J* = 14.6, 3.5 Hz, 1H), 2.93 (p, *J* = 6.9 Hz, 1H), 1.25 (d, *J* = 6.9 Hz, 6H).

^{13}C-NMR (101 MHz, $CDCl_3$): δ 151.7, 129.9, 127.7, 127.0, 79.8, 75.7, 34.1, 23.9.

IR (ATR, neat): 3016, 2968, 1638, 1538, 1384, 1243, 1127.

HRMS (ESI) *m/z*, calcd for $C_{11}H_{14}N_2O_5$–HNO_3: 191.0946; found 191.0943.

1-(4-chlorophenyl)-2-nitroethyl nitrate (14)

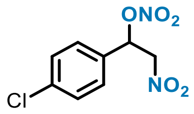

Compound **14** was obtained according to general procedure **GP1** from 1-chloro-4-vinylbenzene (0.5 mmol, 1.0 equiv). Isolated as a light-yellow liquid (84% yield).

^{1}H-NMR (300 MHz, $CDCl_3$): δ 7.46 – 7.35 (m, 4H), 6.54 (dd, *J* = 9.9, 3.7 Hz, 1H), 4.82 (dd, *J* = 14.6, 9.9 Hz, 1H), 4.60 (dd, *J* = 14.6, 3.7 Hz, 1H).

^{13}C-NMR (75 MHz, $CDCl_3$): δ 136.8, 131.1, 130.4, 130.0, 129.9, 128.3, 79.0, 75.4.

IR (ATR, neat): 3016, 2993, 1583, 1437, 1343, 1258, 1148.

HRMS (ESI) *m/z*, calcd for $C_8H_7ClN_2O_5$–HNO_3: 183.0087; found 183.0085.

1-(4-bromophenyl)-2-nitroethyl nitrate (15)

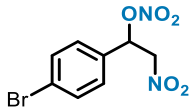

Compound **15** was obtained according to general procedure **GP1** from 1-bromo-4-vinylbenzene (0.5 mmol, 1.0 equiv). Isolated as a yellow solid (85% yield).

^{1}H-NMR (300 MHz, $CDCl_3$): δ 7.60 (d, *J* = 8.5 Hz, 2H), 7.31 (d, *J* = 8.4 Hz, 2H), 6.53 (dd, *J* = 9.9, 3.7 Hz, 1H), 4.82 (dd, *J* = 14.6, 9.9 Hz, 1H), 4.60 (dd, *J* = 14.6, 3.8 Hz, 1H).

^{13}C-NMR (75 MHz, $CDCl_3$): δ 132.9, 131.6, 130.5, 128.5, 125.0, 79.0, 75.3.

IR (ATR, neat): 3024, 2974, 1548, 1494, 1359, 1348, 1258 1083.

HRMS (ESI) *m/z*, calcd for $C_8H_7BrN_2O_5$–HNO_3: 226.9582; found 226.9580.

1-(4-fluorophenyl)-2-nitroethyl nitrate (16)

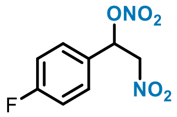

Compound **16** was obtained according to general procedure **GP1** from 1-fluoro-4-vinylbenzene (0.5 mmol, 1.0 equiv). Isolated as a light-yellow liquid (88% yield).

^{1}H-NMR (300 MHz, $CDCl_3$): δ 7.43 (dd, *J* = 8.7, 5.0 Hz, 2H), 7.15 (t, *J* = 8.6 Hz, 2H), 6.56 (dd, *J* = 9.9, 3.8 Hz, 1H), 4.84 (dd, *J* = 14.5, 9.9 Hz, 1H), 4.60 (dd, *J* = 14.5, 3.8 Hz, 1H).

^{13}C-NMR (75 MHz, $CDCl_3$): δ 163.9 (d, *J* = 251.0 Hz), 129.1 (d, *J* = 8.7 Hz), 128.5, 116.9 (d, *J* = 22.1 Hz), 79.0, 75.5.

^{19}F-NMR (282 MHz, $CDCl_3$): δ -109.4.

IR (ATR, neat): 3031, 2921, 1635, 1557, 1377, 1266, 1085.

HRMS (ESI) *m/z*, calcd for $C_8H_7FN_2O_5$–HNO_3: 167.0383; found 167.0382.

1-(2-fluorophenyl)-2-nitroethyl nitrate (17)

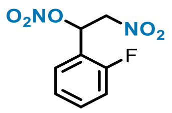

Compound **17** was obtained according to general procedure **GP1** from 1-fluoro-2-vinylbenzene (0.5 mmol, 1.0 equiv). Isolated as a yellow liquid (83% yield).

^{1}H-NMR (300 MHz, $CDCl_3$): δ 7.59 – 7.48 (m, 2H), 7.38 – 7.23 (m, 2H), 6.95 (dd, *J* = 9.9, 3.4 Hz, 1H), 4.99 (dd, *J* = 14.8, 9.9 Hz, 1H), 4.79 (dd, *J* = 14.8, 3.4 Hz, 1H).

^{13}C-NMR (75 MHz, $CDCl_3$): δ 161.6, 158.3, 132.3 (d, *J* = 8.5 Hz), 128.1 (d, *J* = 2.8 Hz), 125.2 (d, *J* = 3.7 Hz), 119.9 (d, *J* = 13.1 Hz), 116.5 (d, *J* = 20.8 Hz), 78.1 – 76.01 (m), 74.3 (dd, *J* = 10.7, 2.7 Hz).

^{19}F-NMR (282 MHz, $CDCl_3$): δ -116.6.

IR (ATR, neat): 3026, 2983, 1637, 1538, 1373, 1265, 1167.

HRMS (ESI) *m/z*, calcd for $C_8H_7FN_2O_5$–HNO_3: 167.0383; found 167.0381.

1-(3-formylphenyl)-2-nitroethyl nitrate (18)

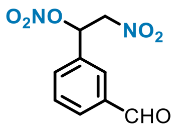

Compound **18** was obtained according to general procedure **GP1** from 3-vinylbenzaldehyde (0.5 mmol, 1.0 equiv). Isolated as a colourless liquid (87% yield).

^{1}H-NMR (300 MHz, $CDCl_3$): δ 10.06 (s, 1H), 8.01 – 7.93 (m, 2H), 7.76 – 7.61 (m, 2H), 6.66 (dd, *J* = 9.8, 3.8 Hz, 1H), 4.88 (dd, *J* = 14.6, 9.8 Hz, 1H), 4.66 (dd, *J* = 14.6, 3.8 Hz, 1H).

^{13}C-NMR (75 MHz, $CDCl_3$): δ 191.0, 137.5, 134.1, 132.5, 132.0, 130.6, 127.4, 78.9, 75.4.

IR (ATR, neat): 3027, 2883, 2737, 1692, 1547, 1373, 1164.

HRMS (ESI) *m/z*, calcd for $C_9H_8N_2O_6$–HNO_3: 177.0426; found 177.0424.

1-(3-methoxyphenyl)-2-nitroethyl nitrate (19)

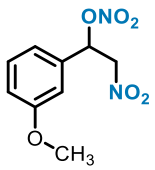

Compound **19** was obtained according to general procedure **GP1** from 1-methoxy-3-vinylbenzene (0.5 mmol, 1.0 equiv). Isolated as a light-yellow liquid (74% yield).

^{1}H-NMR (300 MHz, $CDCl_3$): δ 7.36 (t, *J* = 7.9 Hz, 1H), 7.04 – 6.89 (m, 3H), 6.54 (dd, *J* = 10.1, 3.5 Hz, 1H), 4.82 (dd, *J* = 14.6, 10.1 Hz, 1H), 4.60 (dd, *J* = 14.6, 3.5 Hz, 1H), 3.83 (s, 3H).

^{13}C-NMR (75 MHz, $CDCl_3$): δ 160.5, 134.1, 130.9, 118.8, 115.8, 112.5, 79.7, 75.7, 55.6.

IR (ATR, neat): 3012, 2947, 1616, 1548, 1384, 1238, 1037.

HRMS (ESI) *m/z*, calcd for $C_9H_{10}N_2O_6$–HNO_3: 179.0582; found 179.0580.

1-(4-(chloromethyl)phenyl)-2-nitroethyl nitrate (20)

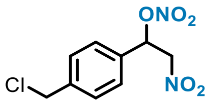

Compound **20** was obtained according to general procedure **GP1** from 1-(chloromethyl)-4-vinylbenzene (0.5 mmol, 1.0 equiv). Isolated as a yellow liquid (91% yield).

^{1}H-NMR (300 MHz, $CDCl_3$): δ 7.51 – 7.41 (m, 4H), 6.58 (dd, *J* = 10.0, 3.6 Hz, 1H), 4.83 (dd, *J* = 14.6, 10.0 Hz, 1H), 4.61 (d, *J* = 13.4 Hz, 3H).

^{13}C-NMR (75 MHz, $CDCl_3$): δ 140.1, 132.7, 129.8, 127.3, 79.3, 75.5, 45.3.

IR (ATR, neat): 3035, 2973, 1536, 1516, 1374, 1274, 1137.

HRMS (ESI) *m/z*, calcd for $C_9H_9ClN_2O_5$–HNO_3: 197.0244; found 197.0243.

1-(2,4-dichlorophenyl)-2-nitroethyl nitrate (21)

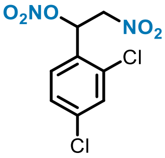

Compound **21** was obtained according to general procedure **GP1** from 2,4-dichloro-1-vinylbenzene (0.5 mmol, 1.0 equiv). Isolated as a colourless liquid (88% yield).

^{1}H-NMR (300 MHz, $CDCl_3$): δ 7.50 (dd, *J* = 1.9, 0.4 Hz, 1H), 7.45 – 7.34 (m, 2H), 6.95 (dd, *J* = 7.0, 5.9 Hz, 1H), 4.72 – 4.66 (m, 2H).

^{13}C-NMR (75 MHz, $CDCl_3$): δ 136.9, 133.1, 130.4, 129.2, 128.5, 127.9, 77.1, 75.9, 73.8.

IR (ATR, neat): 2986, 1658, 1536, 1384, 122, 1136.

HRMS (ESI) *m/z*, calcd for $C_8H_6Cl_2N_2O_5$–HNO_3: 216.9697; found 216.9695.

2-nitro-1-(4-(4,4,5,5-tetramethyl-1,3,2-dioxaborolan-2-yl)phenyl)ethyl nitrate (22)

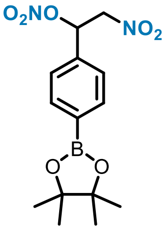

Compound **22** was obtained according to general procedure **GP1** from 4,4,5,5-tetramethyl-2-(4-vinylphenyl)-1,3,2-dioxaborolane (0.5 mmol, 1.0 equiv). Isolated as a colourless liquid (87% yield).

^{1}H-NMR (300 MHz, $CDCl_3$): δ 7.89 (d, *J* = 8.2 Hz, 2H), 7.46 – 7.38 (m, 2H), 6.57 (dd, *J* = 10.1, 3.5 Hz, 1H), 4.82 (dd, *J* = 14.6, 10.1 Hz, 1H), 4.59 (dd, *J* = 14.6, 3.5 Hz, 1H), 1.34 (s, 12H).

^{13}C-NMR (75 MHz, $CDCl_3$): δ 136.0, 135.2, 126.0, 84.4, 79.8, 75.5, 25.0.

IR (ATR, neat): 2980, 2933, 1612, 1542, 1352, 1126, 1064, 739.

HRMS (ESI) *m/z*, calcd for $C_{14}H_{19}BN_2O_7$–HNO_3: 275.1329; found 275.1327.

4-(2-nitro-1-(nitrooxy)ethyl)phenyl acetate (23)

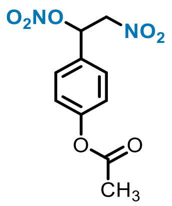

Compound **23** was obtained according to general procedure **GP1** from 4-vinylphenyl acetate (0.5 mmol, 1.0 equiv). Isolated as a light-yellow liquid (88% yield).

^{1}H-NMR (300 MHz, $CDCl_3$): δ 7.50 (d, *J* = 8.6 Hz, 2H), 7.25 (d, *J* = 8.7 Hz, 2H), 6.63 (dd, *J* = 10.1, 3.5 Hz, 1H), 4.88 (dd, *J* = 14.7, 10.1 Hz, 1H), 4.66 (dd, *J* = 14.7, 3.5 Hz, 1H), 2.37 (s, 3H).

^{13}C-NMR (75 MHz, $CDCl_3$): δ 169.2, 152.3, 130.0, 128.2, 123.0, 79.1, 75.5, 21.2.

IR (ATR, neat): 3003, 1659, 1578, 1573, 1483, 1374, 1125.

HRMS (ESI) *m/z*, calcd for $C_{10}H_{10}N_2O_7$–HNO_3: 207.0532; found 207.0530.

1-(4-methoxyphenyl)-2-nitropropyl nitrate (24)

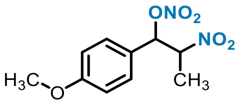

Compound **24** was obtained according to general procedure **GP1** from (Z)-1-methoxy-4-(prop-1-en-1-yl)benzene (0.5 mmol, 1.0 equiv). Isolated as a colorless liquid (72% yield).

^{1}H-NMR (300 MHz, $CDCl_3$): δ 7.33 (d, *J* = 8.7 Hz, 2H), 6.96 (d, *J* = 8.7 Hz, 2H), 6.17 (d, *J* = 10.2 Hz, 1H), 4.87 (dq, *J* = 10.2, 6.9 Hz, 1H), 3.83 (s, 3H), 1.37 (d, *J* = 6.9 Hz, 3H).

^{13}C-NMR (75 MHz, $CDCl_3$): δ 160.7, 129.5, 126.0, 114.6, 87.2, 79.3, 55.5, 16.5.

IR (ATR, neat): 2963, 1636, 1564, 1473, 1395, 1328, 1107, 1008.

HRMS (ESI) *m/z*, calcd for $C_{12}H_{10}N_2O_5$–HNO_3: 193.0739; found 193.0737.

2-nitro-1-(3-nitrophenyl)ethyl nitrate (25)

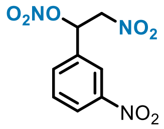

Compound **25** was obtained according to general procedure **GP1** from 1-nitro-3-vinylbenzene (0.5 mmol, 1.0 equiv). Isolated as a yellow liquid (83% yield).

^{1}H-NMR (300 MHz, $CDCl_3$): δ 8.38 – 8.30 (m, 2H), 7.84 – 7.77 (m, 1H), 7.70 (t, *J* = 8.2 Hz, 1H), 6.68 (dd, *J* = 9.7, 3.9 Hz, 1H), 4.89 (dd, *J* = 14.6, 9.7 Hz, 1H), 4.68 (dd, *J* = 14.7, 3.9 Hz, 1H).

^{13}C-NMR (75 MHz, $CDCl_3$): δ 142.6, 134.9, 132.8, 131.0, 125.5, 122.0, 78.2, 75.1.

IR (ATR, neat): 2993, 1645, 1632, 1435, 1385, 1271, 1045.

HRMS (ESI) *m/z*, calcd for $C_8H_7N_3O_7$–HNO_3: 194.0328; found 194.0324.

1-([1,1'-biphenyl]-4-yl)-2-nitroethyl nitrate (26)

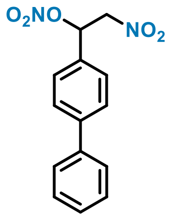

Compound **26** was obtained according to general procedure **GP2** from 4-vinyl-1,1'-biphenyl (0.5 mmol, 1.0 equiv). Isolated as a light-yellow liquid (82% yield).

^{1}H-NMR (300 MHz, $CDCl_3$): δ 7.70 – 7.64 (m, 2H), 7.61 – 7.55 (m, 2H), 7.53 – 7.39 (m, 5H), 6.63 (dd, *J* = 10.0, 3.6 Hz, 1H), 4.89 (dd, *J* = 14.6, 10.1 Hz, 1H), 4.65 (dd, *J* = 14.6, 3.6 Hz, 1H).

^{13}C-NMR (75 MHz, $CDCl_3$): δ 143.6, 139.9, 131.3, 129.1, 128.3, 128.2, 127.4, 127.3, 79.7, 75.6, 48.6, 24.9, 11.9.

IR (ATR, neat): 3016, 2984, 1626, 1573, 1368, 1233, 1068.

HRMS (ESI) *m/z*, calcd for $C_{14}H_{12}N_2O_5$–HNO_3: 225.0790; found 225.0788.

2-nitro-1-(4-((nitrooxy)methyl)phenyl)ethyl nitrate (27)

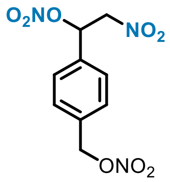

Compound **27** was obtained according to general procedure **GP1** from 4-vinylbenzyl nitrate (0.5 mmol, 1.0 equiv). Isolated as a light-yellow liquid (90% yield).

^{1}H-NMR (300 MHz, $CDCl_3$): δ 7.49 (t, *J* = 2.3 Hz, 4H), 6.59 (dd, *J* = 10.0, 3.6 Hz, 1H), 5.44 (s, 2H), 4.83 (dd, *J* = 14.6, 10.0 Hz, 1H), 4.66 – 4.58 (m, 1H).

^{13}C-NMR (75 MHz, $CDCl_3$): δ 135.0, 133.8, 130.03, 127.4, 79.2, 75.4, 73.7.

IR (ATR, neat): 3030, 2974, 1651, 1493, 1381, 1226, 1174.

HRMS (ESI) *m/z*, calcd for $C_9H_9N_3O_8$–HNO_3: 224.0433; found 224.0431.

1-(naphthalen-1-yl)-2-nitroethyl nitrate (28)

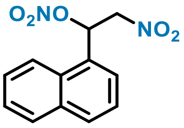

Compound **28** was obtained according to general procedure **GP2** from 1-vinylnaphthalene (0.5 mmol, 1.0 equiv). Isolated as a light-yellow liquid (81% yield).

^{1}H-NMR (300 MHz, $CDCl_3$): δ 8.12 (dq, *J* = 8.6, 1.0 Hz, 1H), 8.01 – 7.89 (m, 2H), 7.72 – 7.49 (m, 4H), 7.40 (dd, *J* = 10.1, 3.0 Hz, 1H), 4.91 (dd, *J* = 14.8, 10.1 Hz, 1H), 4.74 (dd, *J* = 14.8, 3.0 Hz, 1H).

^{13}C-NMR (75 MHz, $CDCl_3$): δ 134.0, 130.9, 129.7, 129.6, 128.4, 128.0, 126.8, 125.5, 124.5, 121.6, 77.3, 75.2.

IR (ATR, neat): 3018, 3001, 2987, 1627, 1568, 1237, 1182.

HRMS (ESI) *m/z*, calcd for $C_{12}H_{10}N_2O_5$–HNO_3: 199.0633; found 199.0630.

1-(naphthalen-2-yl)-2-nitroethyl nitrate (29)

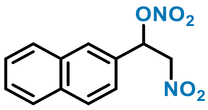

Compound **29** was obtained according to general procedure **GP2** from 1-vinylnaphthalene (0.5 mmol, 1.0 equiv). Isolated as a yellow liquid (81% yield).

^{1}H-NMR (300 MHz, $CDCl_3$): δ 7.98 – 7.84 (m, 4H), 7.62 – 7.54 (m, 2H), 7.46 (dd, *J* = 8.6, 1.8 Hz, 1H), 6.75 (dd, *J* = 10.1, 3.6 Hz, 1H), 4.94 (dd, *J* = 14.6, 10.1 Hz, 1H), 4.69 (dd, *J* = 14.6, 3.6 Hz, 1H).

^{13}C-NMR (75 MHz, $CDCl_3$): δ 134.1, 133.1, 129.9, 129.7, 128.3, 128.0, 127.7, 127.4, 127.1, 123.1, 80.0, 75.6.

IR (ATR, neat): 3036, 3005, 2994, 1626, 1573, 1227, 1147.

HRMS (ESI) *m/z*, calcd for $C_{12}H_{10}N_2O_5$–HNO_3: 199.0633; found 199.0630.

1-(anthracen-9-yl)-2-nitroethyl nitrate (30)

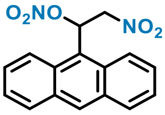

Compound **30** was obtained according to general procedure **GP2** from 9-vinylanthracene (0.5 mmol, 1.0 equiv). Isolated as a light-yellow liquid (78% yield).

^{1}H-NMR (300 MHz, $CDCl_3$): δ 8.63 (s, 1H), 8.16 – 8.00 (m, 4H), 7.65 (ddd, *J* = 8.8, 6.6, 1.5 Hz, 2H), 7.55 (ddd, *J* = 7.8, 6.6, 1.0 Hz, 2H), 7.06 (dd, *J* = 8.2, 3.2 Hz, 1H), 6.22 (dd, *J* = 13.2, 8.2 Hz, 1H), 5.00 (dd, *J* = 13.2, 3.2 Hz, 1H).

^{13}C-NMR (75 MHz, $CDCl_3$): δ 134.3, 131.8, 131.6, 130.2, 128.7, 127.4, 125.6, 122.1, 81.5, 71.2.

IR (ATR, neat): 3073, 3001, 2974, 1636, 1563, 1279.

HRMS (ESI) *m/z*, calcd for $C_{16}H_{12}N_2O_5$–HNO_3: 249.0790; found 249.0787.

2-nitro-1,1-diphenylethan-1-ol (31)[7]

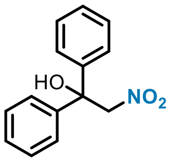

Compound **31** was obtained according to general procedure **GP1** from ethene-1,1-diyldibenzene (0.5 mmol, 1.0 equiv). Isolated as a white solid (57% yield).

^{1}H-NMR (300 MHz, $CDCl_3$): δ 7.48 – 7.42 (m, 4H), 7.41 – 7.28 (m, 6H), 5.09 (s, 2H), 4.61 (s, 1H).

^{13}C-NMR (75 MHz, $CDCl_3$): δ 142.0, 128.8, 128.34, 125.9, 82.7, 77.3.

2-nitro-1-(4-((nitrooxy)methyl)phenyl)ethyl nitrate (32)

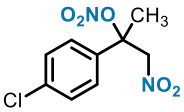

Compound **32** was obtained according to general procedure **GP1** from 1-chloro-4-(prop-1-en-2-yl)benzene (0.5 mmol, 1.0 equiv). Isolated as a light-yellow liquid (74% yield).

^{1}H-NMR (300 MHz, CDCl$_3$): δ 7.43 (d, J = 8.7 Hz, 2H), 7.33 (d, J = 8.8 Hz, 2H), 5.18 – 5.05 (m, 1H), 4.85 (d, J = 11.8 Hz, 1H), 2.04 (s, 3H).

^{13}C-NMR (75 MHz, CDCl$_3$): δ 136.6, 135.8, 129.8, 125.9, 86.2, 81.0, 23.4.

IR (ATR, neat): 2973, 1628, 1489, 1431, 1347, 1225, 1009.

HRMS (ESI) *m/z*, calcd for $C_9H_9ClN_2O_5$–HNO_3: 197.0244; found 197.0242.

1-nitro-2-phenylpropan-2-yl nitrate (33)

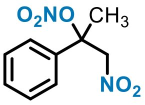

Compound **33** was obtained according to general procedure **GP1** from prop-1-en-2-ylbenzene (0.5 mmol, 1.0 equiv). Isolated as a light-yellow liquid (80% yield).

^{1}H-NMR (300 MHz, CDCl$_3$): δ 7.49 – 7.35 (m, 5H), 5.18 (d, J = 11.7 Hz, 1H), 4.87 (d, J = 11.7 Hz, 1H), 2.05 (s, 3H).

^{13}C-NMR (75 MHz, CDCl$_3$): δ 138.2, 129.6, 124.3, 86.7, 81.1, 23.5.

IR (ATR, neat): 3006, 2984, 2894, 1547, 1471, 1363, 1285.

HRMS (ESI) *m/z*, calcd for $C_9H_{10}N_2O_5$–HNO_3: 163.0633; found 163.0631.

2-nitro-2,3-dihydro-1H-inden-1-yl nitrate (34)

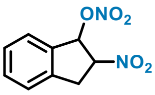

Compound **34** was obtained according to general procedure **GP1** from 1*H*-indene (0.5 mmol, 1.0 equiv). Isolated as a yellow liquid (82% yield).

Isomer 34a: ^{1}H-NMR (300 MHz, CDCl$_3$): δ 7.50 – 7.43 (m, 2H), 7.41 – 7.29 (m, 2H), 6.91 (d, J = 3.4 Hz, 1H), 5.34 (ddd, J = 8.6, 5.0, 3.5 Hz, 1H), 3.80 (dd, J = 17.2, 8.6 Hz, 1H), 3.59 (dd, J = 17.2, 5.0 Hz, 1H).

^{13}C-NMR (75 MHz, CDCl$_3$): δ 139.8, 133.3, 131.5, 128.7, 126.1, 125.2, 88.3, 88.2, 36.8.

Isomer 34b: ^{1}H-NMR (300 MHz, CDCl$_3$): δ 7.51 – 7.41 (m, 2H), 7.40 – 7.33 (m, 2H), 6.66 (d, J = 6.2 Hz, 1H), 5.50 (dt, J = 7.3, 6.3 Hz, 1H), 3.93 – 3.77 (m, 1H), 3.41 (dd, J = 16.6, 7.3 Hz, 1H).

^{13}C-NMR (75 MHz, CDCl$_3$): δ 139.7, 133.3, 131.7, 128.5, 126.3, 125.5, 84.3, 82.9, 34.0.

IR (ATR, neat): 3012, 2924, 2886, 1535, 1473, 1384.

HRMS (ESI) *m/z*, calcd for $C_9H_8N_2O_5$–HNO_3: 161.0477; found 161.0475.

ethyl 3-nitro-4-(nitrooxy)-4-phenylbutanoate (35)

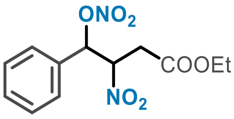

Compound **35** was obtained according to general procedure **GP1** from methyl (*E*)-4-phenylbut-3-enoate (0.5 mmol, 1.0 equiv). Isolated as a yellow liquid (78% yield).

^{1}H-NMR (300 MHz, $CDCl_3$): δ 7.44 – 7.30 (m, 5H), 5.57 (t, *J* = 3.7 Hz, 1H), 5.07 (dt, *J* = 10.4, 3.2 Hz, 1H), 3.64 (s, 3H), 3.31 (dd, *J* = 17.8, 10.4 Hz, 1H), 2.66 – 2.54 (m, 2H).

^{13}C-NMR (75 MHz, $CDCl_3$): δ 170.6, 138.2, 129.1, 129.0, 125.8, 88.0, 74.0, 52.5, 30.7.

IR (ATR, neat): 3004, 2996, 1702, 1648, 1552, 1387, 1295.

HRMS (ESI) *m/z*, calcd for $C_{11}H_{12}N_2O_7$–HNO_3: 221.0688; found 221.0685.

2-methyl-1-nitro-3-phenylpropan-2-yl nitrate (36)

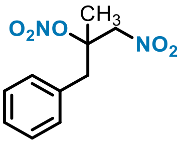

Compound **35** was obtained according to general procedure **GP1** from (2-methylallyl)benzene (0.5 mmol, 1.0 equiv). Isolated as a colourless liquid (71% yield).

^{1}H-NMR (300 MHz, $CDCl_3$): δ 7.37 (d, *J* = 7.6 Hz, 3H), 7.25 (dd, *J* = 7.4, 2.1 Hz, 2H), 4.98 (d, *J* = 12.0 Hz, 1H), 4.86 (d, *J* = 12.0 Hz, 1H), 3.31 (d, *J* = 14.2 Hz, 1H), 3.19 (d, *J* = 14.2 Hz, 1H), 1.59 (s, 3H).

^{13}C-NMR (75 MHz, $CDCl_3$): δ 133.00, 130.75, 128.99, 128.07, 87.51, 78.42, 41.79, 21.00.

IR (ATR, neat): 3065, 2930, 1656, 1634, 1497, 1456, 1435, 1385, 1263, 1180.

HRMS (ESI) *m/z*, calcd for $C_{10}H_{12}N_2O_5$–HNO_3: 177.0790; found 177.0787.

1-nitro-4-phenylbutan-2-yl nitrate (37)

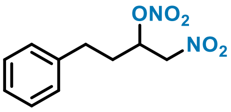

Compound **36** was obtained according to general procedure **GP1** from but-3-en-1-ylbenzene (0.5 mmol, 1.0 equiv). Isolated as a colourless liquid (57% yield).

^{1}H-NMR (300 MHz, $CDCl_3$): δ 7.39 – 7.26 (m, 3H), 7.23 – 7.16 (m, 2H), 5.72 – 5.58 (m, 1H), 4.64 – 4.53 (m, 2H), 2.82 (dt, *J* = 8.6, 6.7 Hz, 2H), 2.21 – 2.08 (m, 2H).

^{13}C-NMR (75 MHz, $CDCl_3$): δ 139.2, 129.0, 128.3, 127.0, 77.8, 75.6, 32.1, 31.0.

IR (ATR, neat): 3009, 2996, 1653, 1569, 1536, 1291.

HRMS (ESI) *m/z*, $[M+H]^+$ calcd for $C_{10}H_{12}N_2O_5$–HNO_3: 177.0790; found 177.0788.

1-cyclohexyl-2-nitroethyl nitrate (38)

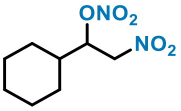

Compound **37** was obtained according to general procedure **GP1** from but-3-en-1-ylbenzene (0.5 mmol, 1.0 equiv). Isolated as a colourless liquid (57% yield).

^{1}H-NMR (300 MHz, $CDCl_3$): δ 5.32 – 5.12 (m, 1H), 5.06 (ddd, *J* = 10.5, 5.8, 2.0 Hz, 1H), 4.61 (dd, *J* = 15.5, 2.0 Hz, 1H), 2.03 (ddt, *J* = 11.8, 5.8, 2.9 Hz, 1H), 1.89 – 1.77 (m, 2H), 1.77 – 1.65 (m, 3H), 1.33 – 1.04 (m, 6H).

^{13}C-NMR (75 MHz, $CDCl_3$): δ 87.6, 72.8, 40.2, 29.3, 28.9, 25.8, 25.8, 25.6.

IR (ATR, neat): 2934, 1634, 1464, 1376, 1273, 1183, 1152, 1036.

HRMS (ESI) *m/z*, $[M+H]^+$ calcd for $C_8H_{14}N_2O_5$–HNO_3: 155.0946; found 155.0943.

tert-butyl 4-(nitromethyl)-4-(nitrooxy)piperidine-1-carboxylate (39)

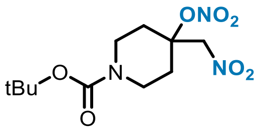

Compound **38** was obtained according to general procedure **GP2** from *tert*-butyl 4-methylenepiperidine-1-carboxylate (0.5 mmol, 1.0 equiv). Isolated as a colourless liquid (53% yield).

^{1}H-NMR (300 MHz, $CDCl_3$): δ 4.93 (br s, 2H), 3.95 (d, *J* = 11.7 Hz, 2H), 3.16 (t, *J* = 12.8 Hz, 2H), 2.27 (d, *J* = 14.5 Hz, 2H), 1.82 (ddd, *J* = 14.6, 11.5, 4.7 Hz, 2H), 1.46 (s, 9H).

^{13}C-NMR (75 MHz, $CDCl_3$): δ 154.4, 84.7, 80.7, 78.7, 31.6, 29.8, 28.5.

IR (ATR, neat): 2896, 1706, 1643, 1552, 1376, 1265.

HRMS (ESI) *m/z*, $[M+H]^+$ calcd for $C_{11}H_{19}N_3O_7$–HNO_3: 242.1267; found 242.1266.

1-nitrodecan-2-yl nitrate (40)

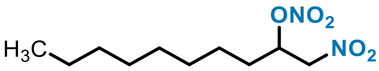

Compound **39** was obtained according to general procedure **GP1** from dec-1-ene (0.5 mmol, 1.0 equiv). Isolated as a colourless liquid (79% yield).

^{1}H-NMR (300 MHz, $CDCl_3$): δ 5.31 – 5.07 (m, 2H), 4.64 – 4.51 (m, 1H), 2.09 – 1.80 (m, 2H), 1.42 – 1.20 (m, 12H), 0.92 – 0.83 (m, 3H).

^{13}C-NMR (75 MHz, $CDCl_3$): δ 82.7, 74.1, 31.8, 31.3, 29.2, 29.1, 28.9, 25.4, 22.7, 14.2.

IR (ATR, neat): 2842, 2874, 1548, 1456, 1378, 1237.

HRMS (ESI) *m/z*, $[M+H]^+$ calcd for $C_{10}H_{20}N_2O_5$–HNO_3: 185.1416; found 185.1414.

4-(2-nitro-1-(nitrooxy)ethyl)benzyl (2S)-2-(4-isobutylphenyl)propanoate (41)

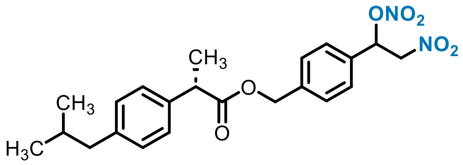

Compound **40** was obtained according to general procedure **GP2** from (*S*)-Ibuprofen (0.5 mmol, 1.0 equiv). Isolated as a yellow solid (77% yield).

^{1}H-NMR (300 MHz, $CDCl_3$): δ 7.37 (d, *J* = 8.3 Hz, 2H), 7.30 (d, *J* = 8.3 Hz, 2H), 7.23 (d, *J* = 8.2 Hz, 2H), 7.13 (d, *J* = 8.1 Hz, 2H), 6.58 (dd, *J* = 10.0, 3.6 Hz, 1H), 5.18 – 5.10 (m, 2H), 4.83 (dd, *J* = 14.6,

10.1 Hz, 1H), 4.60 (dd, J = 14.6, 3.6 Hz, 1H), 3.80 (q, J = 7.1 Hz, 1H), 2.50 (d, J = 7.2 Hz, 2H), 1.89 (dt, J = 13.5, 6.7 Hz, 1H), 1.55 (d, J = 7.2 Hz, 3H), 0.94 (d, J = 6.6 Hz, 6H).

^{13}C-NMR (75 MHz, $CDCl_3$): δ 174.5, 140.9, 138.9, 137.5, 132.2, 129.5, 128.6, 127.3, 127.0, 79.4, 75.5, 65.40, 45.2, 45.1, 30.3, 22.5, 18.4.

IR (ATR, neat): 3011, 2996, 2964, 1714, 1555, 1254.

HRMS (ESI) *m/z*, calcd for $C_{22}H_{26}N_2O_7$–HNO_3: 367.1784; found 367.1782.

2-(3,5,5,6,8,8-hexamethyl-5,6,7,8-tetrahydronaphthalen-2-yl)-1-nitropropan-2-yl nitrate (42)

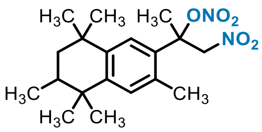

Compound **41** was obtained according to general procedure **GP2** from 1,1,2,4,4,7-hexamethyl-6-(prop-1-en-2-yl)-1,2,3,4-tetrahydronaphthalene (0.5 mmol, 1.0 equiv). Isolated as a colorless liquid (84% yield).

^{1}H-NMR (300 MHz, $CDCl_3$): δ 7.33 (d, J = 3.0 Hz, 1H), 7.12 (s, 1H), 5.00 (dd, J = 12.6, 1.5 Hz, 1H), 4.66 (dd, J = 12.6, 1.2 Hz, 1H), 2.52 (s, 3H), 1.85 (dqd, J = 13.3, 6.7, 2.5 Hz, 1H), 1.72 (s, 3H), 1.62 (t, J = 13.2 Hz, 1H), 1.40 – 1.33 (m, 2H), 1.31 (d, J = 0.9 Hz, 3H), 1.27 (d, J = 5.4 Hz, 3H), 1.23 (d, J = 4.4 Hz, 3H), 1.05 (s, 3H), 0.98 (d, J = 6.8 Hz, 3H).

^{13}C-NMR (75 MHz, $CDCl_3$): δ 146.1, 146.0, 142.7, 142.7, 136.9, 136.9, 132.1, 131.8, 131.8, 123.8, 123.8, 83.9, 83.9, 74.8, 74.7, 43.8, 37.4, 34.6, 34.3, 32.5, 32.4, 32.1, 32.0, 28.5, 28.5, 26.7, 26.7, 25.0, 22.1, 16.9.

IR (ATR, neat): 3002, 2985, 1636, 1526, 1375, 1267, 1036.

HRMS (ESI) *m/z*, $[M+H]^+$ calcd for $C_{19}H_{28}N_2O_5$–HNO_3: 301.2042; found 301.2040.

3-(nitromethyl)-3-phenylisobenzofuran-1(3*H*)-one (43)[1]

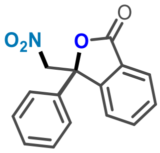

Compound **43** was obtained according to general procedure **GP2** from 2-(1-phenylvinyl)benzoic acid (0.5 mmol, 1.0 equiv). Isolated as a white solid (77% yield).

^{1}H-NMR (300 MHz, $CDCl_3$): δ 8.03 – 7.88 (m, 1H), 7.80 – 7.73 (m, 2H), 7.67 – 7.53 (m, 3H), 7.49 – 7.38 (m, 3H), 5.26 (d, J = 13.1 Hz, 1H), 5.17 (d, J = 13.1 Hz, 1H).

3-(nitromethyl)-3-phenylisobenzofuran-1(3*H*)-one (trans) (44)[8]

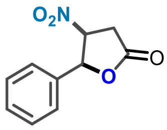

Compound **44** was obtained according to general procedure **GP2** from (E)-4-phenylbut-3-enoic acid (0.5 mmol, 1.0 equiv). Isolated as a yellow solid (79% yield).

^{1}H-NMR (300 MHz, $CDCl_3$): δ 7.45 (td, J = 6.2, 3.3 Hz, 3H), 7.39 – 7.31 (m, 2H), 5.97 (s, 1H), 5.16 (dt, J = 8.2, 2.9 Hz, 1H), 3.42 – 3.27 (m, 1H), 3.07 (dd, J = 18.8, 8.2 Hz, 1H).

^{13}C-NMR (75 MHz, $CDCl_3$): δ 171.6, 135.7, 129.8, 129.6, 124.9, 86.6, 82.7, 31.9.

(*Z*)-5-nitro-6-phenylhex-5-enoic acid (45)

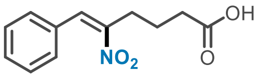

Compound **45** was obtained according to general procedure **GP2** from (*E*)-6-phenylhex-5-enoic acid (0.5 mmol, 1.0 equiv). Isolated as a colorless liquid (71% yield).

^{1}H-NMR (300 MHz, $CDCl_3$): δ 8.10 (s, 1H), 7.55 – 7.36 (m, 5H), 2.97 – 2.87 (m, 2H), 2.51 (t, *J* = 7.0 Hz, 2H), 2.07 – 1.94 (m, 2H).

^{13}C-NMR (75 MHz, $CDCl_3$): δ 179.2, 151.0, 134.7, 132.1, 130.4, 129.9, 129.2, 33.3, 26.7, 22.8.

IR (ATR, neat): 3306, 3005, 2996, 2953, 1674, 1575, 1336, 1274, 1063.

HRMS (ESI) *m/z*, calcd for $C_{12}H_{12}O_4N$: 234.0772; found 234.0770.

5-nitro-6-(nitrooxy)-6-phenylhexanoic acid (46)

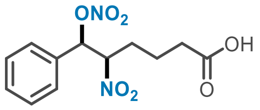

Compound **46** was obtained according to general procedure **GP2** from (*E*)-6-phenylhex-5-enoic acid (0.5 mmol, 1.0 equiv). Isolated as a yellow liquid (9% yield).

^{1}H-NMR (300 MHz, $CDCl_3$): δ 7.50 – 7.36 (m, 6H), 6.19 (d, *J* = 10.2 Hz, 1H), 4.82 (td, *J* = 10.4, 3.3 Hz, 1H), 2.27 (td, *J* = 7.1, 4.3 Hz, 2H), 1.95 – 1.83 (m, 1H), 1.58 (dq, *J* = 9.4, 6.7 Hz, 2H), 1.48 – 1.39 (m, 1H).

IR (ATR, neat): 3302, 3014, 2983, 1663, 1574, 1347, 1297.

HRMS (ESI) *m/z*, calcd for $C_{12}H_{13}O_7N2$: 297.0728; found 297.0730.

4,4-dimethyl-2-(nitromethyl)-1-tosylpyrrolidine (47)

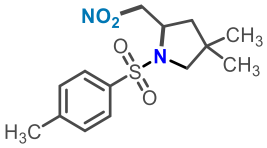

Compound **45** was obtained according to general procedure **GP2** from *N*-(2,2-dimethylpent-4-en-1-yl)-4-methylbenzenesulfonamide (0.5 mmol, 1.0 equiv). Isolated as a colorless liquid (68% yield).

^{1}H-NMR (300 MHz, $CDCl_3$): δ 7.75 (d, *J* = 8.3 Hz, 2H), 7.41 – 7.30 (m, 2H), 5.18 (dd, *J* = 12.8, 4.2 Hz, 1H), 4.48 (dd, *J* = 12.8, 8.9 Hz, 1H), 4.15 (dtd, *J* = 8.9, 7.7, 4.2 Hz, 1H), 3.21 (d, *J* = 10.5 Hz, 1H), 3.05 (dd, *J* = 10.5, 1.3 Hz, 1H), 2.44 (s, 3H), 1.85 (ddd, *J* = 12.9, 7.5, 1.2 Hz, 1H), 1.70 – 1.62 (m, 1H), 1.06 (s, 3H), 0.51 (s, 3H).

^{13}C-NMR (75 MHz, $CDCl_3$): δ 144.4, 133.5, 130.1, 127.9, 79.8, 61.6, 56.8, 44.7, 37.7, 26.3, 25.6, 21.7.

IR (ATR, neat): 3002, 2994, 2875, 1624, 1567, 1297, 1092.

HRMS (ESI) *m/z*, calcd for $C_{14}H_{20}N_2O_4S$: 312.1144; found 312.1142.

(*E*)-1-(tert-butyl)-4-(2-nitrovinyl)benzene (48)[1]

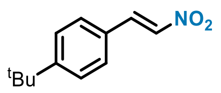

Compound **42** was obtained according to general procedure **GP3** from 1-chloro-4-(prop-1-en-2-yl)benzene (0.5 mmol, 1.0 equiv). Isolated as a yellow liquid (85% yield).

^{1}H-NMR (300 MHz, $CDCl_3$): δ 8.00 (d, *J* = 13.7 Hz, 1H), 7.58 (d, *J* = 13.7 Hz, 1H), 7.48 (d, *J* = 1.5 Hz, 4H), 1.34 (s, 9H).

(*E*)-1-chloro-4-(1-nitroprop-1-en-2-yl)benzene (49)[9]

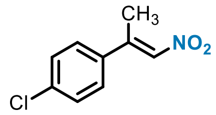

Compound **43** was obtained according to general procedure **GP3** from 1-chloro-4-(prop-1-en-2-yl)benzene (0.5 mmol, 1.0 equiv). Isolated as a colourless liquid (74% yield).

^{1}H-NMR (300 MHz, $CDCl_3$): δ 7.44 – 7.36 (m, 4H), 7.28 (d, *J* = 1.5 Hz, 1H), 2.62 (d, *J* = 1.5 Hz, 3H).

^{13}C-NMR (75 MHz, $CDCl_3$): δ 148.6, 136.8, 136.8, 136.6, 129.5, 128.3, 18.6.

(*E*)-3-(2-nitrovinyl)benzaldehyde (50)[10]

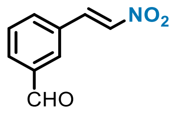

Compound **44** was obtained according to general procedure **GP3** from 3-vinylbenzaldehyde (0.5 mmol, 1.0 equiv). Isolated as a colourless liquid (83% yield).

^{1}H-NMR (300 MHz, $CDCl_3$): δ 10.07 (s, 1H), 8.11 – 7.96 (m, 3H), 7.85 – 7.77 (m, 1H), 7.72 – 7.61 (m, 2H).

^{13}C-NMR (75 MHz, $CDCl_3$): δ 191.1, 138.6, 137.5, 137.4, 134.5, 133.0, 131.3, 130.4, 129.7.

2-nitro-1*H*-indene (51)[2]

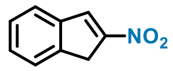

Compound **45** was obtained according to general procedure **GP3** from 1*H*-indene (0.5 mmol, 1.0 equiv). Isolated as a colorless liquid (81% yield).

^{1}H-NMR (300 MHz, $CDCl_3$): δ 7.92 (td, *J* = 2.0, 0.7 Hz, 1H), 7.65 – 7.59 (m, 1H), 7.55 – 7.38 (m, 4H), 3.99 (d, *J* = 1.9 Hz, 2H).

^{13}C-NMR (75 MHz, $CDCl_3$): δ 153.5, 141.9, 139.5, 136.4, 130.1, 128.0, 125.4, 124.8, 36.7.

(*E*)-4,4,5,5-tetramethyl-2-(4-(2-nitrovinyl)phenyl)-1,3,2-dioxaborolane (52)[11]

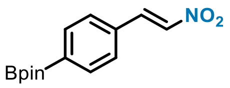

Compound **46** was obtained according to general procedure **GP3** from 4,4,5,5-tetramethyl-2-(4-vinylphenyl)-1,3,2-dioxaborolane (0.5 mmol, 1.0 equiv). Isolated as a colorless liquid (75% yield).

^{1}H-NMR (300 MHz, $CDCl_3$): δ 8.00 (d, *J* = 13.7 Hz, 1H), 7.87 (d, *J* = 8.2 Hz, 2H), 7.61 (d, *J* = 13.7 Hz, 1H), 7.56 – 7.49 (m, 2H), 1.36 (s, 12H).

^{13}C-NMR (75 MHz, $CDCl_3$): δ 139.0, 137.8, 135.7, 132.6, 128.4, 84.4, 25.0.

(*E*)-1-chloro-4-(2-nitrovinyl)benzene (53)[2]

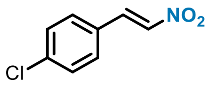

Compound **47** was obtained according to general procedure **GP3** from 1-chloro-4-vinylbenzene (0.5 mmol, 1.0 equiv). Isolated as a colourless liquid (86% yield).

^{1}H-NMR (300 MHz, $CDCl_3$): δ 7.97 (d, *J* = 13.7 Hz, 1H), 7.56 (d, *J* = 13.7 Hz, 1H), 7.49 (d, *J* = 8.6 Hz, 2H), 7.44 (d, *J* = 8.7 Hz, 2H).

tert-butyl 4-(nitromethylene)piperidine-1-carboxylate (54)

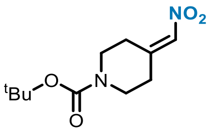

Compound **48** was obtained according to general procedure **GP3** from *tert*-butyl 4-methylenepiperidine-1-carboxylate (0.5 mmol, 1.0 equiv). Isolated as a yellow liquid (51% yield).

^{1}H-NMR (300 MHz, $CDCl_3$): δ 6.99 (t, *J* = 1.3 Hz, 1H), 3.56 (q, *J* = 5.9 Hz, 4H), 2.99 (t, *J* = 6.2 Hz, 2H), 2.36 – 2.29 (m, 2H), 1.48 (s, 9H).

^{13}C-NMR (75 MHz, $CDCl_3$): δ 150.8, 134.1, 80.6, 78.8, 33.0, 29.9, 29.0, 28.5, 28.2.

IR (ATR, neat): 3012, 2973, 1796, 1717, 1655, 1573, 1538, 1353, 1266.

HRMS (ESI) *m/z*, calcd for $C_{11}H_{18}N_2O_4$: 242.1267; found 242.1265.

(*E*)-4-(2-nitrovinyl)benzaldehyde (55)[12]

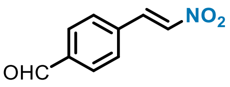

Compound **49** was obtained according to general procedure **GP3** from 4-vinylbenzaldehyde (0.5 mmol, 1.0 equiv). Isolated as a colourless liquid (86% yield).

^{1}H-NMR (300 MHz, $CDCl_3$): δ 10.07 (s, 1H), 8.03 (d, *J* = 13.7 Hz, 1H), 7.97 (d, *J* = 8.3 Hz, 2H), 7.72 (d, *J* = 8.3 Hz, 2H), 7.65 (d, *J* = 13.7 Hz, 1H).

^{13}C-NMR (75 MHz, $CDCl_3$): δ 191.2, 139.3, 138.5, 137.4, 135.7, 130.5, 129.7.

(*E*)-1-(2-nitrovinyl)naphthalene (56)[13]

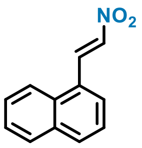

Compound **50** was obtained according to general procedure **GP3** from 1-vinylnaphthalene (0.5 mmol, 1.0 equiv). Isolated as a colourless liquid (68% yield).

^{1}H-NMR (300 MHz, $CDCl_3$): δ 8.85 (d, *J* = 13.4 Hz, 1H), 8.18 – 8.12 (m, 1H), 8.01 (d, *J* = 8.2 Hz, 1H), 7.96 – 7.90 (m, 1H), 7.79 – 7.74 (m, 1H), 7.69 – 7.56 (m, 3H), 7.56 – 7.49 (m, 1H).

^{13}C-NMR (75 MHz, $CDCl_3$): δ 138.7, 136.3, 133.9, 132.7, 131.7, 129.2, 127.9, 127.2, 127.0, 126.5, 125.6, 123.1.

(*E*)-1-nitro-3-(2-nitrovinyl)benzene (57)[7]

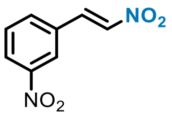

Compound **51** was obtained according to general procedure **GP3** from 1-nitro-3-vinylbenzene (0.5 mmol, 1.0 equiv). Isolated as a colourless liquid (72% yield).

^{1}H-NMR (300 MHz, $CDCl_3$): δ 8.42 (t, *J* = 2.0 Hz, 1H), 8.38 – 8.32 (m, 1H), 8.06 (d, *J* = 13.7 Hz, 1H), 7.91 – 7.85 (m, 1H), 7.72 – 7.64 (m, 2H).

^{13}C-NMR (75 MHz, $CDCl_3$): δ 139.4, 136.4, 134.6, 131.9, 130.7, 126.3, 123.6.

(*E*)-(2-nitroprop-1-en-1-yl)benzene (58)[1]

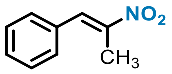

Compound **52** was obtained according to general procedure **GP3** from 1-nitro-3-vinylbenzene (0.5 mmol, 1.0 equiv). Isolated as a colourless liquid (70% yield).

^{1}H-NMR (300 MHz, $CDCl_3$): δ 8.10 (d, *J* = 1.2 Hz, 1H), 7.47 – 7.41 (m, 5H), 2.46 (d, *J* = 1.1 Hz, 3H).

ethyl-2-nitro-3-phenylacrylate (59)[14]

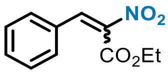

Compound **52** was obtained according to general procedure **GP3** from ethyl cinnamate (0.5 mmol, 1.0 equiv). Isolated as a yellow oil (81% yield).

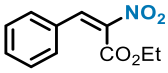

***E*-59: ^{1}H-NMR** (300 MHz, $CDCl_3$): δ 8.08 (s, 1H), 7.54 – 7.41 (m, 5H), 4.44 (q, *J* = 7.2 Hz, 2H), 1.35 (t, *J* = 7.2 Hz, 3H).

***E*-59: ^{13}C-NMR** (75 MHz, $CDCl_3$): δ 161.3, 136.7, 132.5, 130.6, 129.9, 129.4, 129.1, 63.3, 13.9.

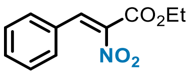

***Z*-59: ^{1}H-NMR** (300 MHz, $CDCl_3$): δ 7.54 (s, 1H), 7.49 – 7.40 (m, 5H), 4.38 (q, *J* = 7.1 Hz, 2H), 1.35 (d, *J* = 7.1 Hz, 3H).

***Z*-59: ^{13}C-NMR** (75 MHz, $CDCl_3$): δ 159.3, 136.7, 133.0, 132.3, 129.9, 129.5, 129.1, 63.2, 14.2.

(*E*)-1-(7,7-dimethyl-2-oxobicyclo[2.2.1]heptan-1-yl)-N-(4-(2-nitrovinyl)phenyl)methanesulfonamide (60)[1]

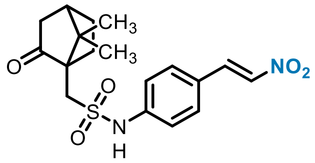

Compound **53** was obtained according to general procedure **GP3** from 1-(7,7-dimethyl-2-oxobicyclo[2.2.1]heptan-1-yl)-*N*-(4-vinylphenyl)methanesulfonamide (0.5 mmol, 1.0 equiv). Isolated as a solid (62% yield).

^{1}H-NMR (300 MHz, $CDCl_3$): δ 10.13 (s, 1H), 8.45 (d, *J* = 2.2 Hz, 1H), 8.20 (d, *J* = 8.8 Hz, 1H), 7.97 (d, *J* = 13.7 Hz, 1H), 7.85 (dd, *J* = 8.9, 2.2 Hz, 1H), 7.61 (d, *J* = 13.7 Hz, 1H), 3.68 (d, *J* = 14.9 Hz, 1H), 3.16 (d, *J* = 14.9 Hz, 1H), 2.47 – 2.26 (m, 2H), 2.18 – 2.04 (m, 2H), 1.93 (d, *J* = 18.6 Hz, 1H), 1.89 – 1.79 (m, 1H), 1.50 (ddd, *J* = 13.1, 9.2, 3.9 Hz, 1H), 1.07 (s, 3H), 0.84 (s, 3H).

^{13}C-NMR (75 MHz, $CDCl_3$): δ 215.2, 138.2, 137.6, 136.2, 136.0, 135.5, 127.5, 124.9, 119.9, 60.5, 58.8, 51.2, 48.8, 42.9, 42.6, 27.1, 25.5, 21.2, 19.9, 19.6, 14.3.

(*E*)-4-(2-nitrovinyl)benzyl (S)-2-(4-isobutylphenyl)propanoate (61)

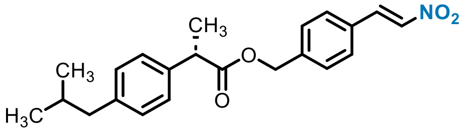

Compound **54** was obtained according to general procedure **GP3** from (*S*)-Ibuprofen (0.5 mmol, 1.0 equiv). Isolated as a yellow liquid (59% yield).

^{1}H-NMR (300 MHz, $CDCl_3$): δ 7.97 (d, *J* = 13.7 Hz, 1H), 7.55 (d, *J* = 13.7 Hz, 1H), 7.46 (d, *J* = 8.2 Hz, 2H), 7.26 (d, *J* = 8.4 Hz, 3H), 7.20 (d, *J* = 8.1 Hz, 2H), 7.10 (d, *J* = 8.2 Hz, 2H), 5.14 (d, *J* = 4.8 Hz, 2H), 3.77 (q, *J* = 7.2 Hz, 1H), 2.46 (d, *J* = 7.2 Hz, 2H), 1.85 (dt, *J* = 13.5, 6.6 Hz, 1H), 1.52 (d, *J* = 7.2 Hz, 3H), 0.91 (d, *J* = 6.6 Hz, 6H).

^{13}C-NMR (75 MHz, $CDCl_3$): δ 174.5, 140.9, 140.7, 138.6, 137.5, 137.4, 129.8, 129.5, 129.3, 128.4, 127.4, 65.5, 45.3, 45.2, 30.4, 22.5, 18.4.

IR (ATR, neat): 3014, 2927, 1693, 1636, 1557, 1374, 1274.

HRMS (ESI) *m/z*, calcd for $C_{22}H_{25}NO_4$: 367.1784; found 367.1782.

methyl (*E*)-3,3-dimethyl-5-nitropent-4-enoate (62)[15]

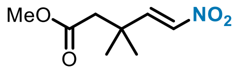

Compound **55** was obtained according to general procedure **GP4** from 3,3-dimethylpent-4-enoic acid (0.5 mmol, 1.0 equiv). Isolated as a colorless liquid (61% yield).

^{1}H-NMR (300 MHz, $CDCl_3$): δ 7.37 (d, *J* = 13.6 Hz, 1H), 6.93 (d, *J* = 13.6 Hz, 1H), 3.66 (s, 3H), 2.45 (s, 2H), 1.25 (s, 7H).

1-(tert-butyl)-4-(1-chloro-2-nitroethyl)benzene (63)[1]

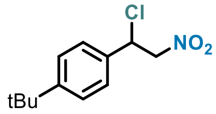

Compound **55** was obtained according to general procedure **GP4** from 1-(tert-butyl)-4-vinylbenzene (0.5 mmol, 1.0 equiv). Isolated as a colorless liquid (63% yield).

^{1}H-NMR (400 MHz, $CDCl_3$): δ 7.43 (d, *J* = 8.5 Hz, 2H), 7.36 (d, *J* = 8.5 Hz, 2H), 5.56 (dd, *J* = 9.2, 5.4 Hz, 1H), 4.91 (dd, *J* = 13.4, 9.2 Hz, 1H), 4.78 (dd, *J* = 13.4, 5.5 Hz, 1H), 1.33 (s, 9H).

1-chloro-4-(1-chloro-2-nitroethyl)benzene (64)[16]

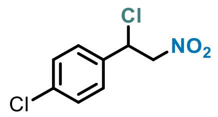

Compound **56** was obtained according to general procedure **GP4** from 1-chloro-4-vinylbenzene (0.5 mmol, 1.0 equiv). Isolated as a colorless liquid (58% yield).

^{1}H-NMR (300 MHz, $CDCl_3$): δ 7.38 (s, 4H), 5.60 – 5.47 (m, 1H), 4.89 (ddd, *J* = 13.5, 8.7, 1.2 Hz, 1H), 4.76 (ddd, *J* = 13.5, 6.1, 1.2 Hz, 1H).

^{13}C-NMR (75 MHz, $CDCl_3$): δ 136.0, 134.5, 129.7, 128.7, 80.7, 56.1.

4-(1-chloro-2-nitroethyl)benzaldehyde (65)[12]

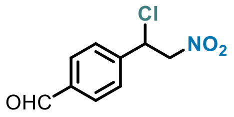

Compound **57** was obtained according to general procedure **GP4** from 4-vinylbenzaldehyde (0.5 mmol, 1.0 equiv). Isolated as a colourless liquid (60% yield).

^{1}H-NMR (300 MHz, $CDCl_3$): δ 10.03 (s, 1H), 7.93 (d, *J* = 8.3 Hz, 2H), 7.62 (d, *J* = 8.2 Hz, 2H), 5.61 (dd, *J* = 8.7, 6.0 Hz, 1H), 4.93 (dd, *J* = 13.6, 8.7 Hz, 1H), 4.82 (dd, *J* = 13.6, 6.0 Hz, 1H).

1-(1-bromo-2-nitroethyl)-4-(tert-butyl)benzene (66)[1]

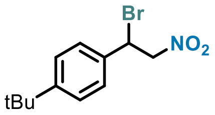

Compound **58** was obtained according to general procedure **GP5** from 1-(tert-butyl)-4-vinylbenzene (0.5 mmol, 1.0 equiv). Isolated as a yellow liquid (59% yield).

^{1}H-NMR (300 MHz, $CDCl_3$): δ 7.43 – 7.31 (m, 4H), 5.57 (dd, *J* = 8.2, 7.1 Hz, 1H), 4.99 (qd, *J* = 13.6, 7.7 Hz, 2H), 1.31 (s, 9H).

1-(1-bromo-2-nitroethyl)-4-chlorobenzene (67)

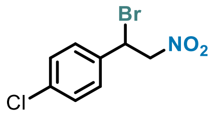

Compound **59** was obtained according to general procedure **GP5** from 1-chloro-4-vinylbenzene (0.5 mmol, 1.0 equiv). Isolated as a white solid (55% yield).

^{1}H-NMR (300 MHz, $CDCl_3$): δ 7.37 (s, 4H), 5.52 (t, *J* = 7.7 Hz, 1H), 5.08 – 4.87 (m, 2H).

^{13}C-NMR (75 MHz, $CDCl_3$): δ 135.9, 135.2, 129.7, 129.0, 80.4, 44.2.

1-(1-bromo-2-nitroethyl)-4-(chloromethyl)benzene (68)[1]

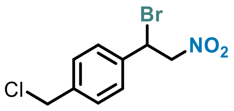

Compound **60** was obtained according to general procedure **GP5** from 1-(chloromethyl)-4-vinylbenzene (0.5 mmol, 1.0 equiv). Isolated as a yellow liquid (53% yield).

^{1}H-NMR (300 MHz, $CDCl_3$): δ 7.43 (s, 4H), 5.55 (t, *J* = 7.7 Hz, 1H), 5.10 – 4.88 (m, 2H), 4.57 (s, 2H).

2-azido-1-(4-(tert-butyl)phenyl)ethyl nitrate (69)

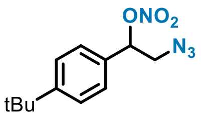

Compound **61** was obtained according to general procedure **GP6** from 1-(tert-butyl)-4-vinylbenzene (0.5 mmol, 1.0 equiv). Isolated as a yellow liquid (29% yield).

^{1}H-NMR (300 MHz, $CDCl_3$): δ 7.35 (d, *J* = 8.4 Hz, 2H), 7.23 (d, *J* = 8.4 Hz, 2H), 5.85 (dd, *J* = 8.7, 4.1 Hz, 1H), 3.66 (dd, *J* = 13.6, 8.7 Hz, 1H), 3.44 (dd, *J* = 13.6, 4.1 Hz, 1H), 1.24 (s, 9H).

^{13}C-NMR (75 MHz, $CDCl_3$): δ 153.1, 131.6, 126.5, 126.2, 83.5, 53.5, 31.3.

2-amino-1-(p-tolyl)ethan-1-ol (70)[17]

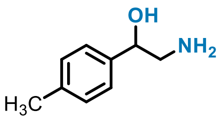

Compound **62** was obtained according to general procedure **GP7** from 1-methyl-4-vinylbenzene (0.5 mmol, 1.0 equiv). Isolated as a off-white solid (61% yield).

^{1}H-NMR (300 MHz, MeOH-d_4): δ 7.30 (d, *J* = 8.1 Hz, 2H), 7.19 (d, *J* = 7.9 Hz, 2H), 5.34 (dd, *J* = 9.6, 3.7 Hz, 1H), 4.72 – 4.51 (m, 2H), 2.33 (s, 3H).

^{13}C-NMR (75 MHz, MeOH-d_4): δ 139.3, 138.3, 130.3, 127.1, 82.8, 72.0, 21.1.

2-amino-1-(4-(tert-butyl)phenyl)ethan-1-ol (71)

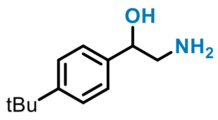

Compound **63** was obtained according to general procedure **GP7** from 1-(tert-butyl)-4-vinylbenzene (0.5 mmol, 1.0 equiv). Isolated as a white solid (69% yield).

^{1}H-NMR (300 MHz, $CDCl_3$): δ 7.37 (d, *J* = 8.4 Hz, 2H), 7.30 (s, 2H), 4.62 (dd, *J* = 7.8, 4.0 Hz, 1H), 3.00 (dd, *J* = 12.8, 4.0 Hz, 1H), 2.83 (dd, *J* = 12.8, 7.8 Hz, 1H), 2.17 (s, 3H), 1.31 (s, 9H).

^{13}C-NMR (75 MHz, $CDCl_3$): δ 150.7, 139.6, 125.8, 125.5, 74.3, 49.3, 34.7, 31.5.

IR (ATR, neat): 3363, 3032, 2935, 2873, 1572, 1451, 1204, 1062, 745.

HRMS (ESI) *m/z*, calcd for $C_{12}H_{19}NO+H$: 194.1539; found 194.1536.

tert-butyl (2-(4-(tert-butyl)phenyl)-2-hydroxyethyl)carbamate (72)[18]

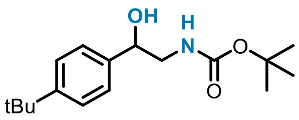

Compound **64** was obtained according to general procedure **GP7** from 1-(tert-butyl)-4-vinylbenzene (0.5 mmol, 1.0 equiv). Isolated as a white solid after two steps (43% yield).

^{1}**H-NMR** (300 MHz, $CDCl_3$): δ 7.38 (d, *J* = 8.6 Hz, 2H), 7.29 (d, *J* = 8.3 Hz, 2H), 4.97 (s, 1H), 4.79 (dd, *J* = 8.1, 3.6 Hz, 1H), 3.46 (d, *J* = 7.2 Hz, 1H), 3.26 (ddd, *J* = 13.8, 7.9, 4.9 Hz, 1H), 1.44 (s, 9H), 1.31 (s, 9H).

^{13}**C-NMR** (75 MHz, $CDCl_3$): δ 150.9, 138.9, 125.8, 125.6, 79.9, 73.9, 48.3, 34.7, 31.5, 28.5.

4-(p-tolyl)-1*H*-1,2,3-triazole (73)[19]

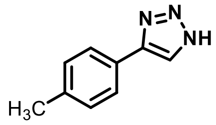

Compound **65** was obtained according to general procedure **GP8** from 1-methyl-4-vinylbenzene (0.5 mmol, 1.0 equiv). Isolated as a white solid (79% yield).

^{1}**H-NMR** (300 MHz, $CDCl_3$): δ 7.96 (s, 1H), 7.71 (d, *J* = 8.1 Hz, 2H), 7.27 (d, *J* = 7.9 Hz, 3H), 2.40 (s, 3H).

4-(1H-1,2,3-triazol-4-yl)phenol (74)[20]

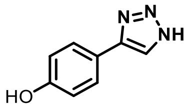

Compound **66** was obtained according to general procedure **GP8** from 4-vinylphenol (0.5 mmol, 1.0 equiv). Isolated as a yellow solid (86% yield).

^{1}**H-NMR** (300 MHz, DMSO-d_6): δ 9.61 (s, 1H), 8.13 (s, 1H), 7.65 (d, J = 8.6 Hz, 2H), 6.83 (d, J = 8.6 Hz, 2H).

4-cyclohexyl-1H-1,2,3-triazole (75)[21]

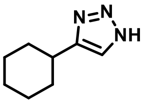

Compound **67** was obtained according to general procedure **GP8** from vinylcyclohexane (0.5 mmol, 1.0 equiv). Isolated as a colorless liquid (63% yield).

^{1}**H-NMR** (300 MHz, $CDCl_3$): δ 8.10 (s, 1H), 4.27 (dd, *J* = 5.7, 2.2 Hz, 1H), 1.73 (p, *J* = 6.0 Hz, 1H), 1.51 – 1.24 (m, 6H), 0.99 – 0.87 (m, 3H).

5. NMR Spectra of Isolated Compounds

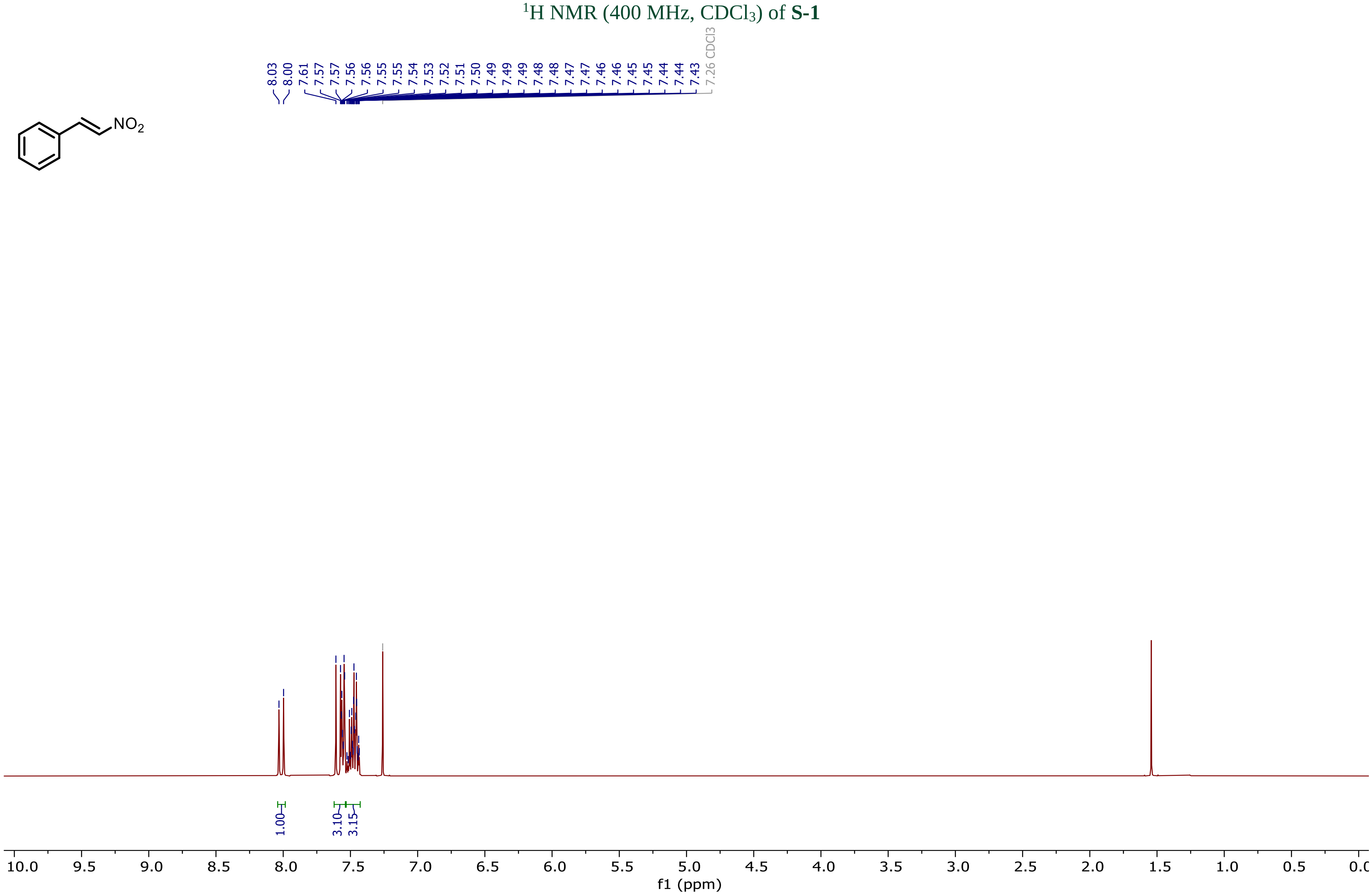

^{1}H NMR (300 MHz, $CDCl_3$) of **S-2**

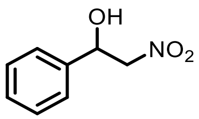

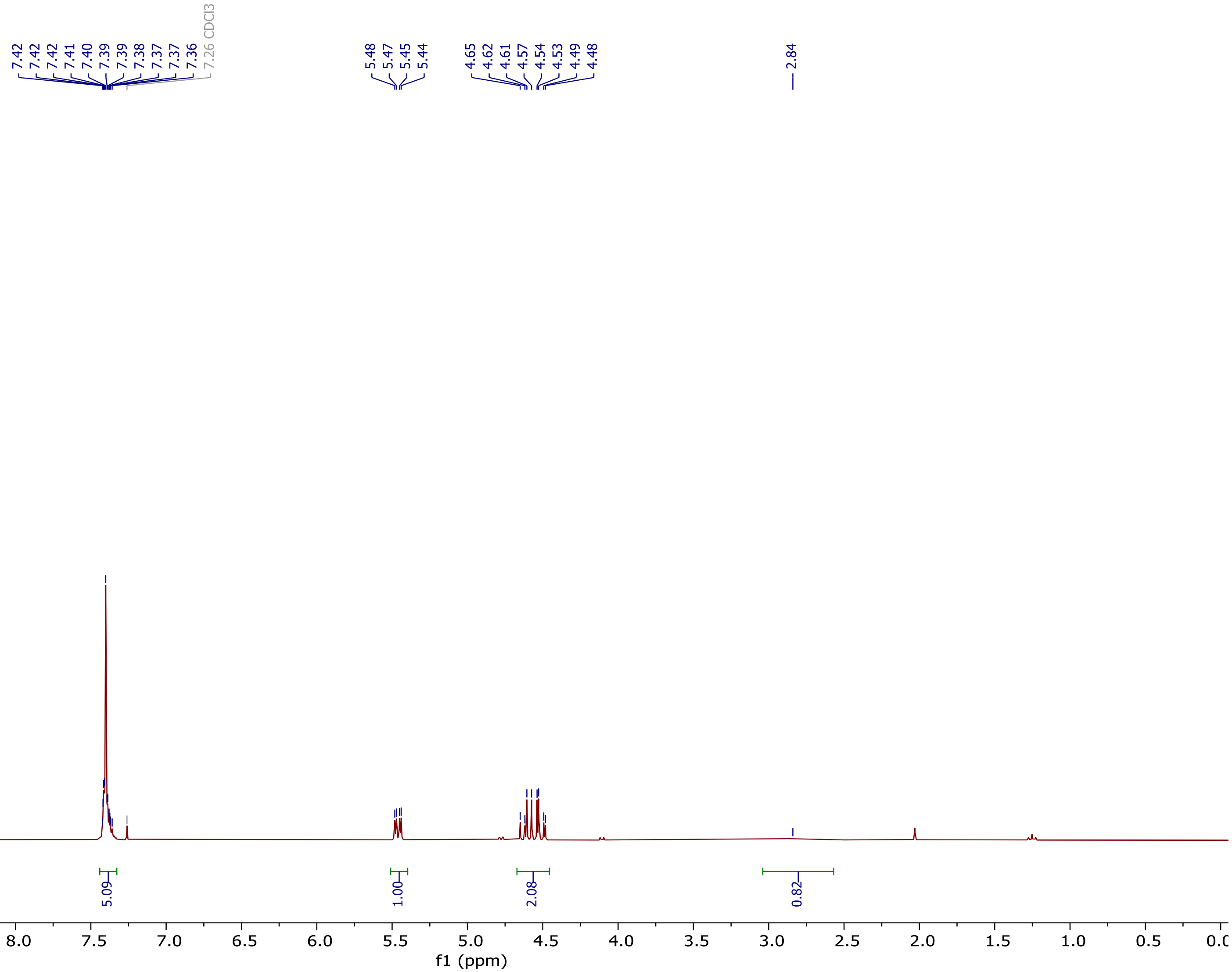

^{1}H NMR (400 MHz, $CDCl_3$) of **S-3**

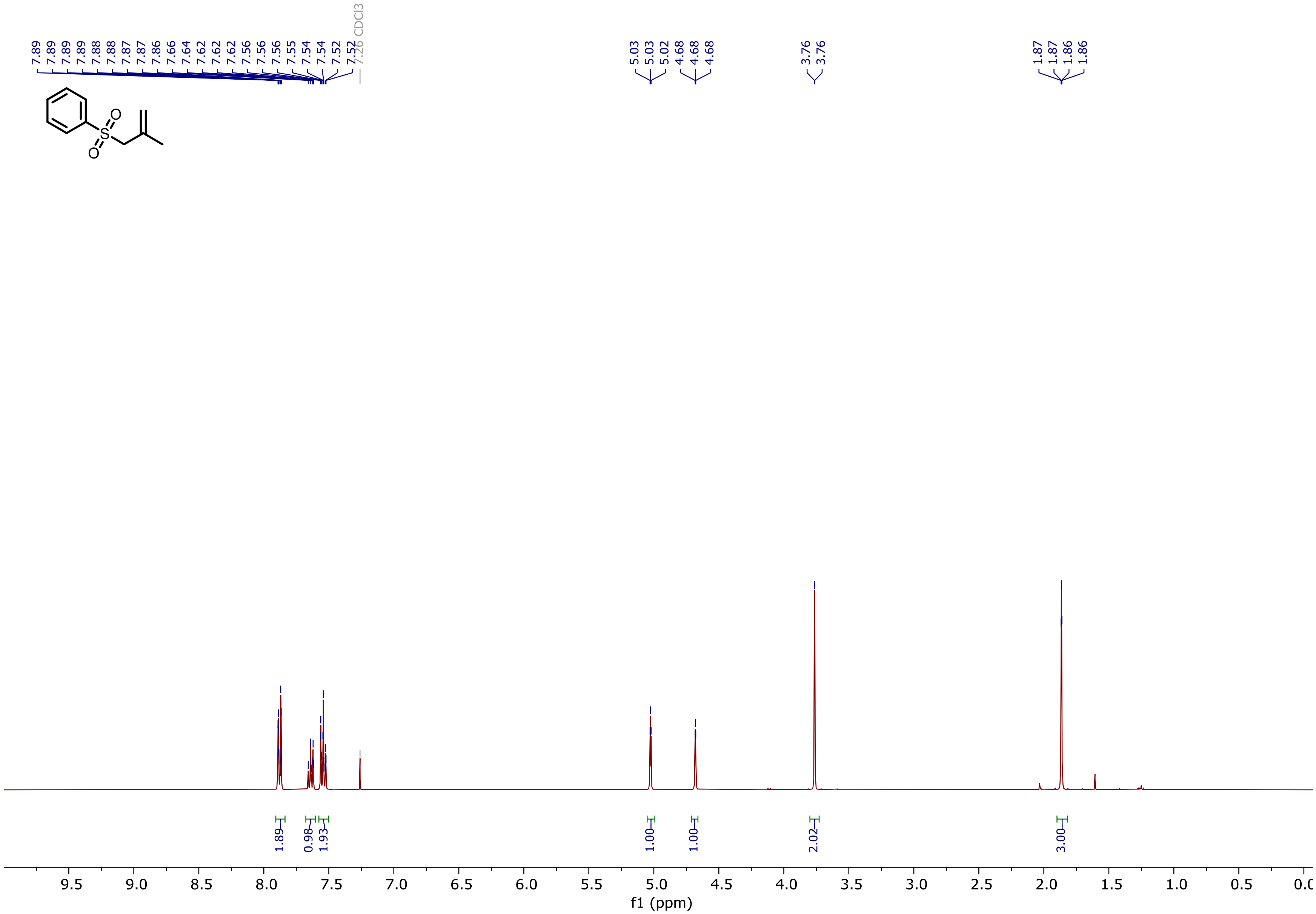

^{1}H NMR (300 MHz, $CDCl_3$) of **S-4**

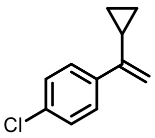

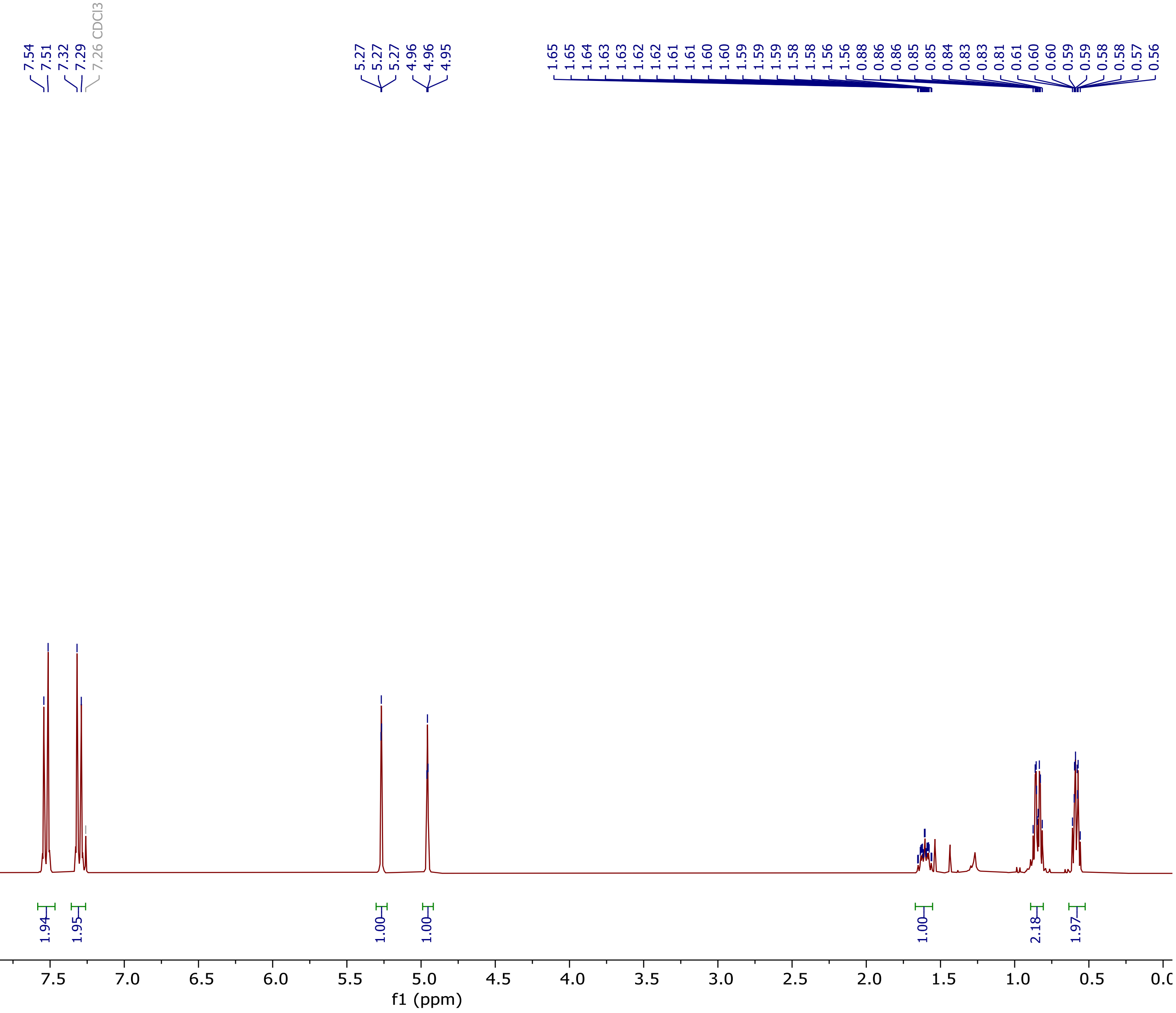

^{13}C NMR (75 MHz, $CDCl_3$) of **S-4**

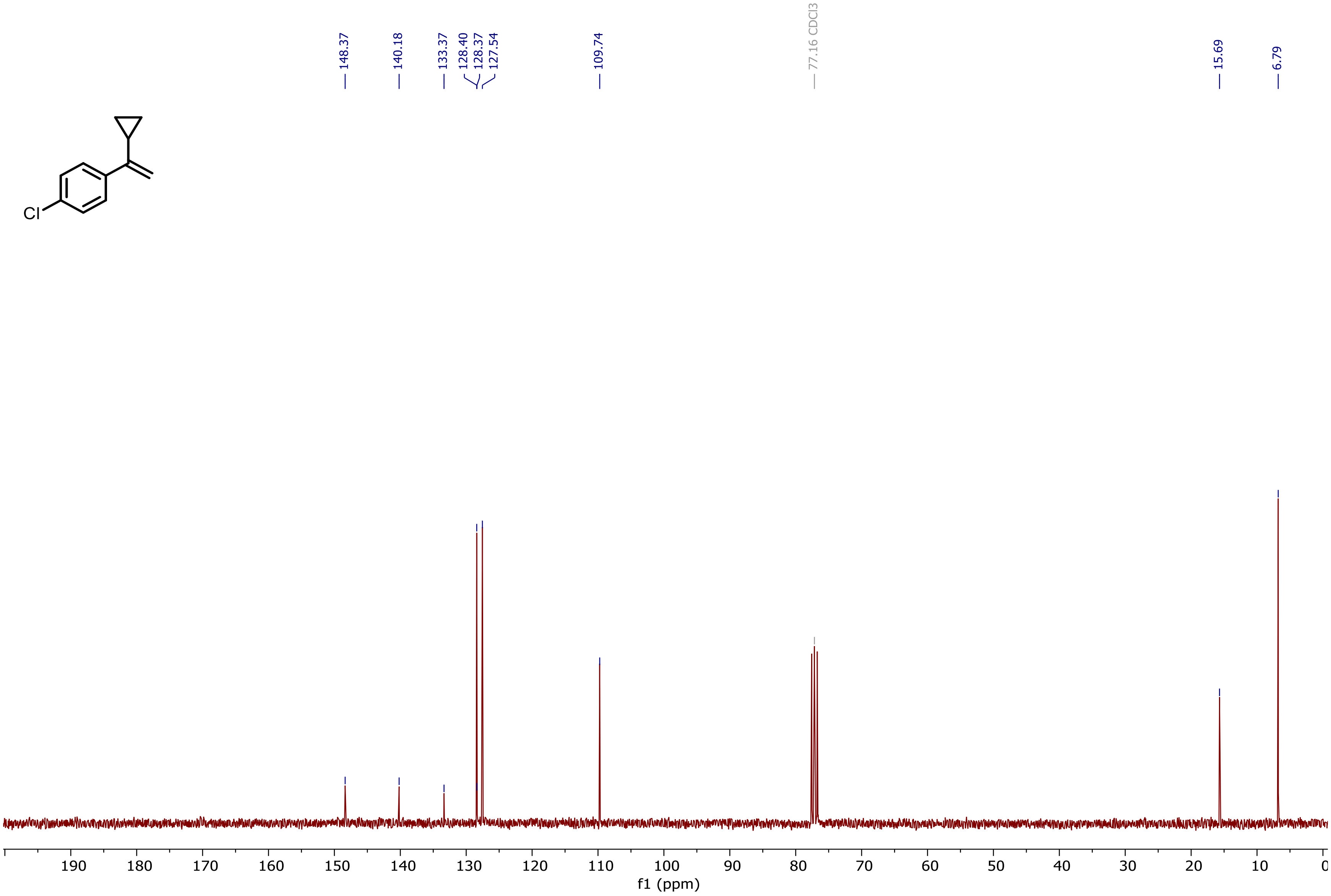

^{1}H NMR (300 MHz, $CDCl_3$) of **S-5**

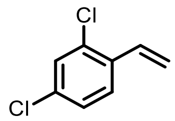

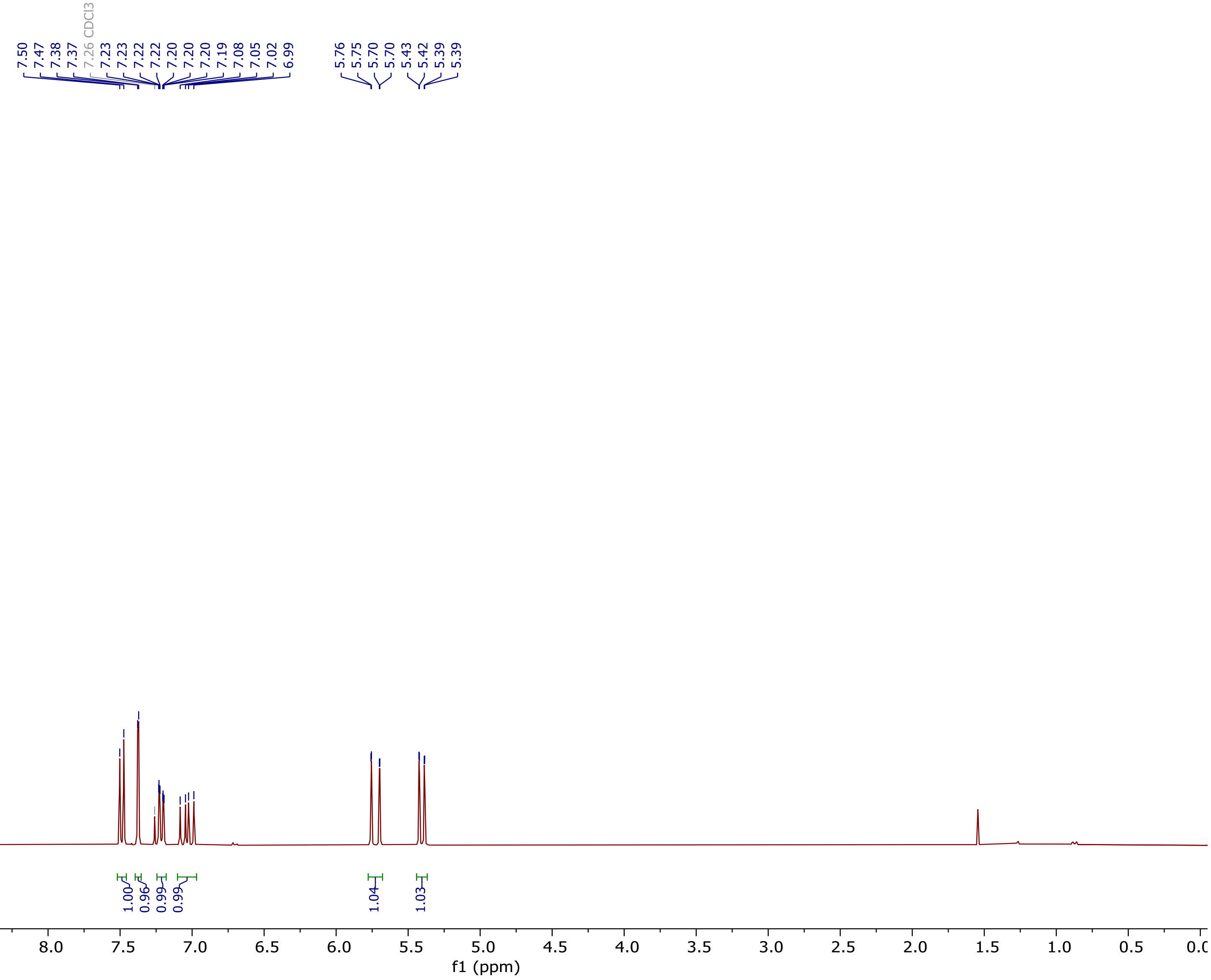

^{13}C NMR (75 MHz, $CDCl_3$) of **S-5**

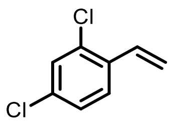

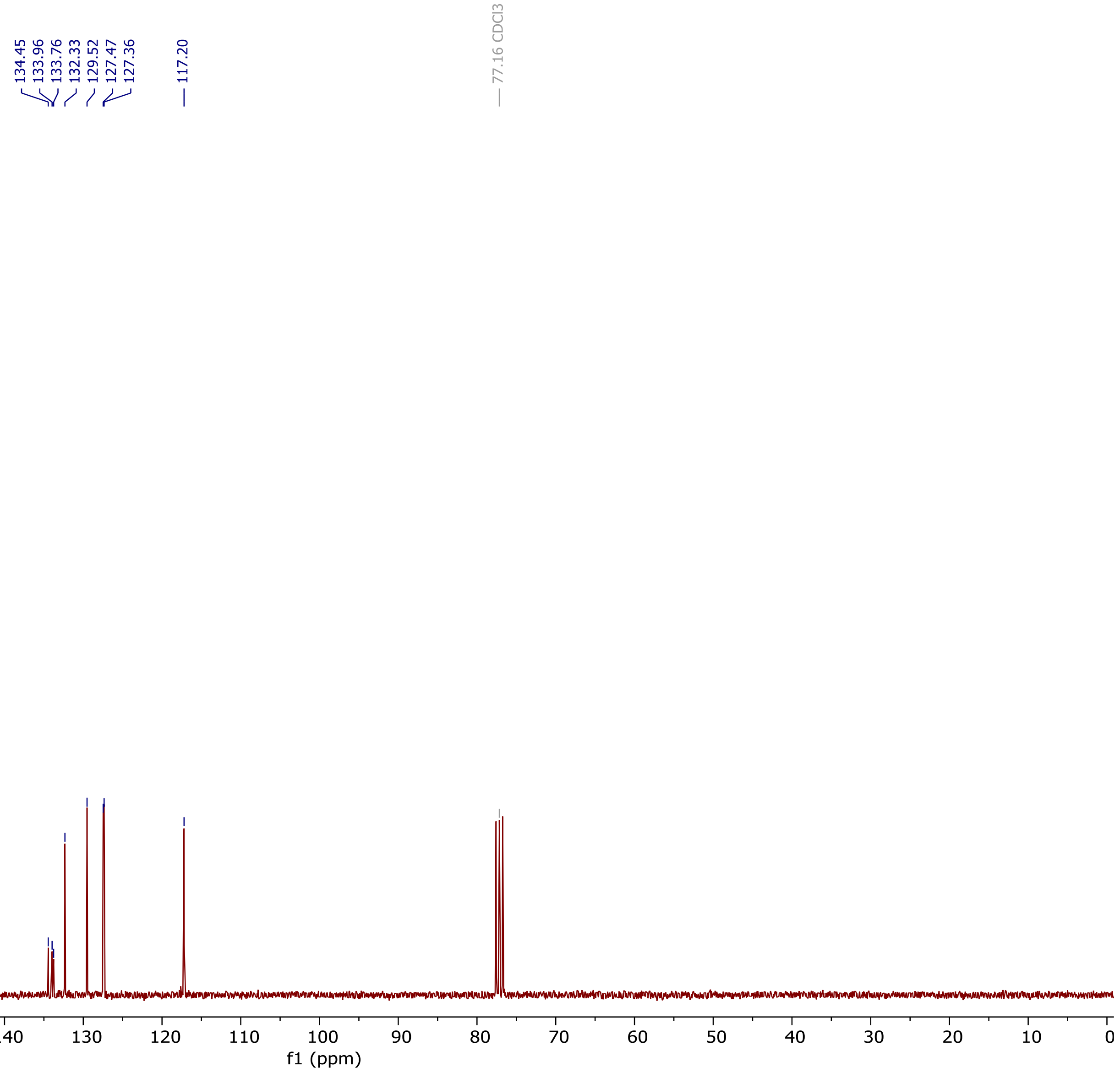

^{1}H NMR (300 MHz, $CDCl_3$) of **S-6**

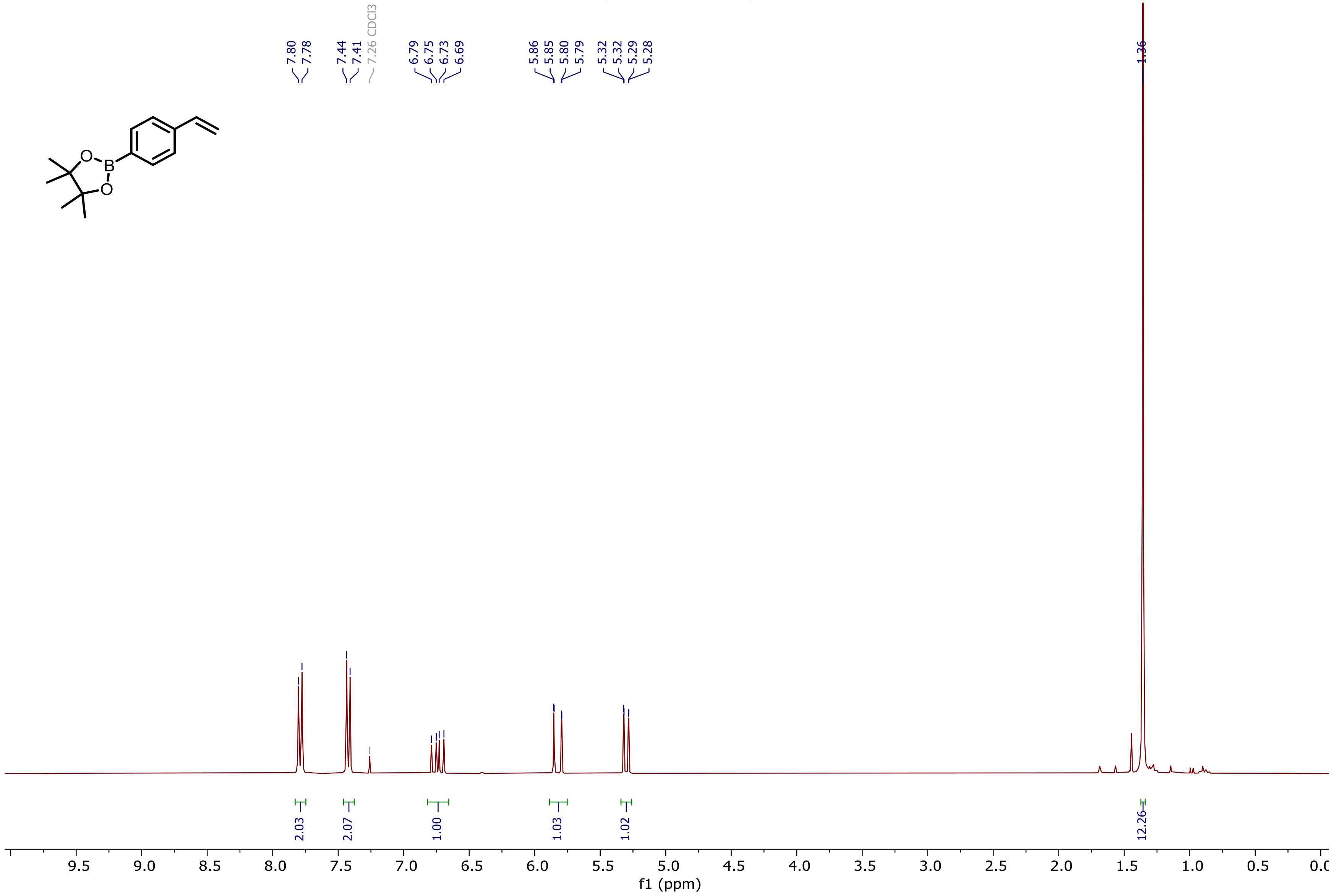

^{13}C NMR (75 MHz, $CDCl_3$) of **S-6**

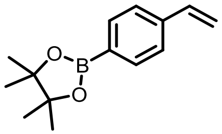

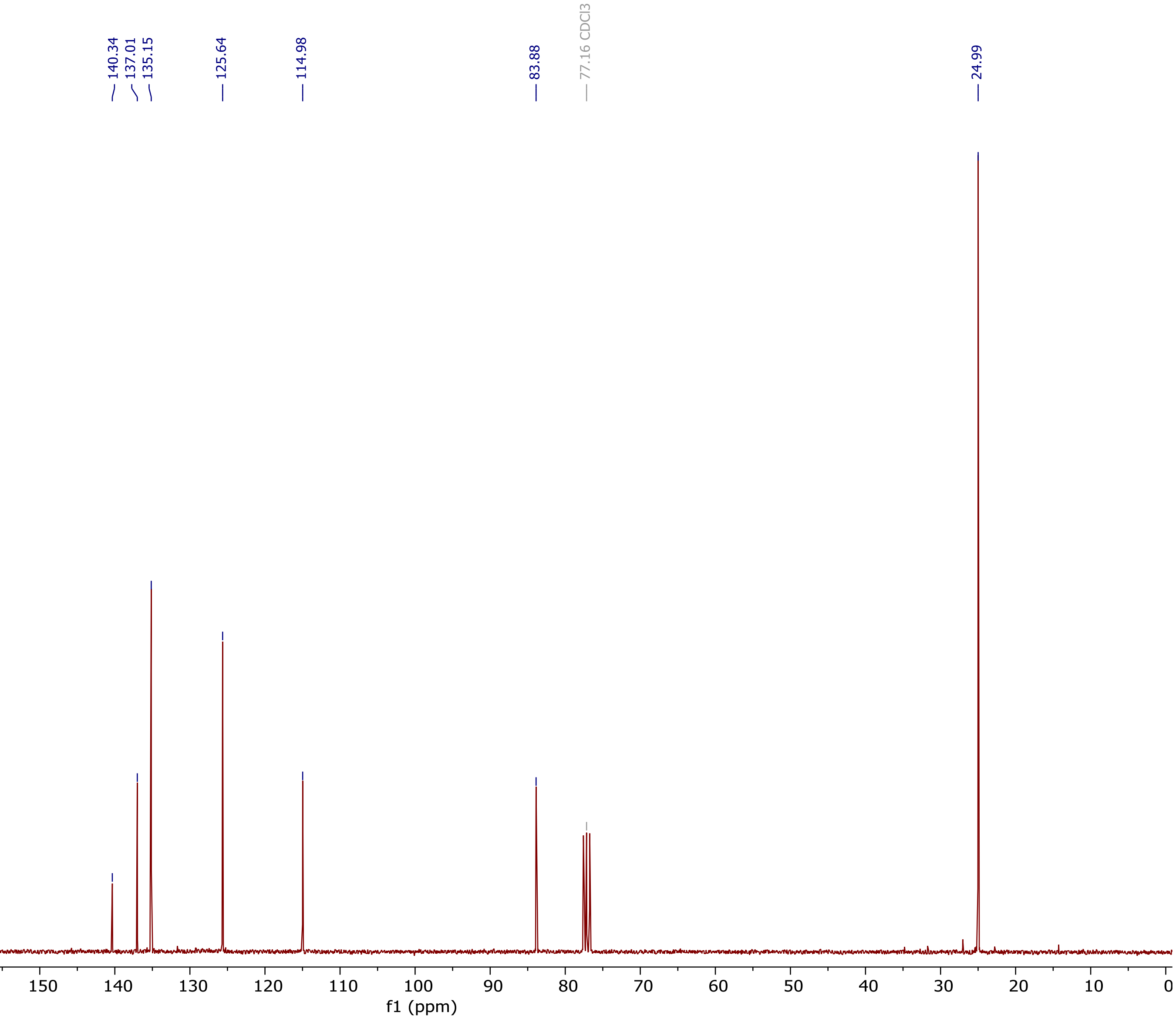

^{1}H NMR (300 MHz, $CDCl_3$) of **S-7**

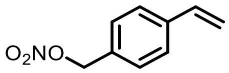

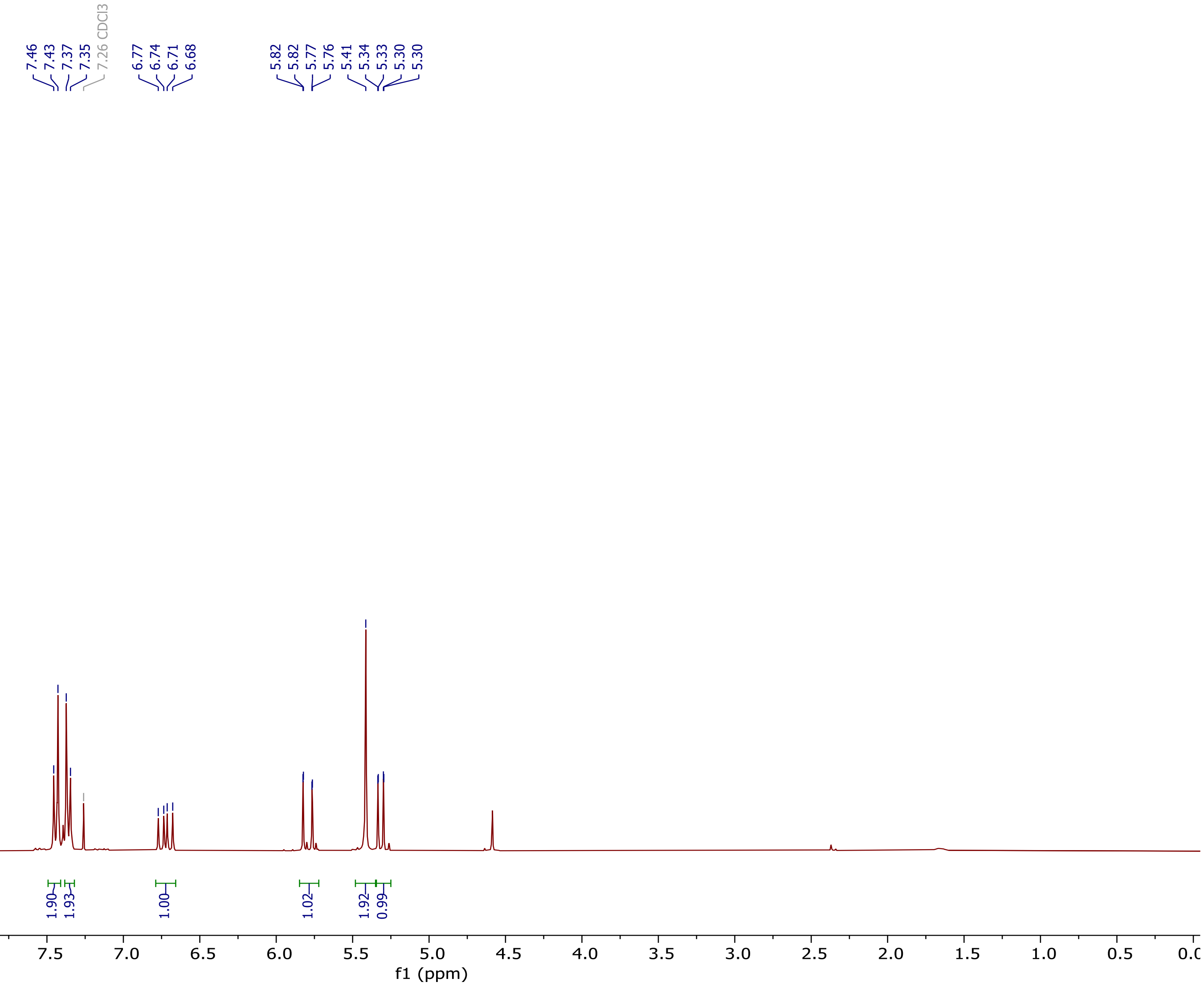

^{13}C NMR (75 MHz, $CDCl_3$) of **S-7**

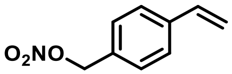

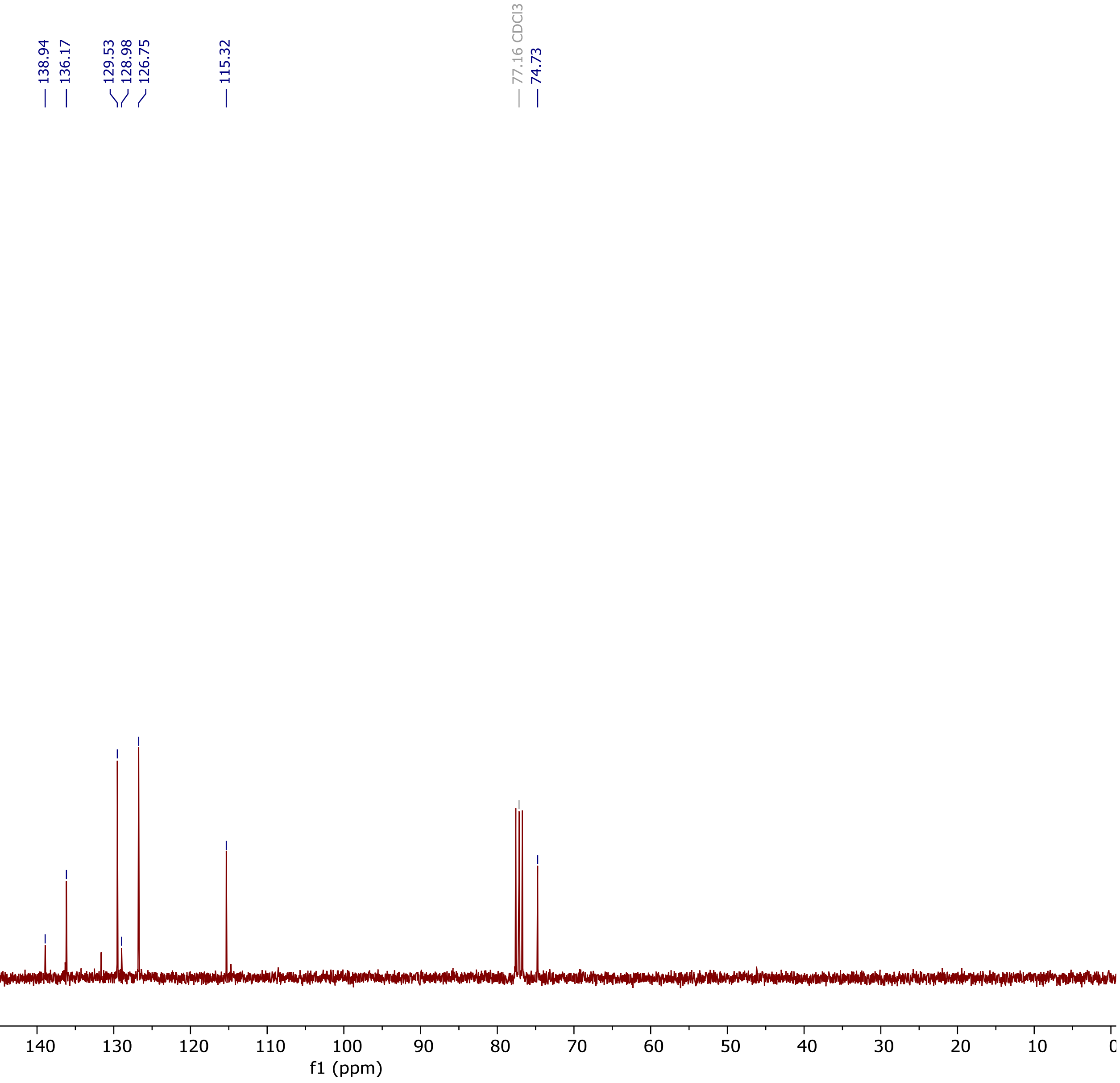

^{1}H NMR (300 MHz, $CDCl_3$) of **1**

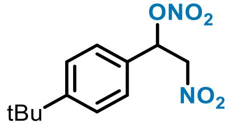

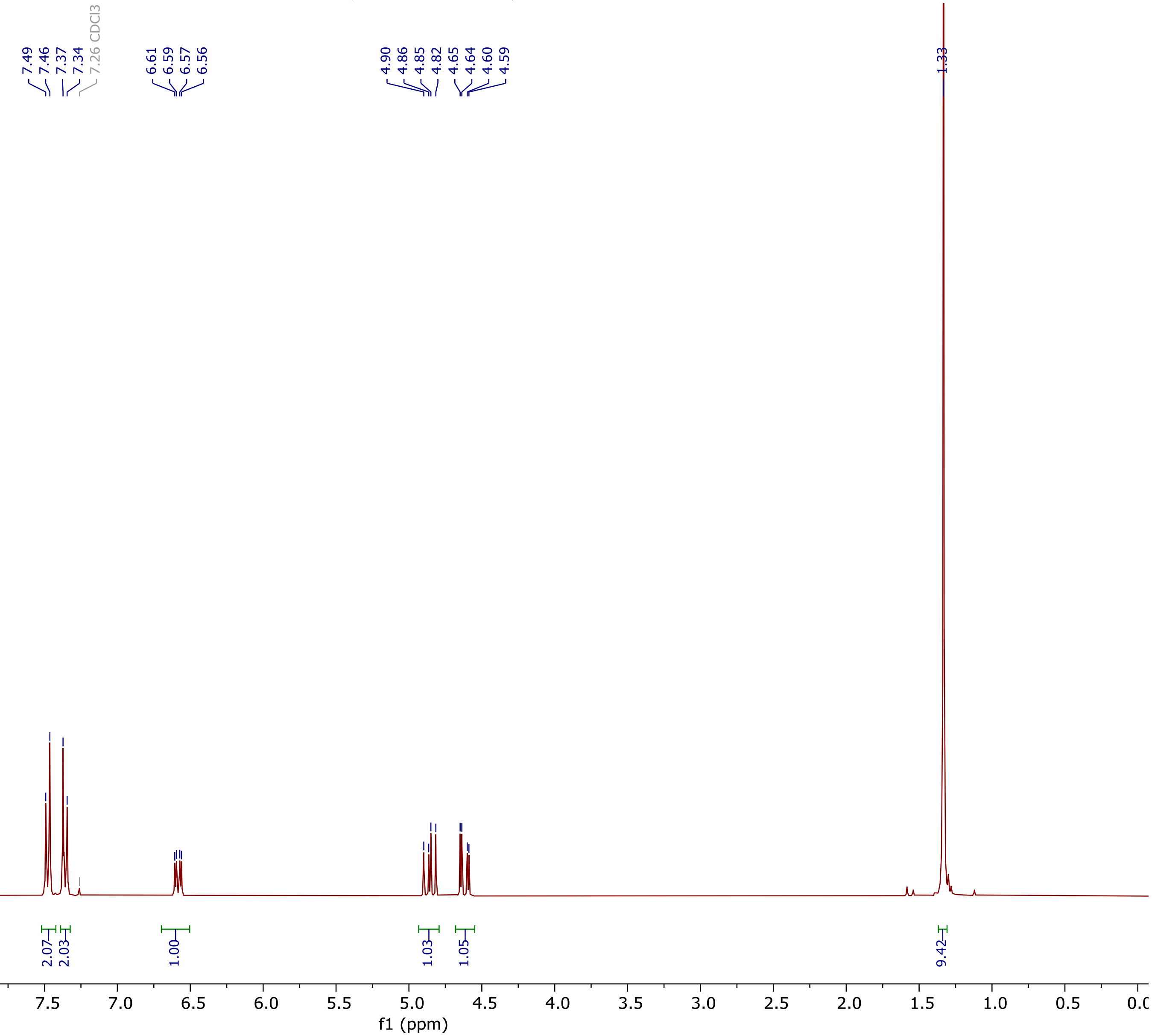

^{13}C NMR (126 MHz, $CDCl_3$) of **1**

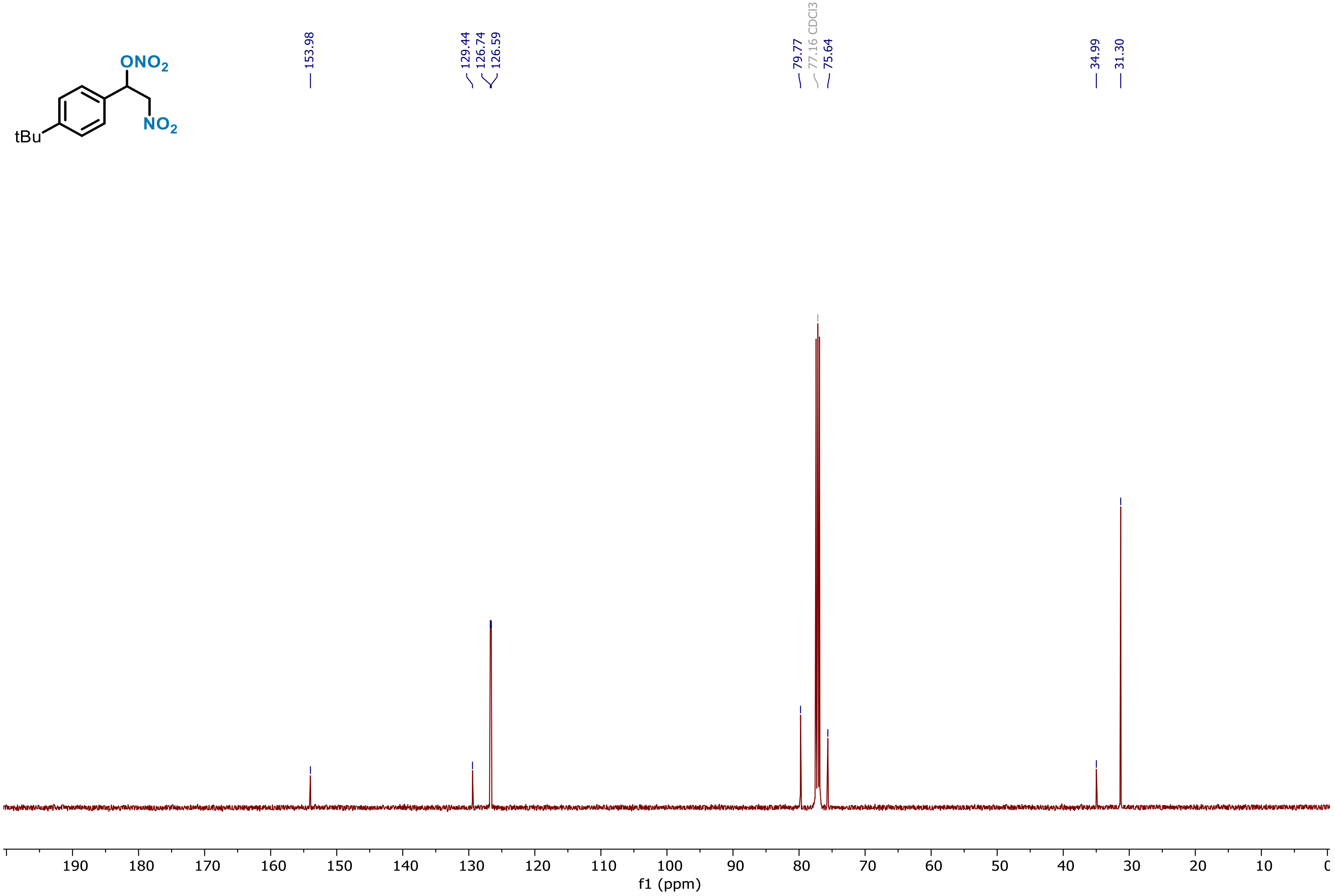

^{1}H NMR (300 MHz, $CDCl_3$) of **2**

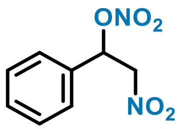

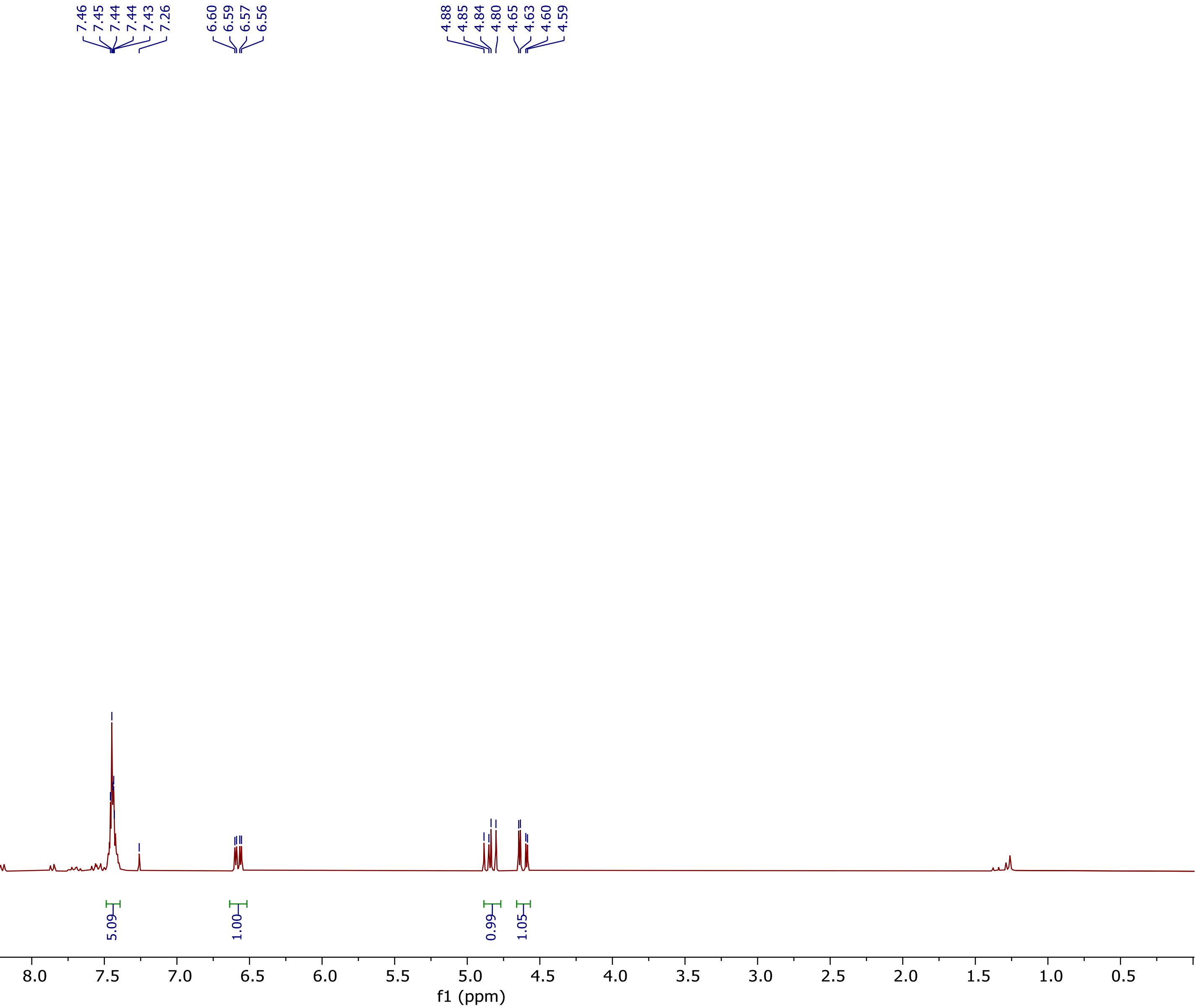

^{13}C NMR (75 MHz, $CDCl_3$) of **2**

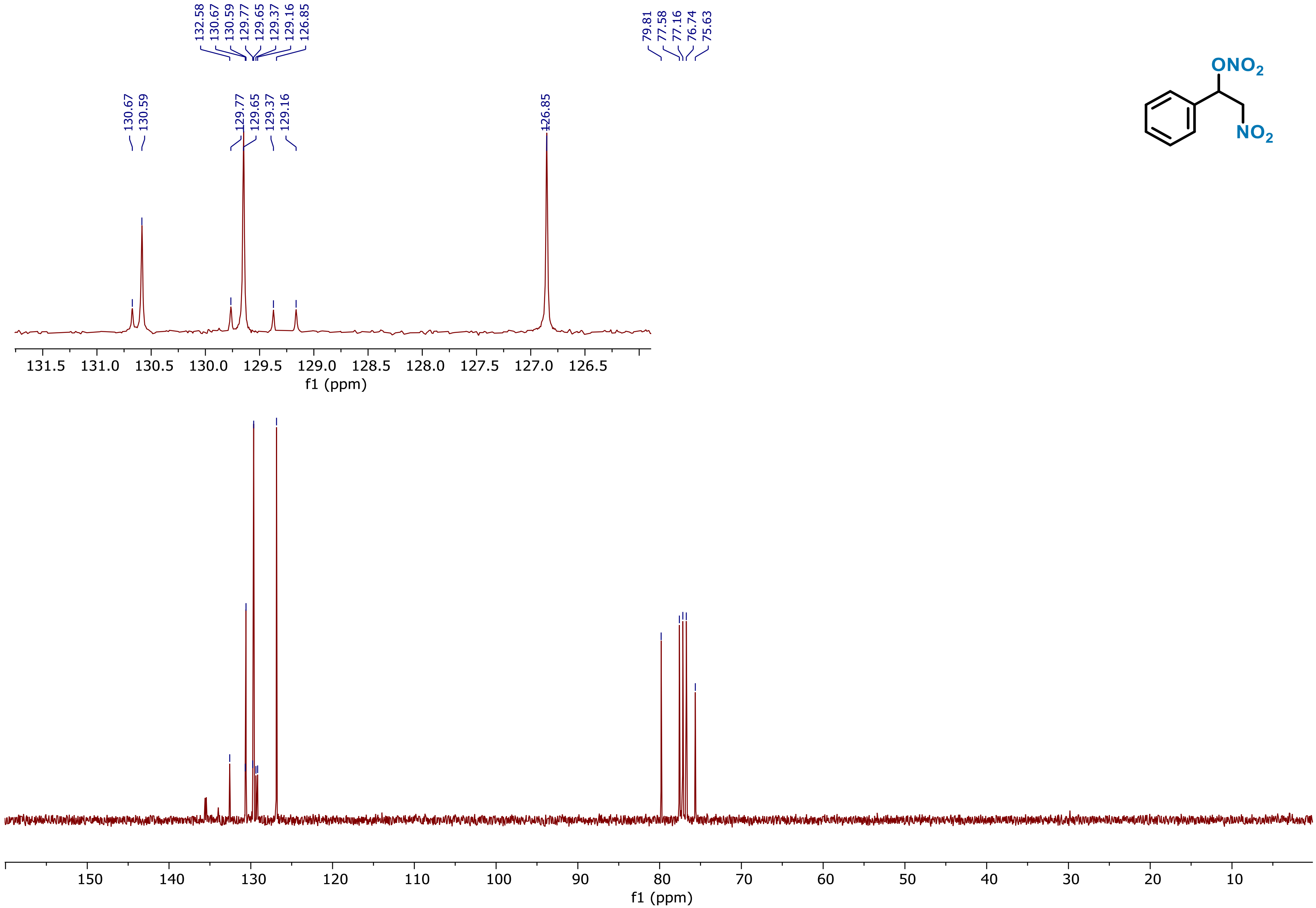

^{1}H NMR (300 MHz, $CDCl_3$) of **3**

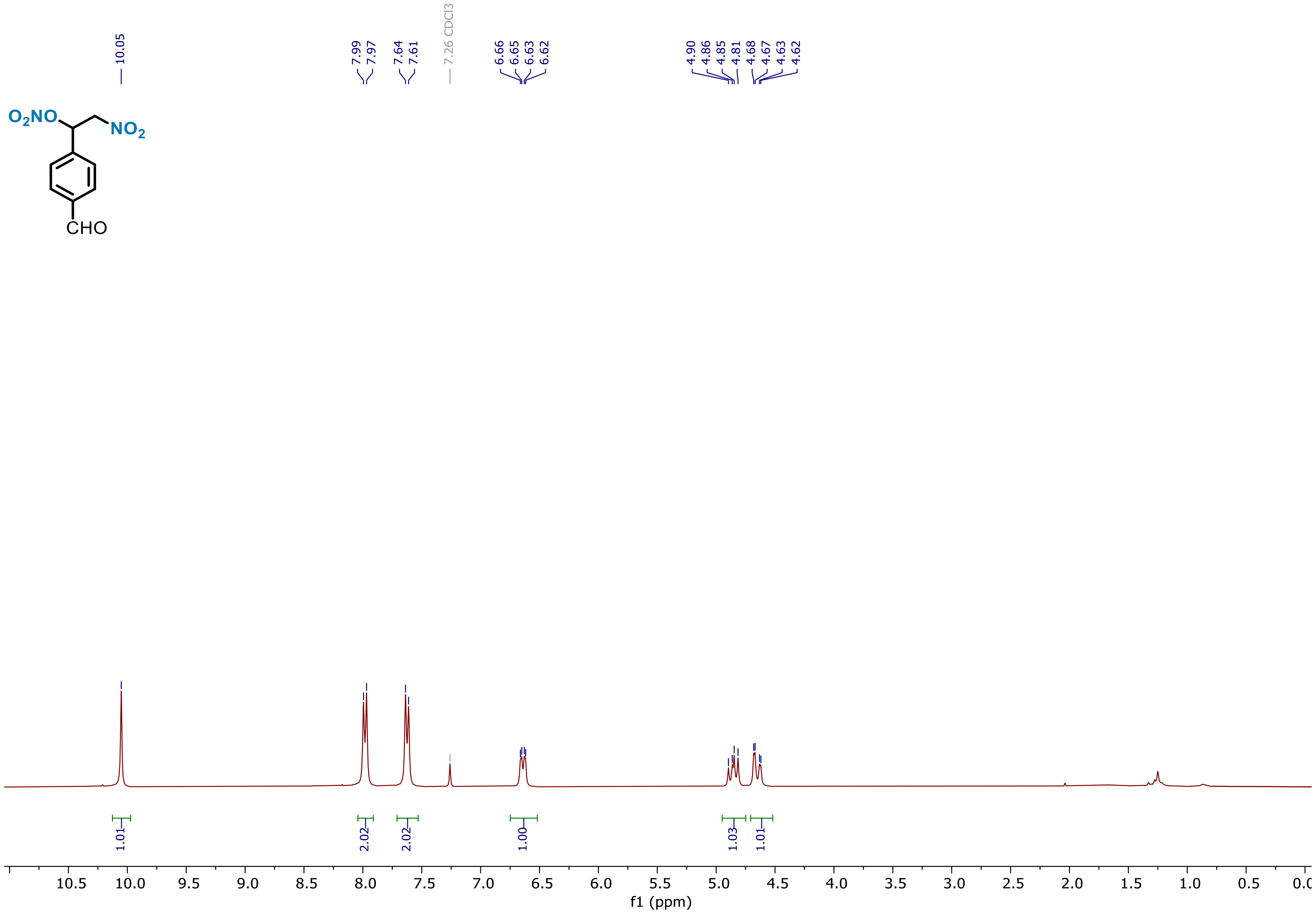

^{13}C NMR (75 MHz, $CDCl_3$) of **3**

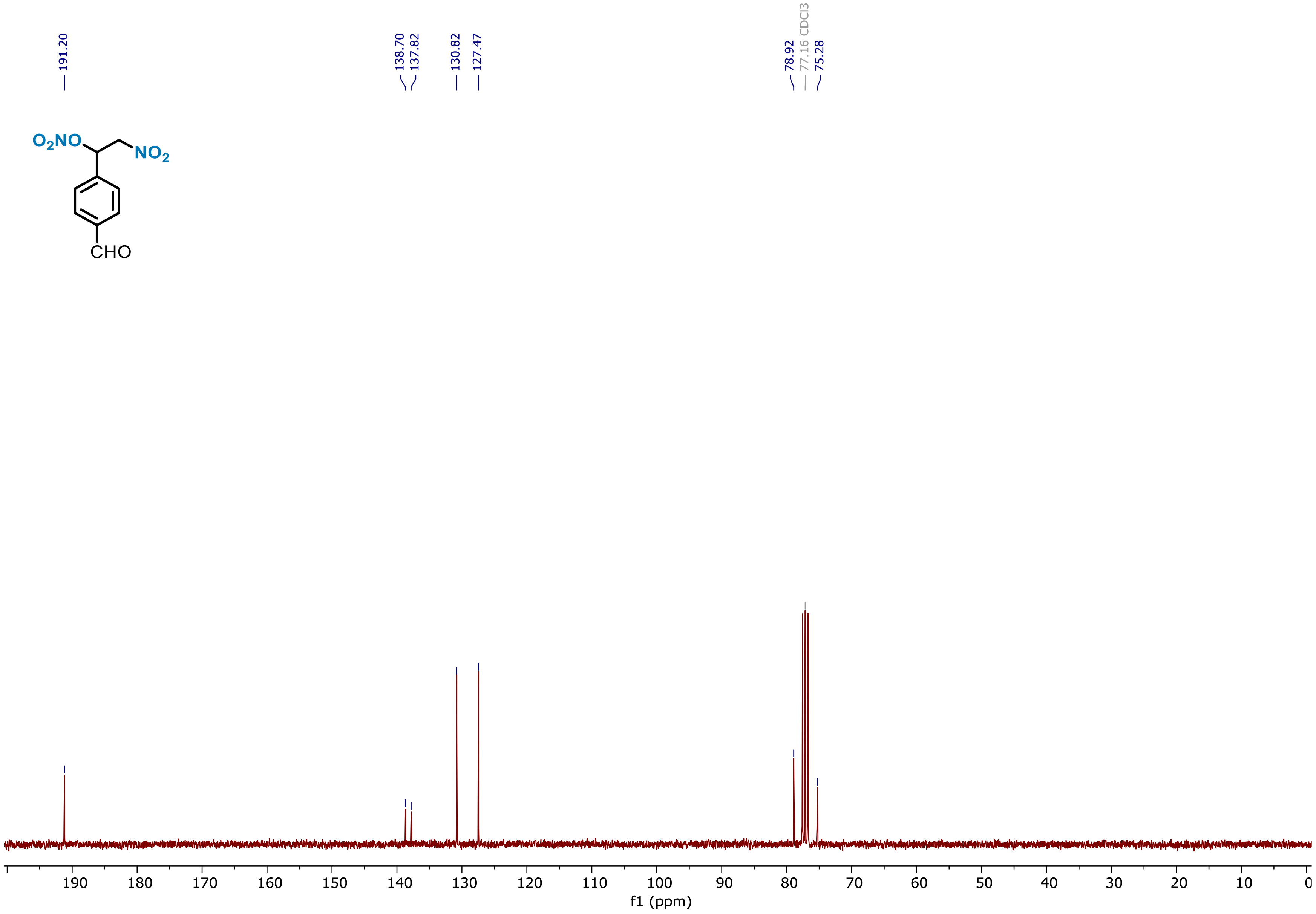

^{1}H NMR (300 MHz, $CDCl_3$) of **9**

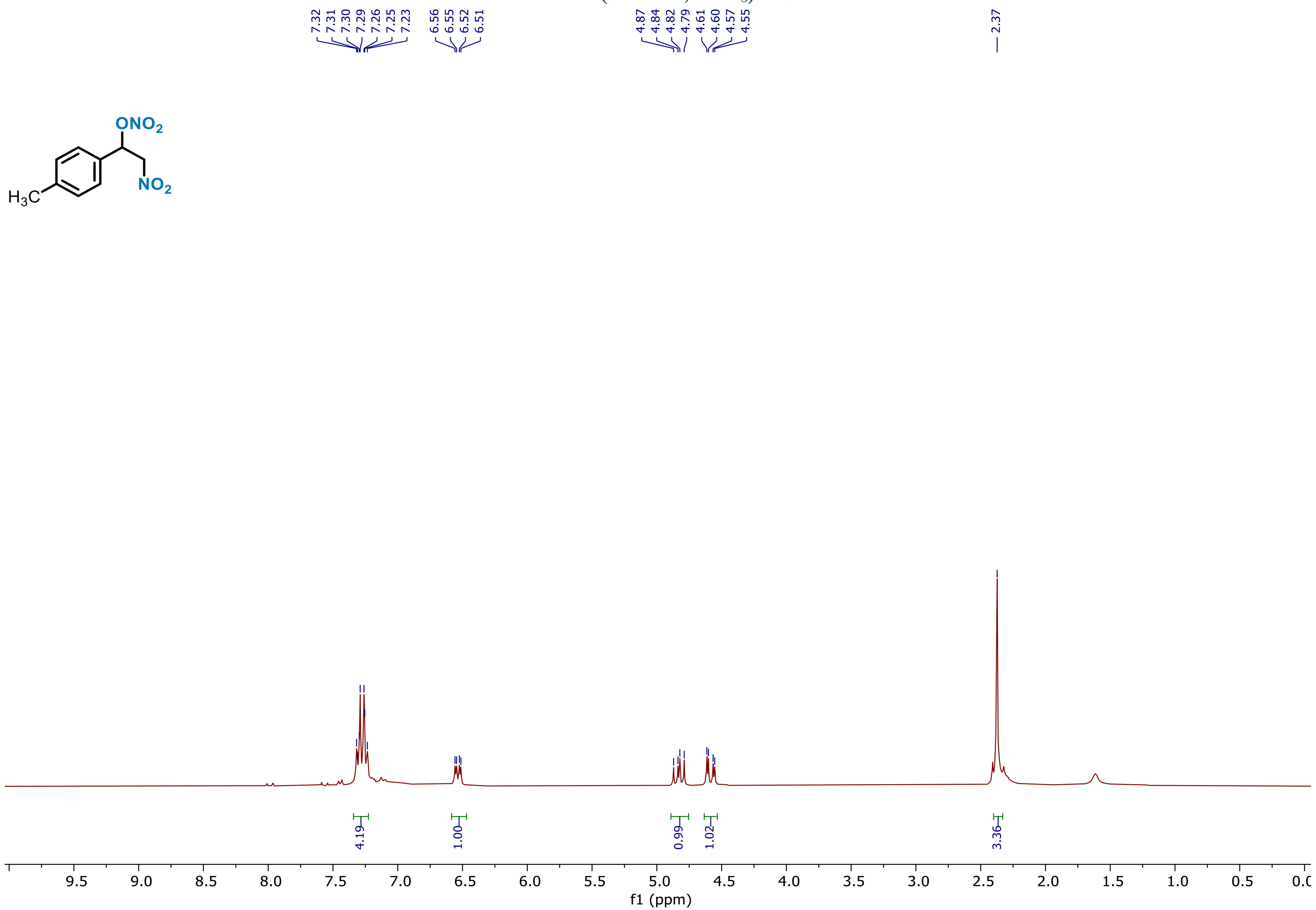

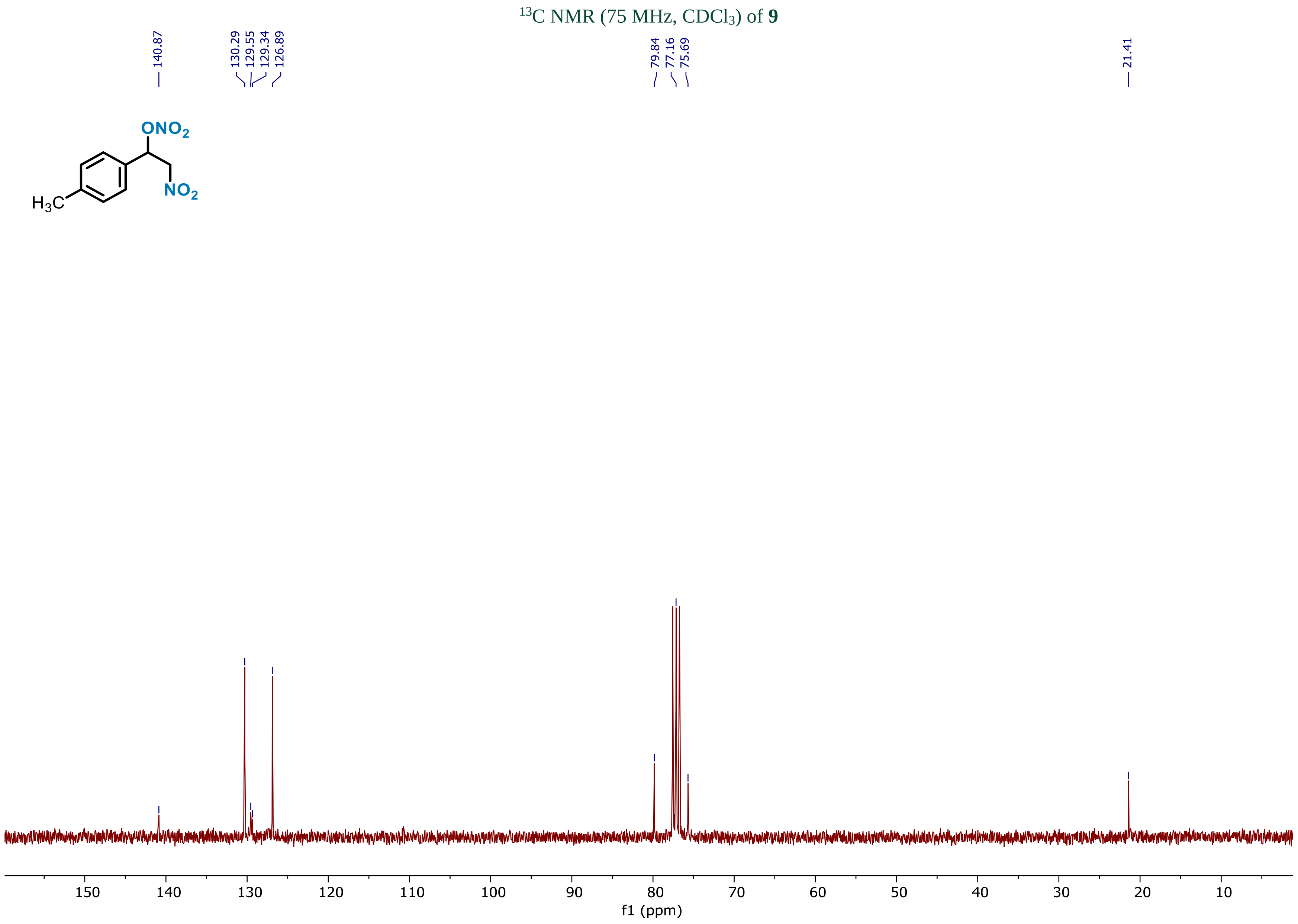

13C NMR (75 MHz, CDCl3) of 9
140.87
130.29
129.55
129.34
126.89
79.84
77.16
75.69
21.41
ONO2
NO2
H3C
150
140
130
120
110
100
90
80
70
60
50
40
30
20
10
f1 (ppm)

^{1}H NMR (300 MHz, $CDCl_3$) of **10**

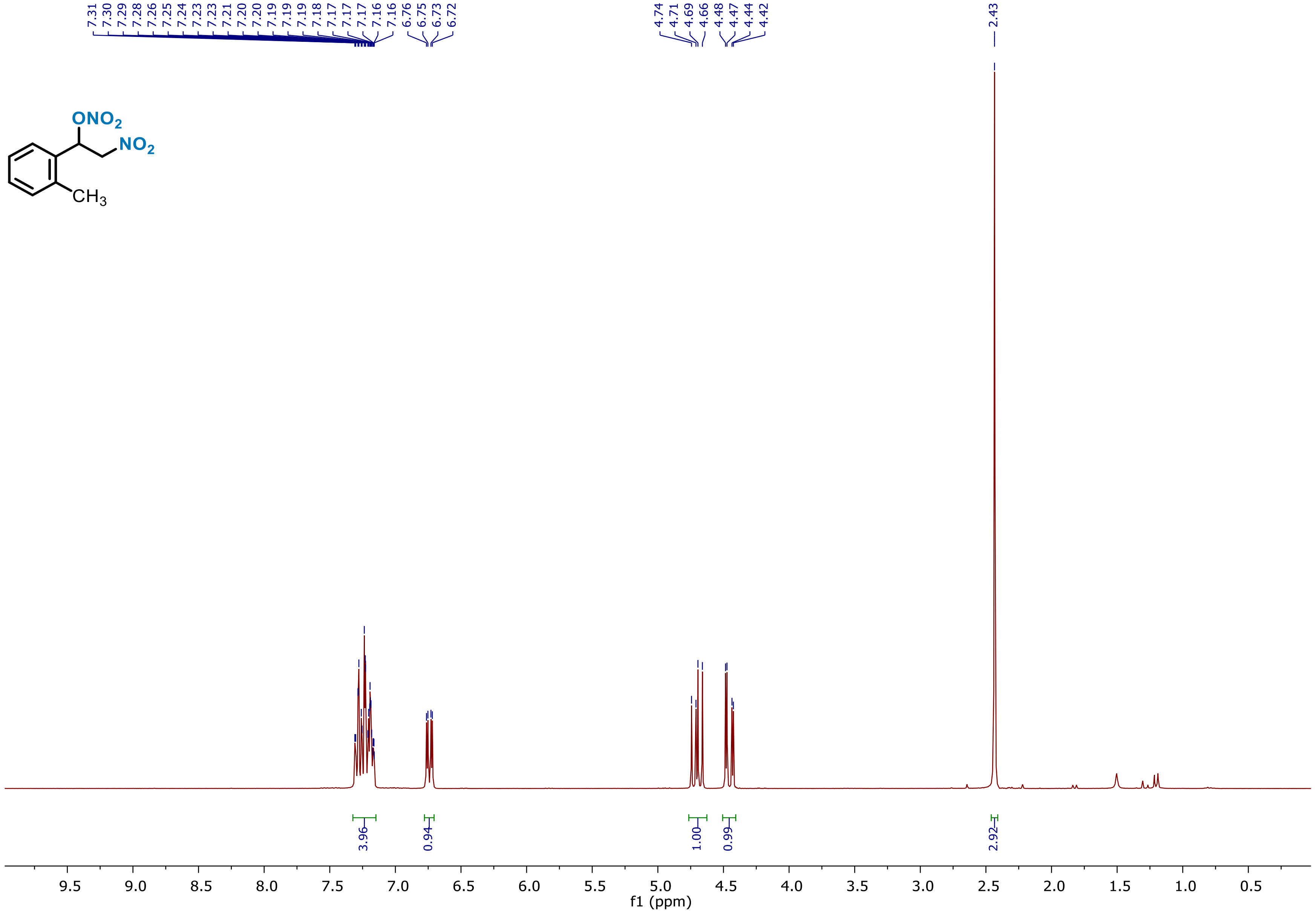

^{13}C NMR (75 MHz, $CDCl_3$) of **10**

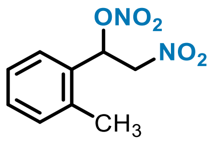

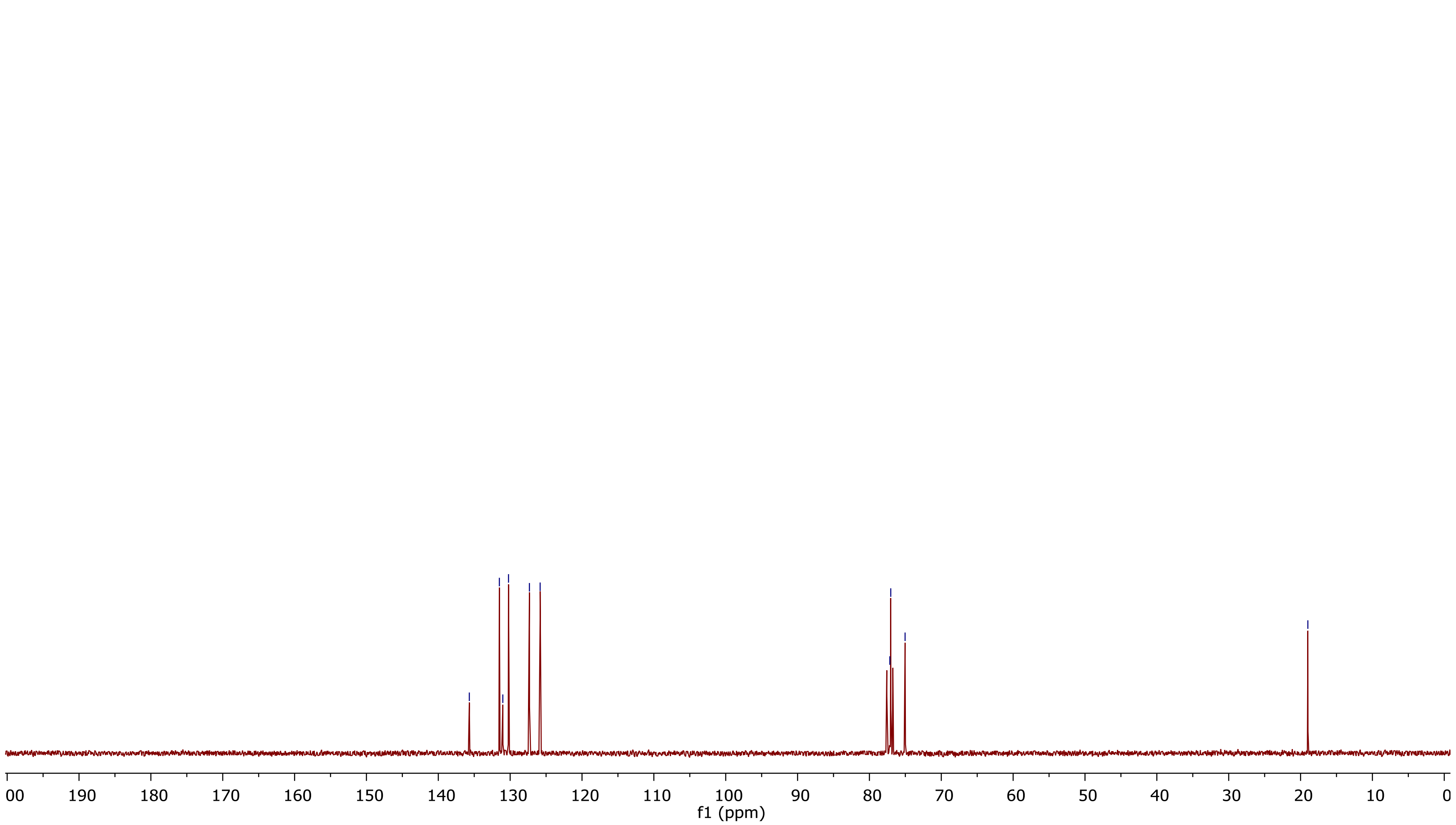

^{1}H NMR (300 MHz, $CDCl_3$) of **11**

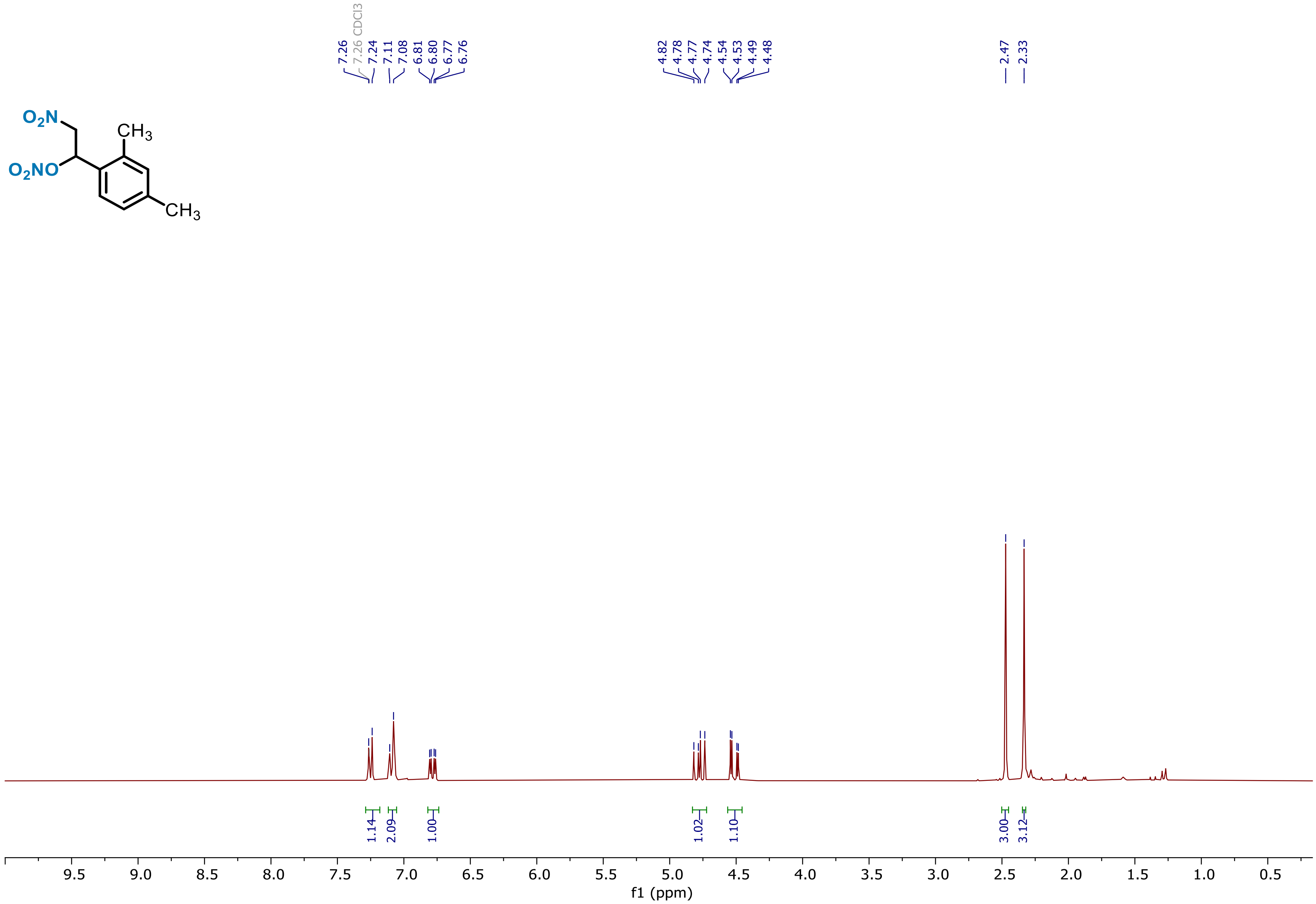

^{13}C NMR (75 MHz, $CDCl_3$) of **11**

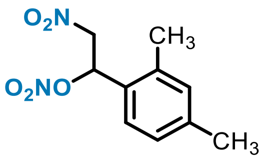

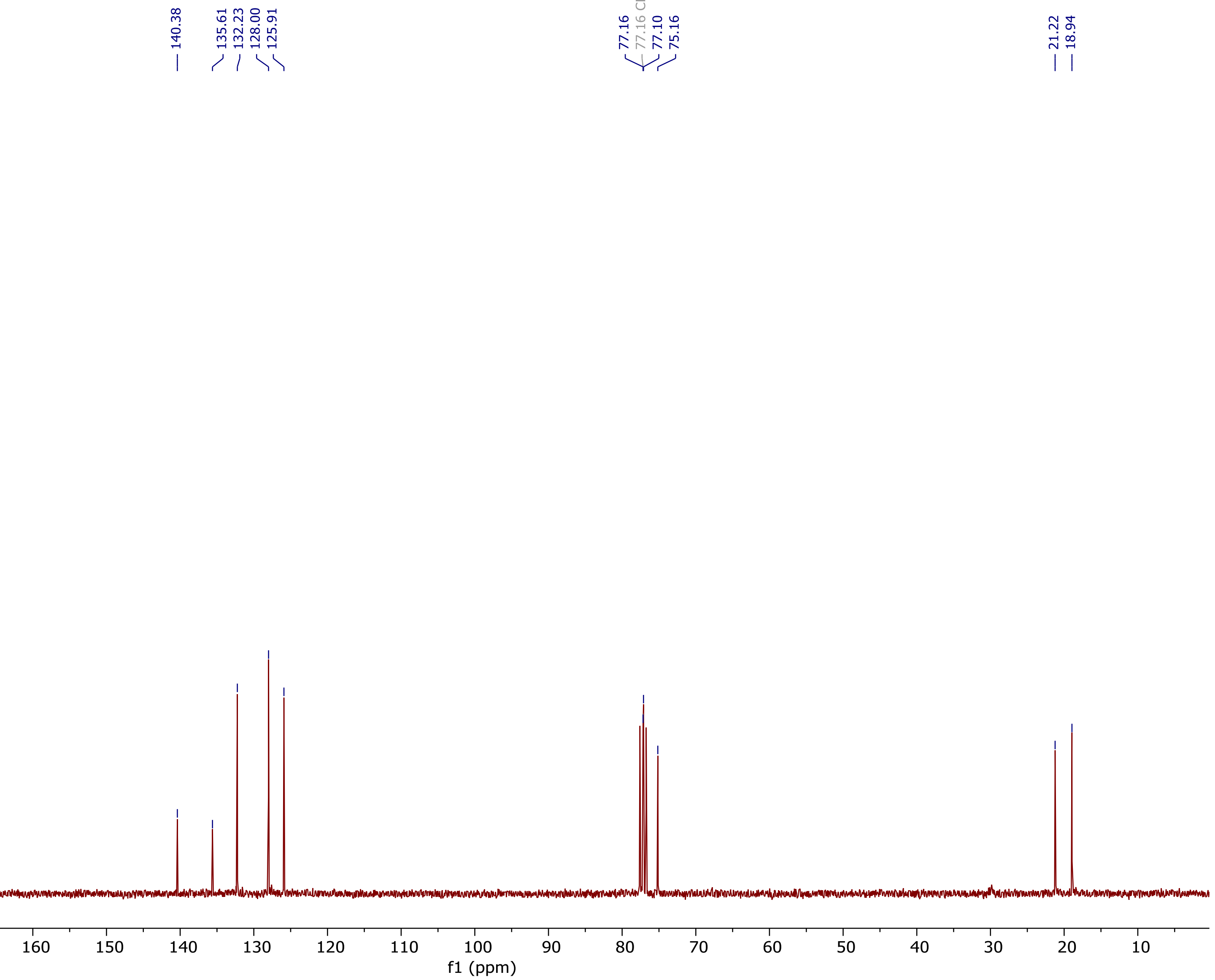

^{1}H NMR (300 MHz, $CDCl_3$) of **12**

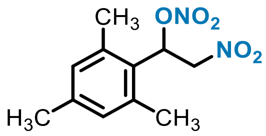

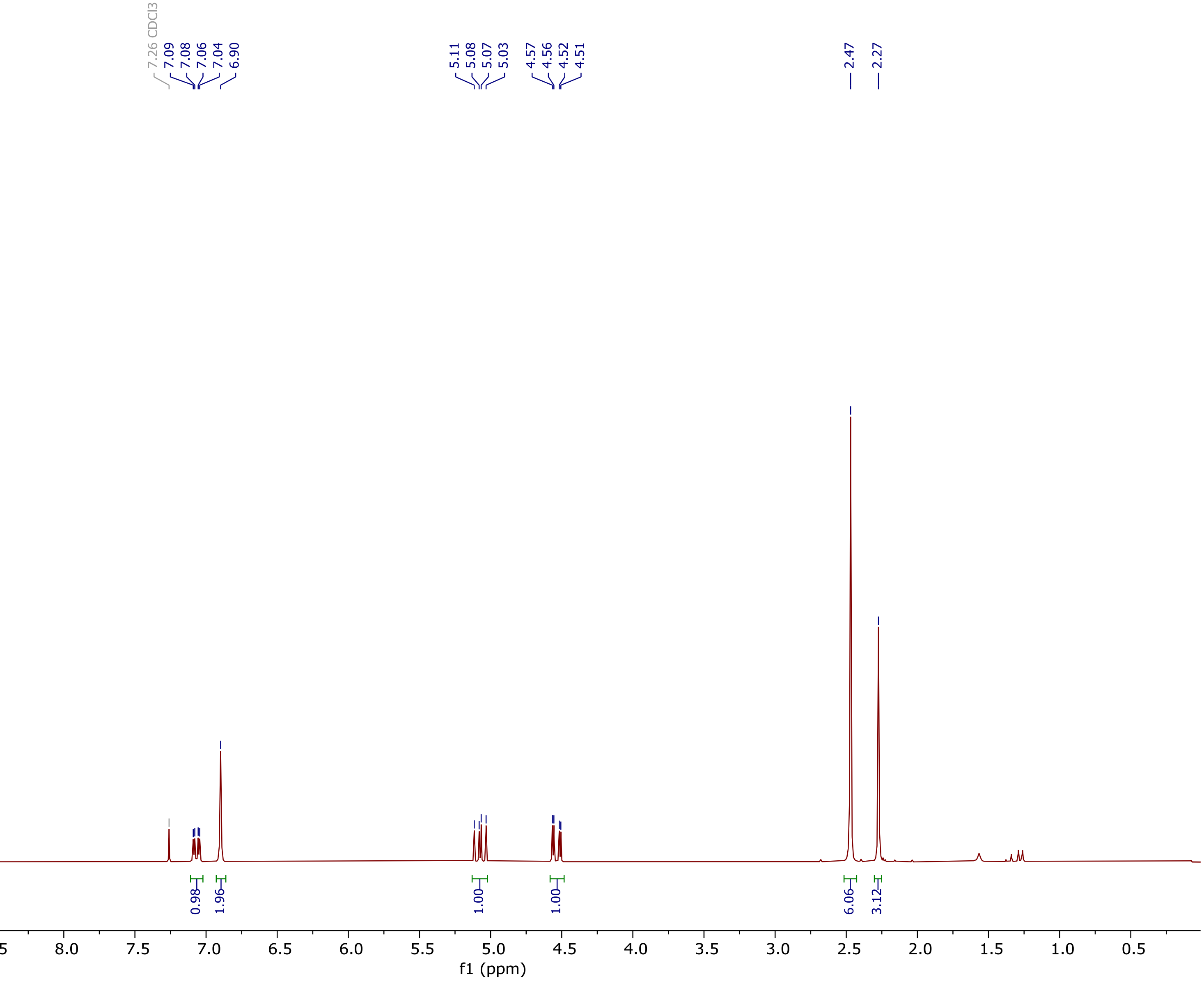

^{13}C NMR (75 MHz, $CDCl_3$) of **12**

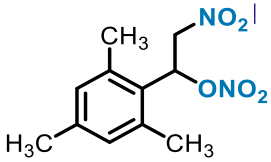

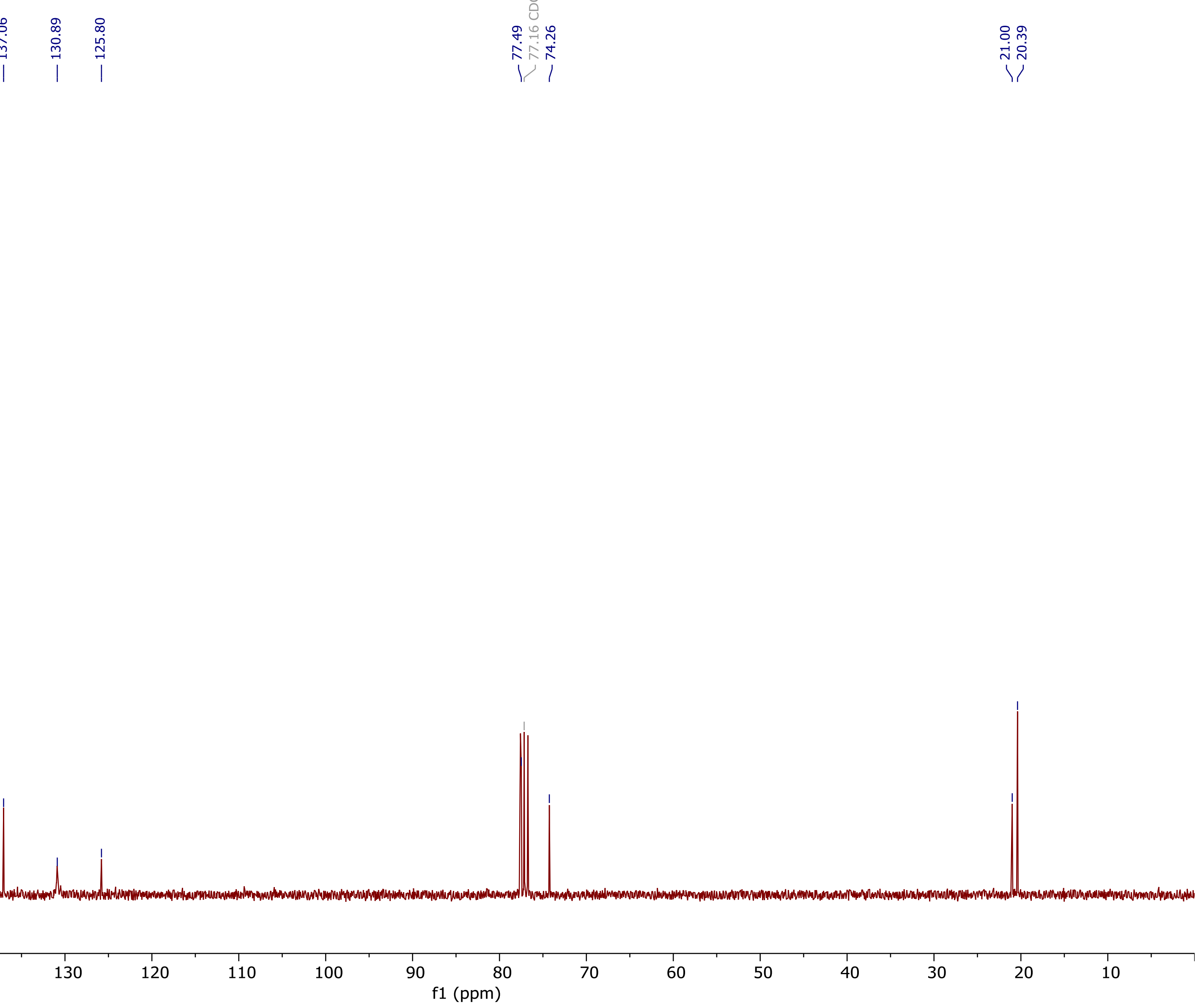

^{1}H NMR (400 MHz, $CDCl_3$) of **13**

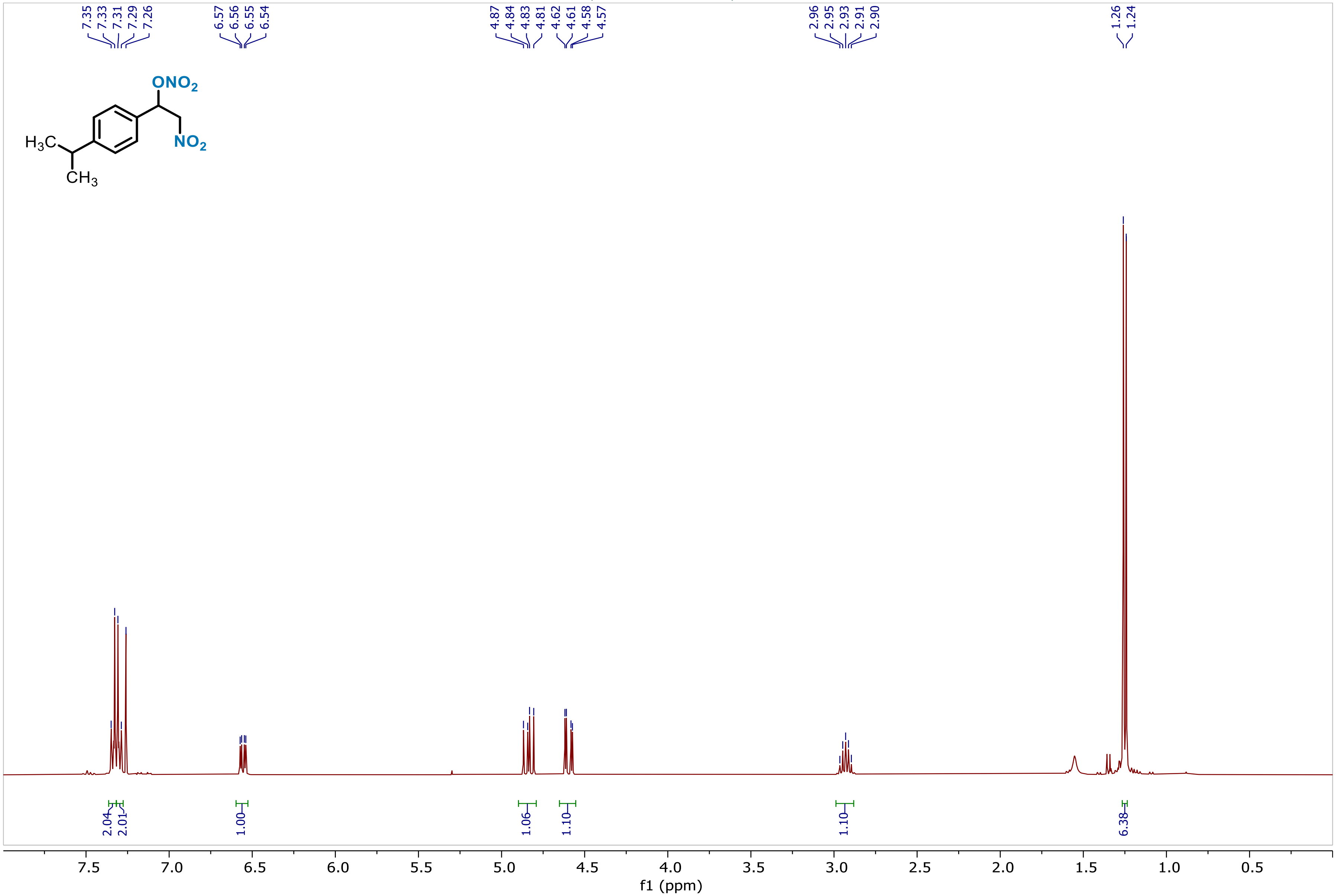

^{13}C NMR (101 MHz, $CDCl_3$) of **13**

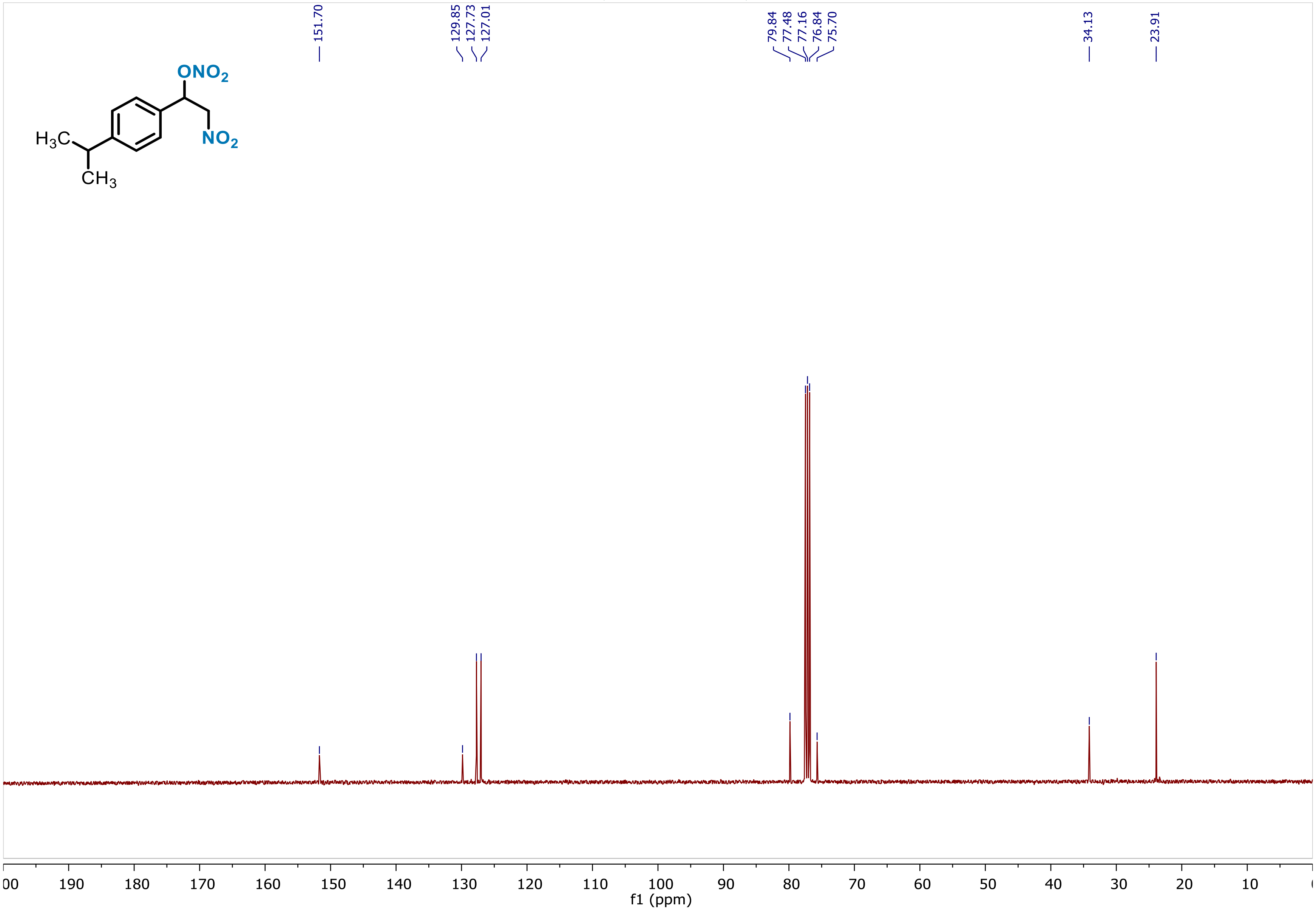

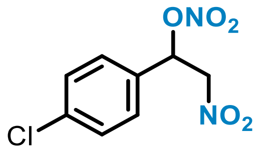

^{1}H NMR (300 MHz, $CDCl_3$) of **14**

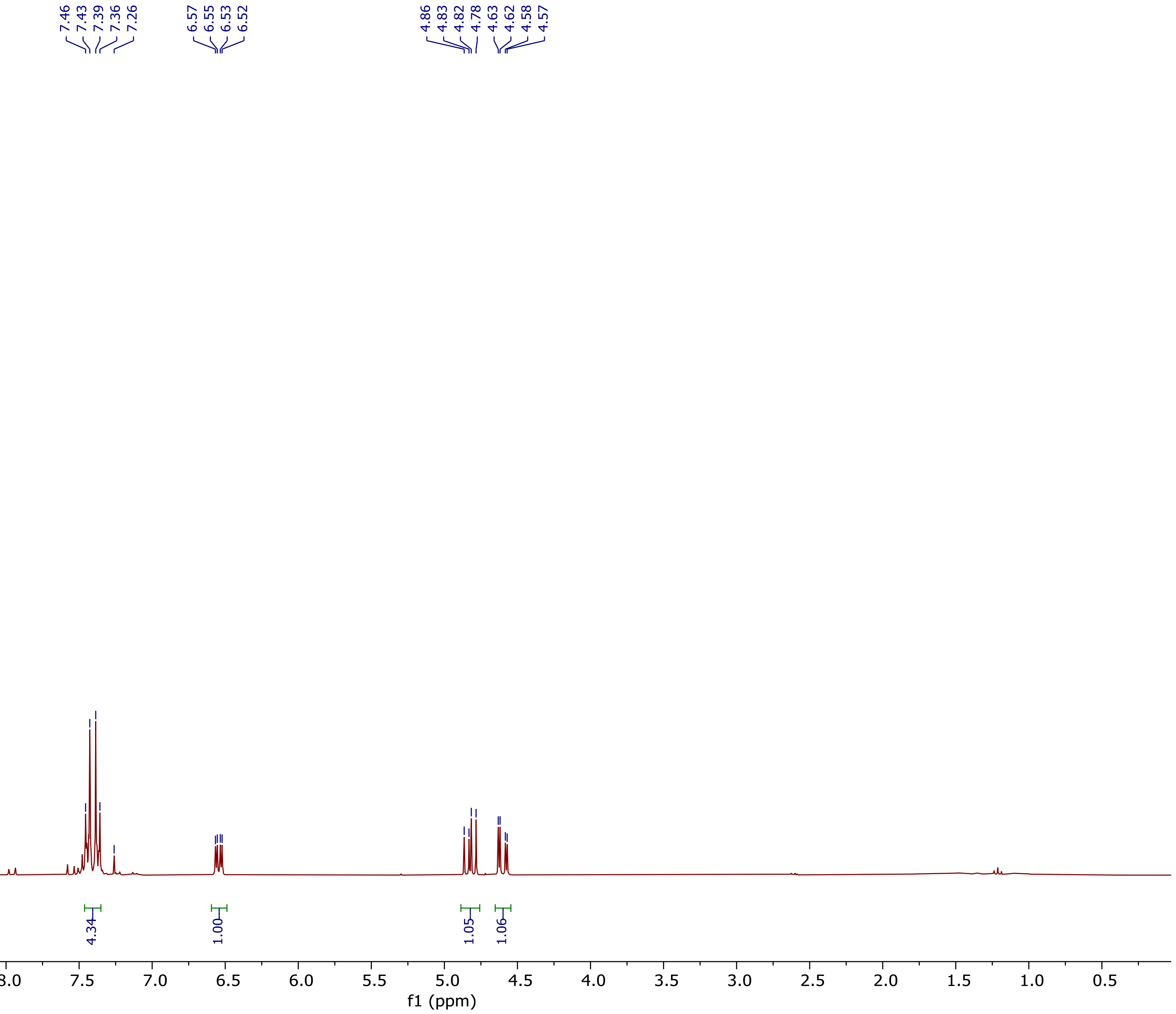

^{13}C NMR (75 MHz, $CDCl_3$) of **14**

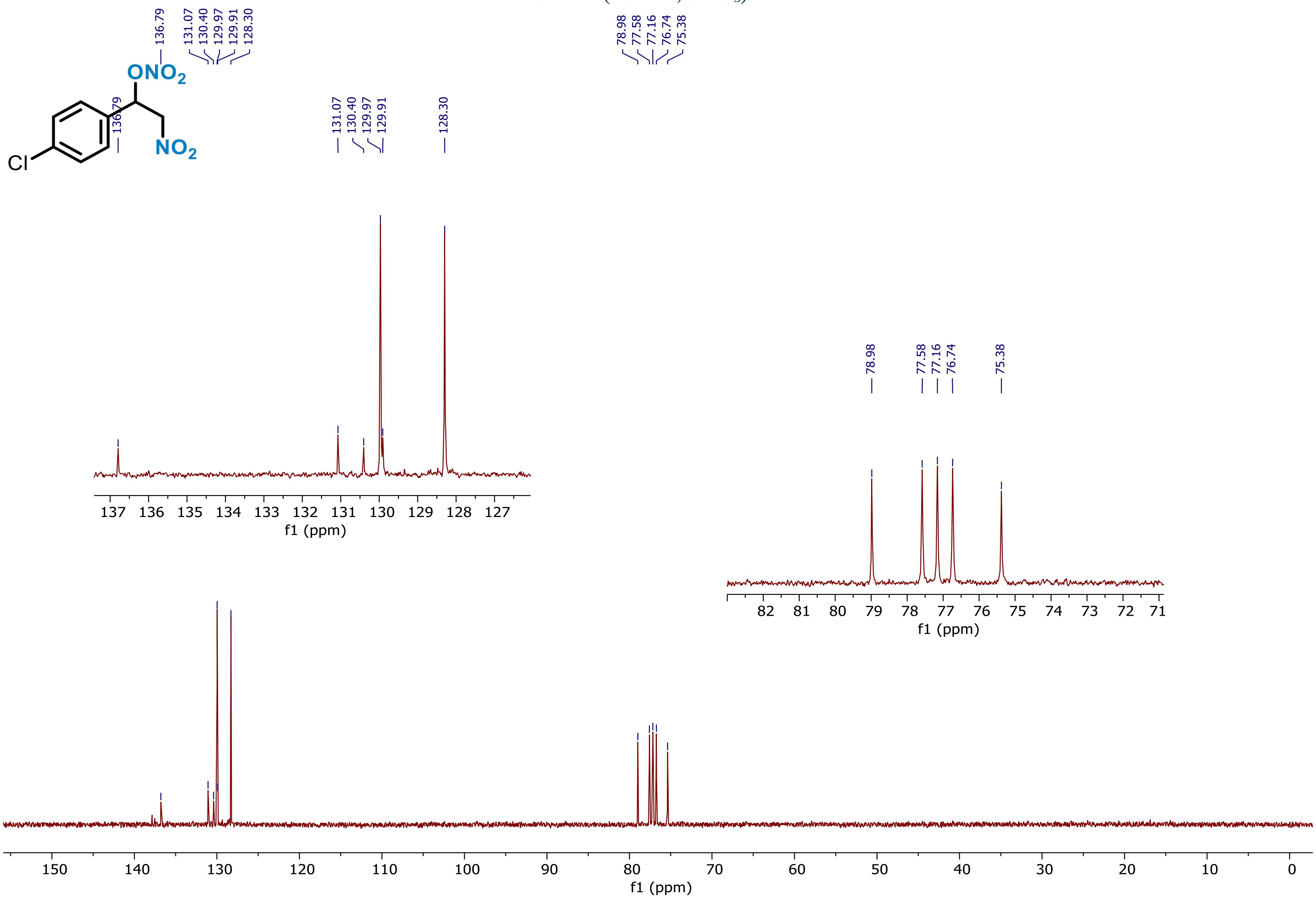

^{1}H NMR (300 MHz, $CDCl_3$) of **15**

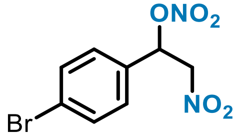

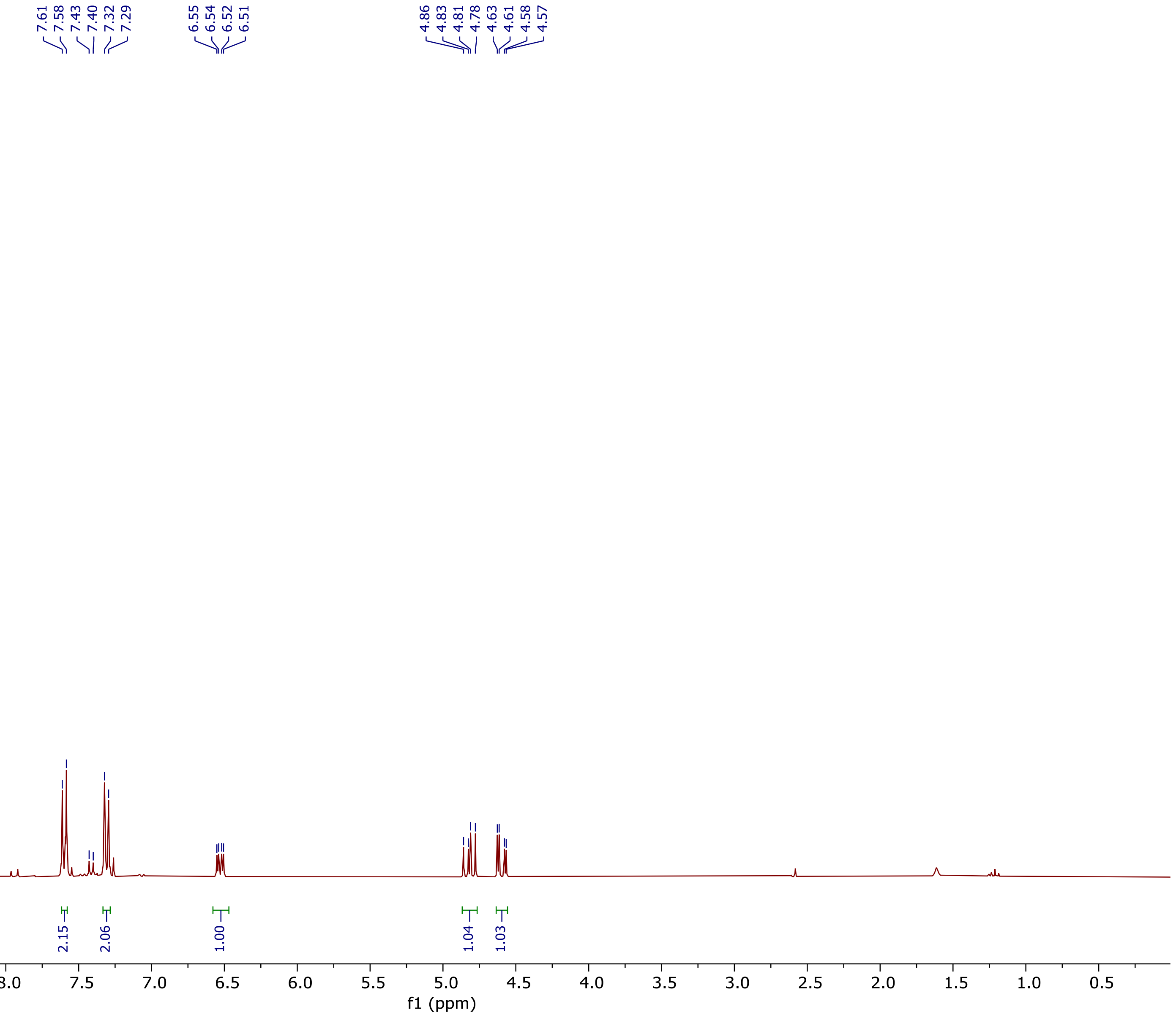

^{13}C NMR (75 MHz, $CDCl_3$) of **15**

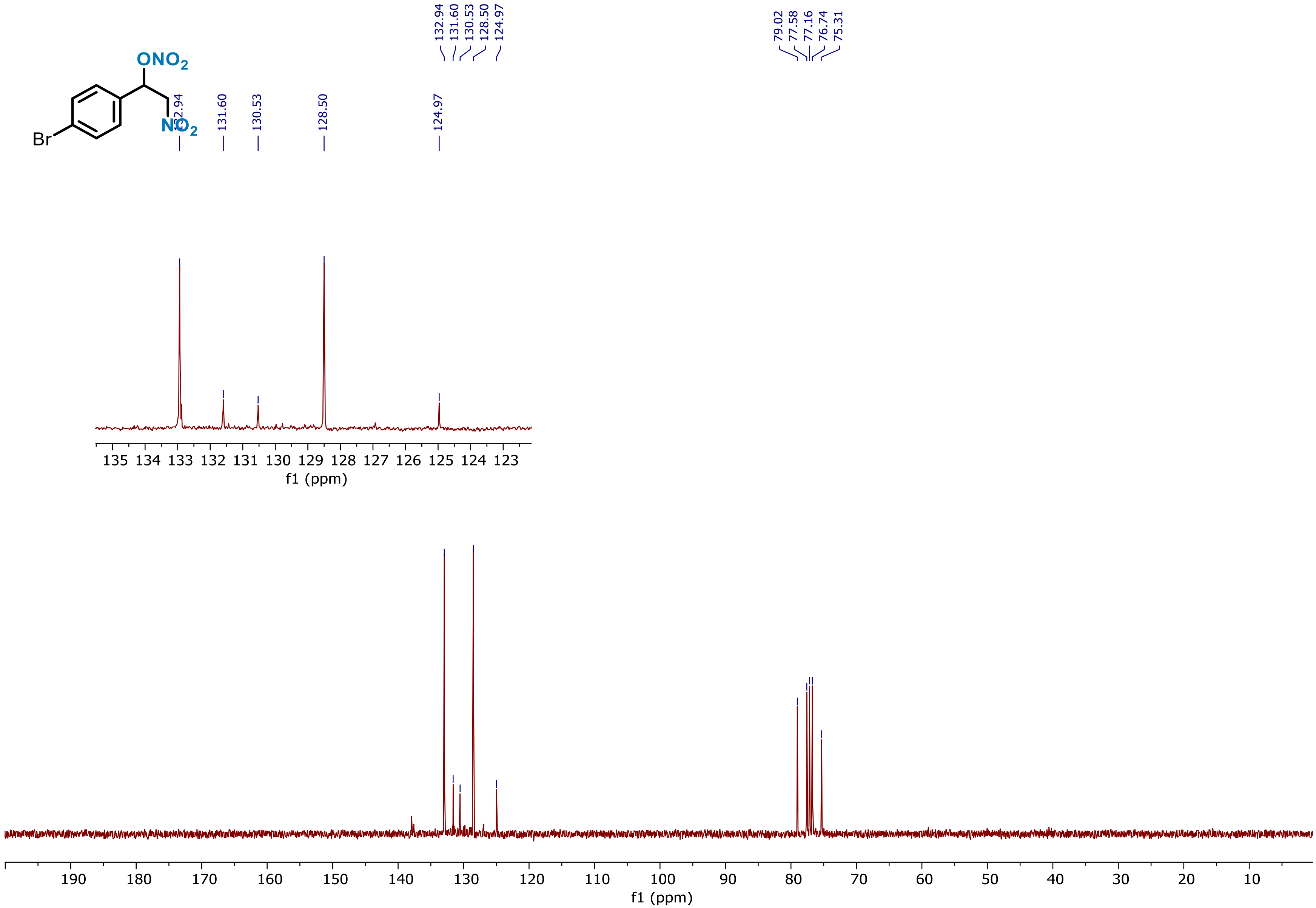

^{1}H NMR (300 MHz, $CDCl_3$) of **16**

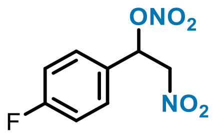

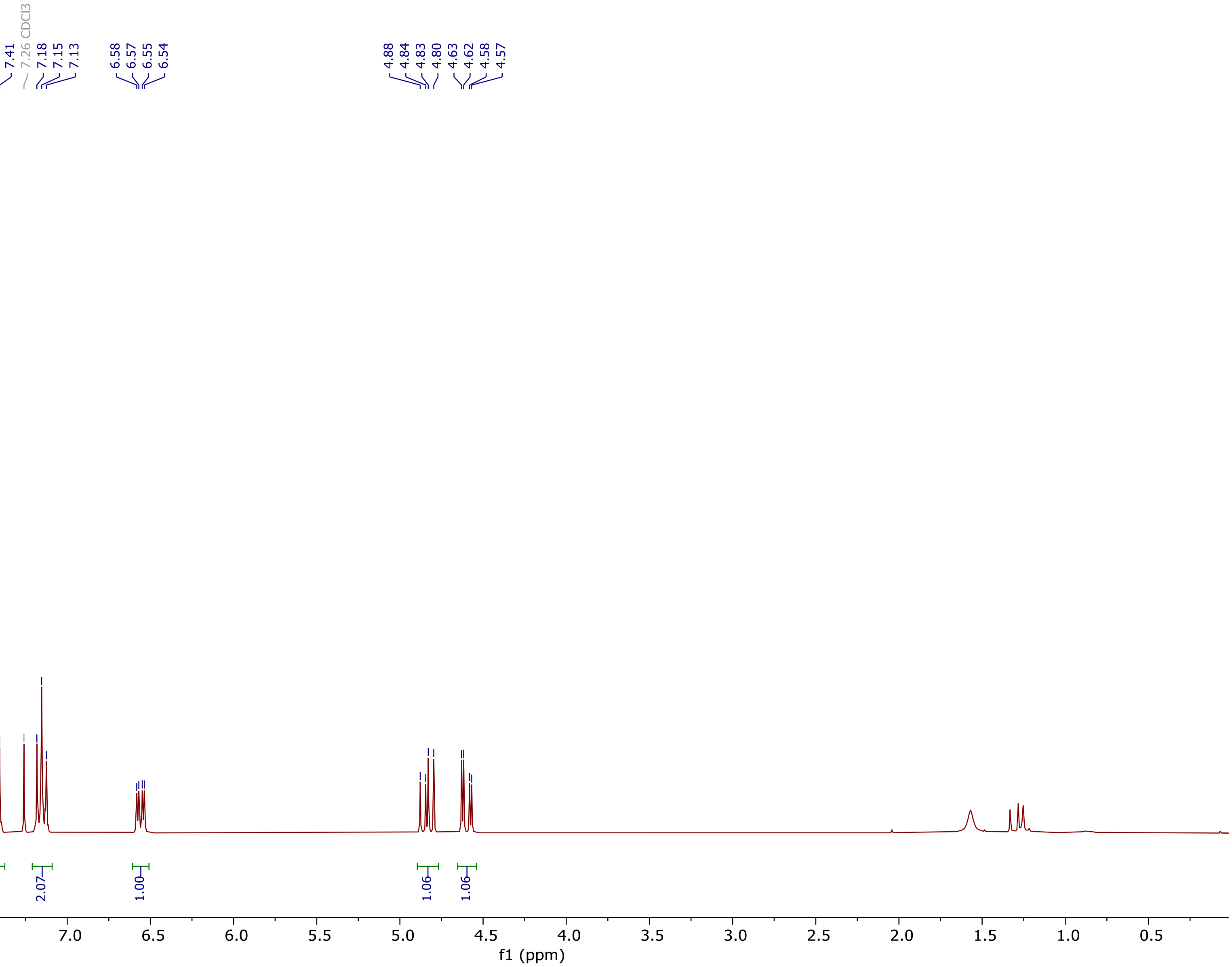

^{13}C NMR (75 MHz, $CDCl_3$) of **16**

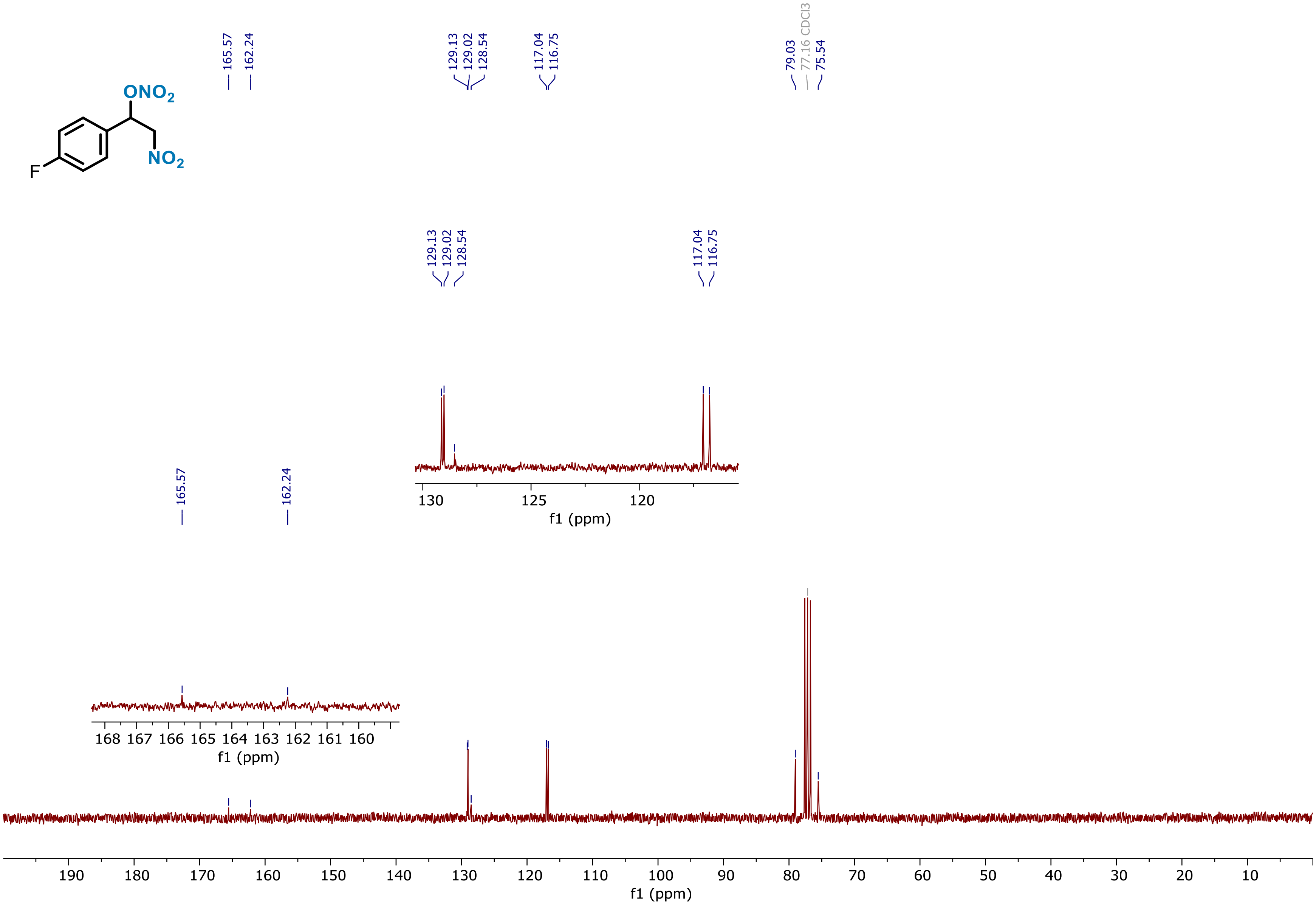

^{19}F NMR (282 MHz, $CDCl_3$) of **16**

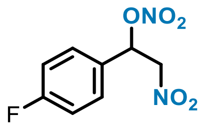

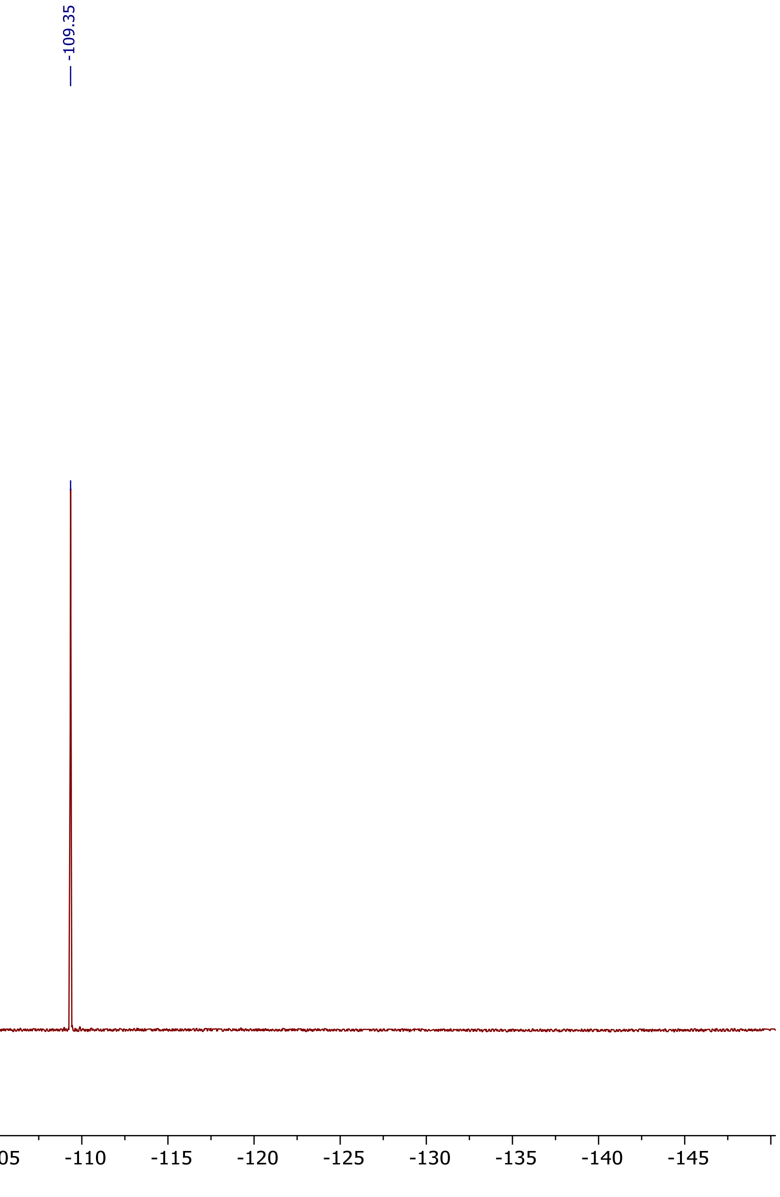

^{1}H NMR (300 MHz, $CDCl_3$) of **17**

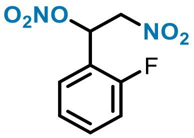

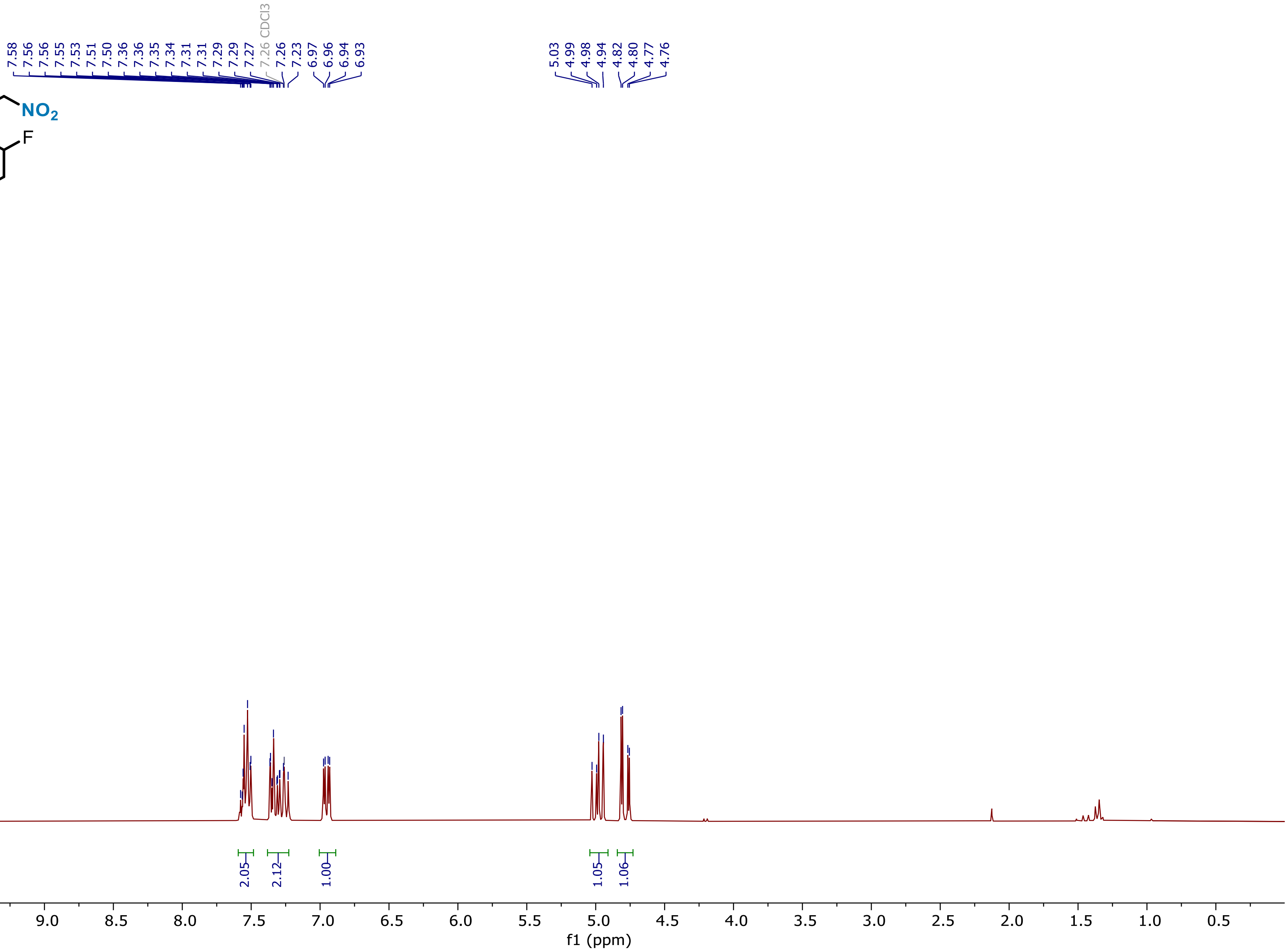

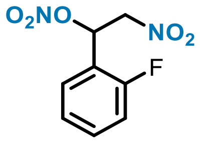

^{13}C NMR (75 MHz, $CDCl_3$) of **17**

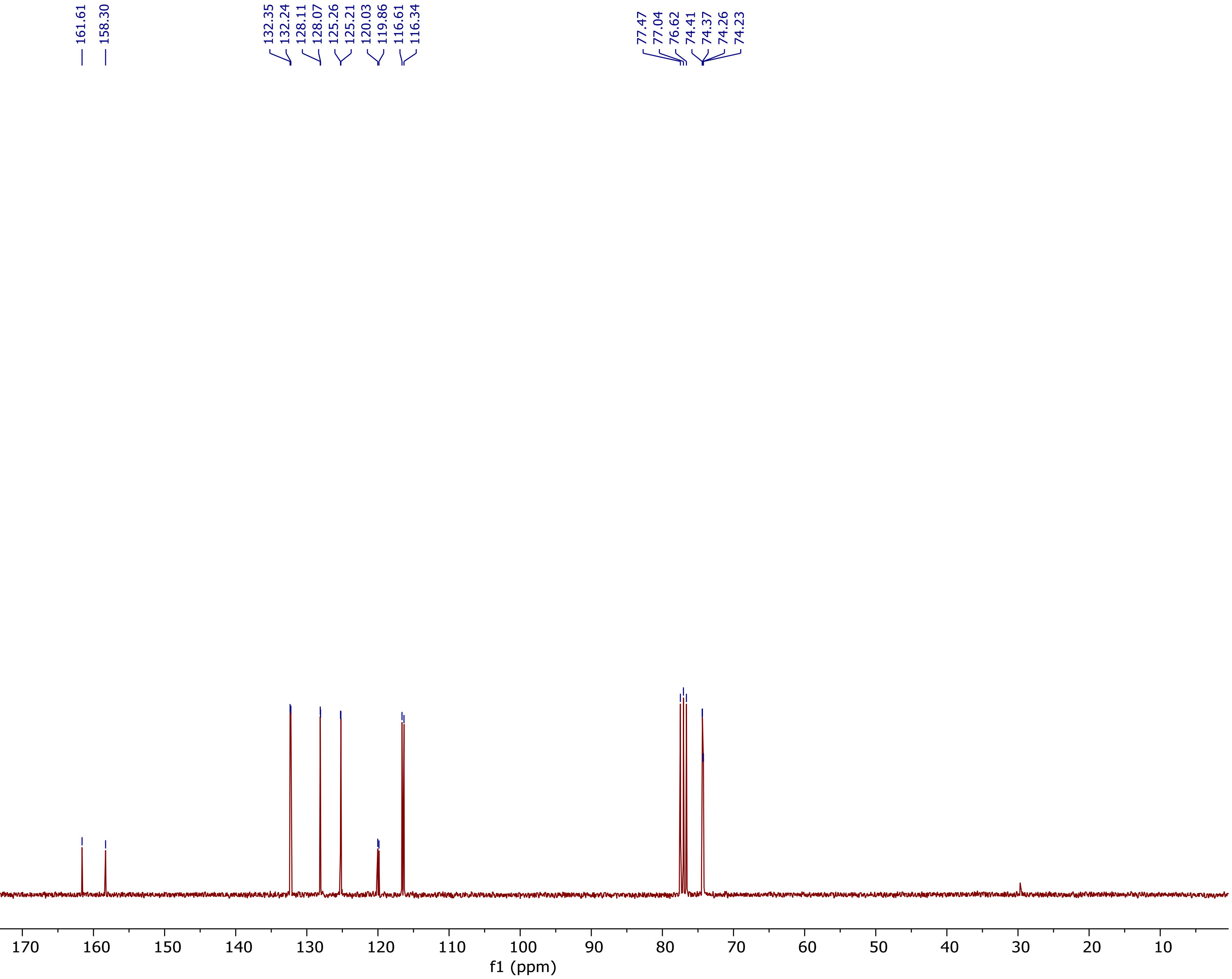

^{19}F NMR (282 MHz, $CDCl_3$) of **17**

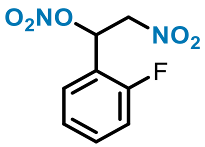

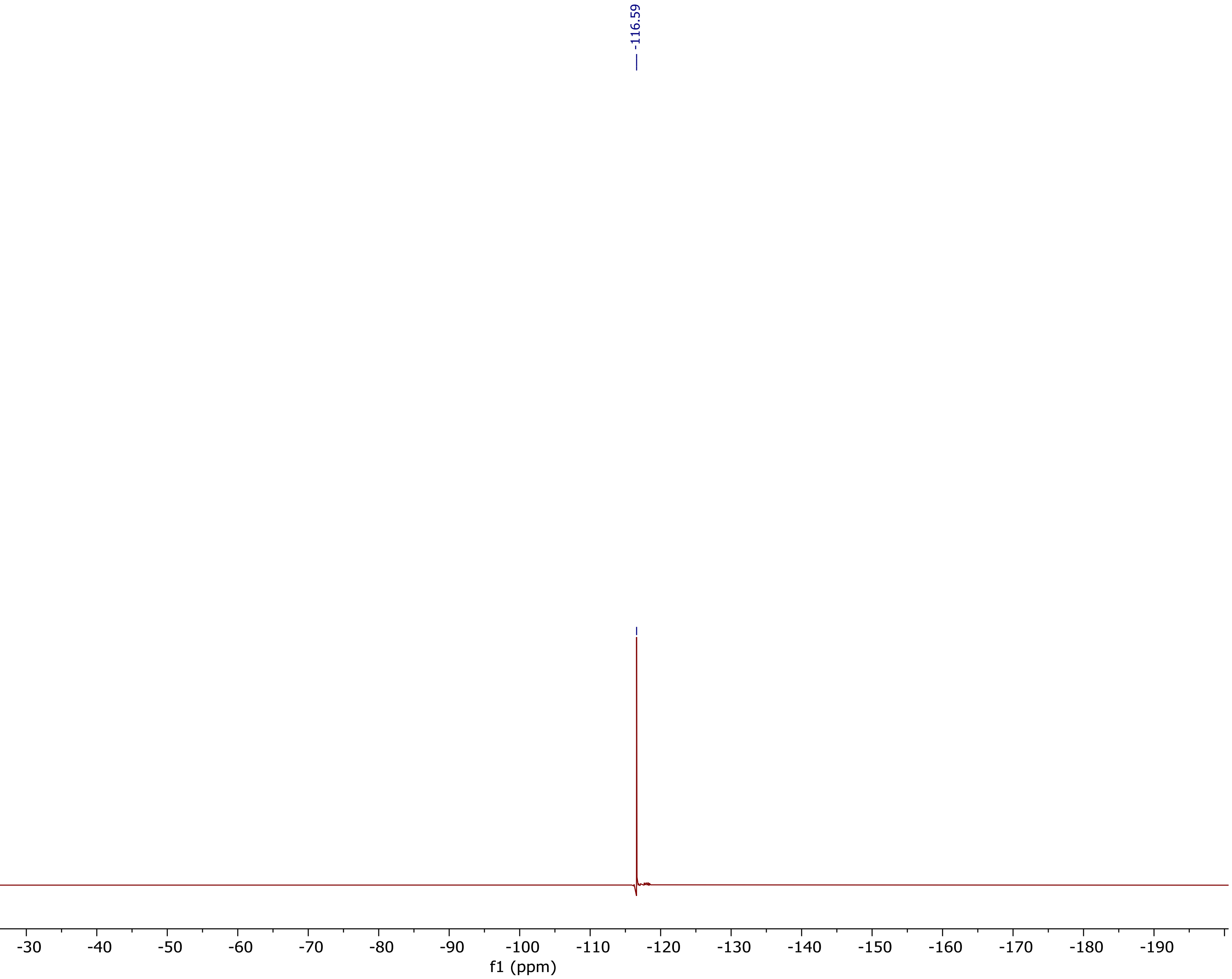

^{1}H NMR (300 MHz, $CDCl_3$) of **18**

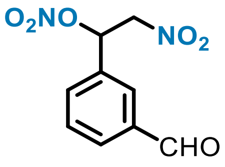

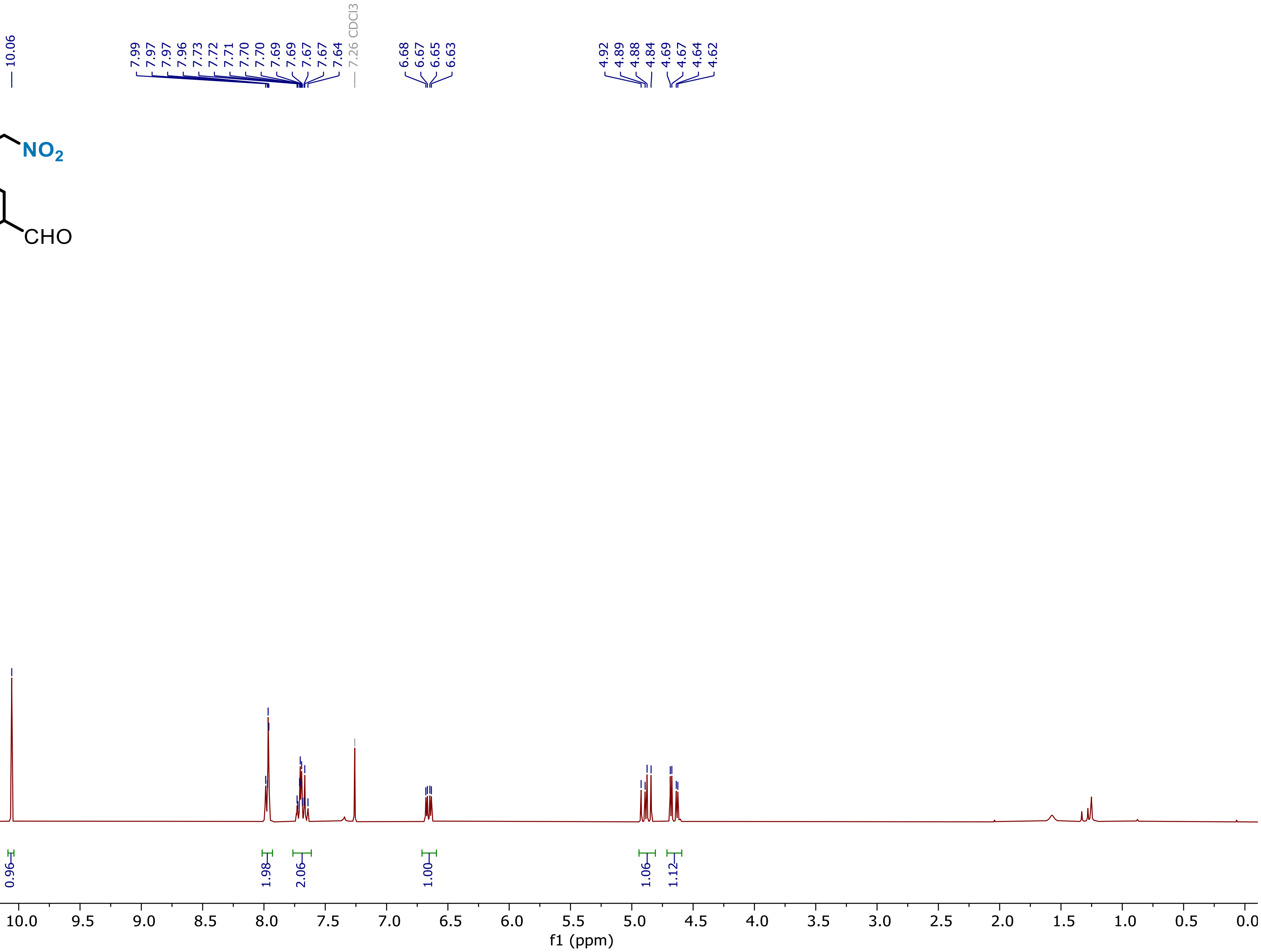

^{13}C NMR (75 MHz, $CDCl_3$) of **18**

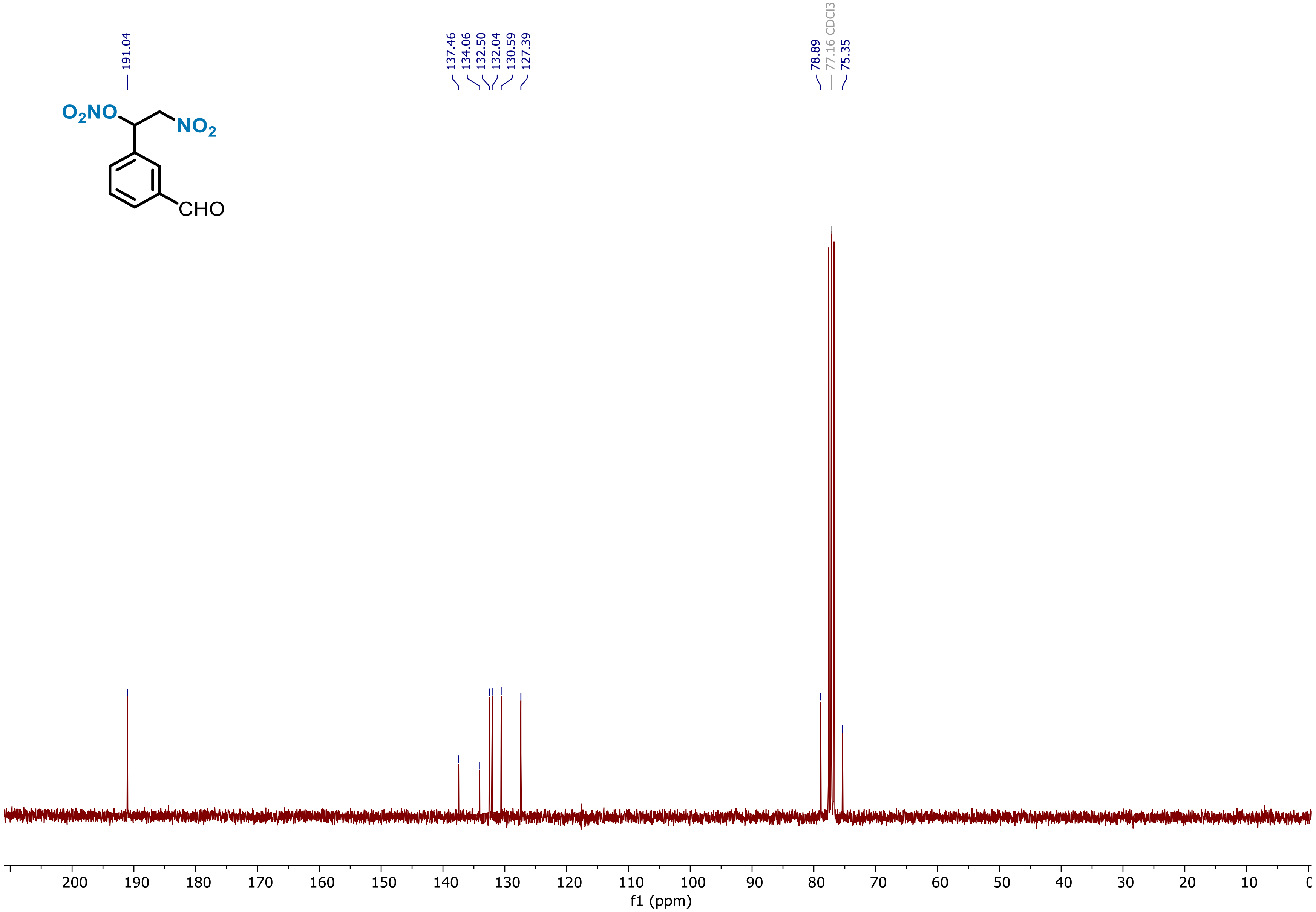

^{1}H NMR (300 MHz, $CDCl_3$) of **19**

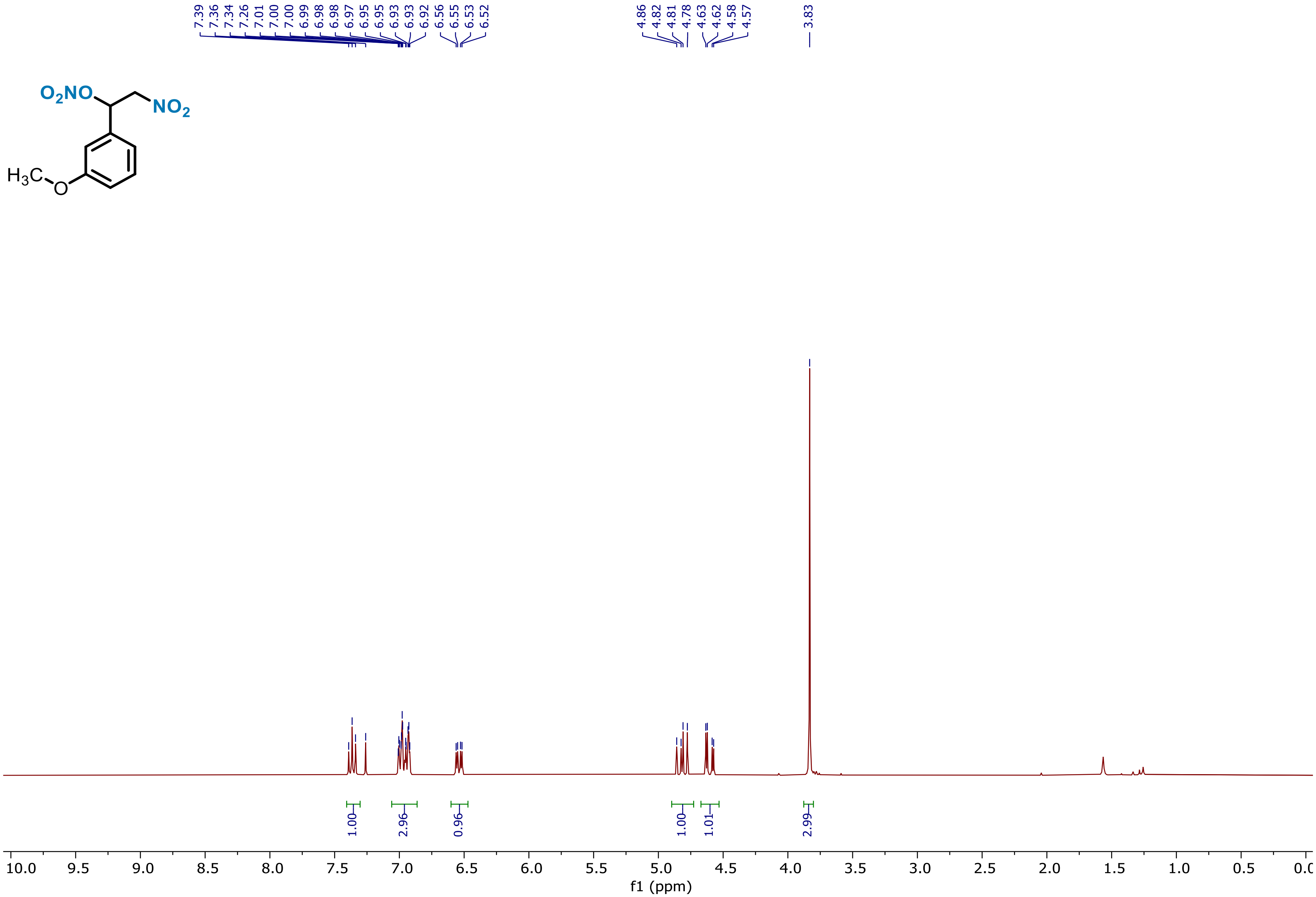

^{13}C NMR (75 MHz, $CDCl_3$) of **19**

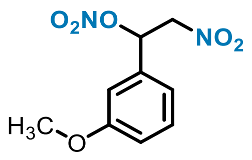

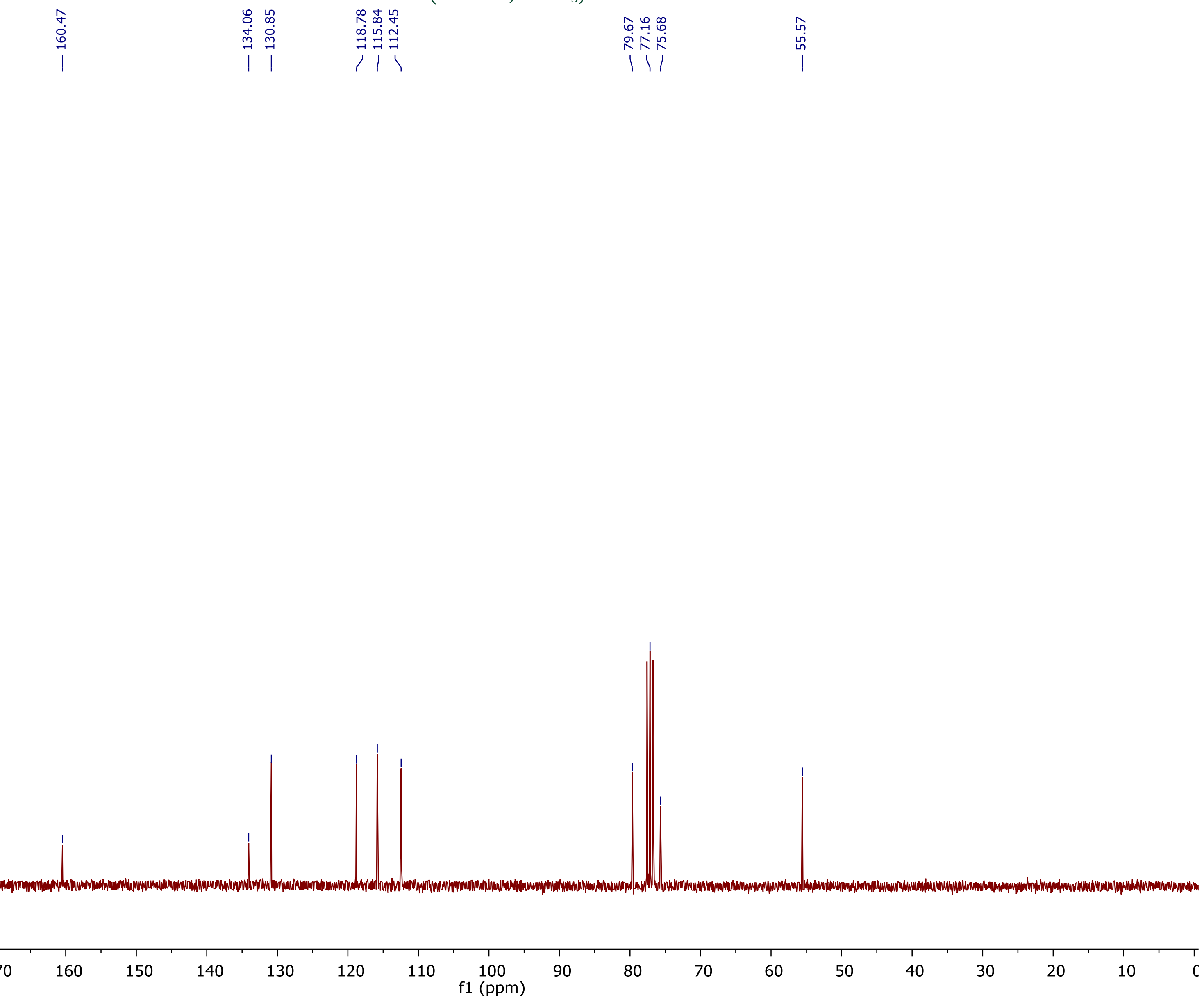

^{1}H NMR (300 MHz, $CDCl_3$) of **20**

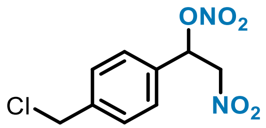

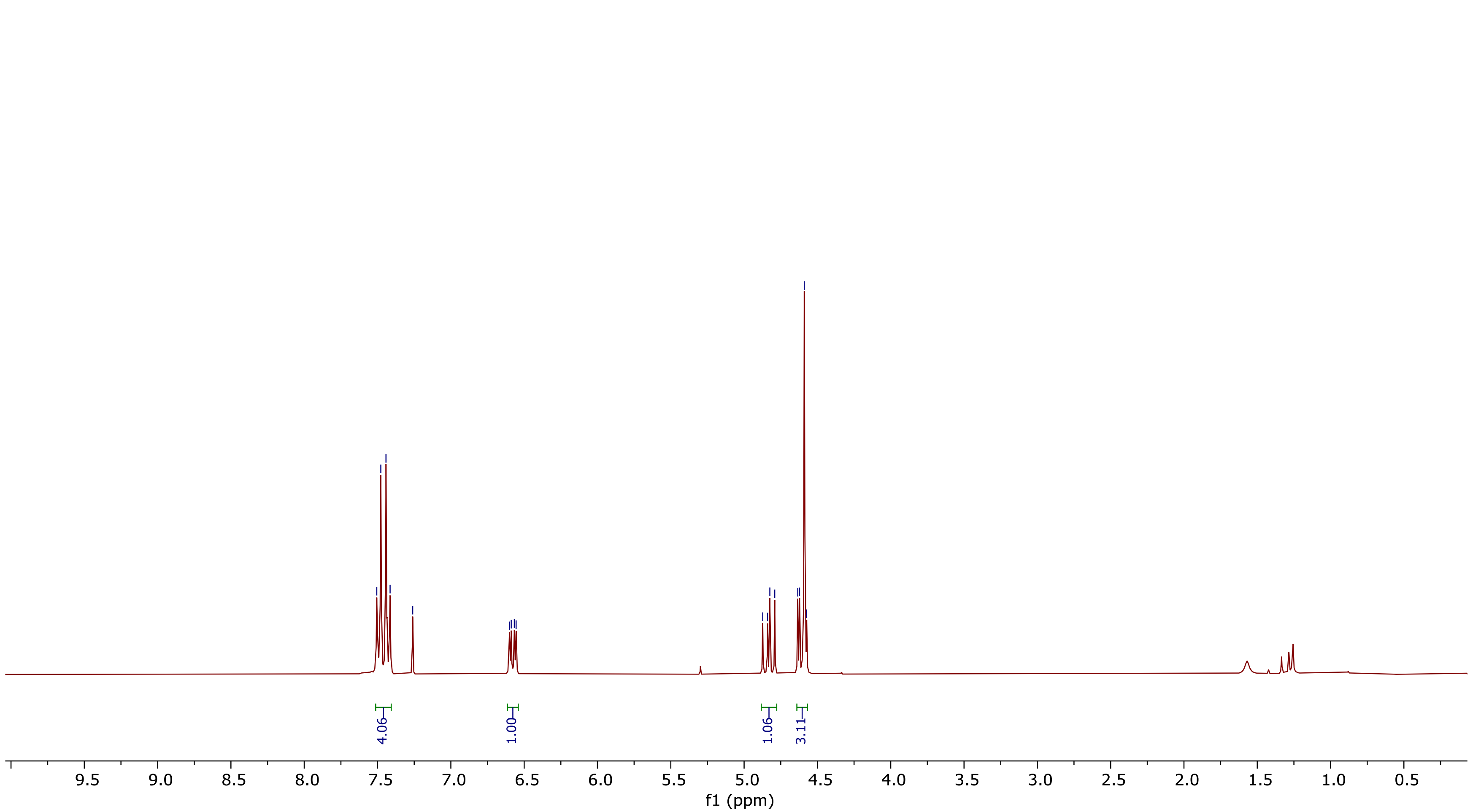

^{13}C NMR (75 MHz, $CDCl_3$) of **20**

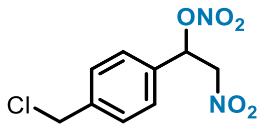

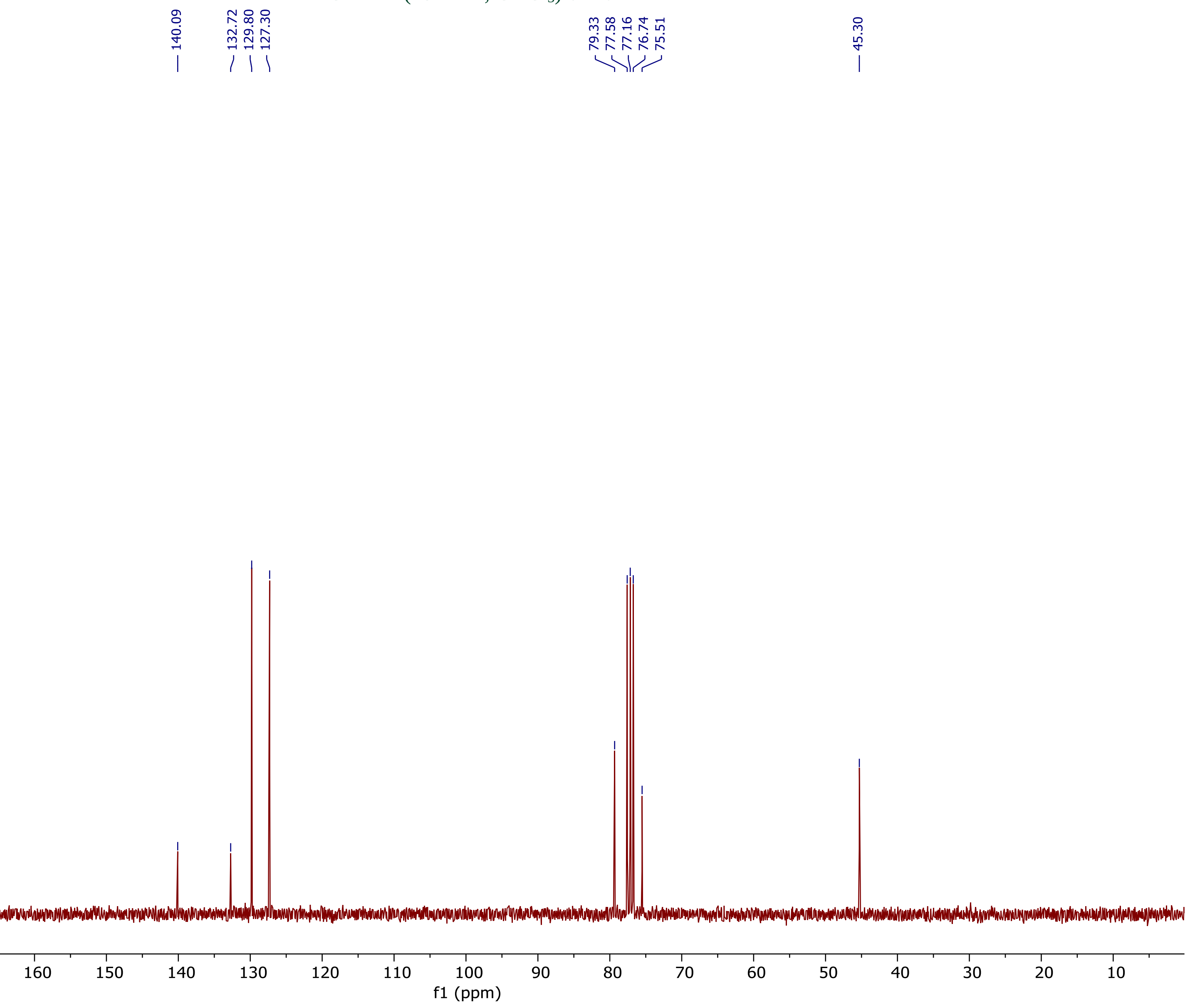

^{1}H NMR (300 MHz, $CDCl_3$) of **21**

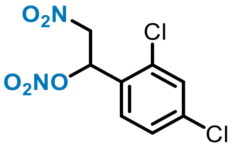

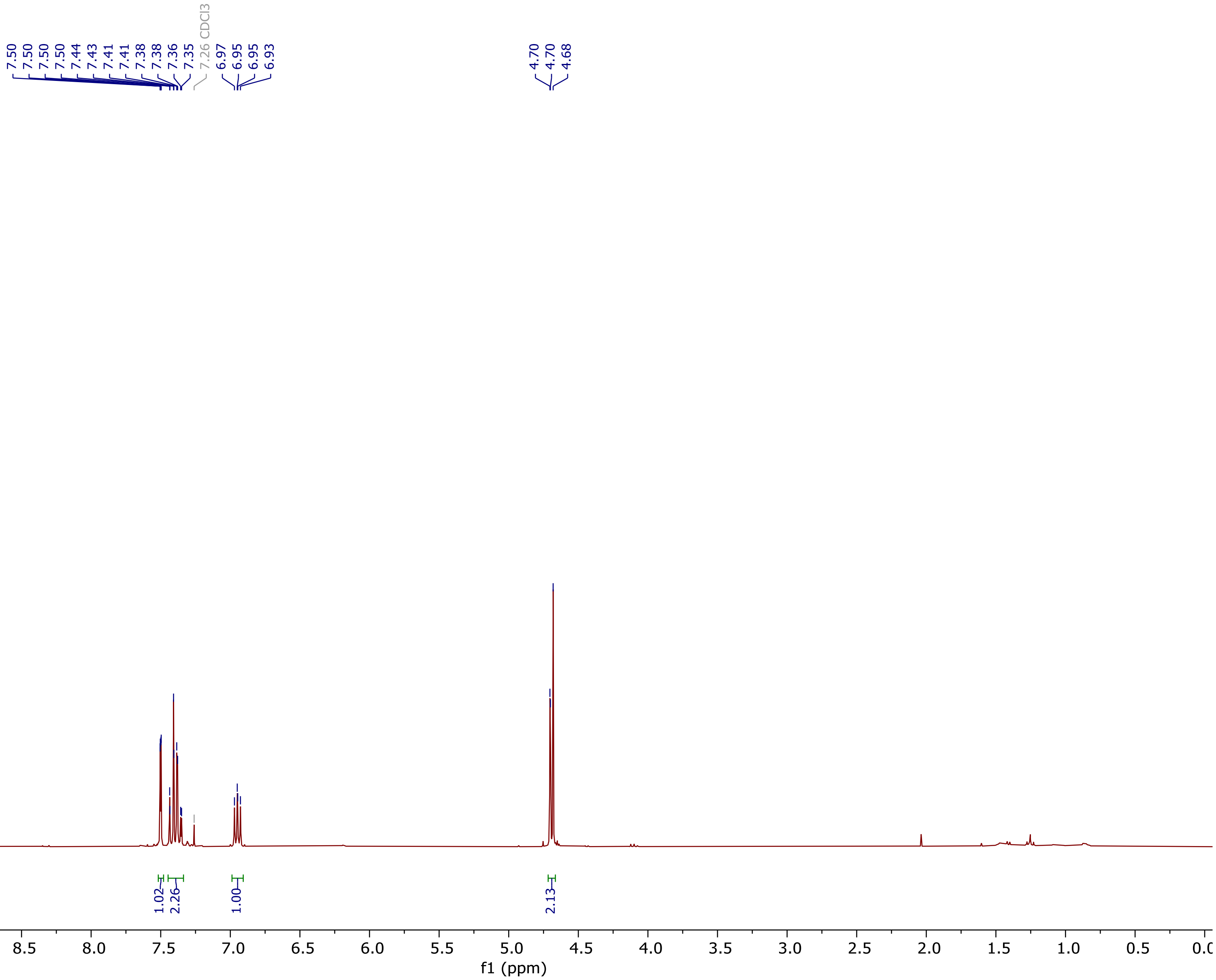

^{13}C NMR (75 MHz, $CDCl_3$) of **21**

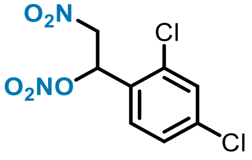

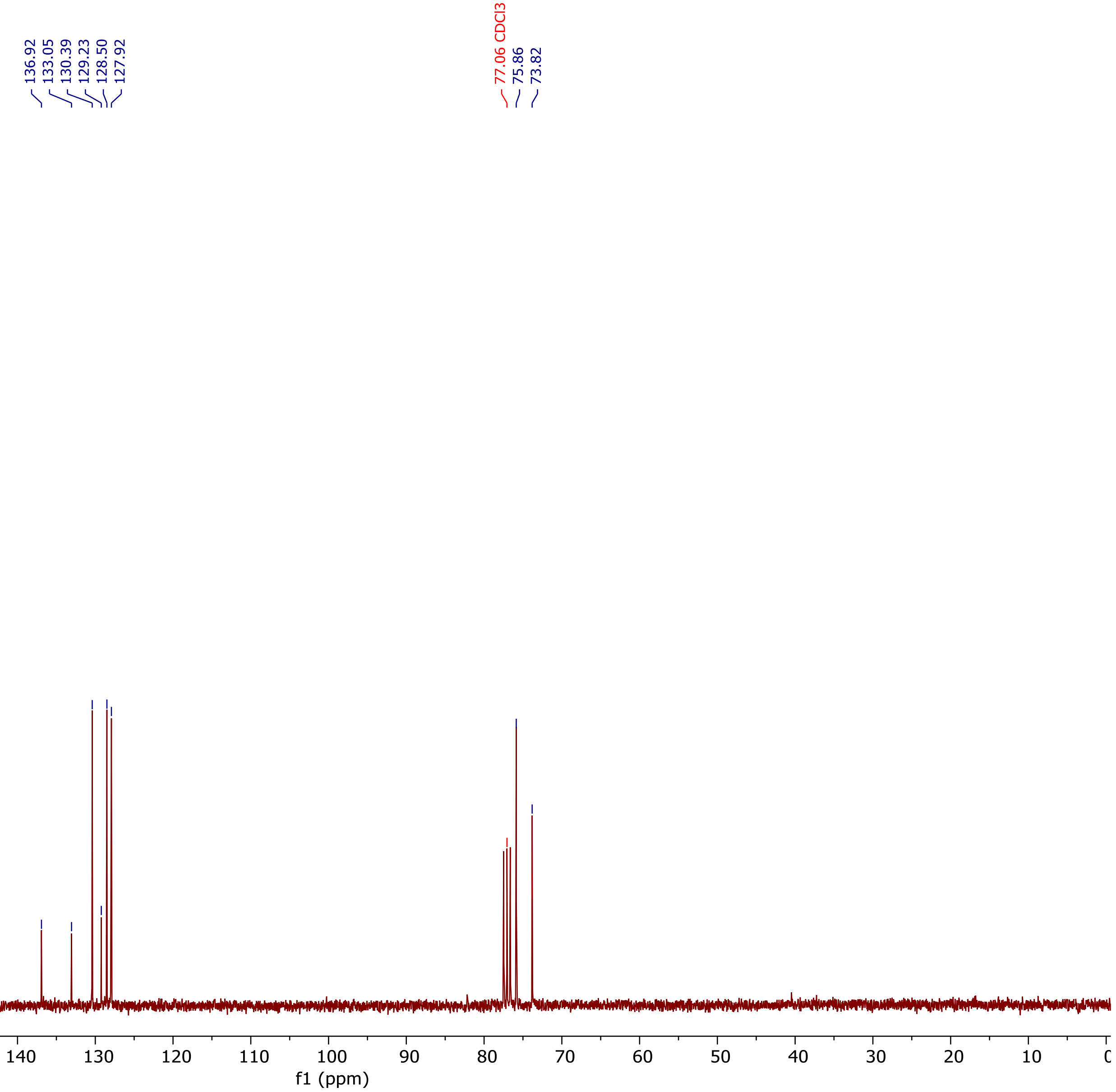

^{1}H NMR (300 MHz, $CDCl_3$) of **22**

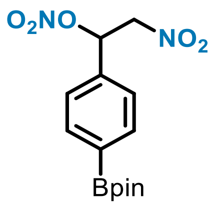

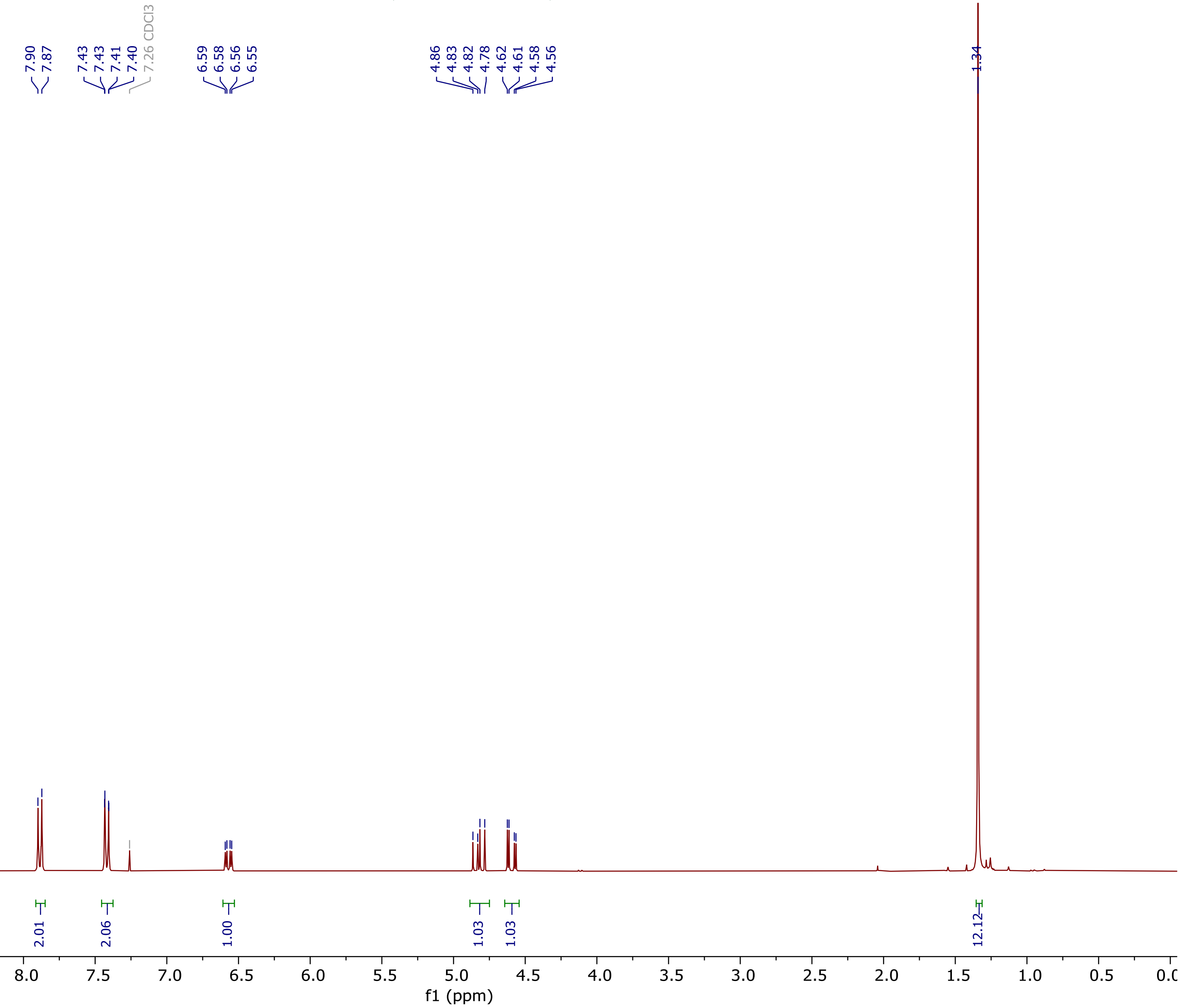

^{13}C NMR (75 MHz, $CDCl_3$) of **22**

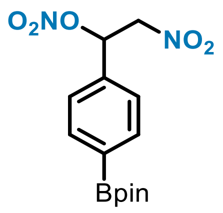

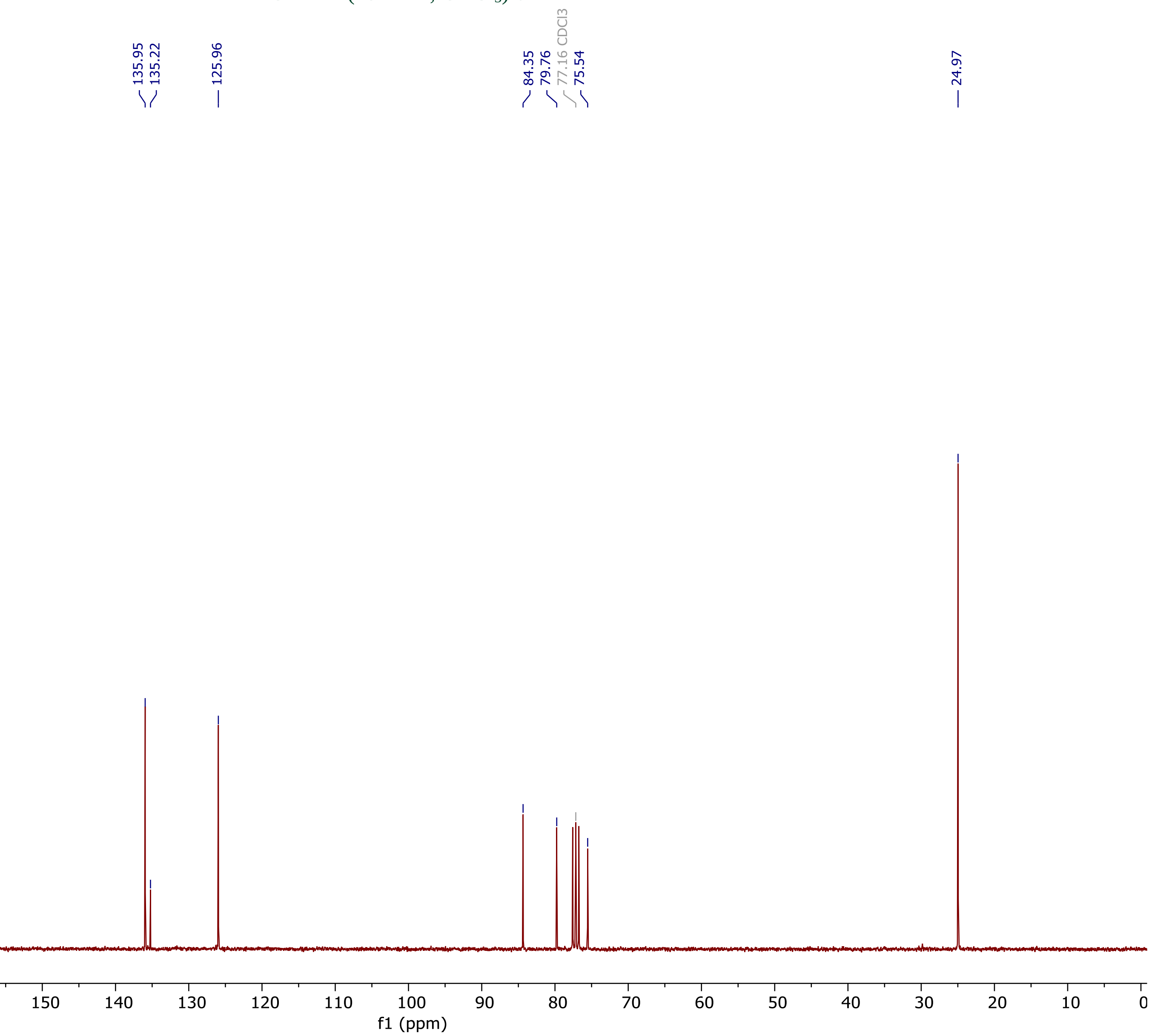

^1H NMR (300 MHz, $CDCl_3$) of **23**

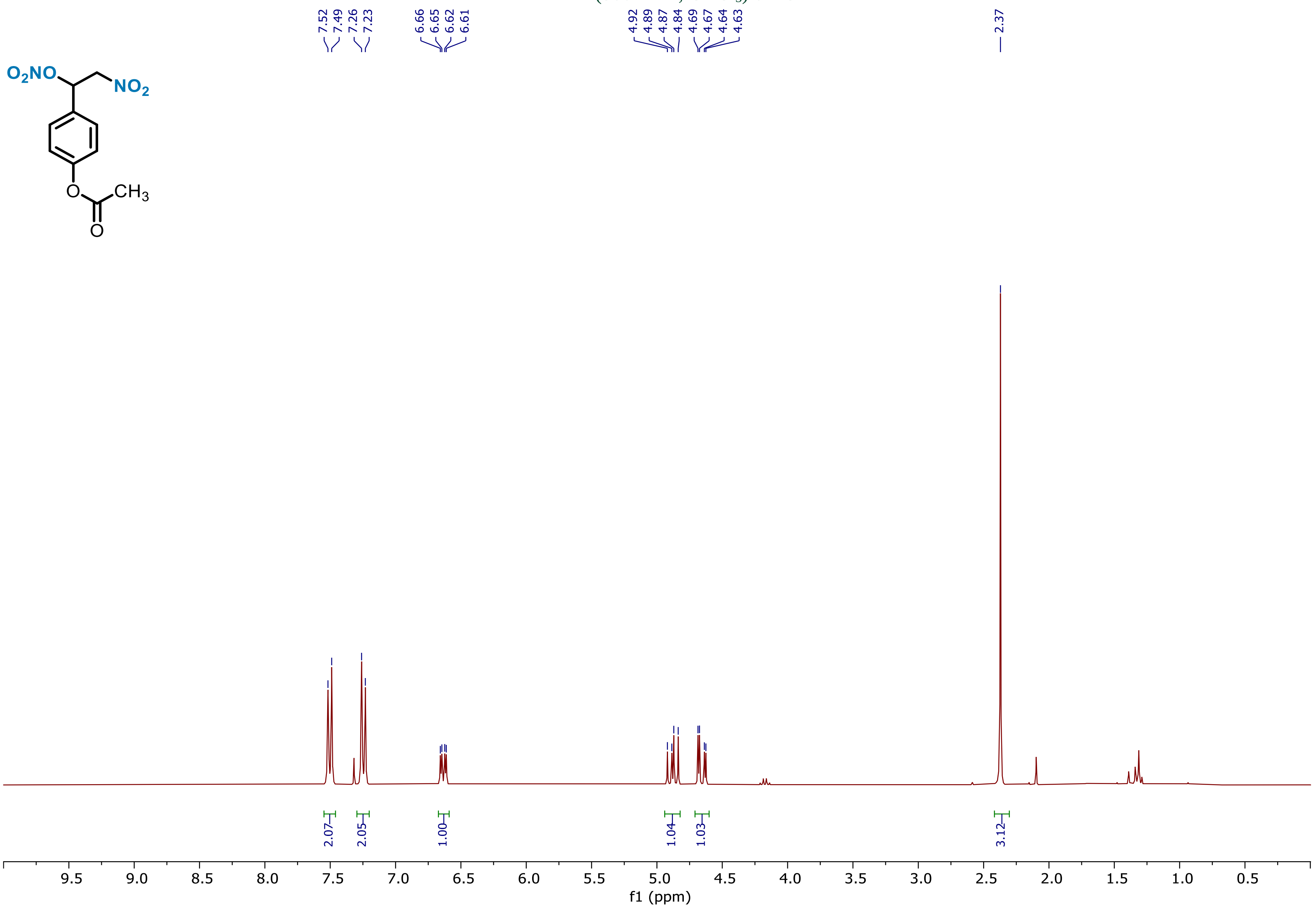

^{13}C NMR (75 MHz, $CDCl_3$) of **23**

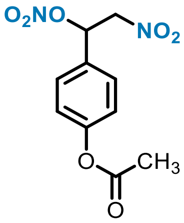

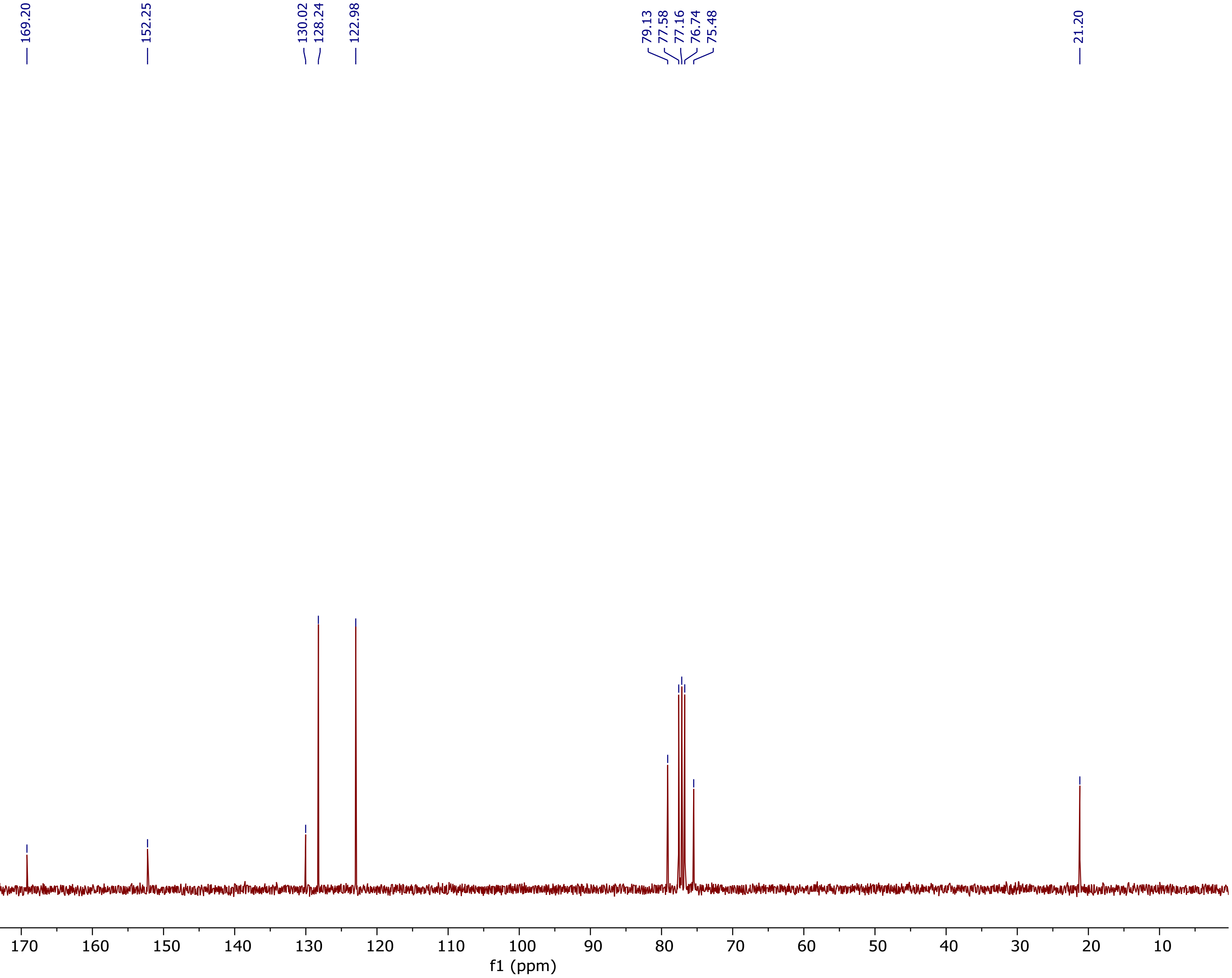

^{1}H NMR (300 MHz, $CDCl_3$) of **24** (unstable, yield is based on NMR purity of isolated compound)

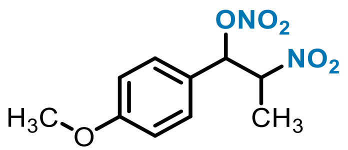

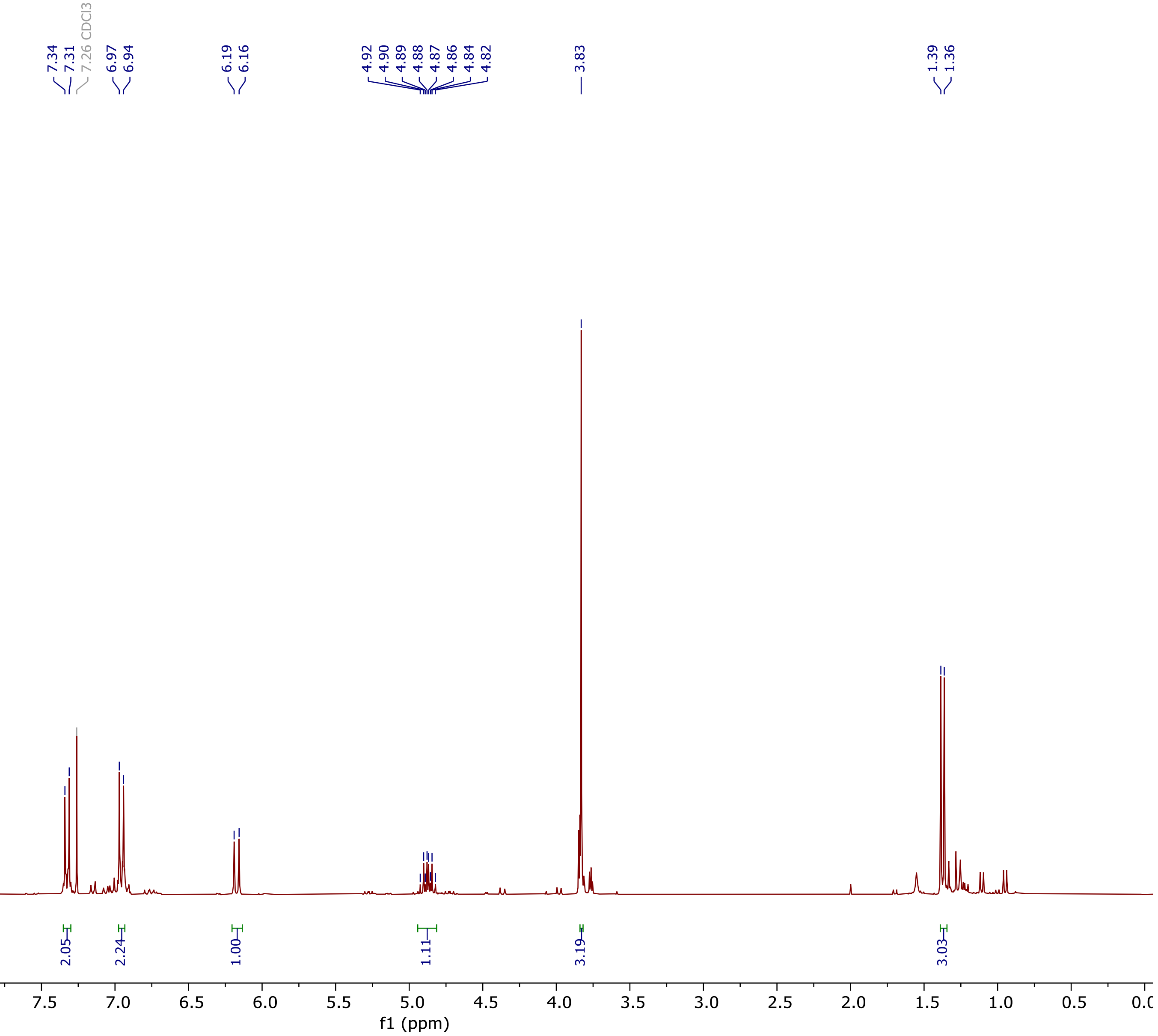

^{13}C NMR (75 MHz, $CDCl_3$) of **24**

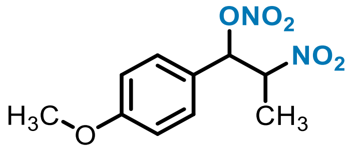

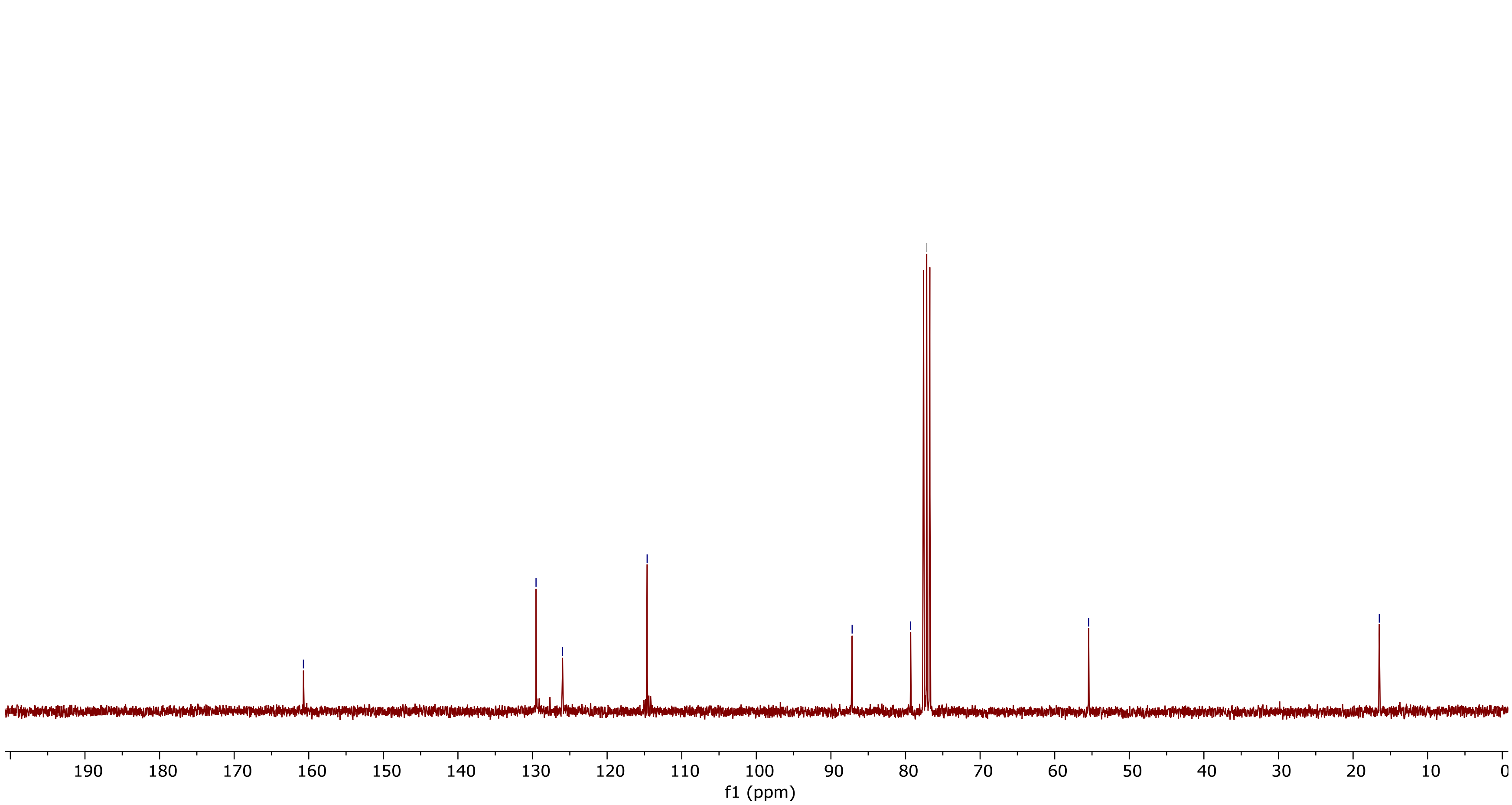

^{1}H NMR (300 MHz, $CDCl_3$) of **25** (unstable, yield is based on NMR purity of isolated compound)

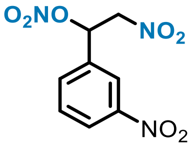

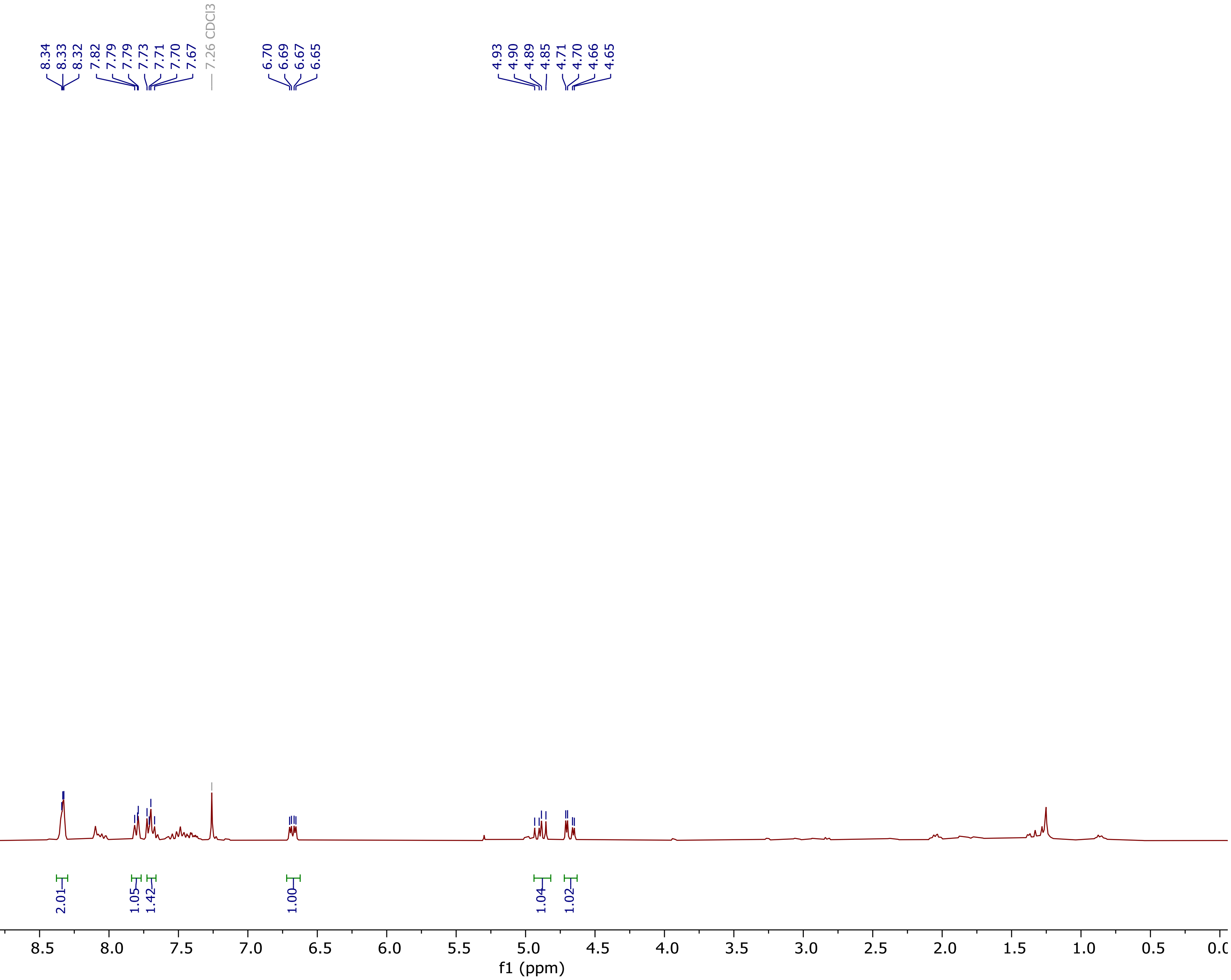

^{13}C NMR (75 MHz, CDCl$_3$) of **25**

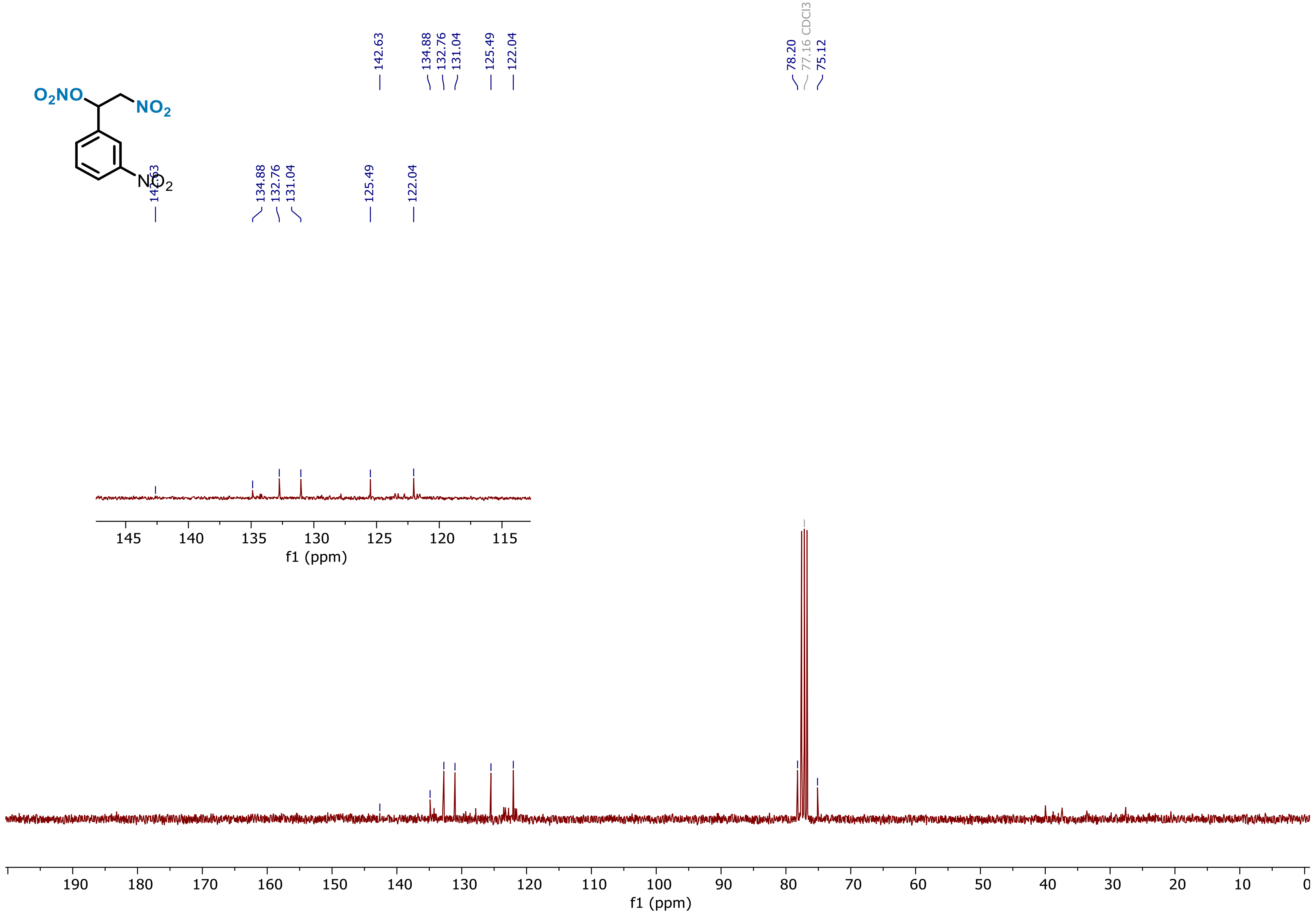

^{1}H NMR (300 MHz, $CDCl_3$) of **26**

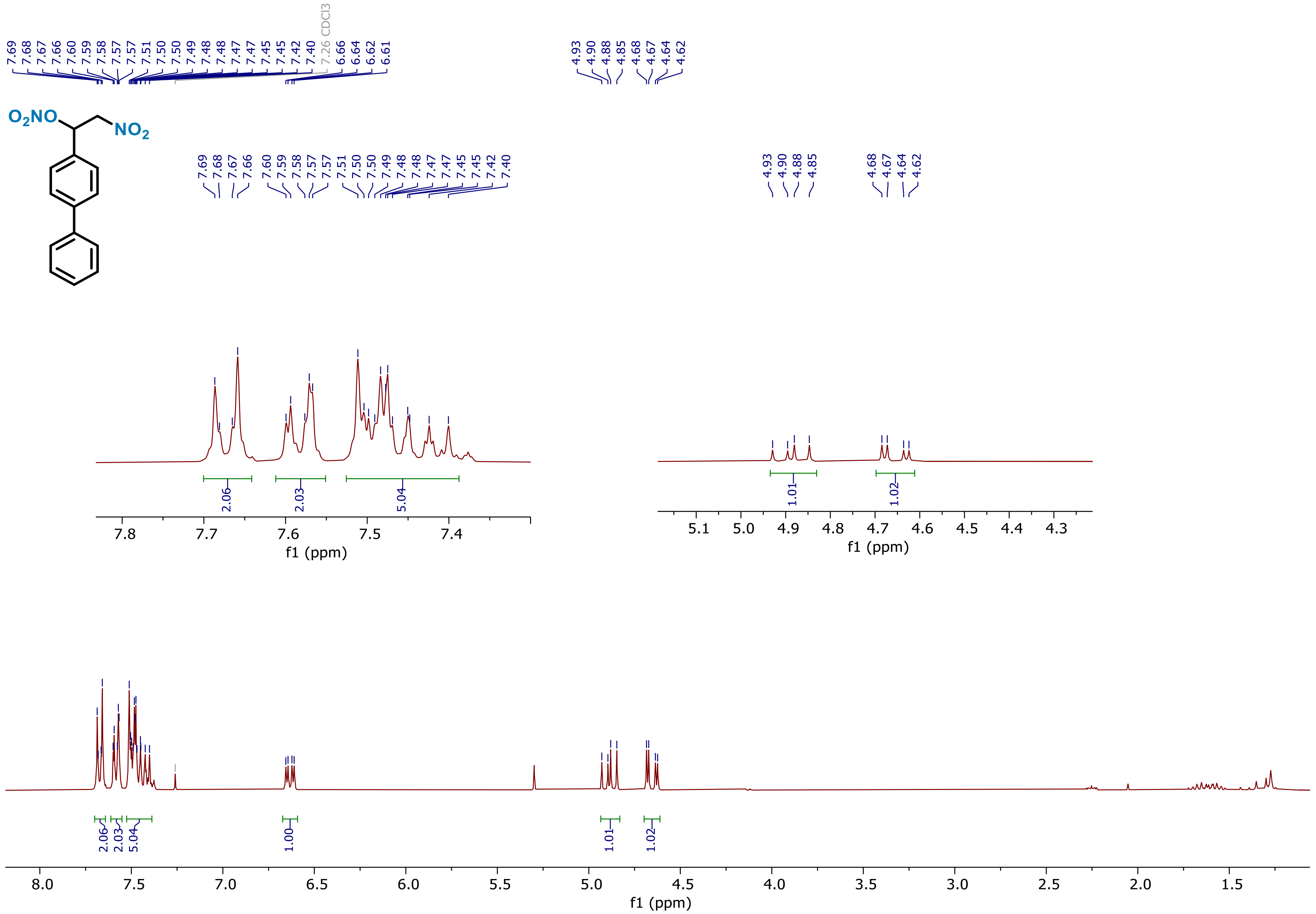

^{13}C NMR (75 MHz, $CDCl_3$) of **26**

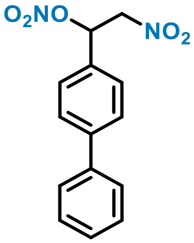

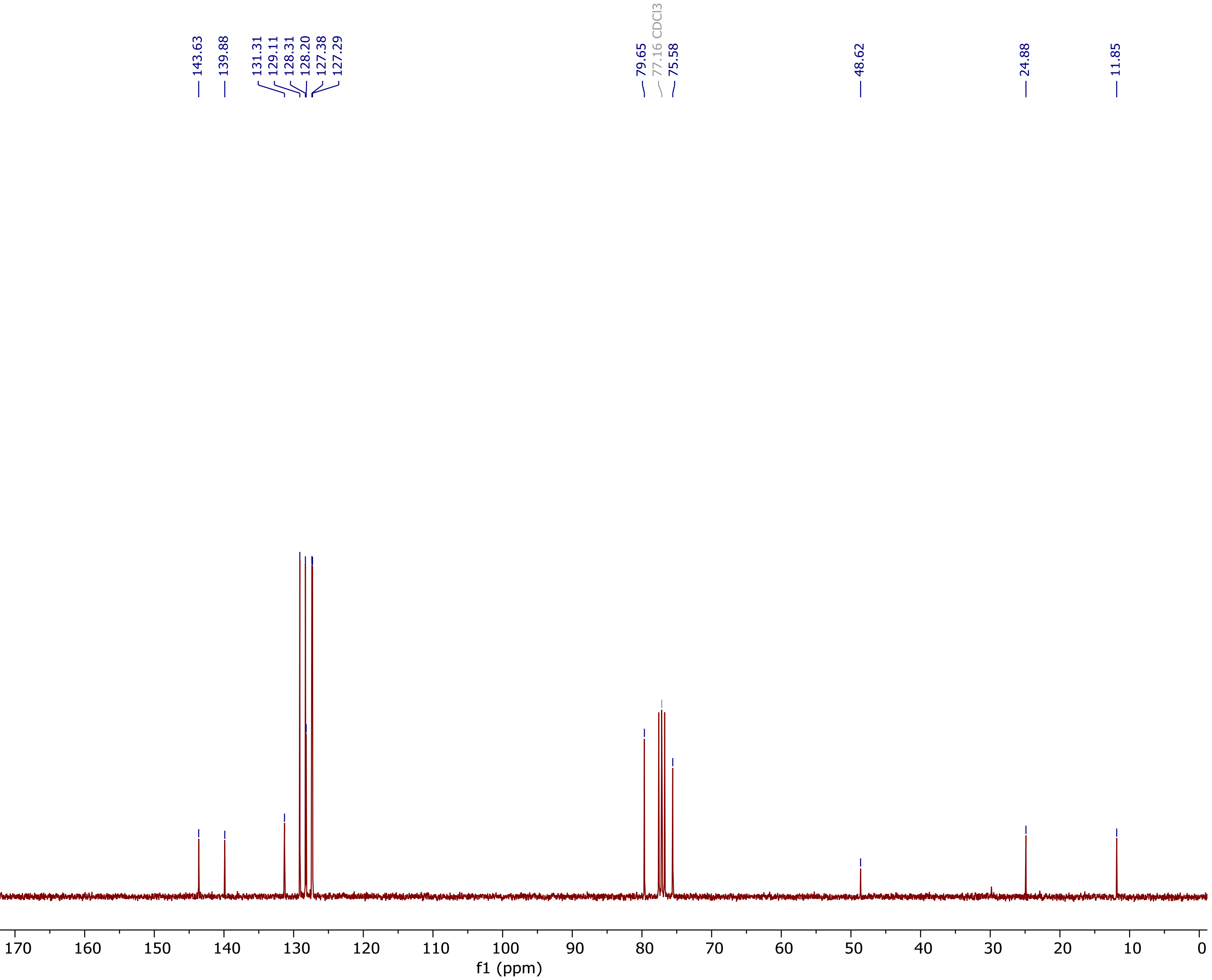

^{1}H NMR (300 MHz, $CDCl_3$) of **27**

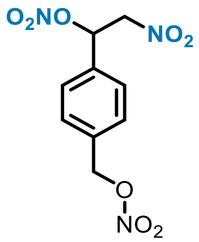

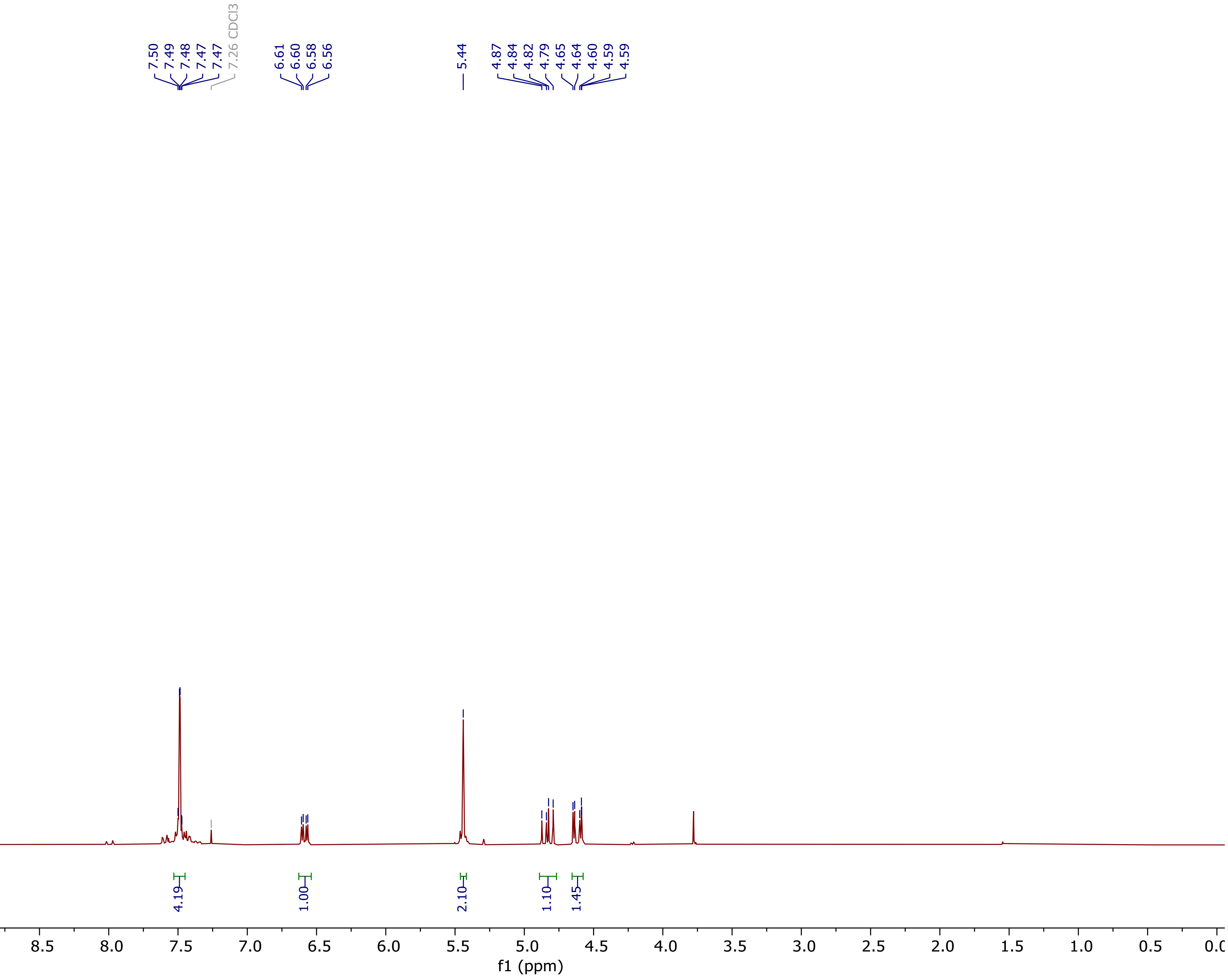

^{13}C NMR (75 MHz, $CDCl_3$) of **27**

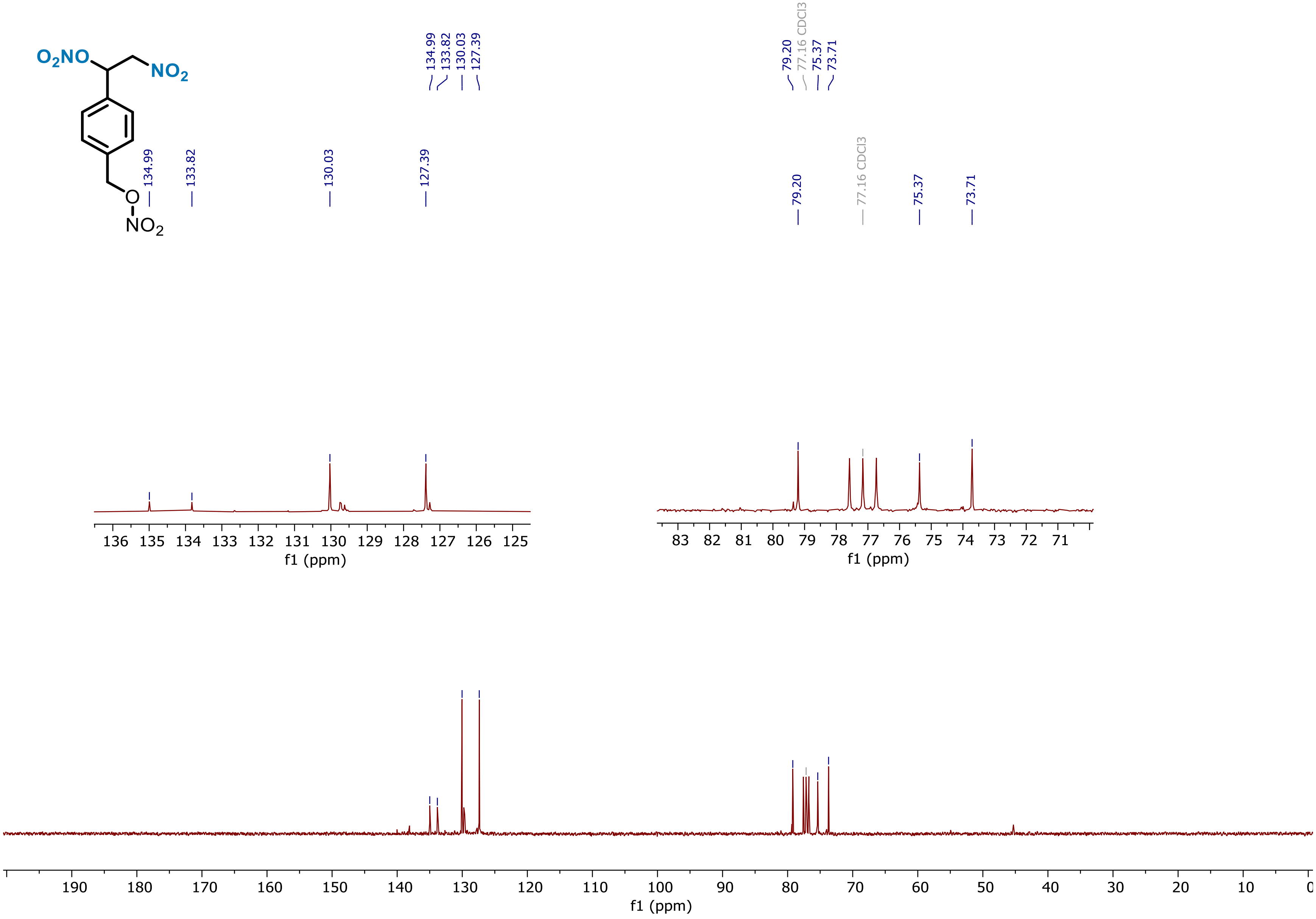

^{1}H NMR (300 MHz, $CDCl_3$) of **28**

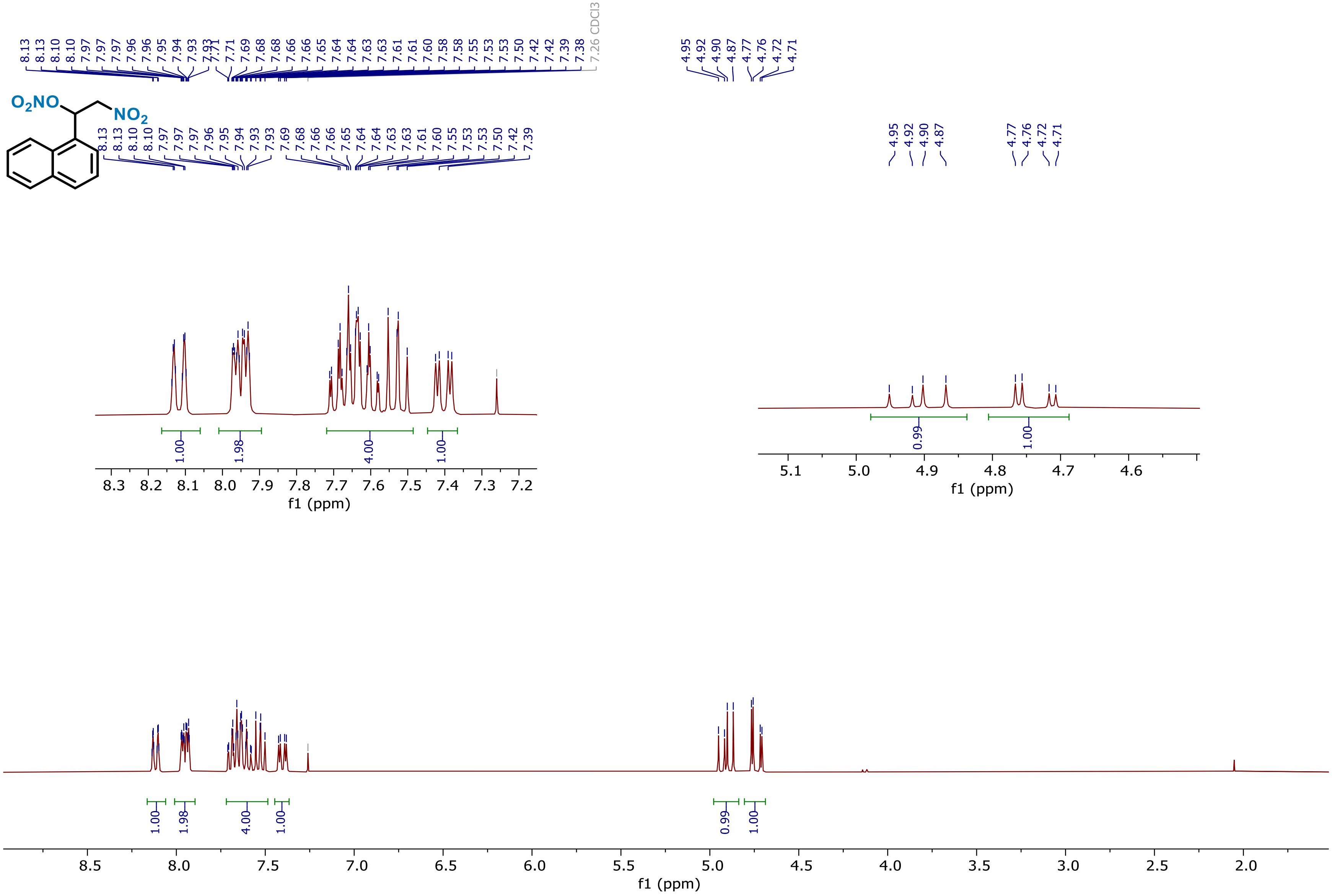

^{13}C NMR (75 MHz, $CDCl_3$) of **28**

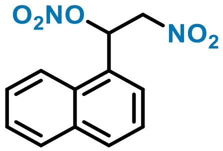

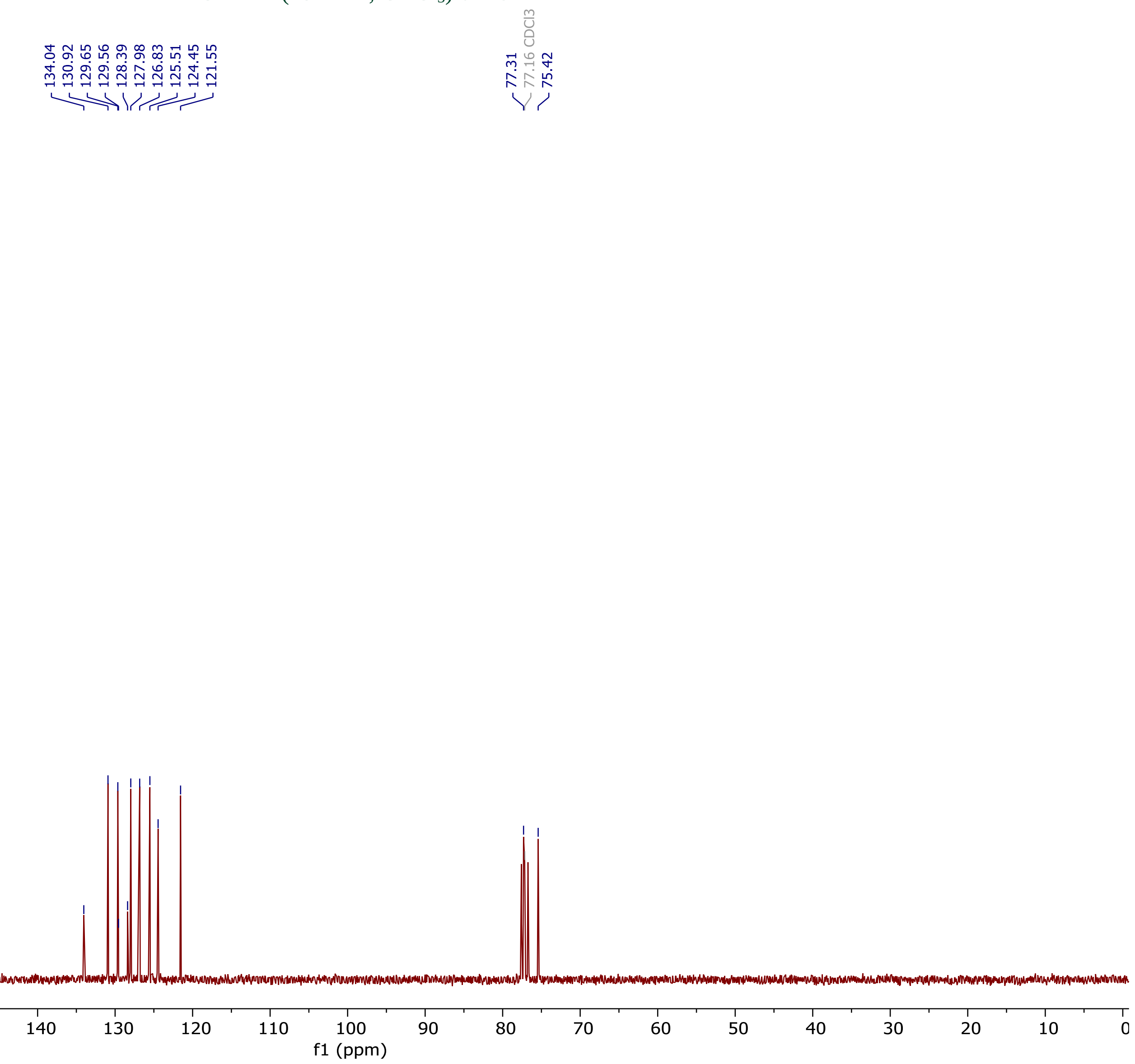

^{1}H NMR (300 MHz, $CDCl_3$) of **29**

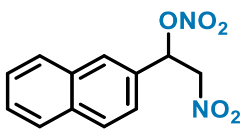

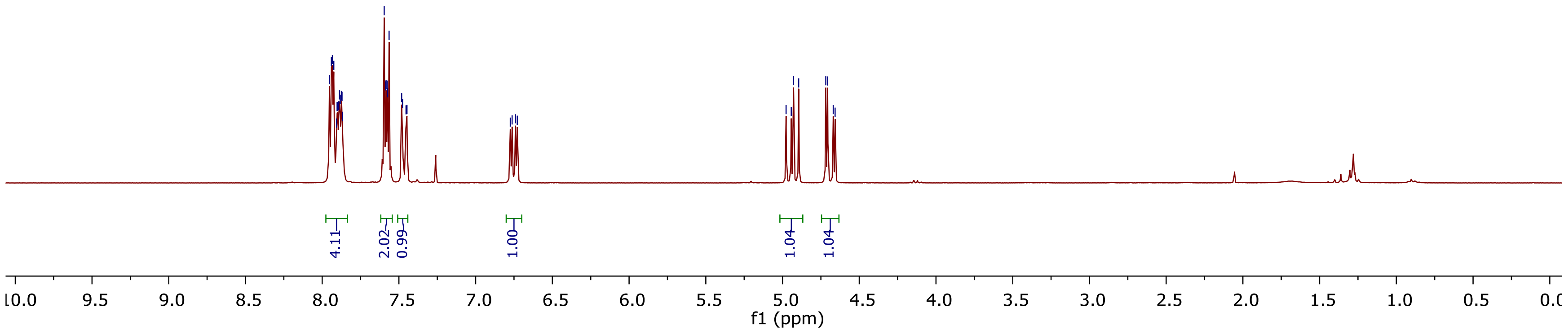

^{13}C NMR (75 MHz, $CDCl_3$) of **29**

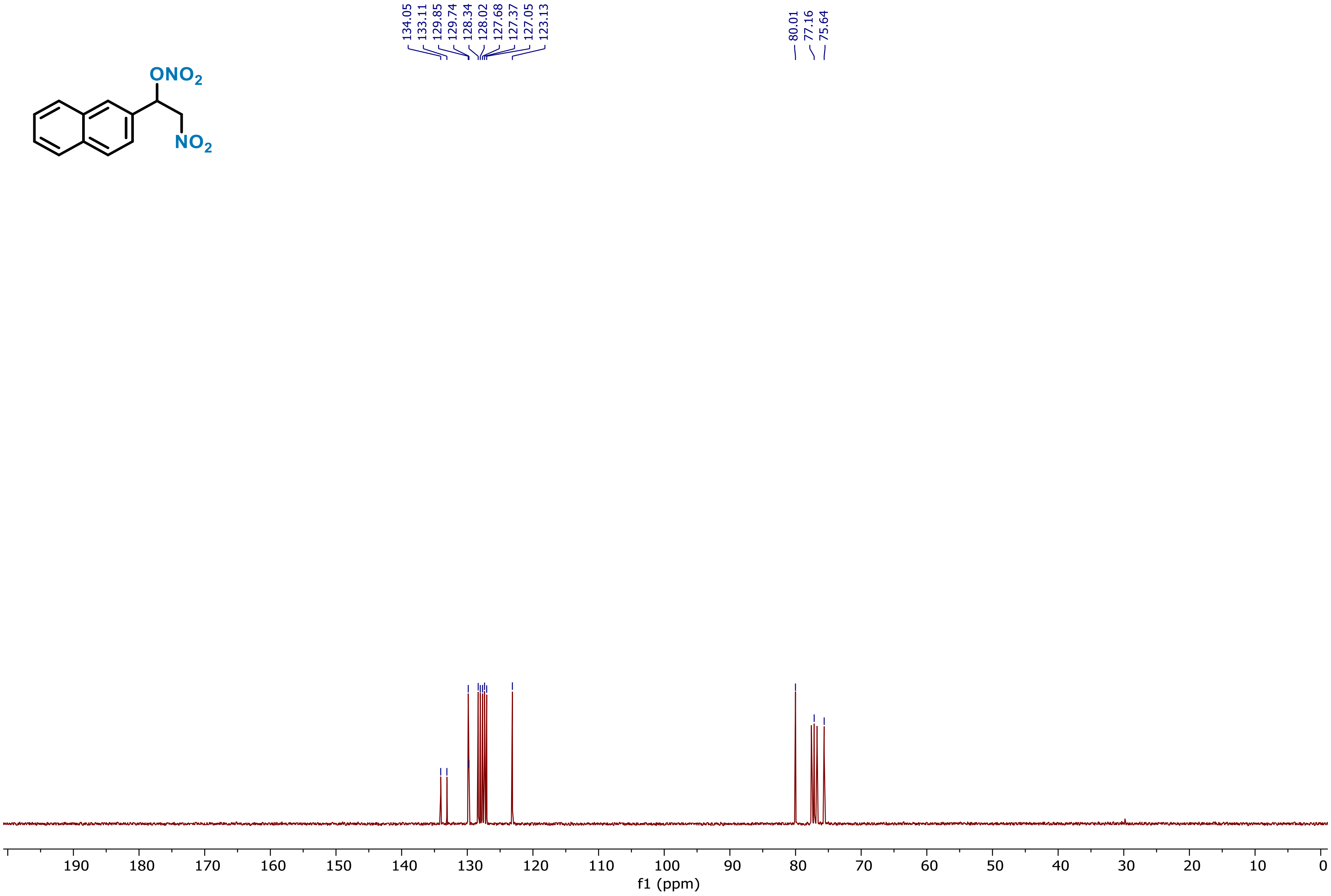

^{1}H NMR (300 MHz, $CDCl_3$) of **30**

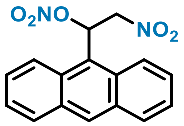

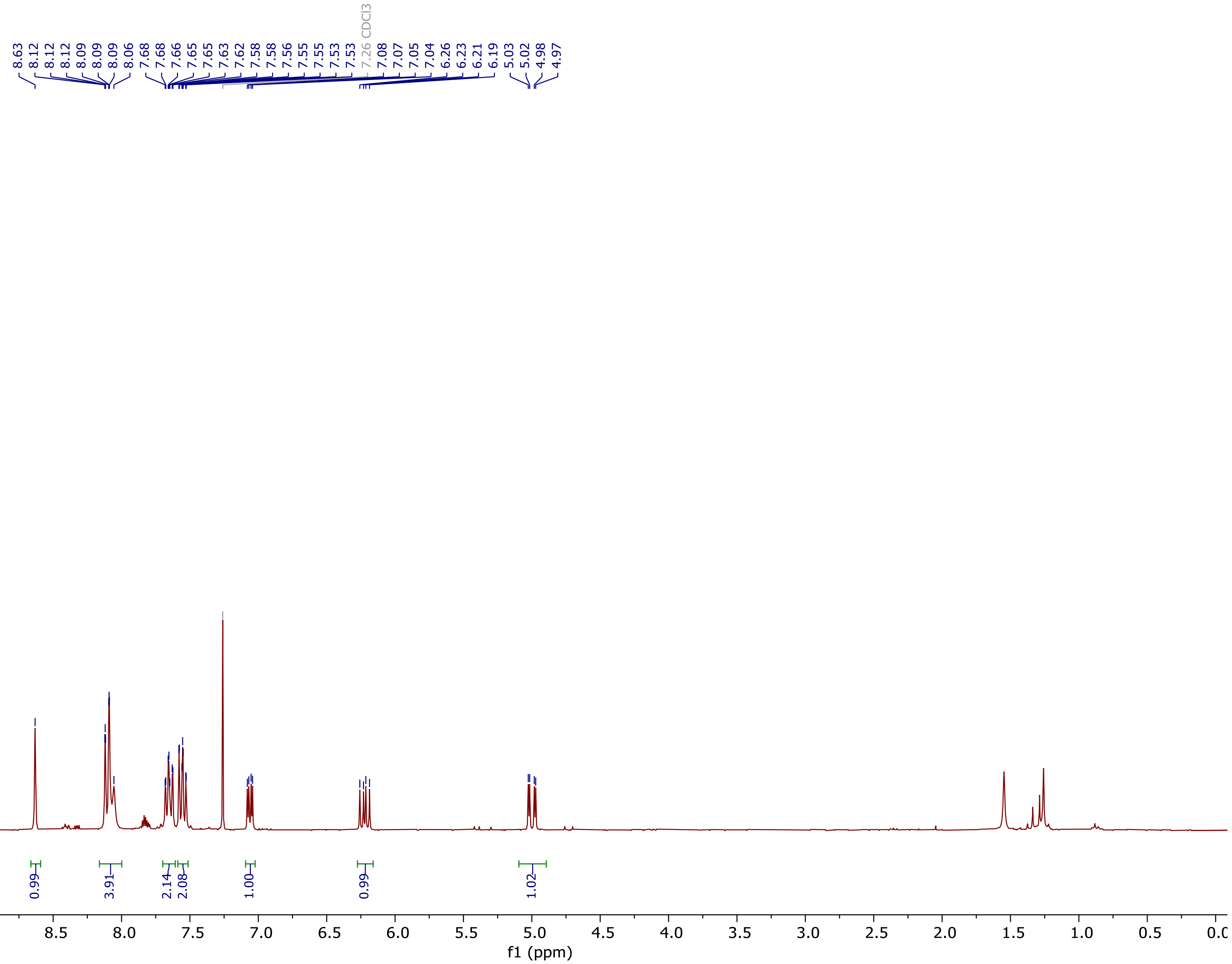

^{13}C NMR (75 MHz, $CDCl_3$) of **30**

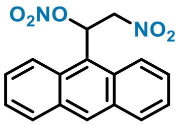

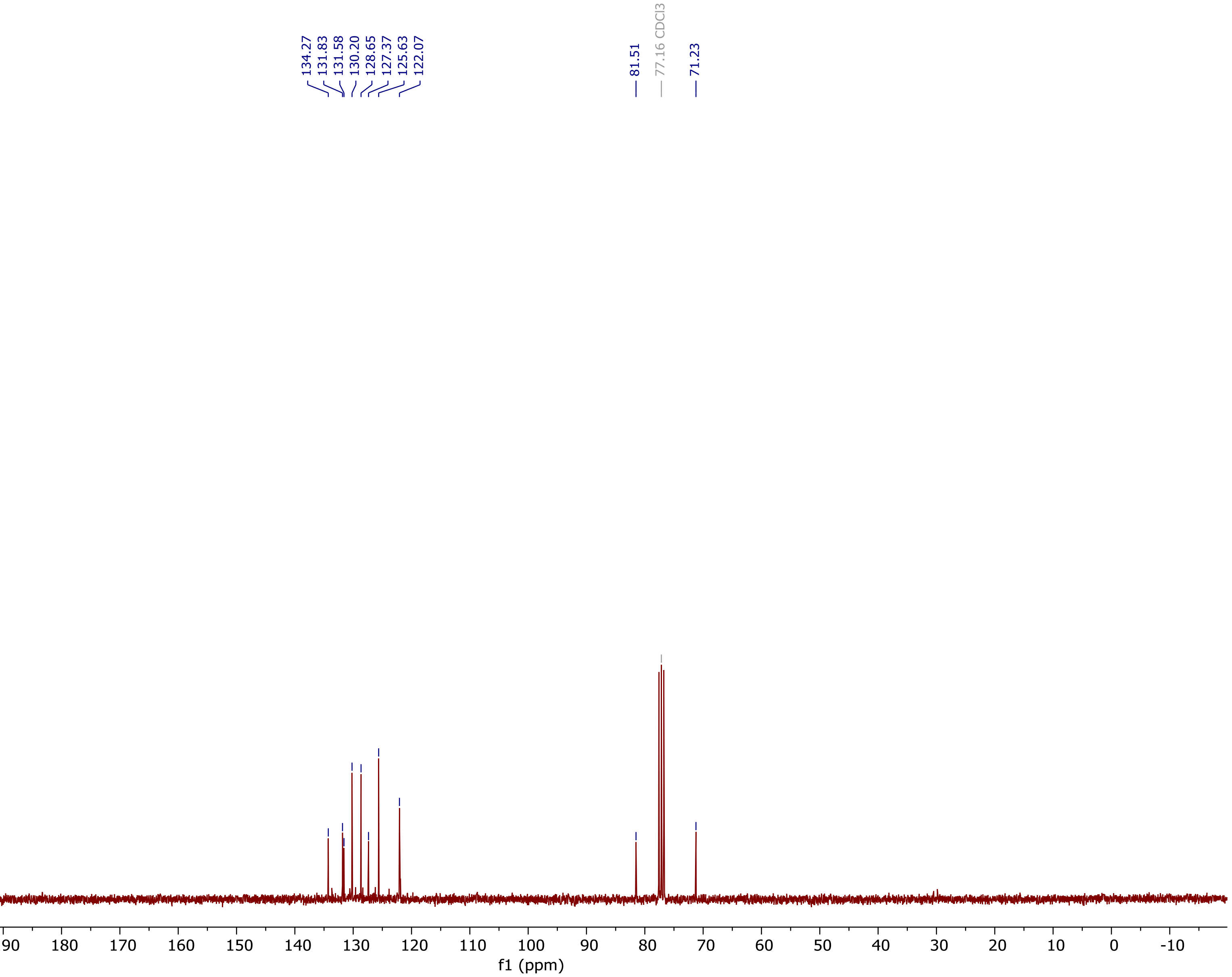

^{1}H NMR (300 MHz, $CDCl_3$) of **31**

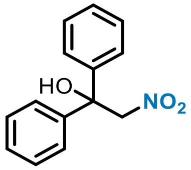
HO
NO_2

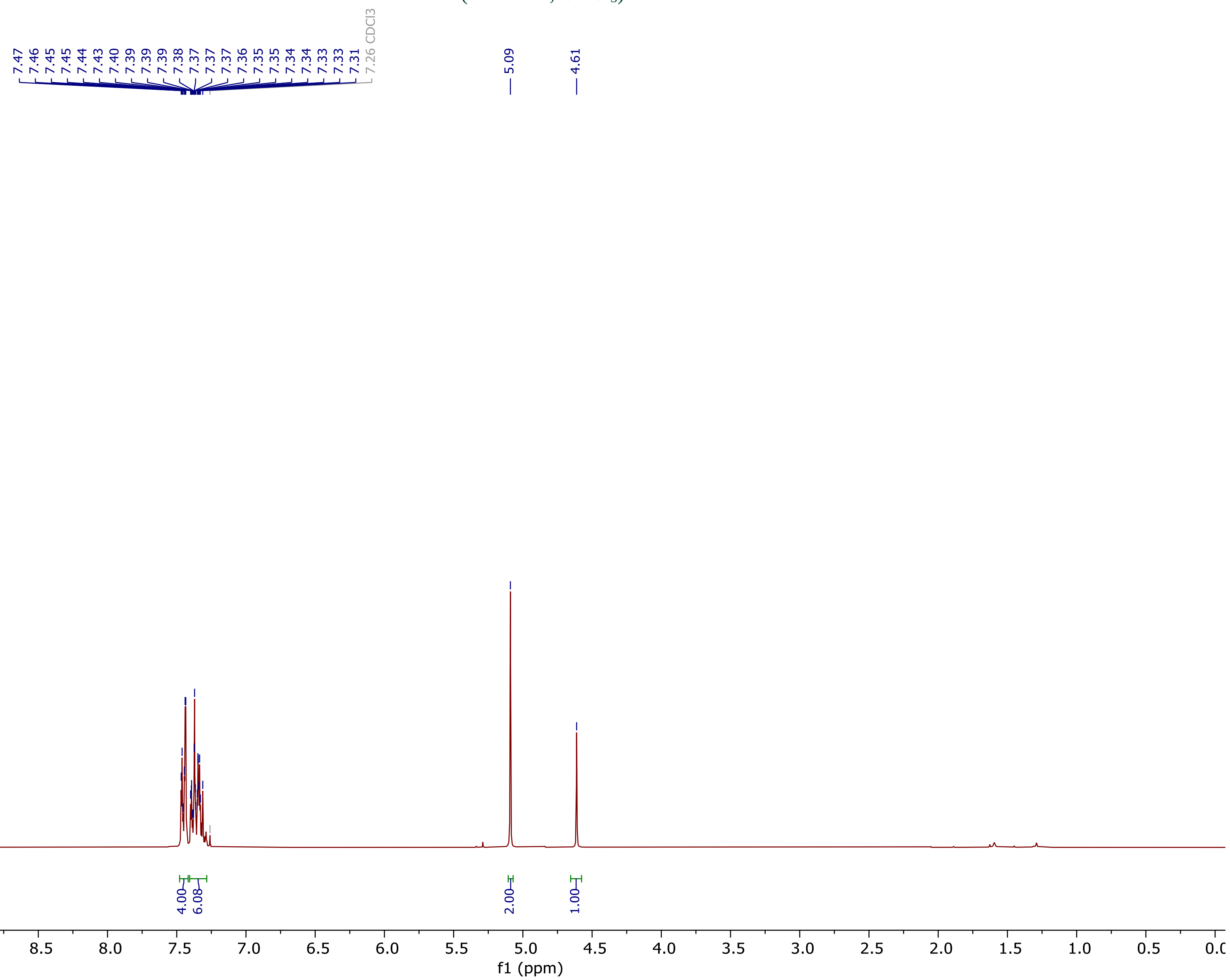
7.47
7.46
7.45
7.45
7.44
7.43
7.40
7.39
7.39
7.39
7.38
7.37
7.37
7.37
7.36
7.35
7.35
7.34
7.34
7.33
7.33
7.31
7.26 CDCl3
5.09
4.61
4.00
6.08
2.00
1.00
9.5
9.0
8.5
8.0
7.5
7.0
6.5
6.0
5.5
5.0
4.5
4.0
3.5
3.0
2.5
2.0
1.5
1.0
0.5
0.0
f1 (ppm)

^{13}C NMR (75 MHz, $CDCl_3$) of **31**

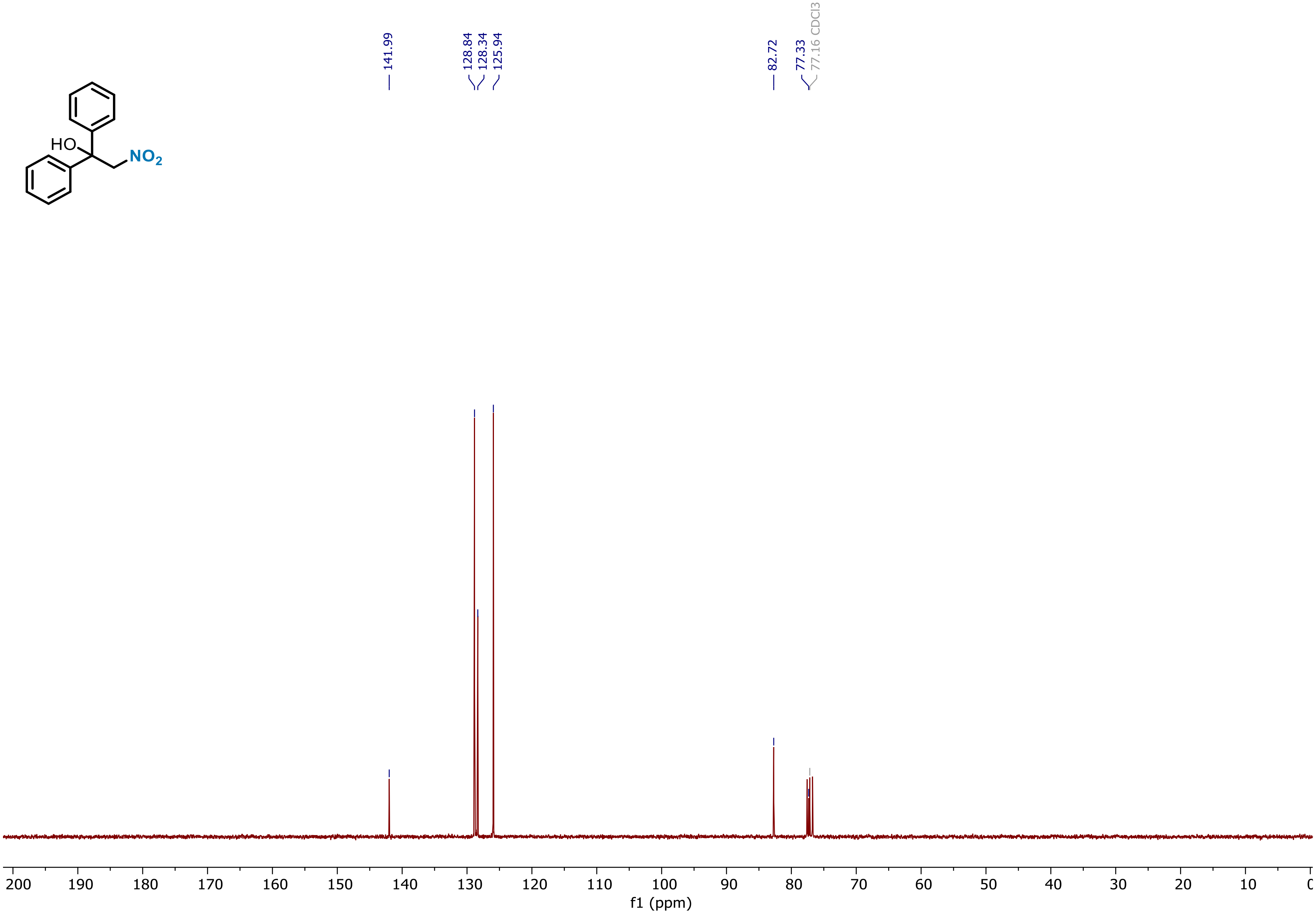

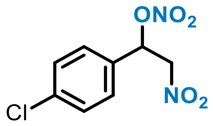

^{1}H NMR (300 MHz, $CDCl_3$) of **32**

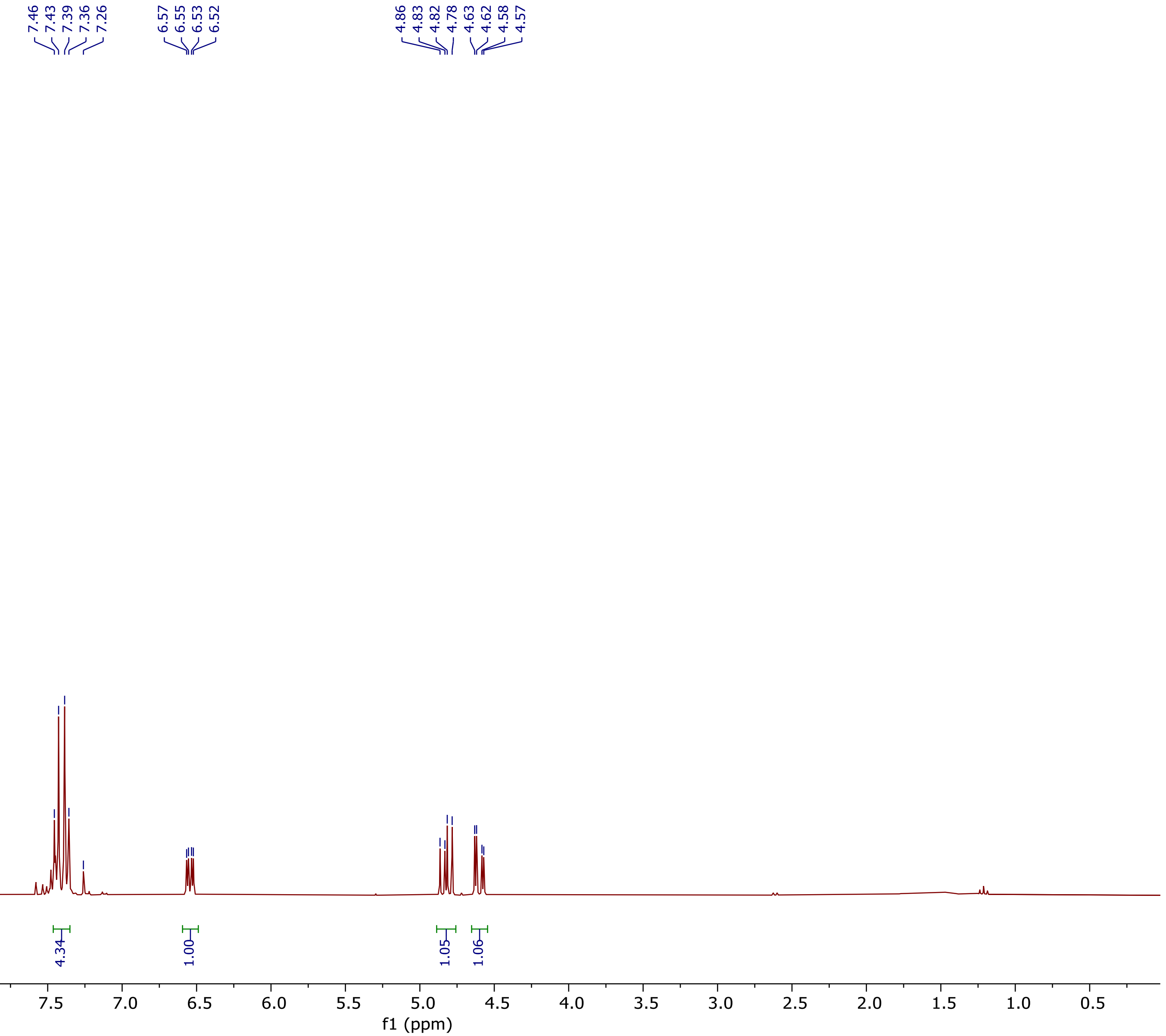

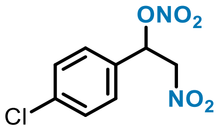

^{13}C NMR (75 MHz, $CDCl_3$) of **32**

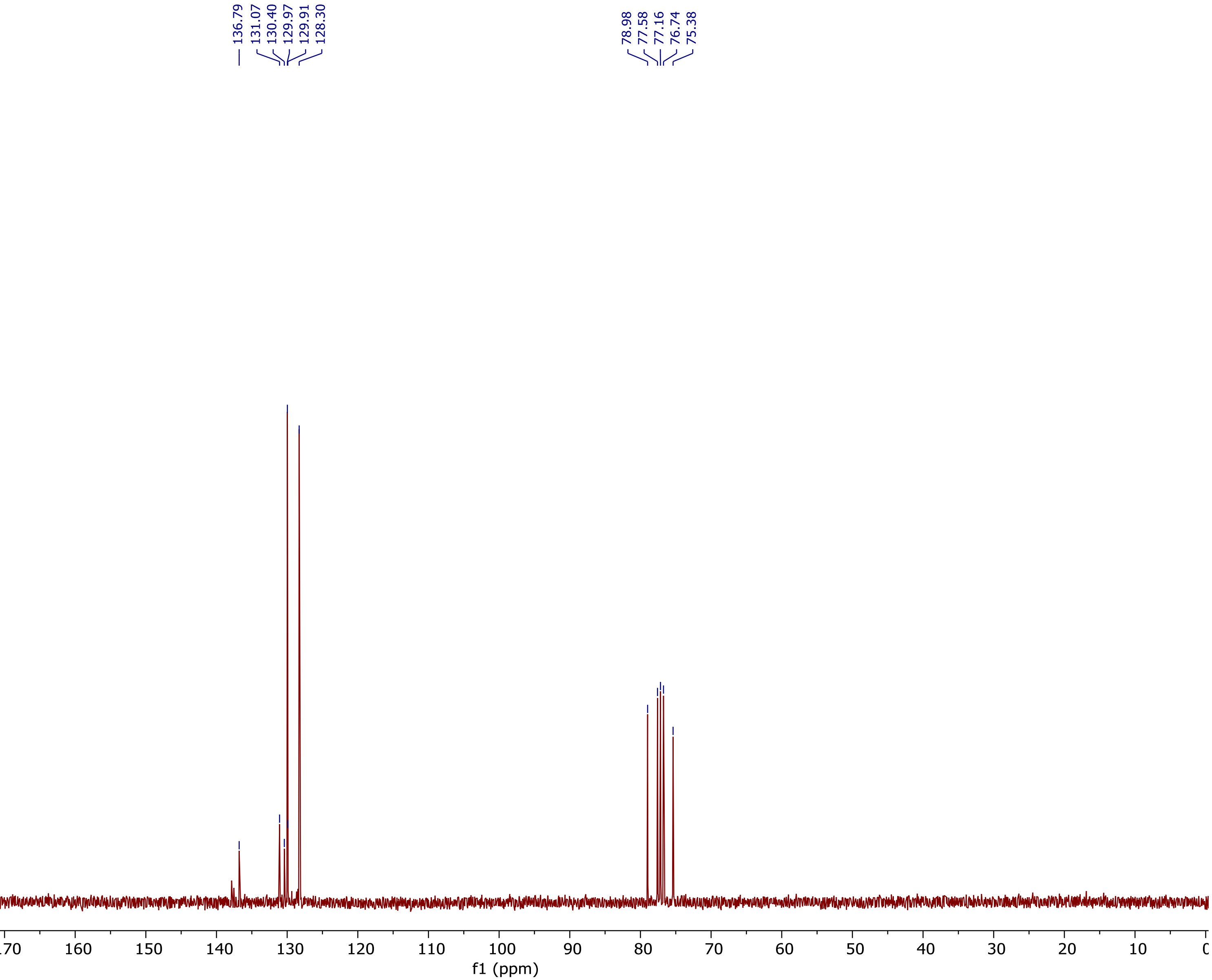

^{1}H NMR (300 MHz, $CDCl_3$) of **32**

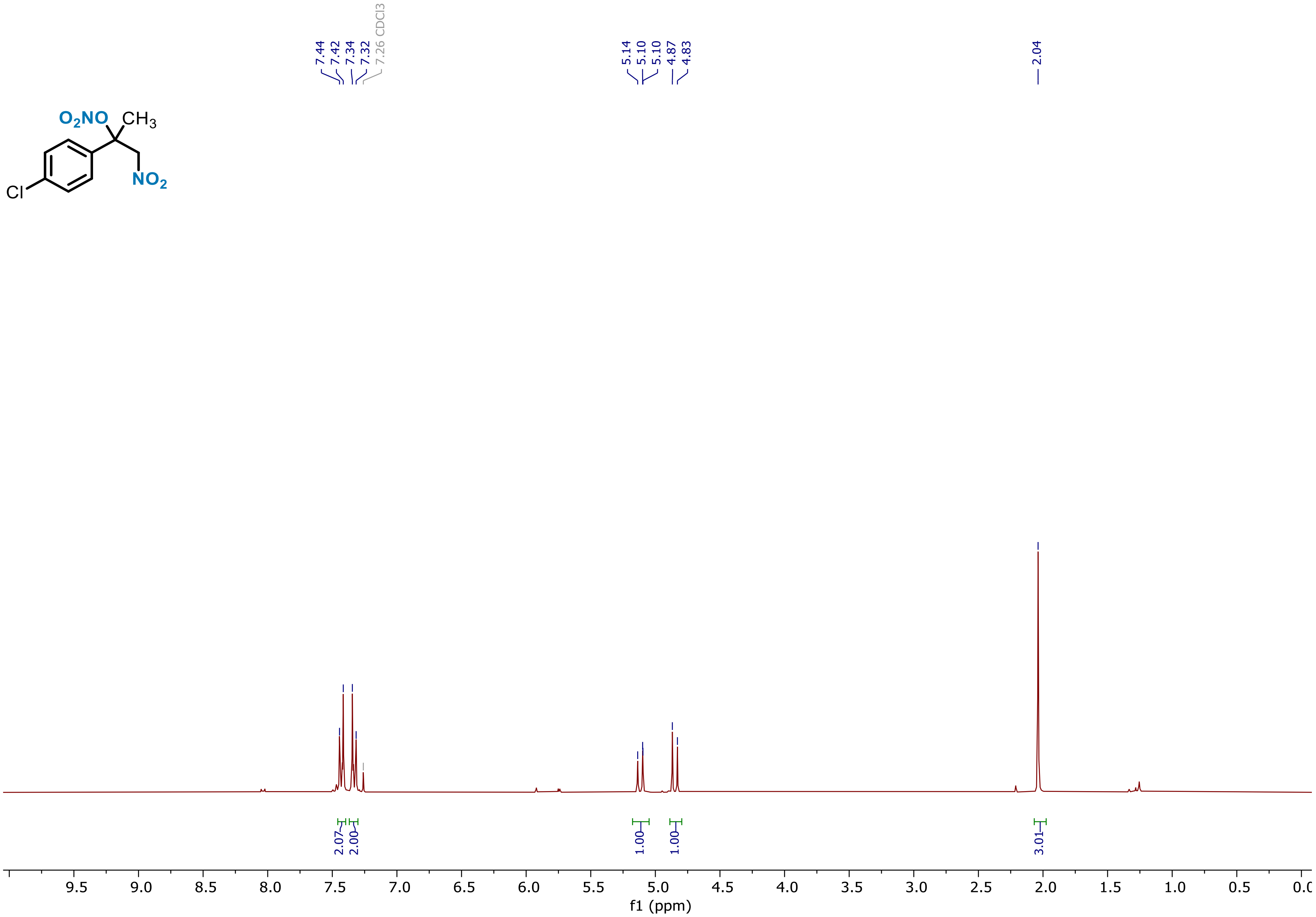

^{13}C NMR (75 MHz, $CDCl_3$) of **32**

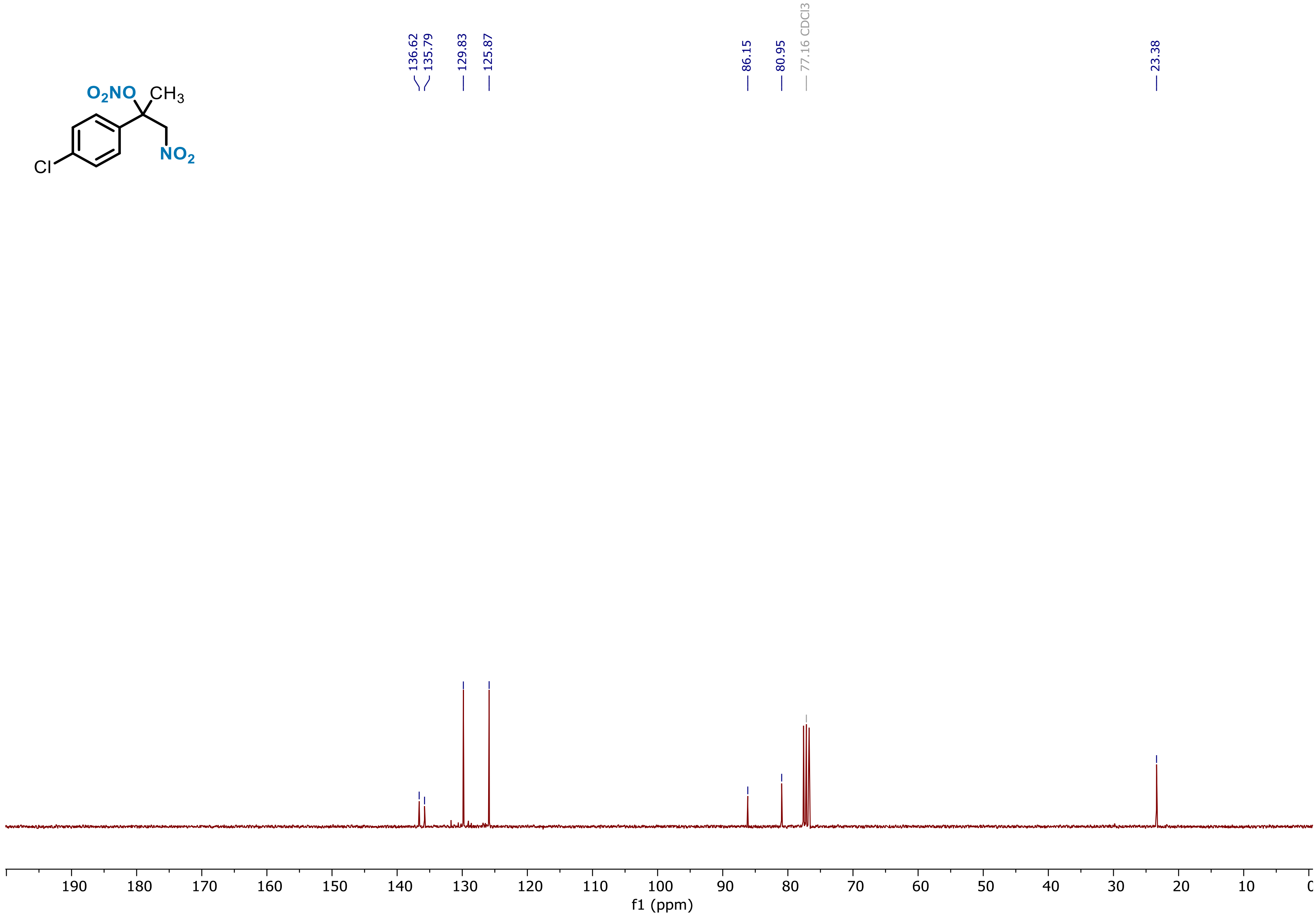

^{1}H NMR (300 MHz, $CDCl_3$) of **33**

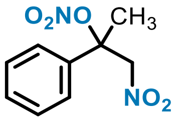

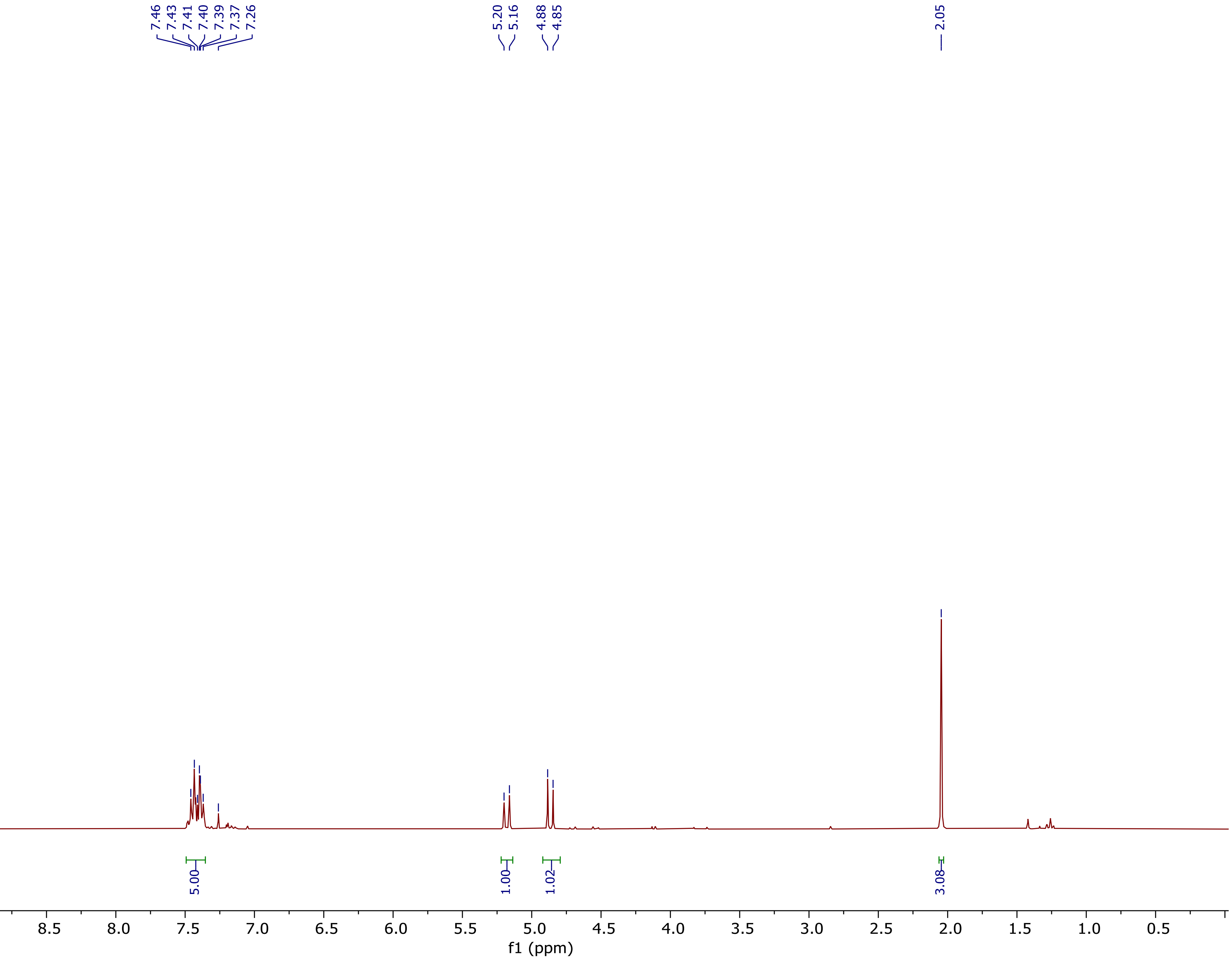

^{13}C NMR (75 MHz, $CDCl_3$) of **33**

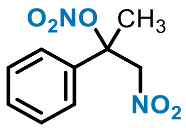

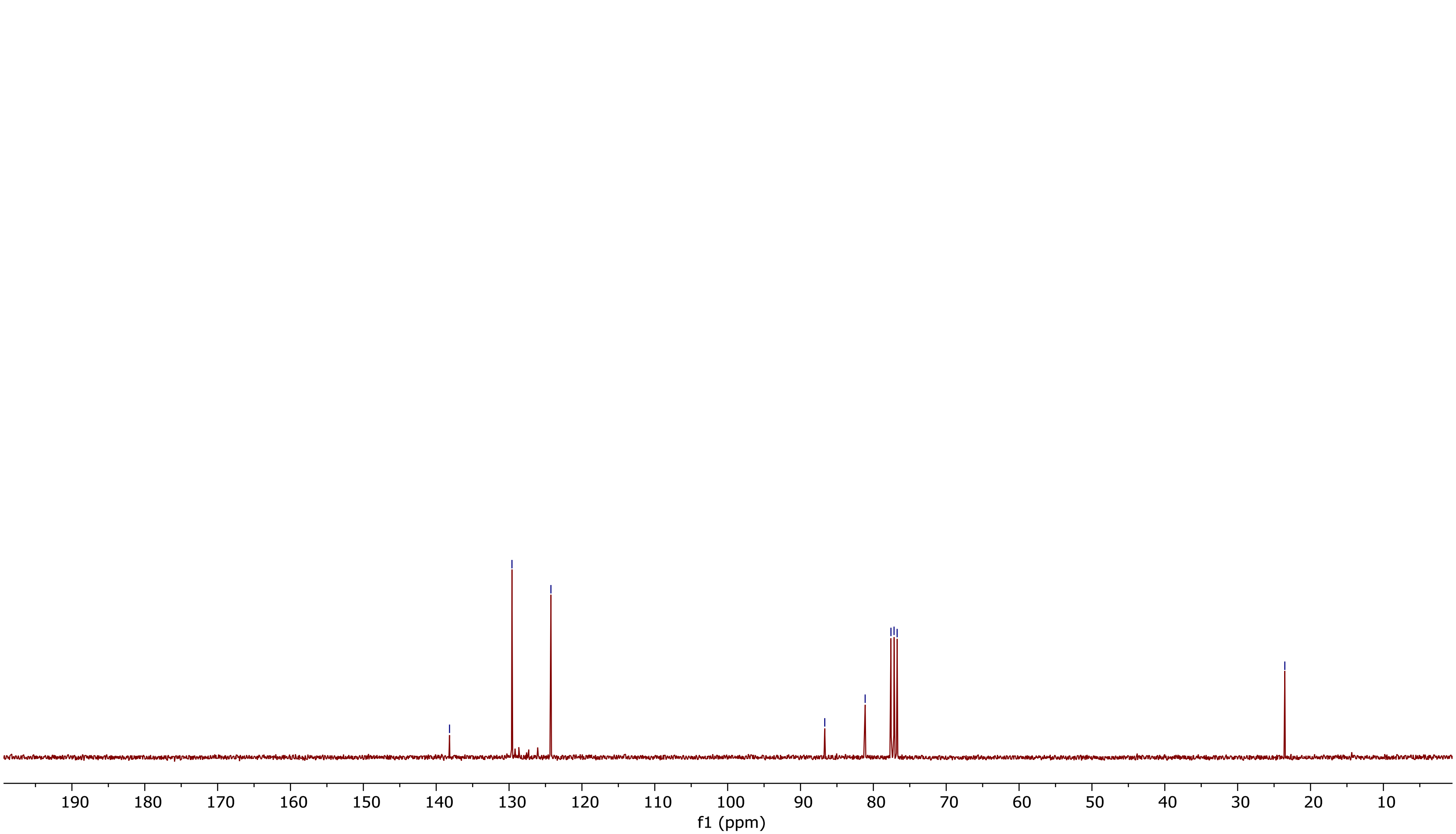

^1H NMR (300 MHz, $CDCl_3$) of **34a**

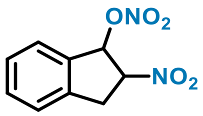

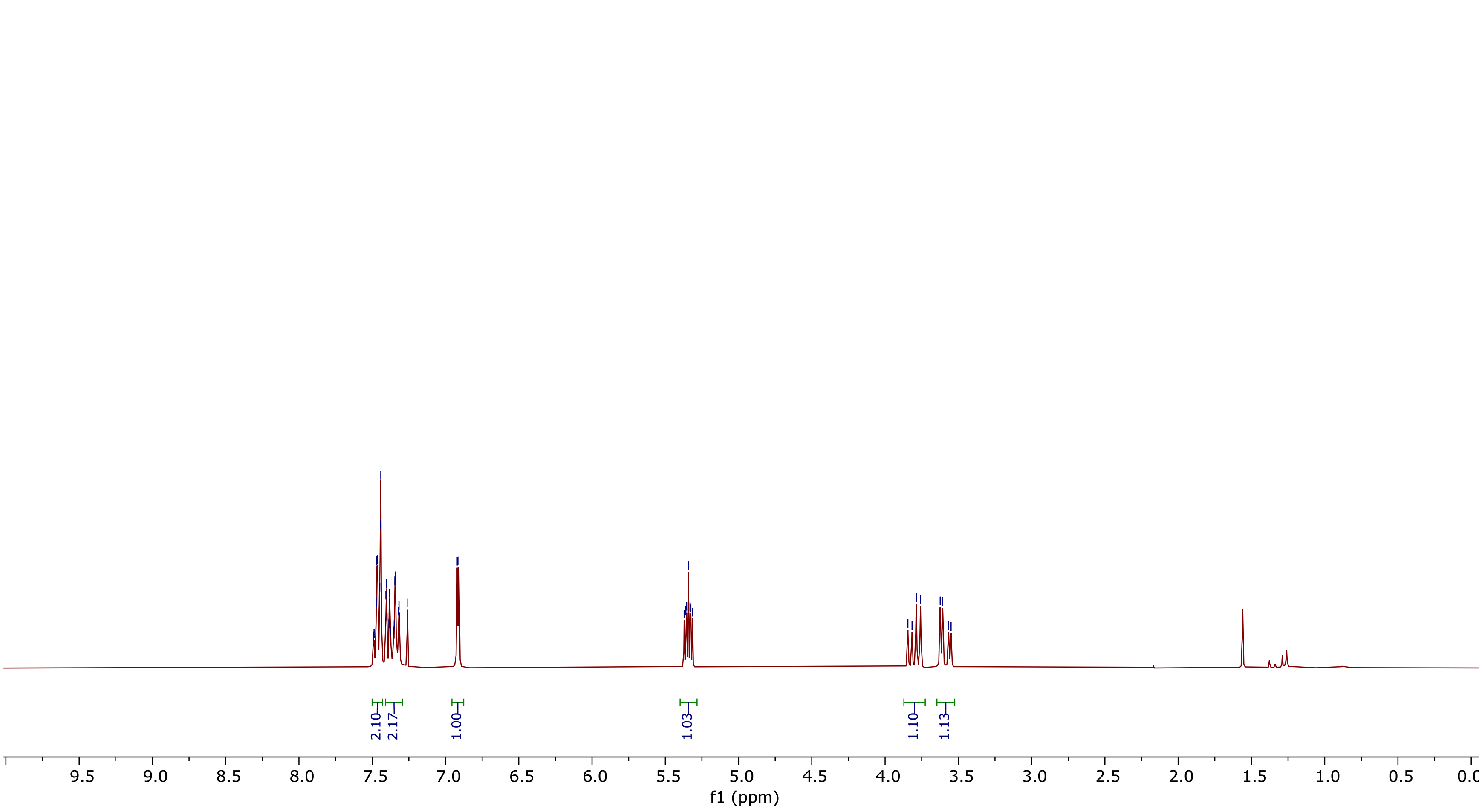

^{13}C NMR (75 MHz, $CDCl_3$) of **34a**

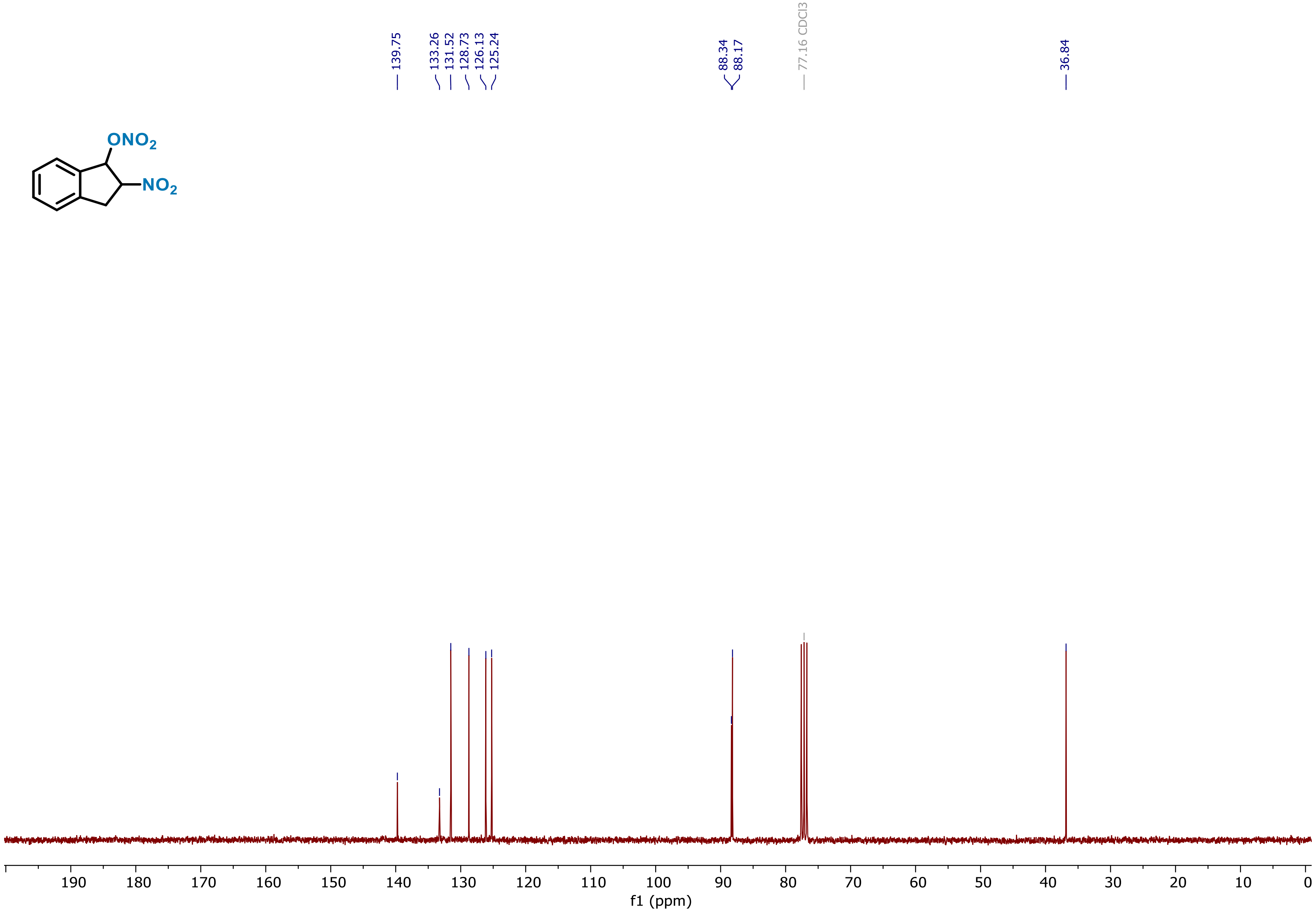

^{1}H NMR (300 MHz, $CDCl_3$) of **34b**

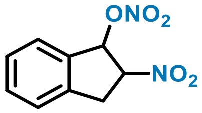

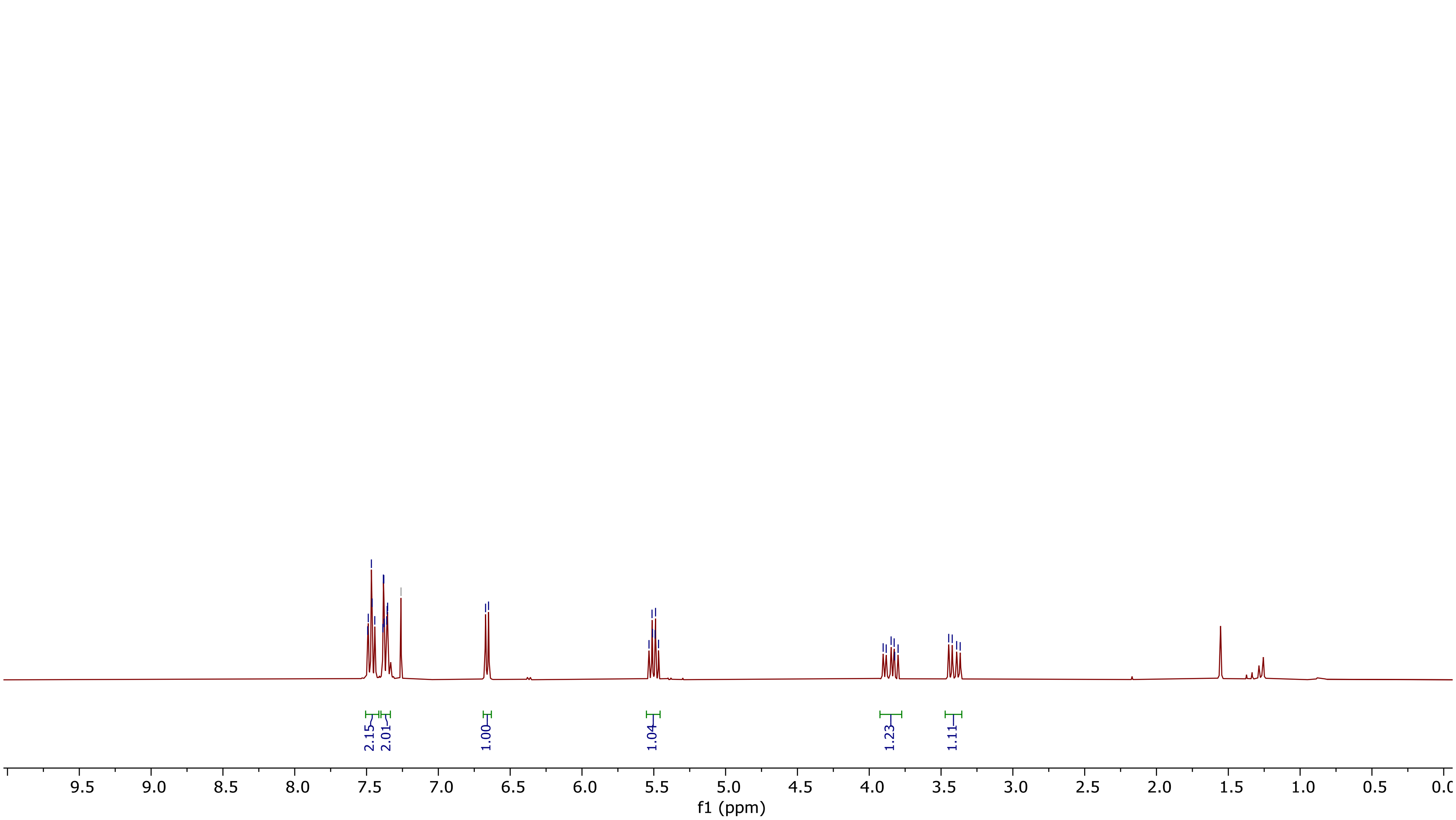

^{13}C NMR (75 MHz, $CDCl_3$) of **34b**

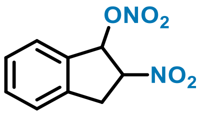
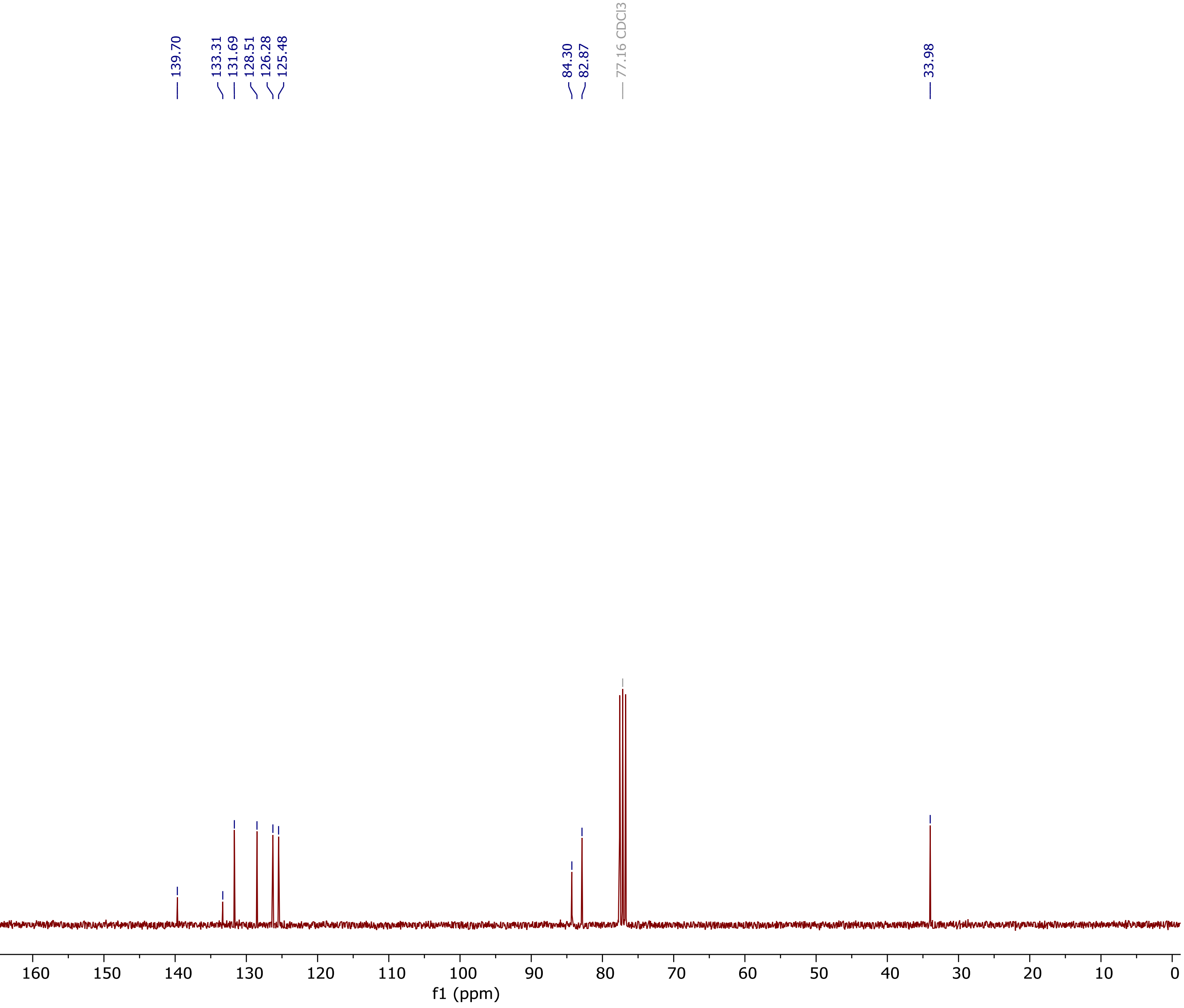

^{1}H NMR (300 MHz, $CDCl_3$) of **35**

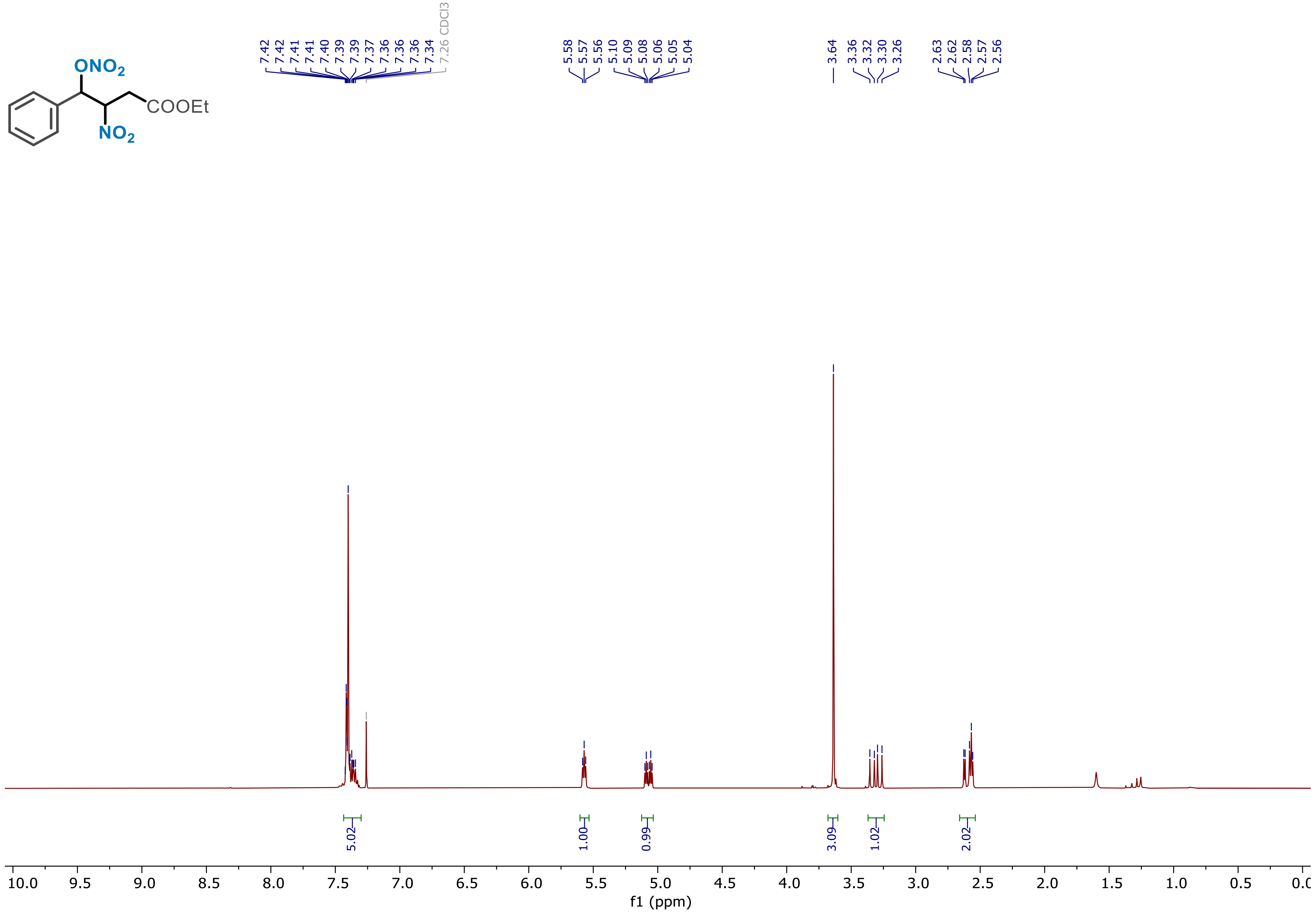

^{13}C NMR (75 MHz, $CDCl_3$) of **35**

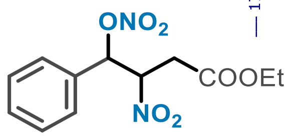

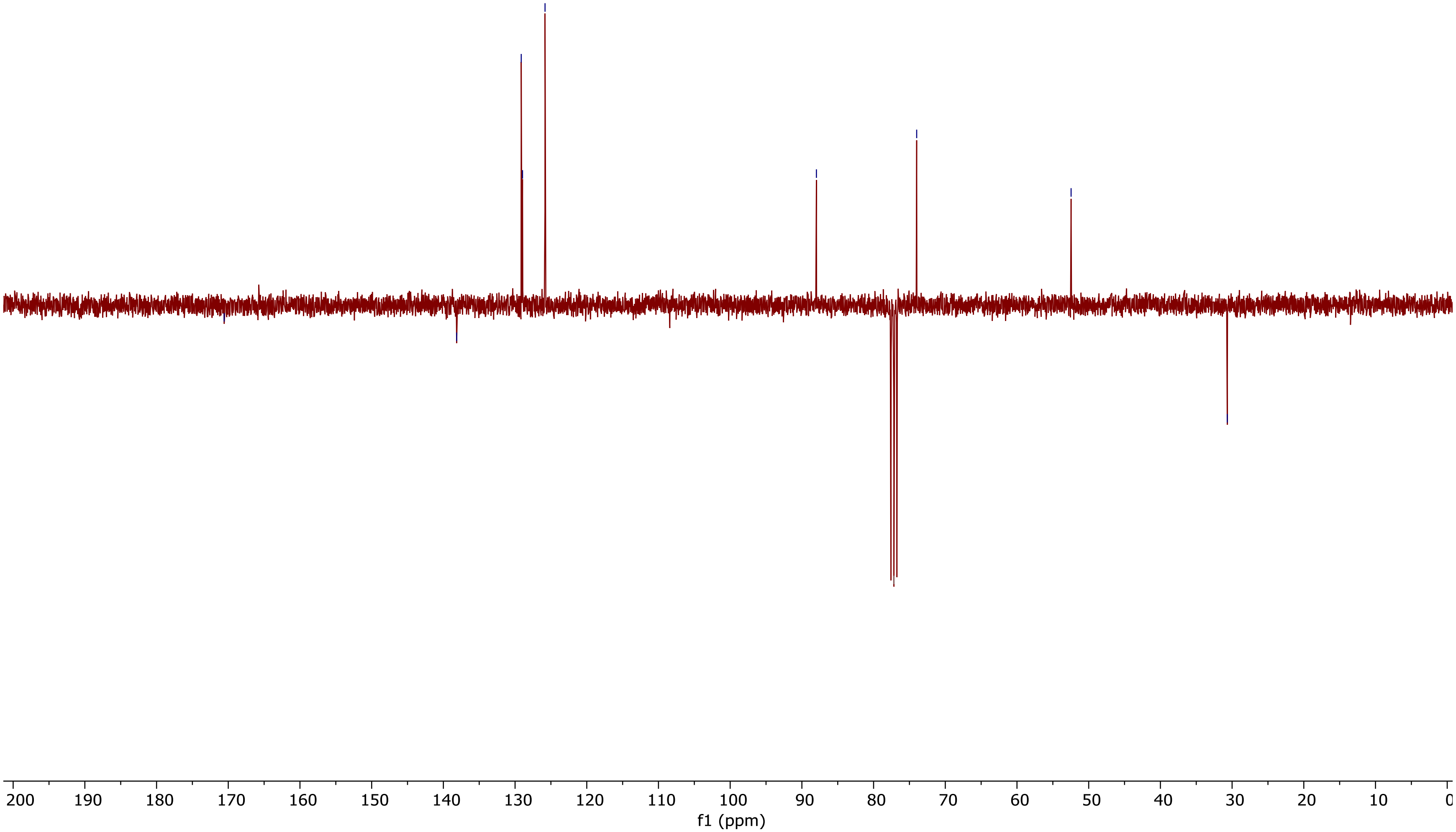

^{1}H NMR (300 MHz, $CDCl_3$) of **36**

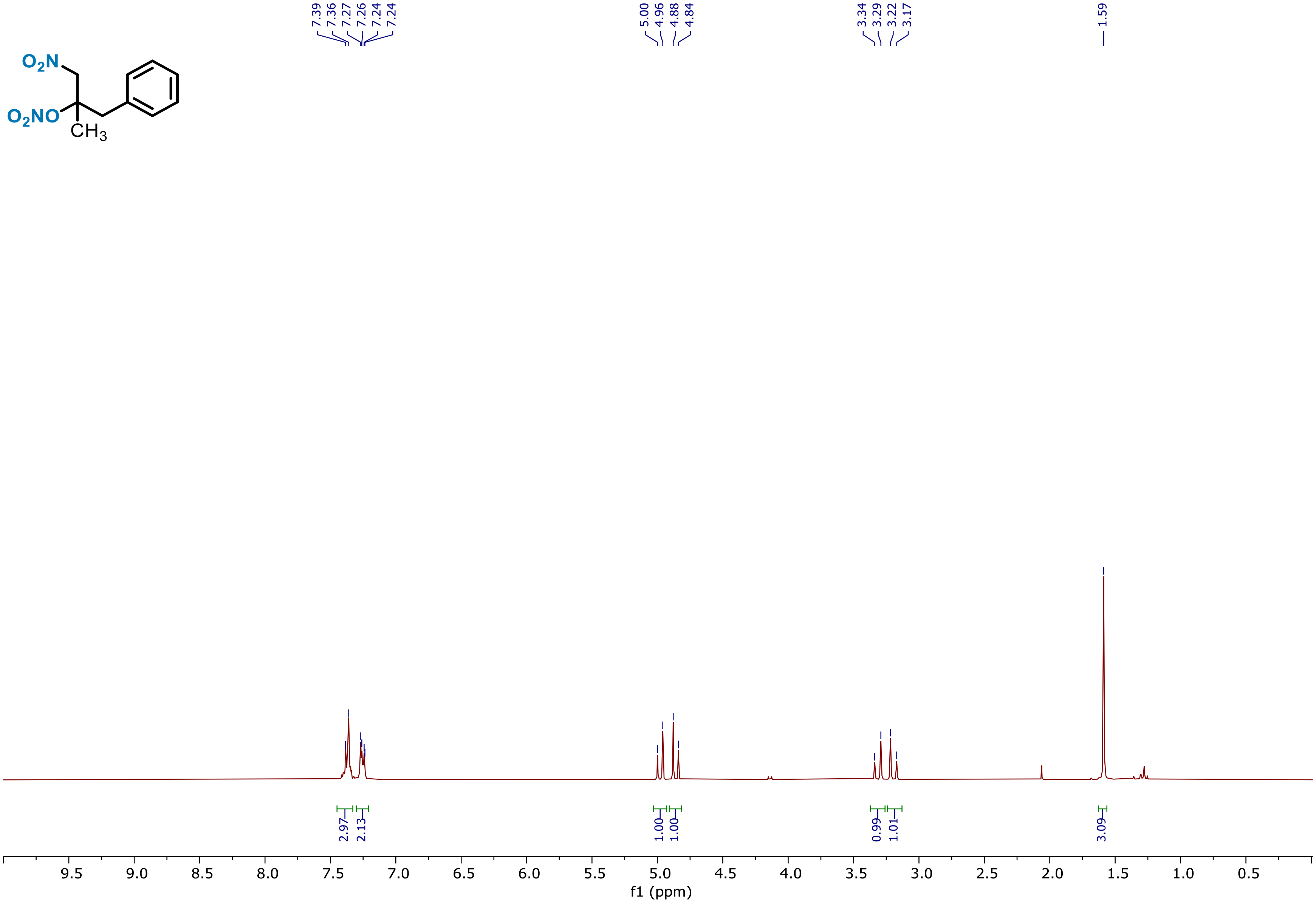

^{13}C NMR (75 MHz, $CDCl_3$) of **36**

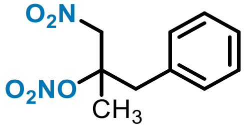

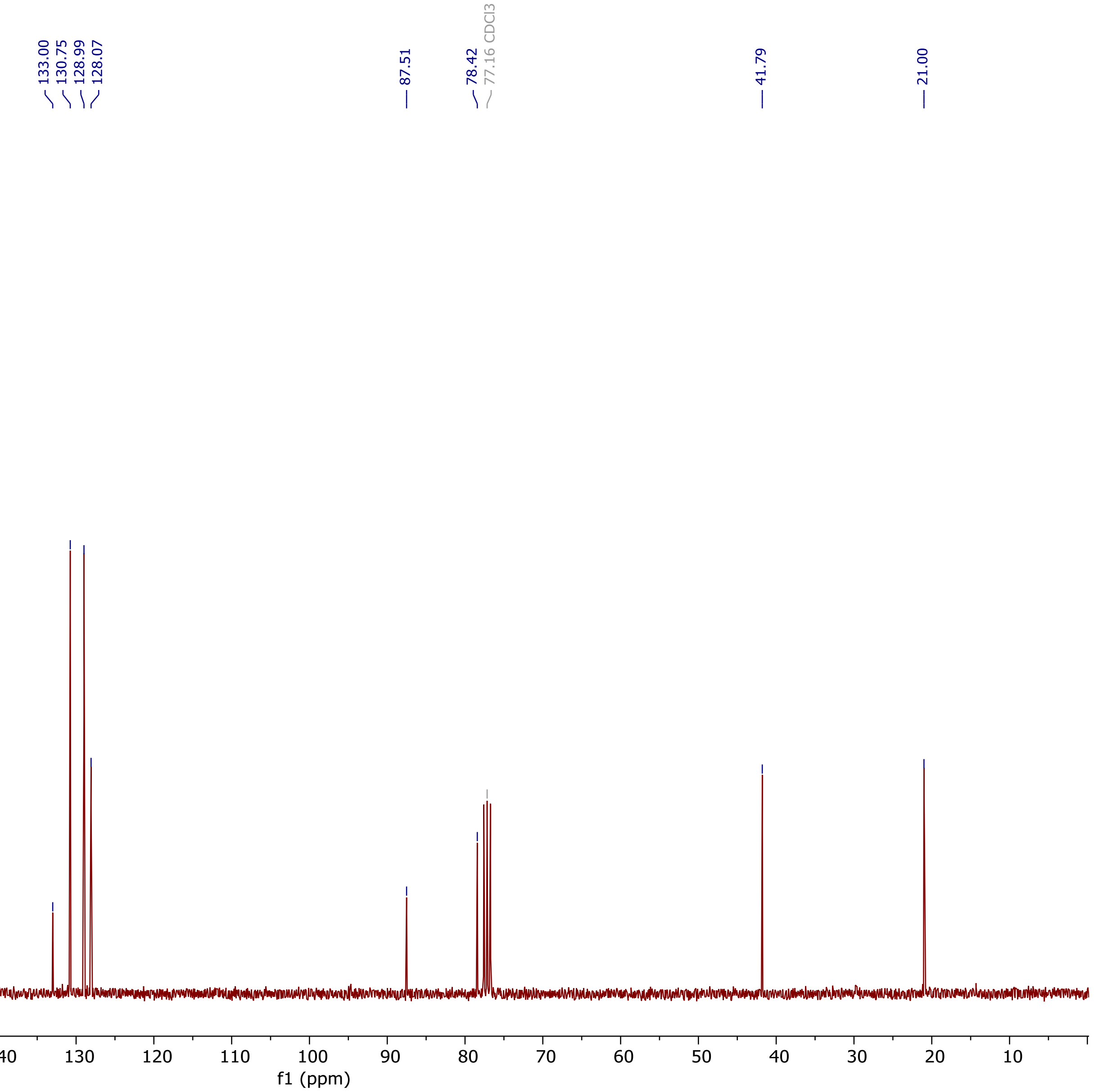

^{1}H NMR (300 MHz, $CDCl_3$) of **37**

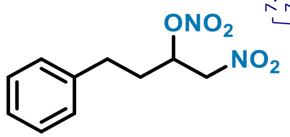

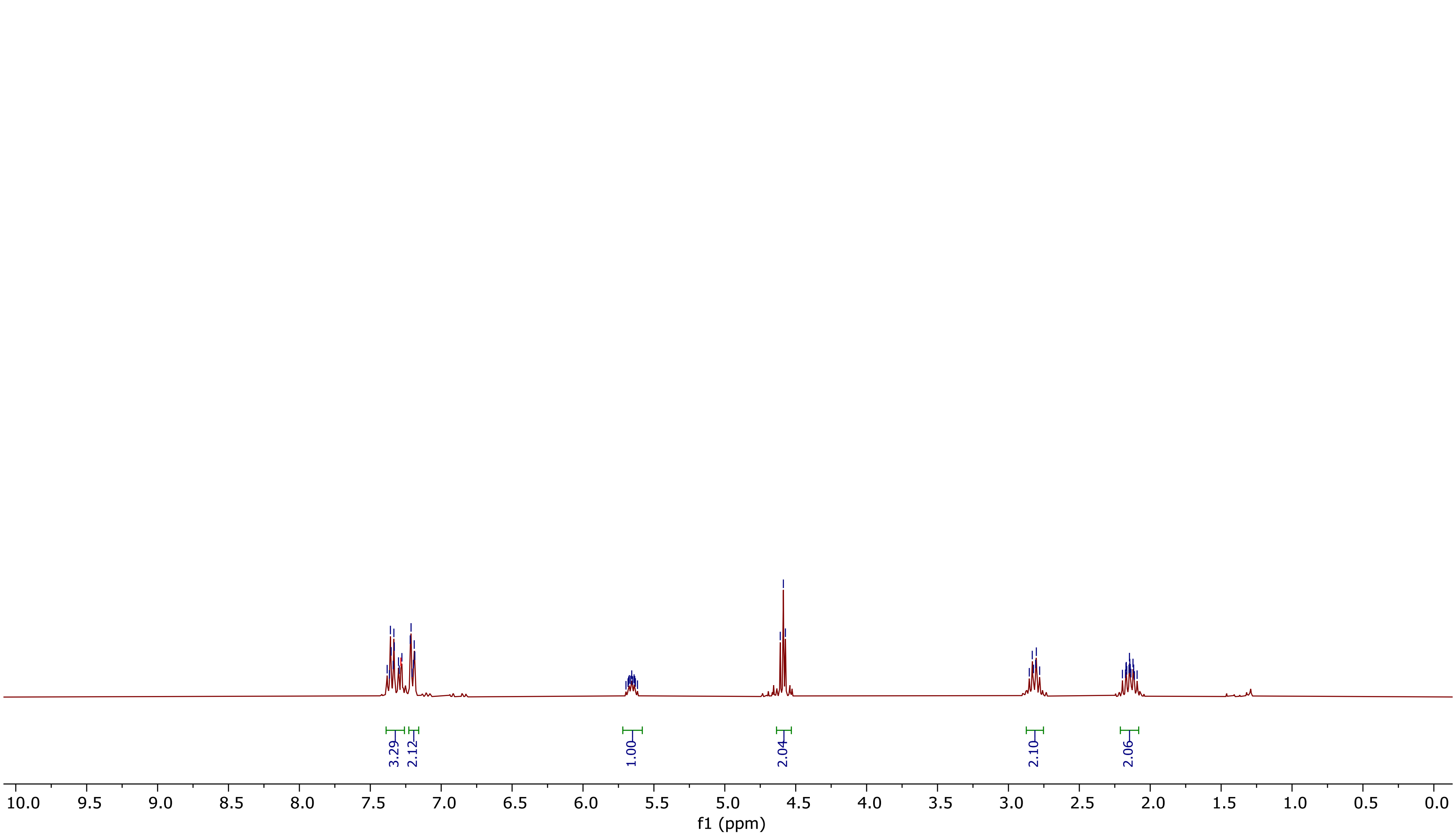

^{13}C NMR (400 MHz, $CDCl_3$) of **37**

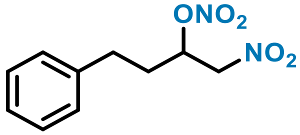

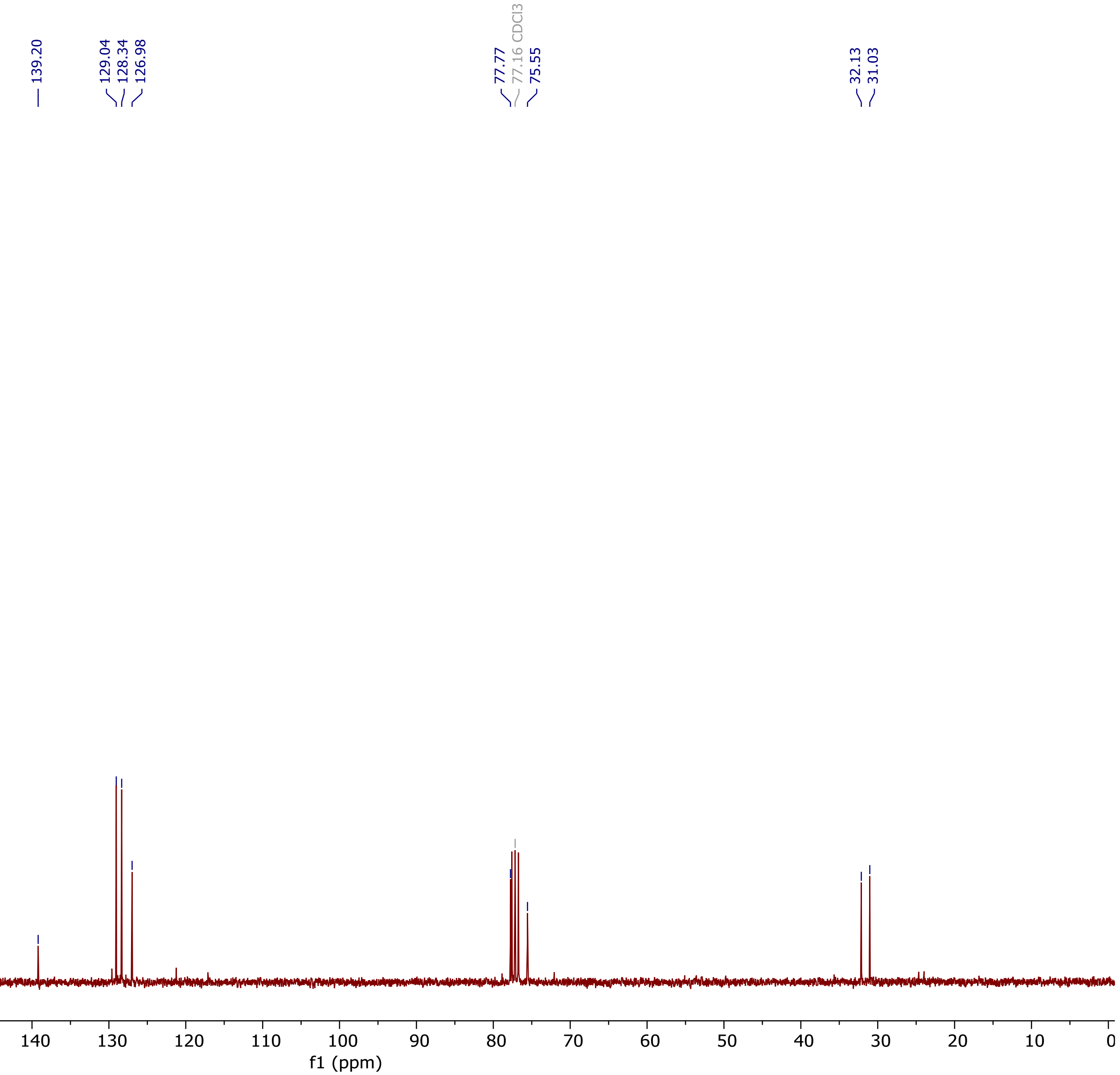

^{1}H NMR (300 MHz, $CDCl_3$) of **38**

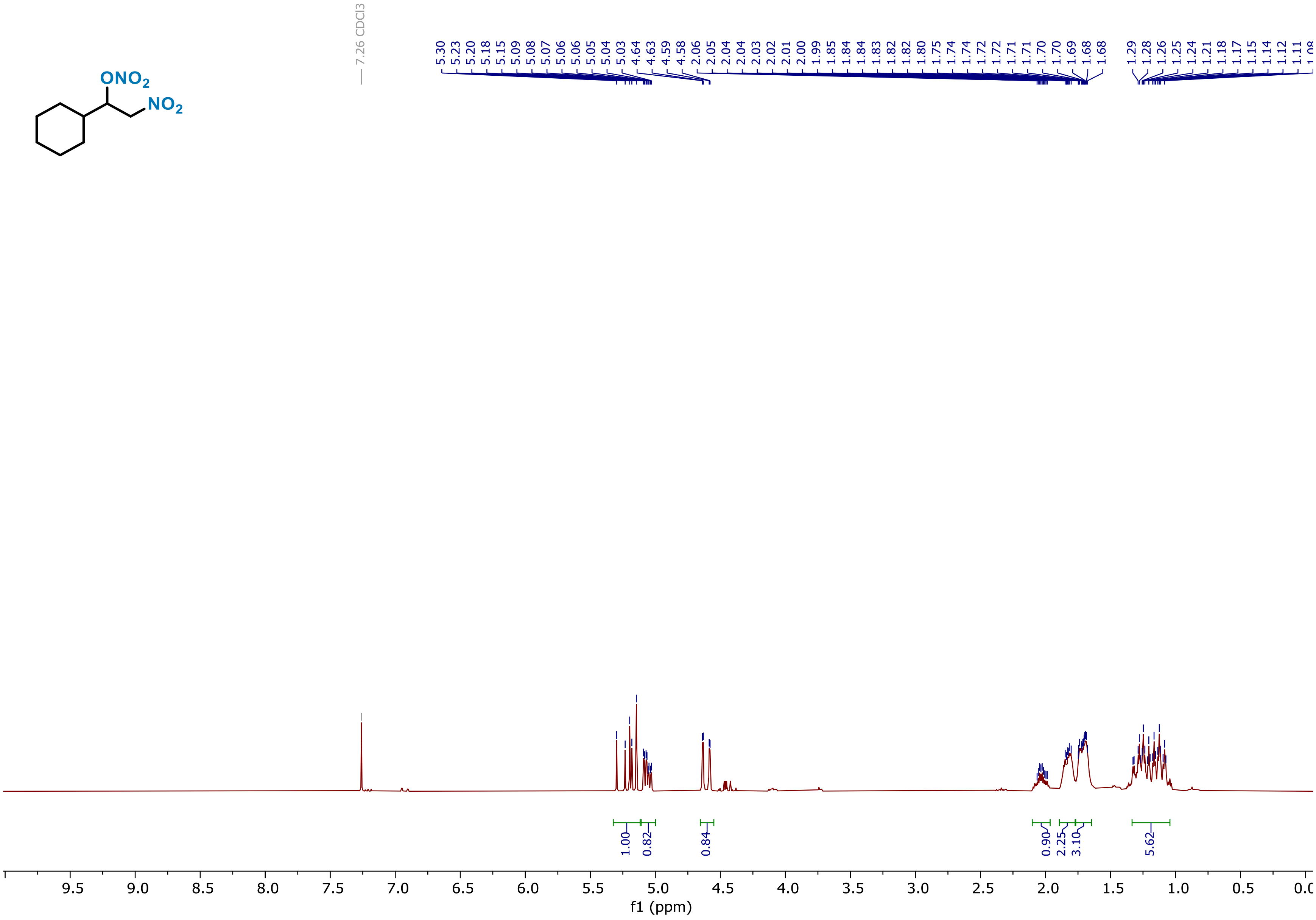

^{13}C NMR (75 MHz, $CDCl_3$) of **38**

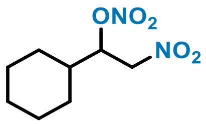

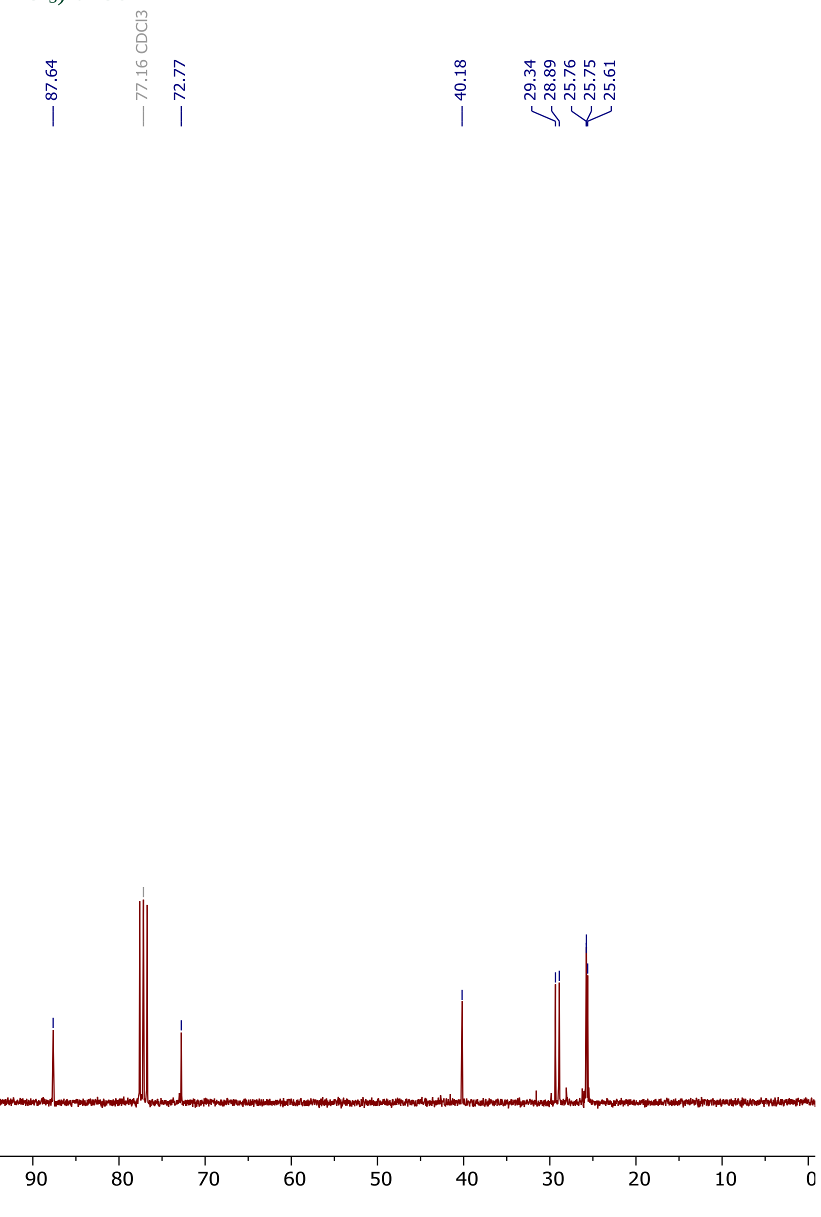

^{1}H NMR (300 MHz, $CDCl_3$) of **39**

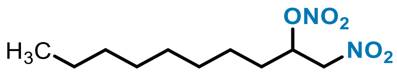

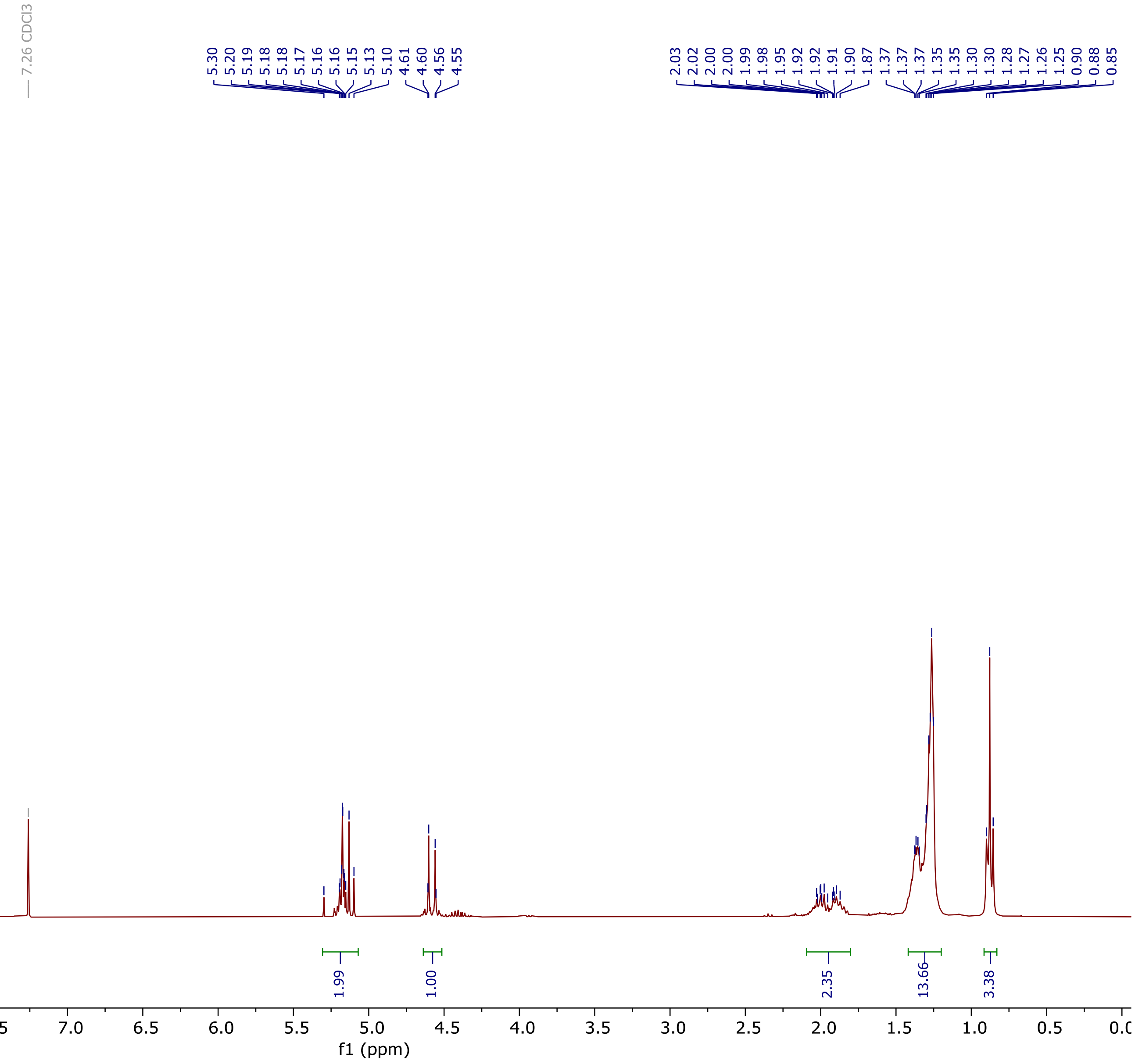

^{13}C NMR (75 MHz, CDCl$_3$) of **39**

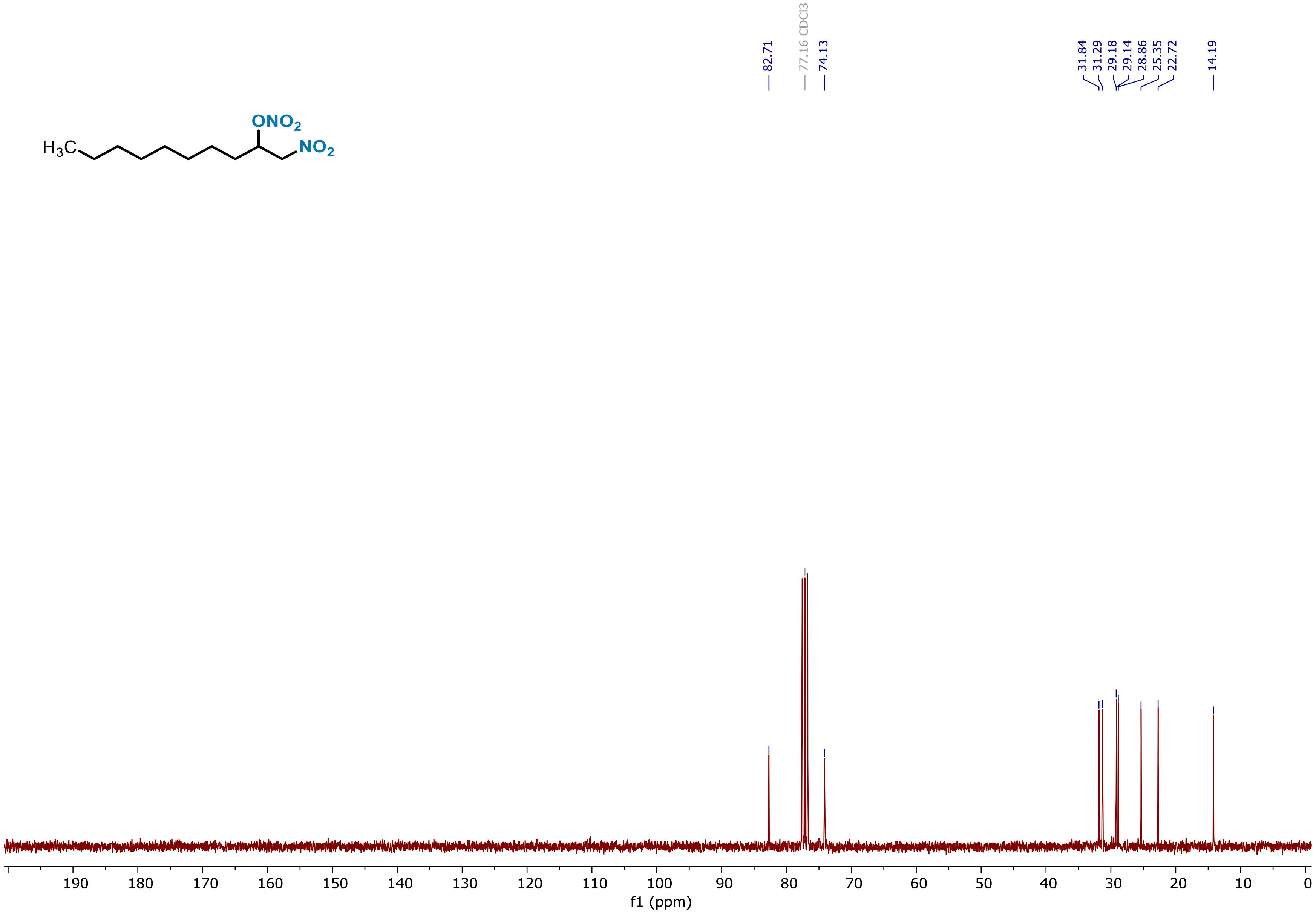

^{1}H NMR (300 MHz, $CDCl_3$) of **40**

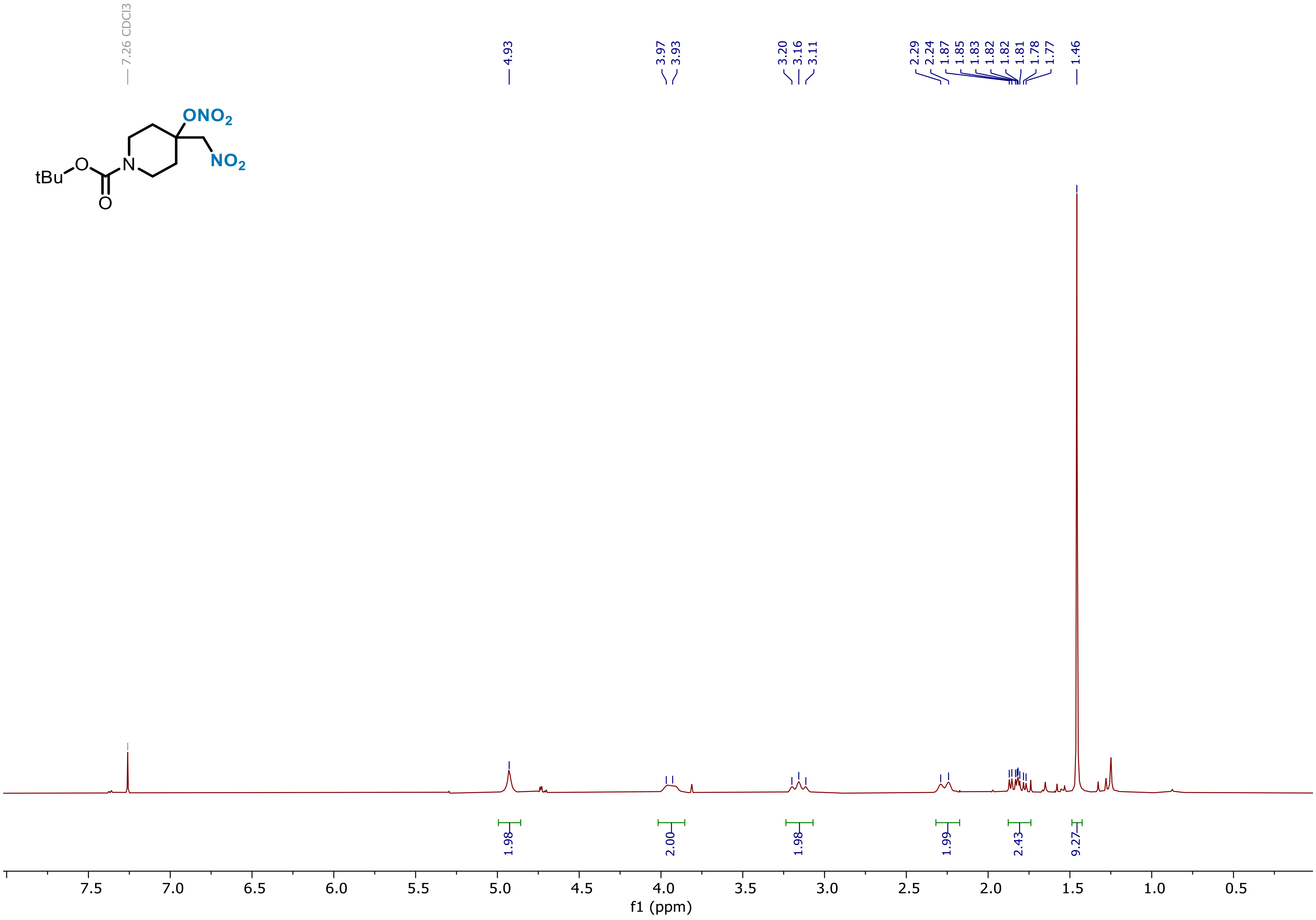

^{13}C NMR (75 MHz, $CDCl_3$) of **40**

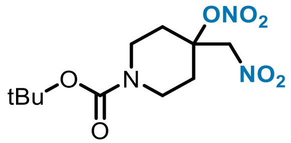

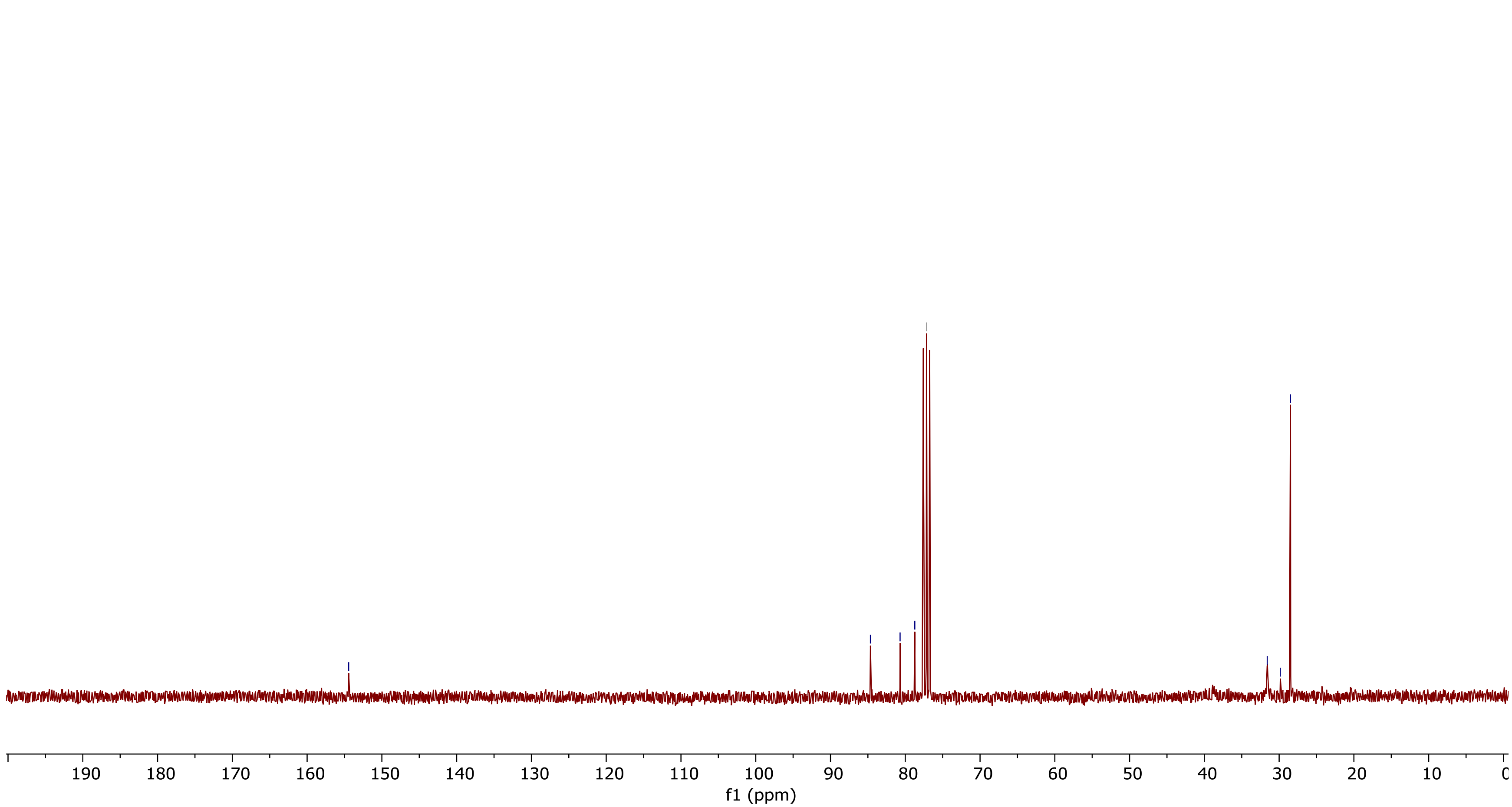

^{1}H NMR (300 MHz, $CDCl_3$) of **40**

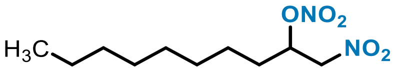

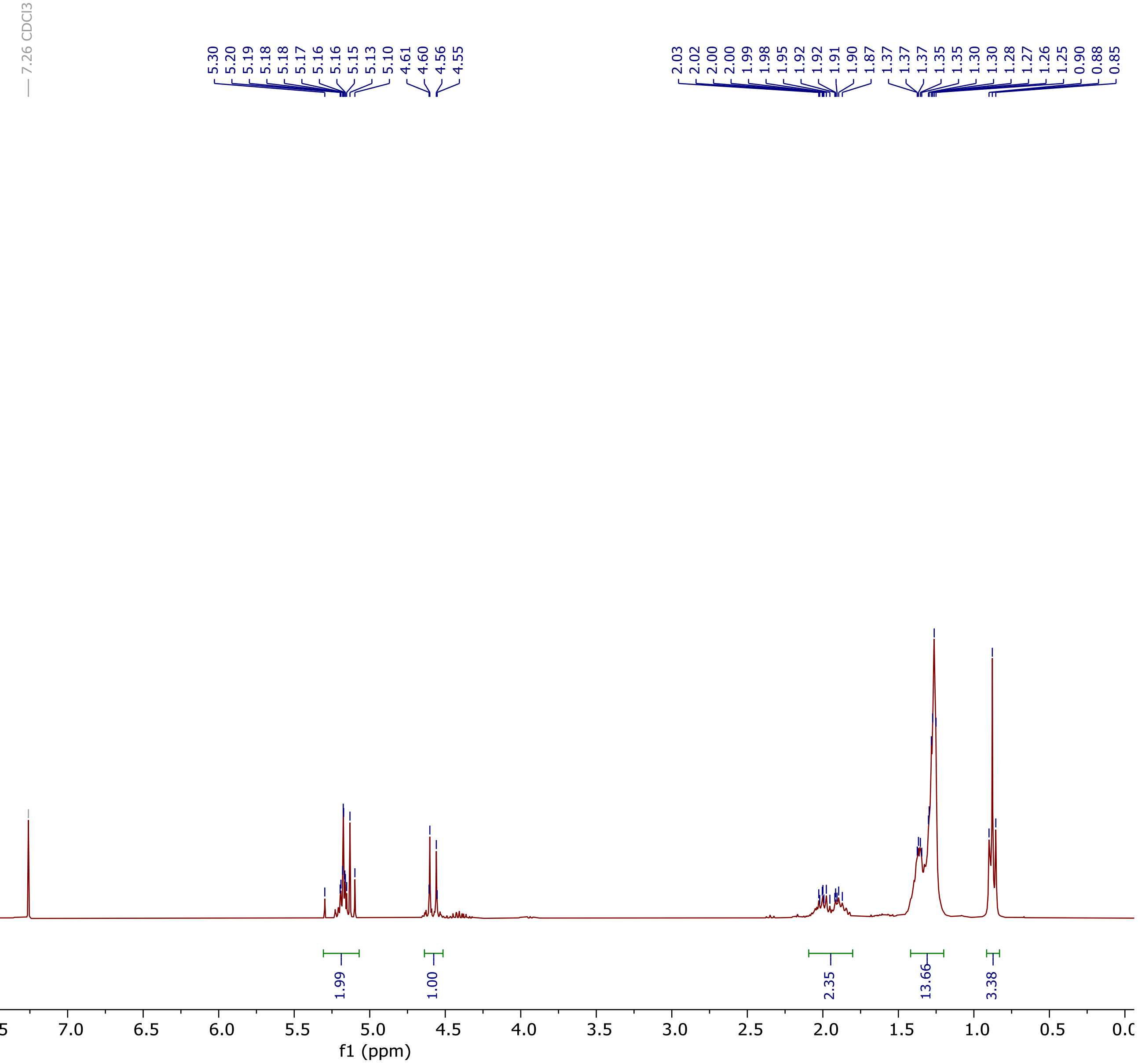

^{13}C NMR (75 MHz, $CDCl_3$) of **40**

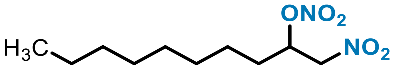

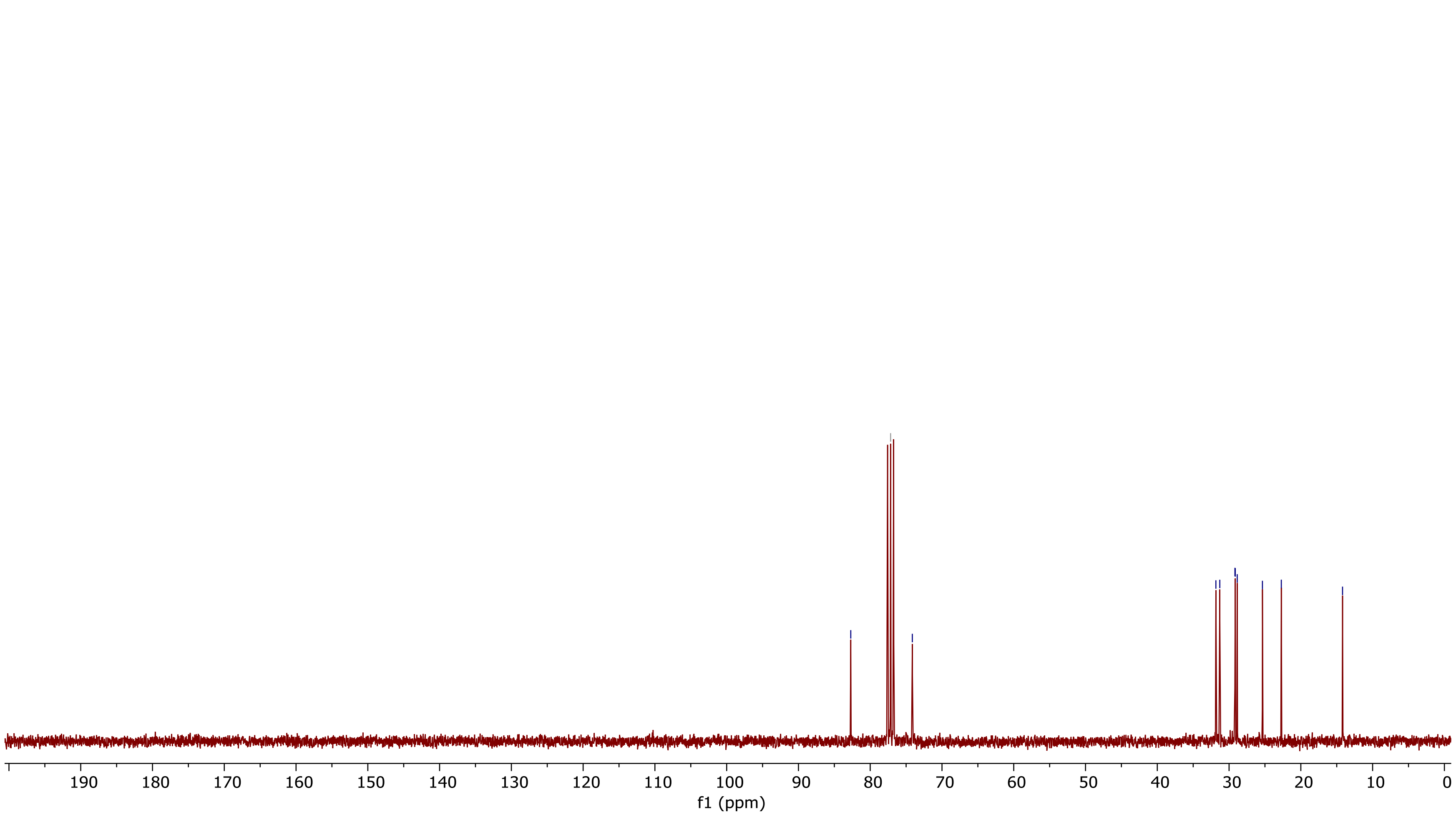

^{1}H NMR (300 MHz, $CDCl_3$) of **7**

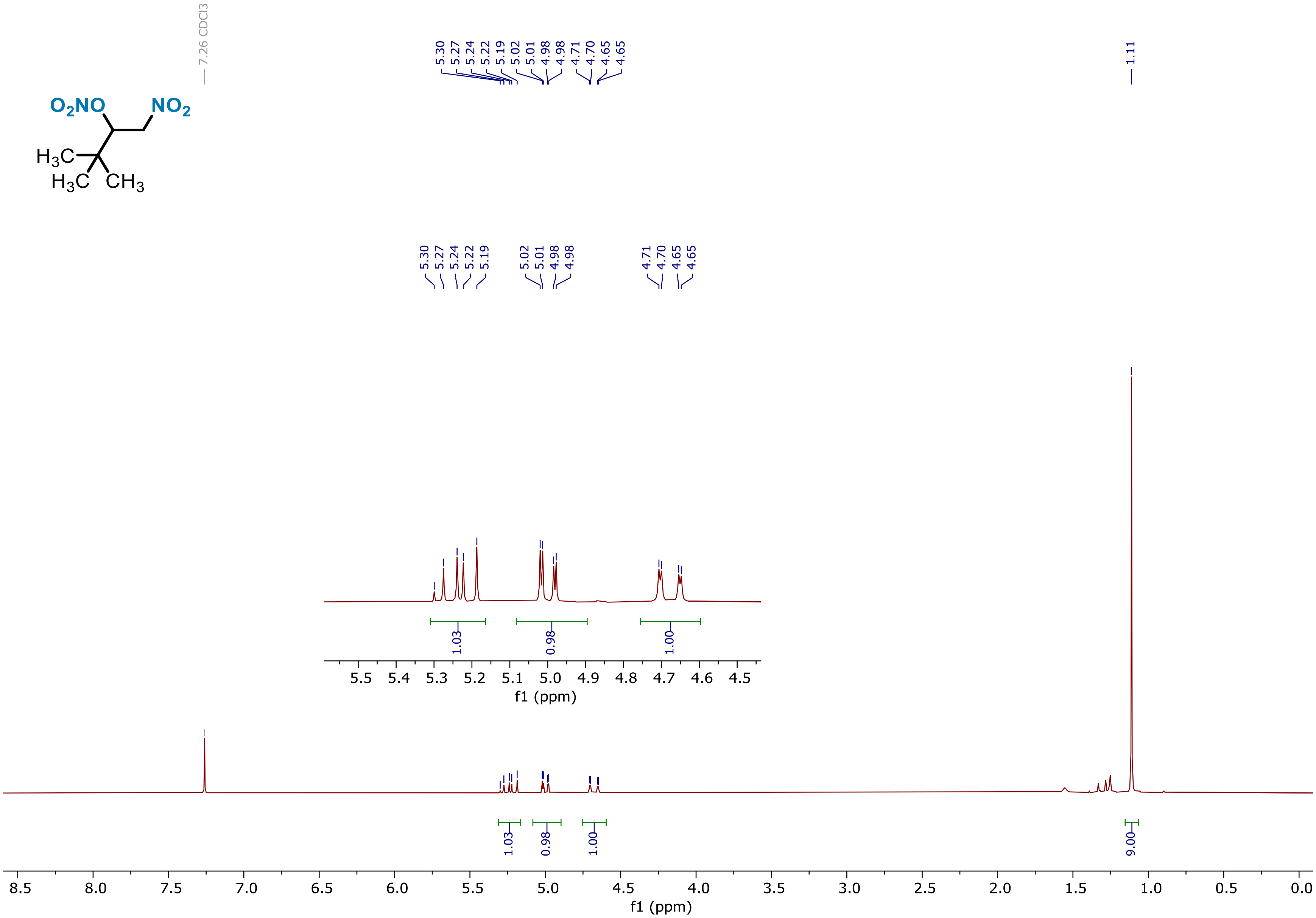

^{13}C NMR (75 MHz, $CDCl_3$) of **7**

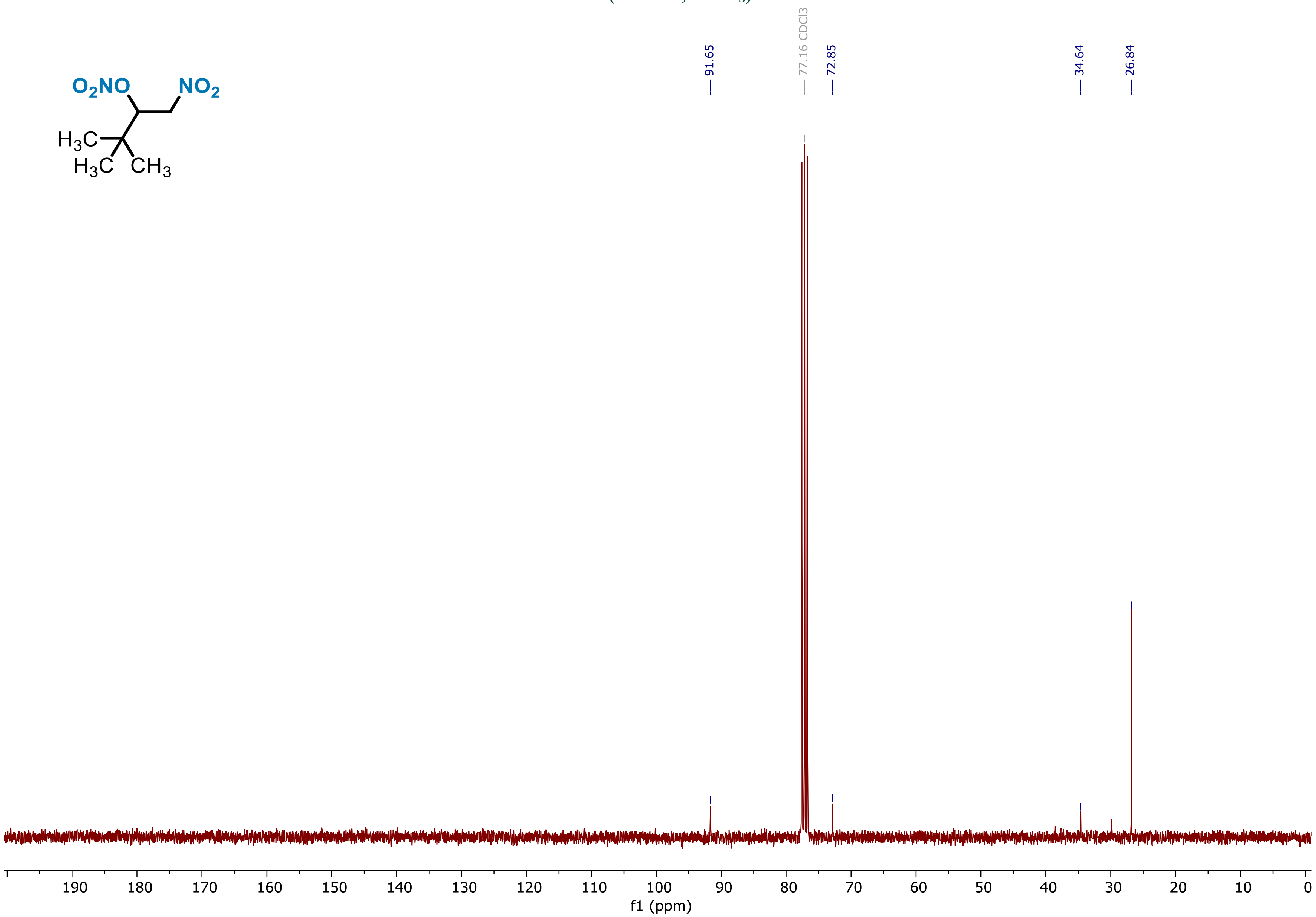

^{1}H NMR (300 MHz, $CDCl_3$) of **41**

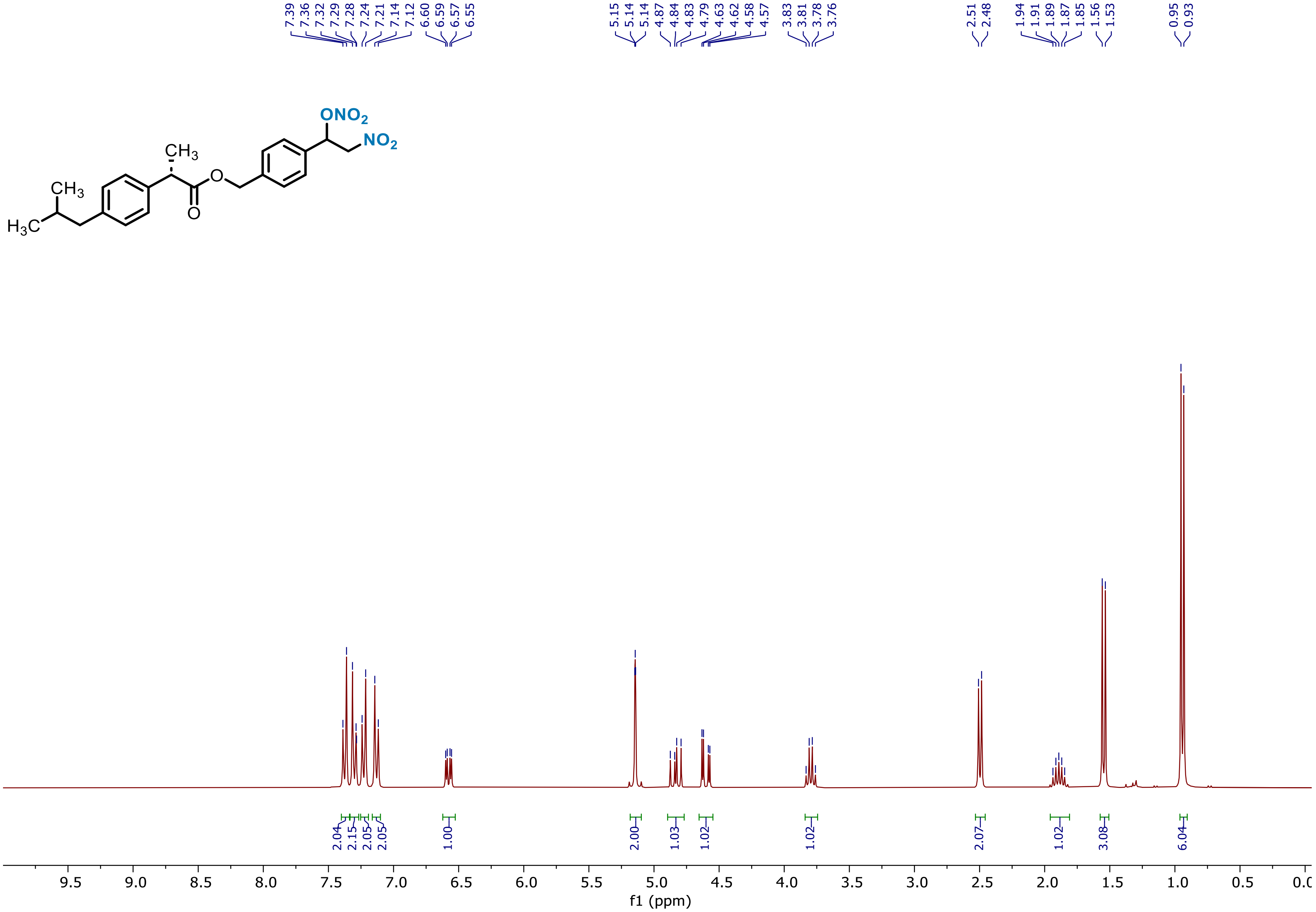

^{13}C NMR (75 MHz, $CDCl_3$) of **41**

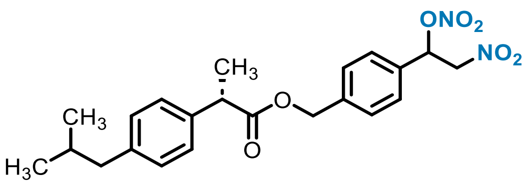

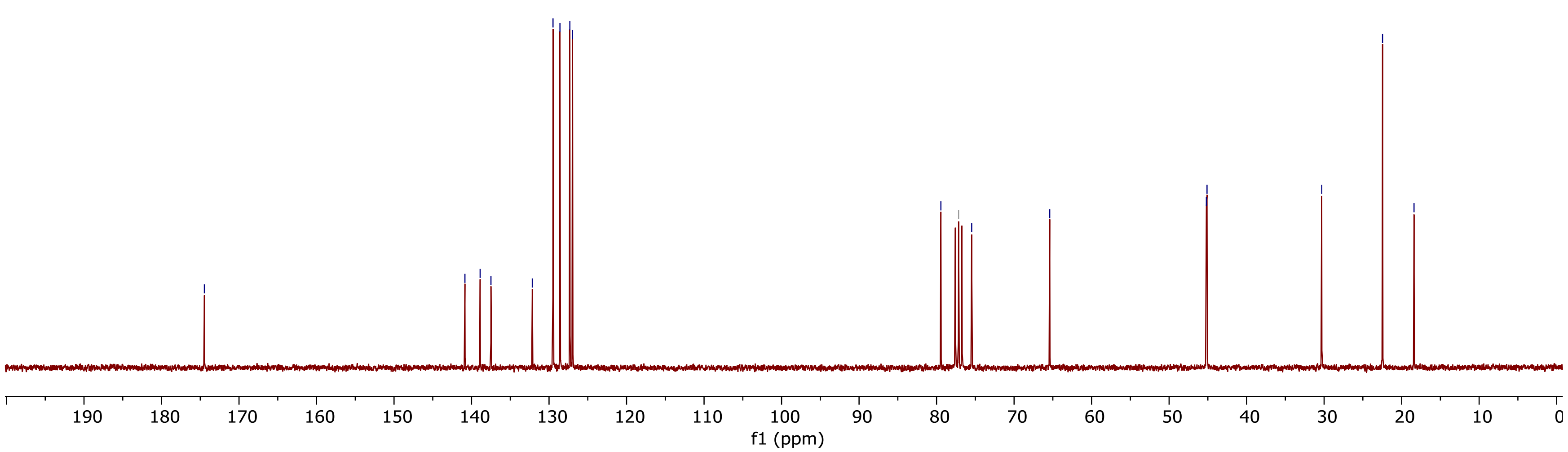

^{1}H NMR (300 MHz, $CDCl_3$) of **42**

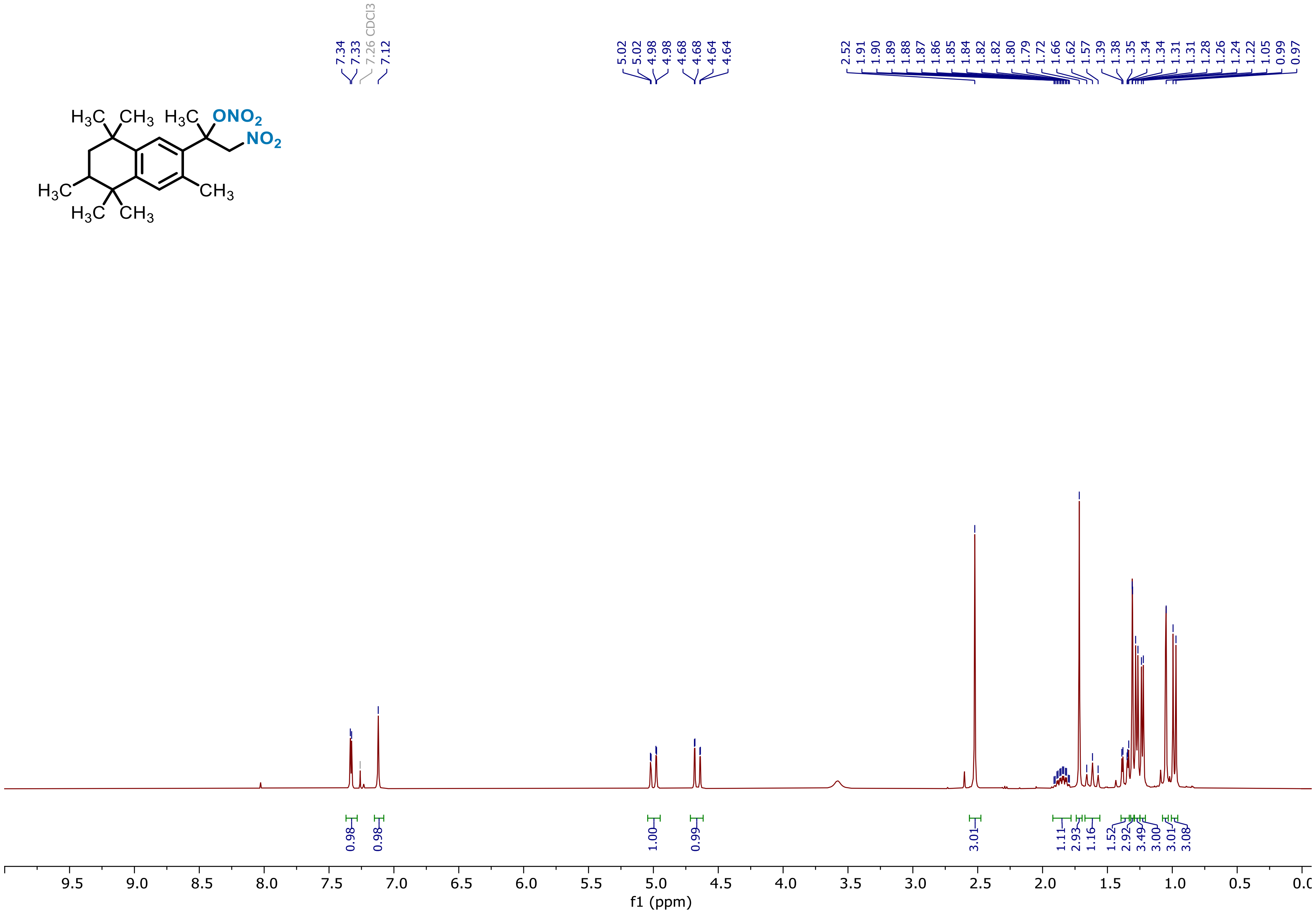

^{13}C NMR (75 MHz, $CDCl_3$) of **42**

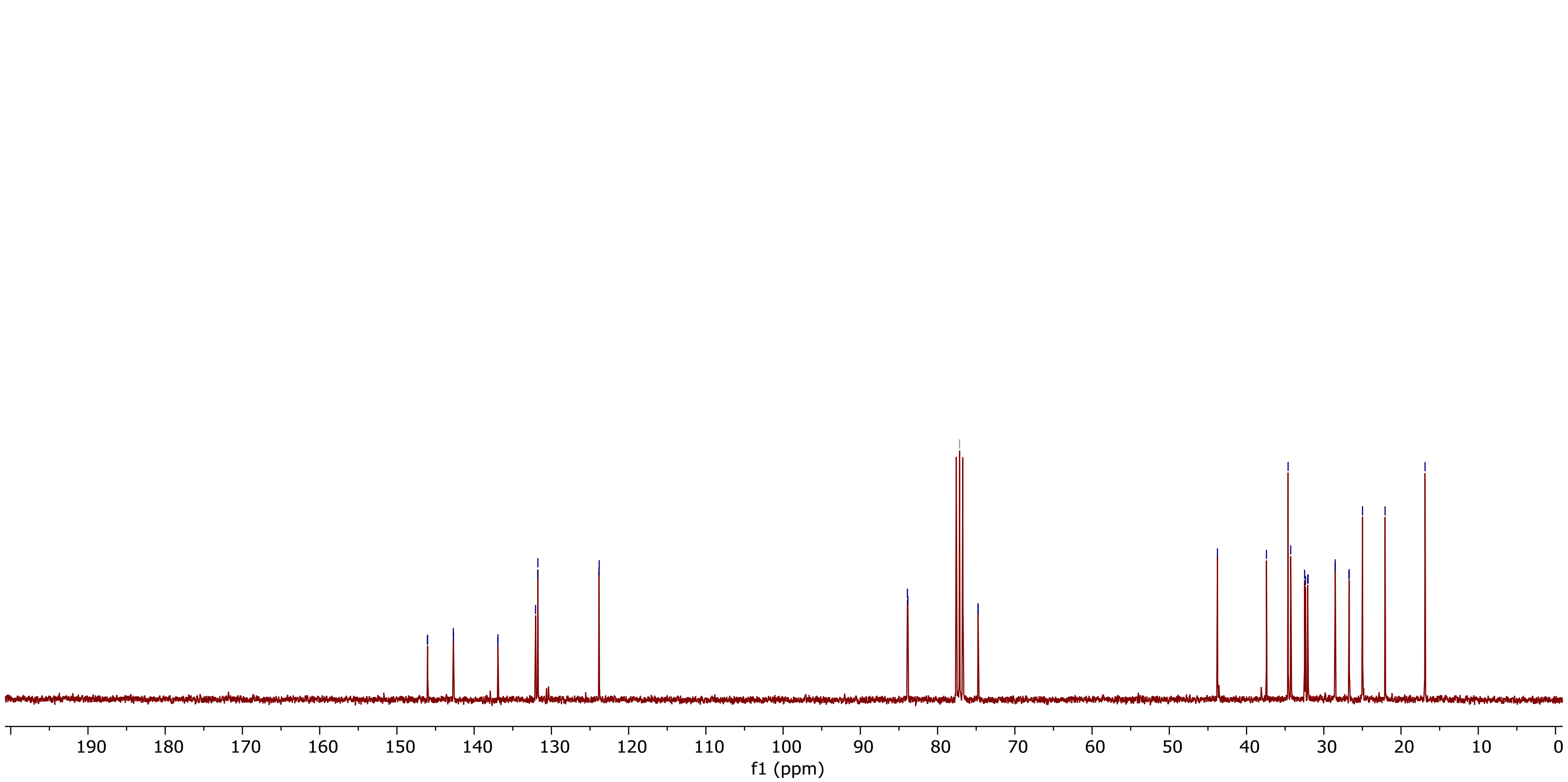

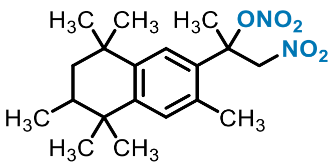

^{1}H NMR (300 MHz, $CDCl_3$) of **44**

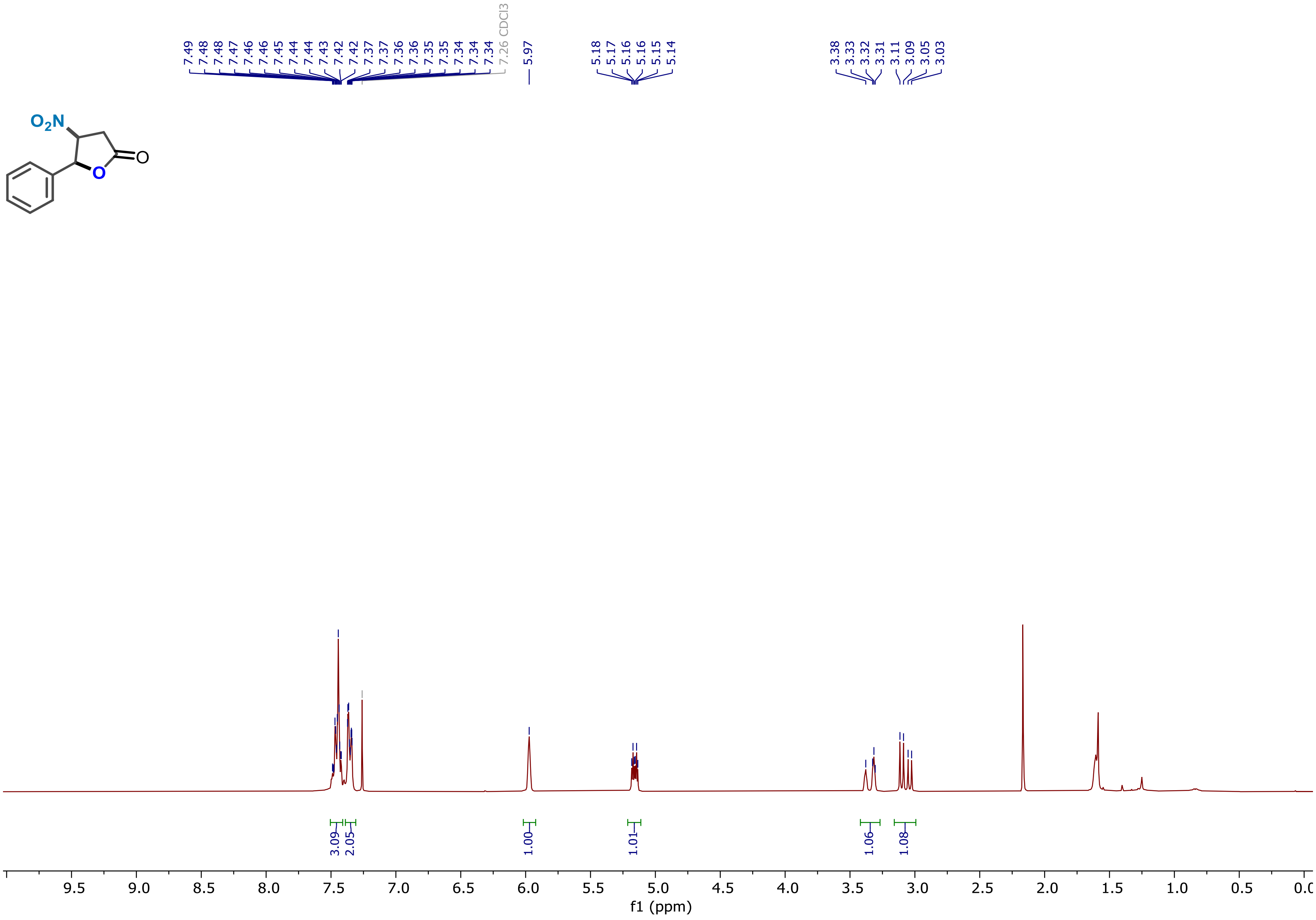

^{13}C NMR (75 MHz, $CDCl_3$) of **44**

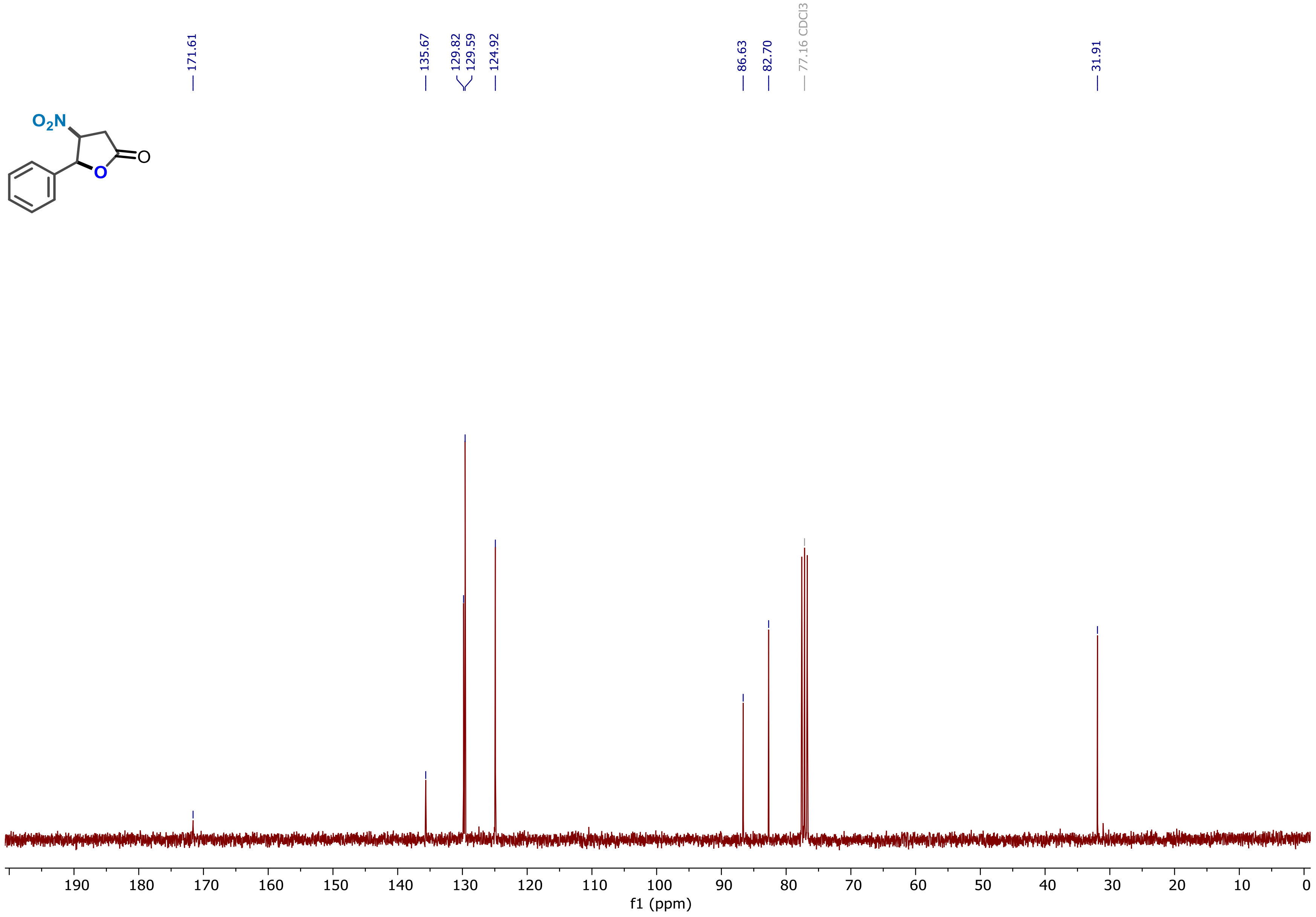

^{1}H NMR (300 MHz, $CDCl_3$) of **45**

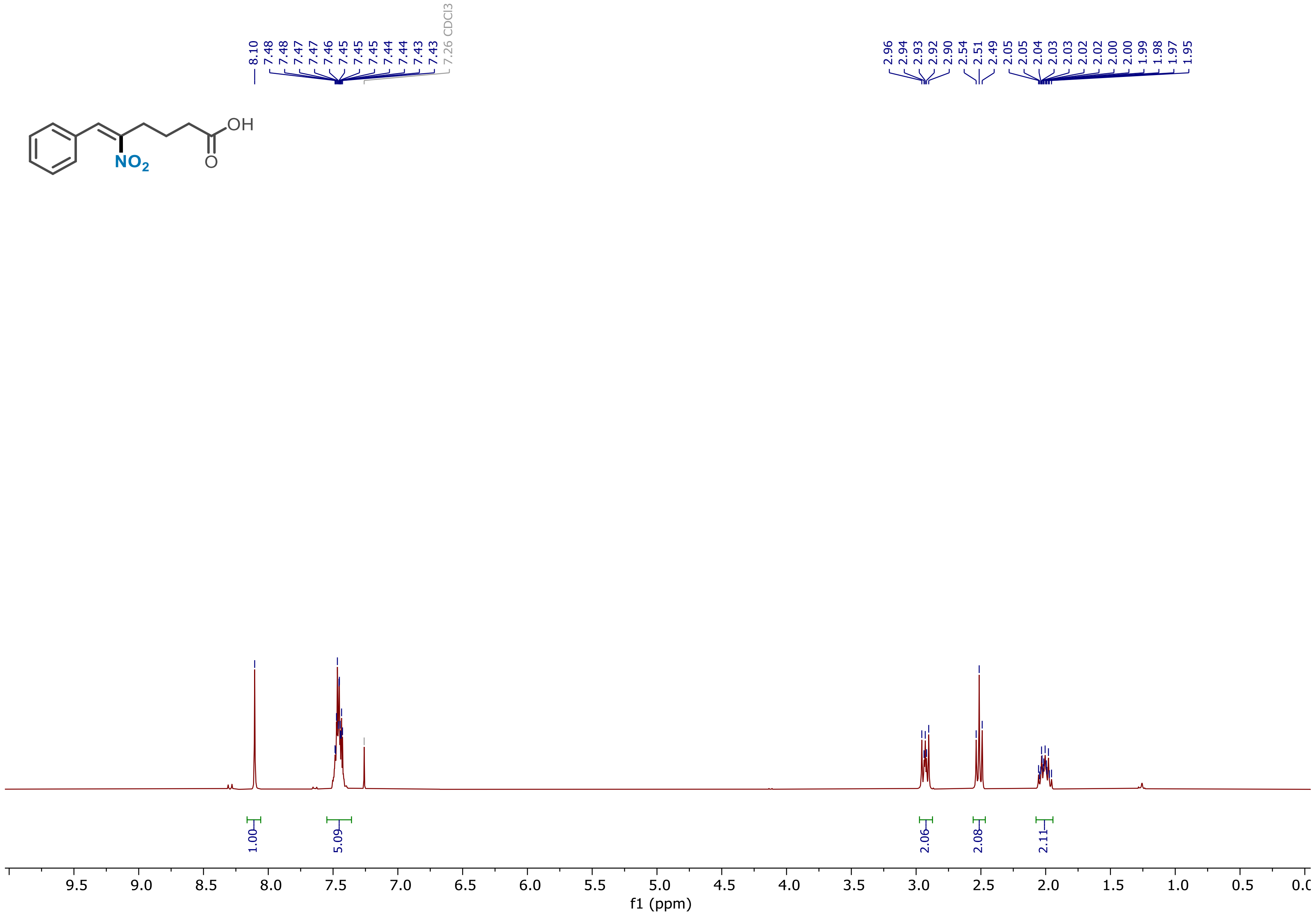

^{13}C NMR (75 MHz, $CDCl_3$) of **45**

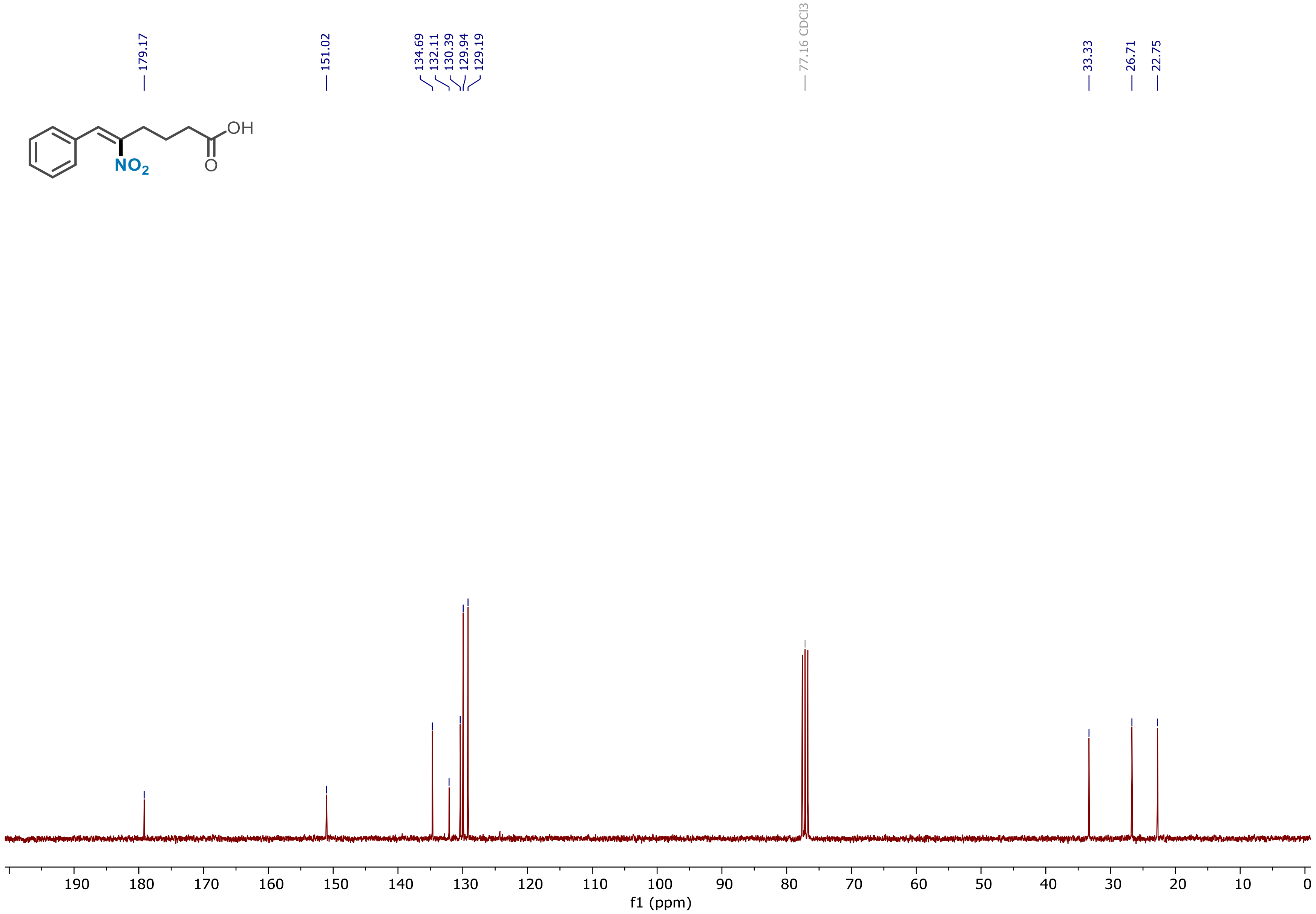

^{1}H NMR (300 MHz, $CDCl_3$) of **46**

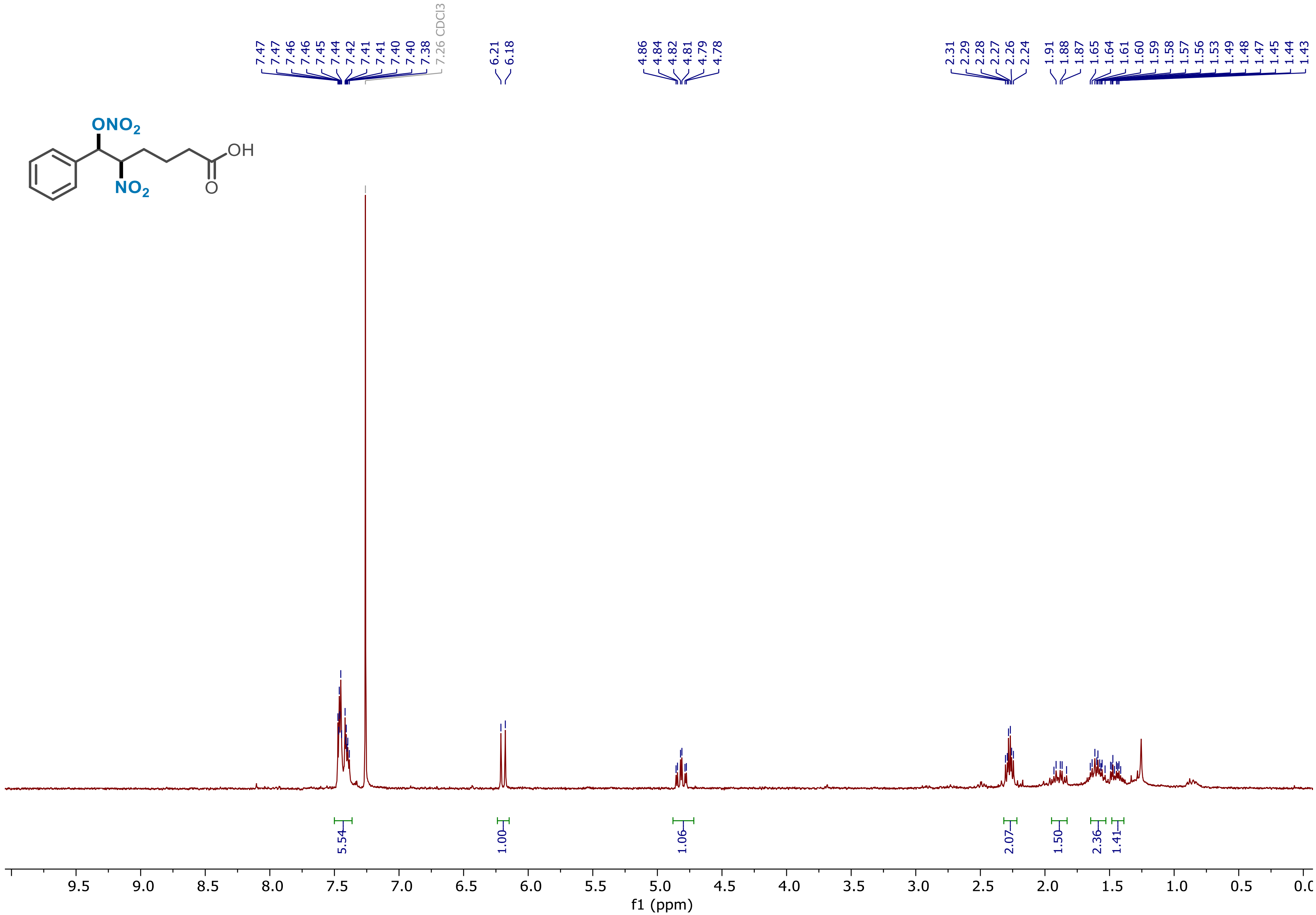

^{1}H NMR (300 MHz, $CDCl_3$) of **47**

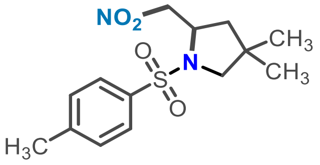

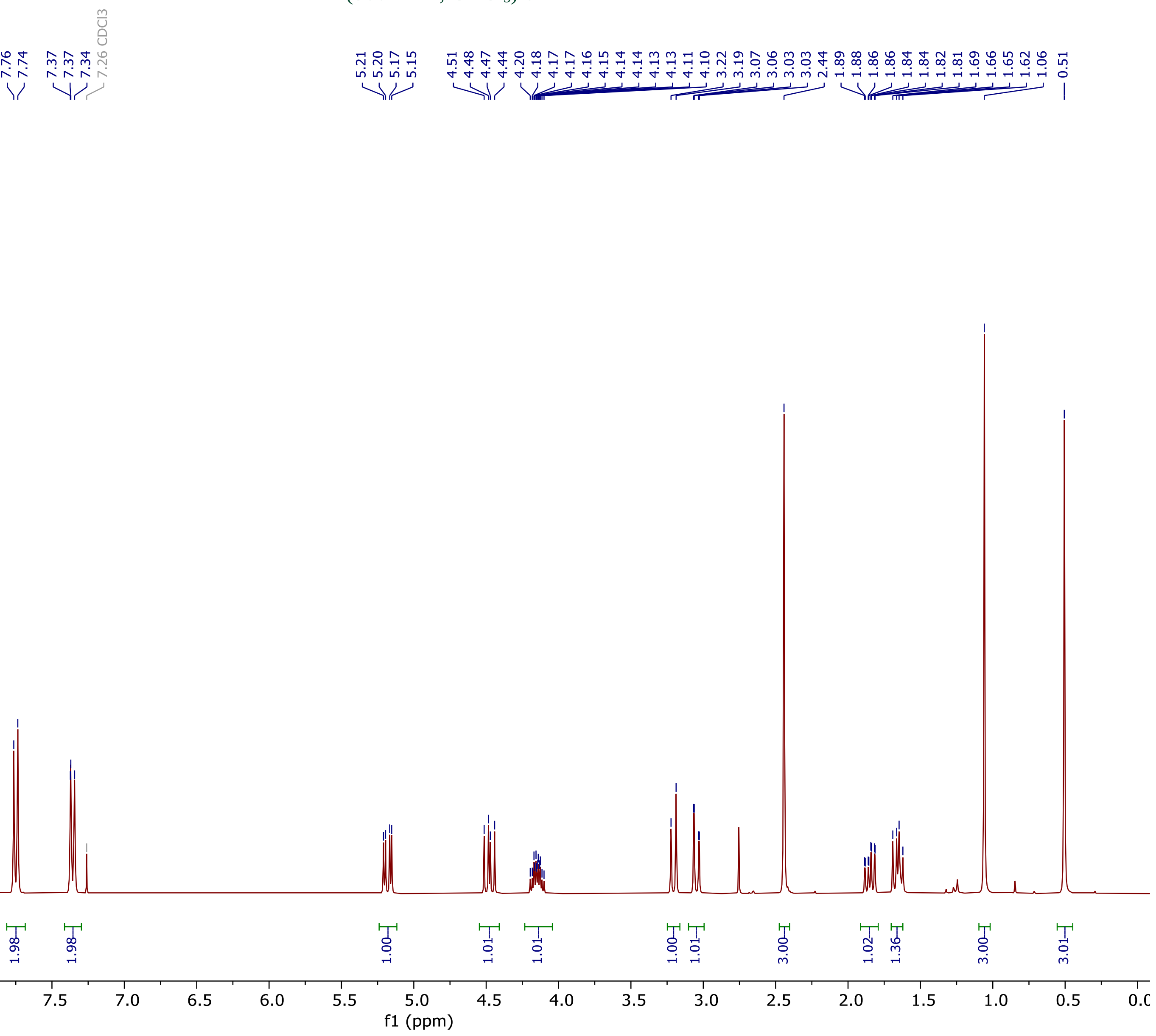

^{13}C NMR (75 MHz, $CDCl_3$) of **47**

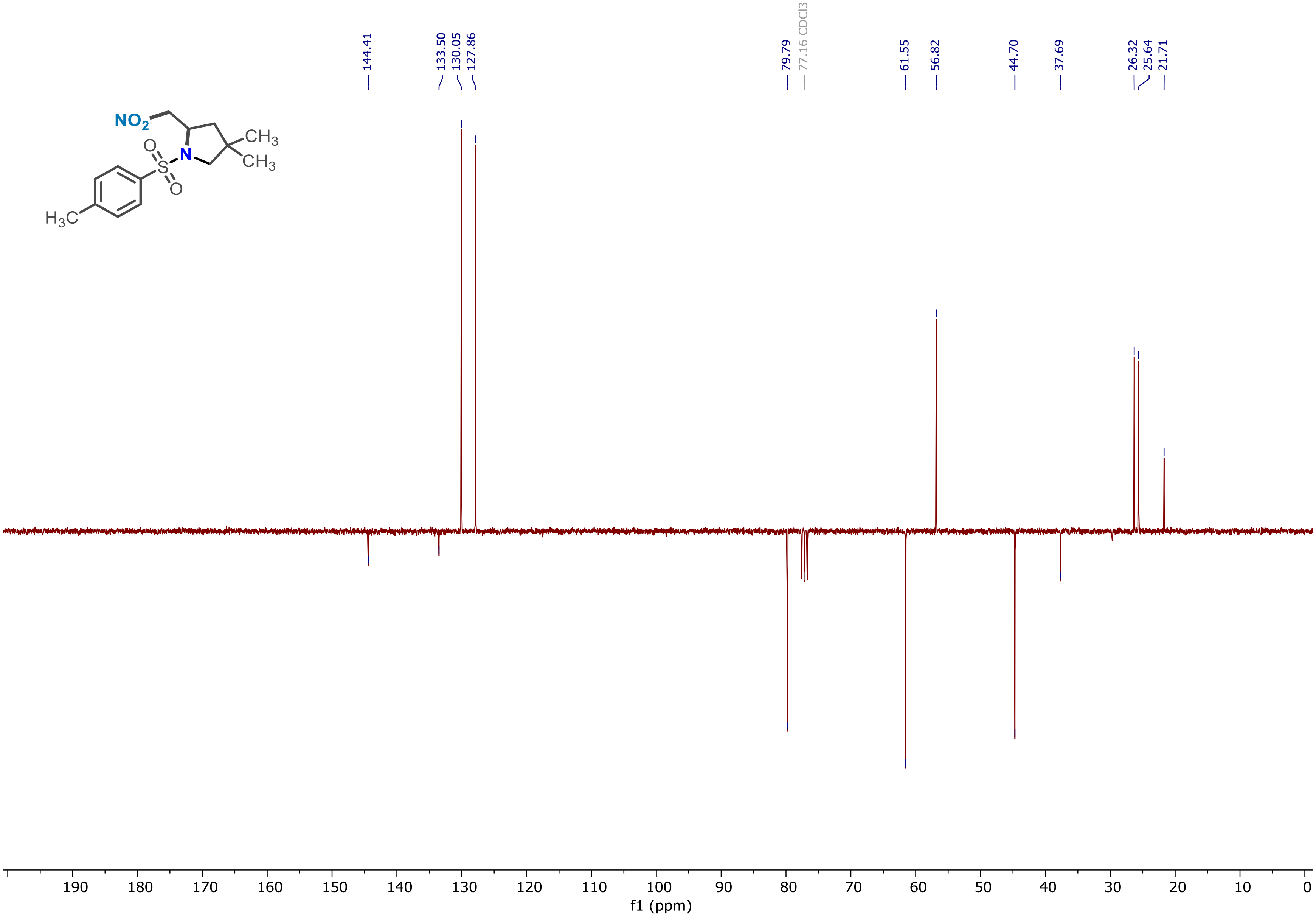

^1H NMR (300 MHz, $CDCl_3$) of **48**

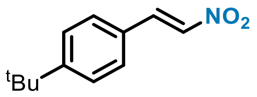

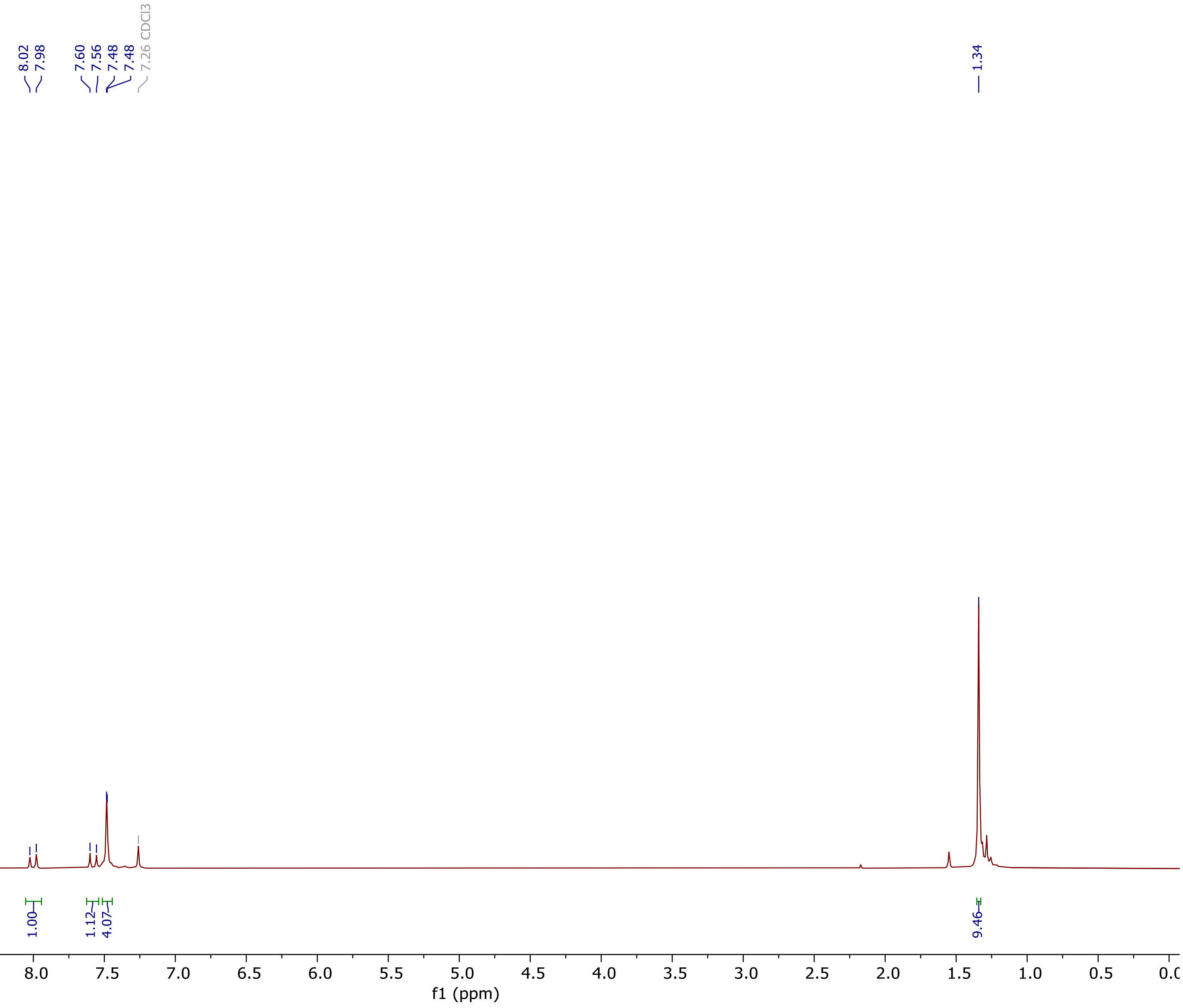

^{1}H NMR (300 MHz, $CDCl_3$) of **49**

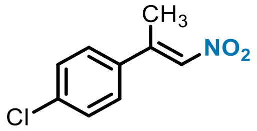

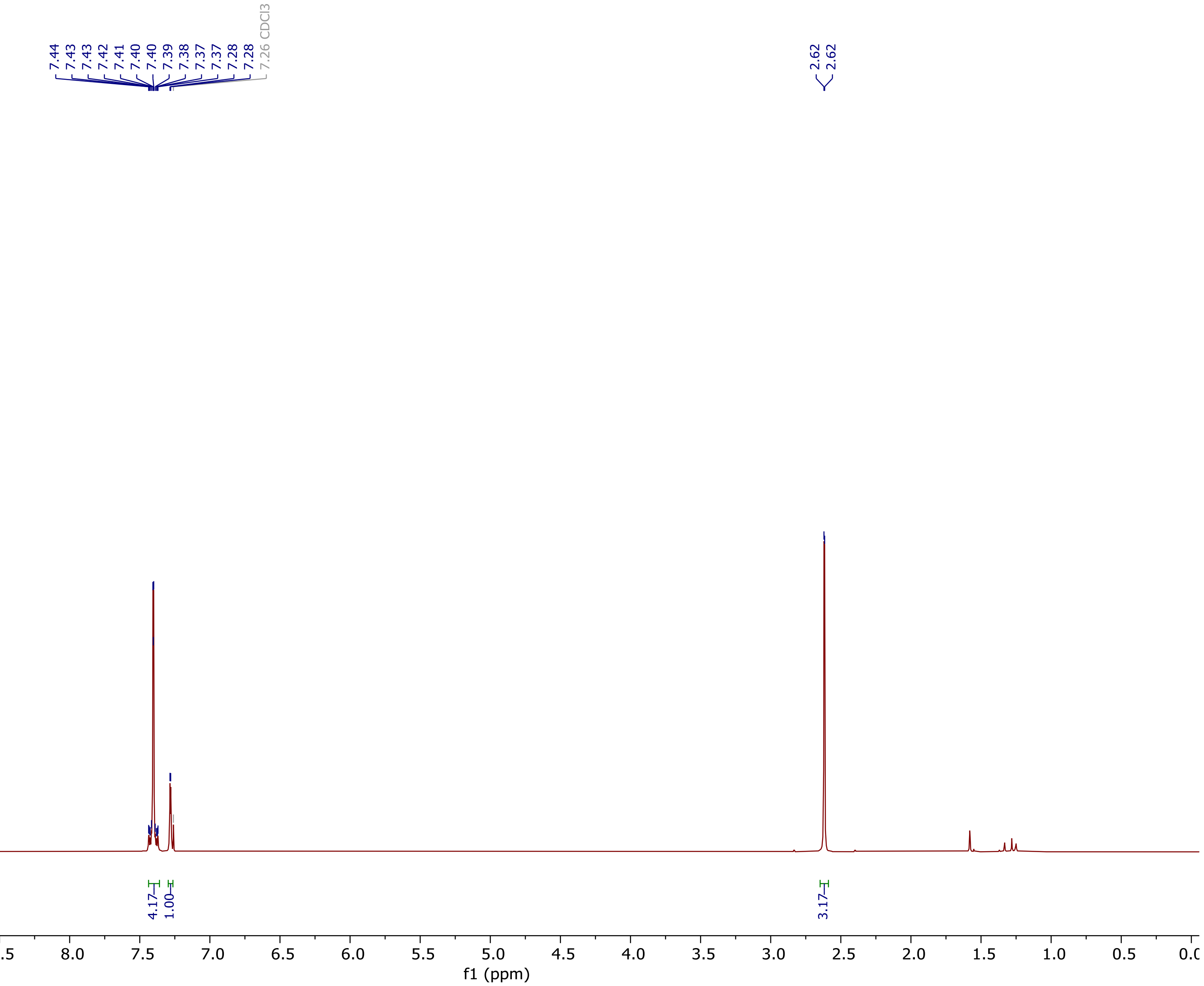

^{13}C NMR (75 MHz, $CDCl_3$) of **49**

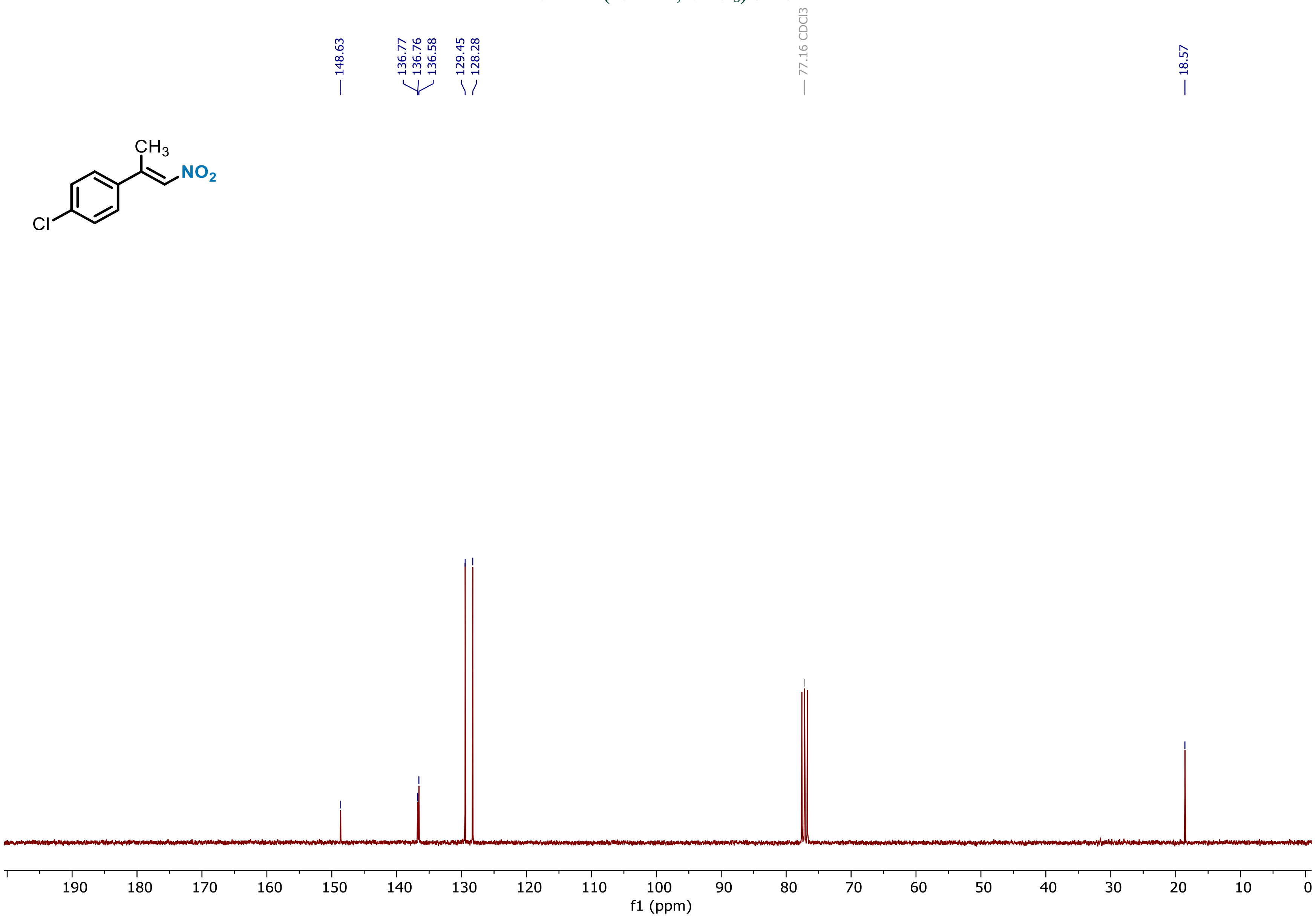

^{1}H NMR (300 MHz, $CDCl_3$) of **50**

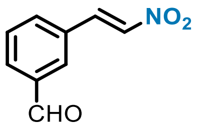

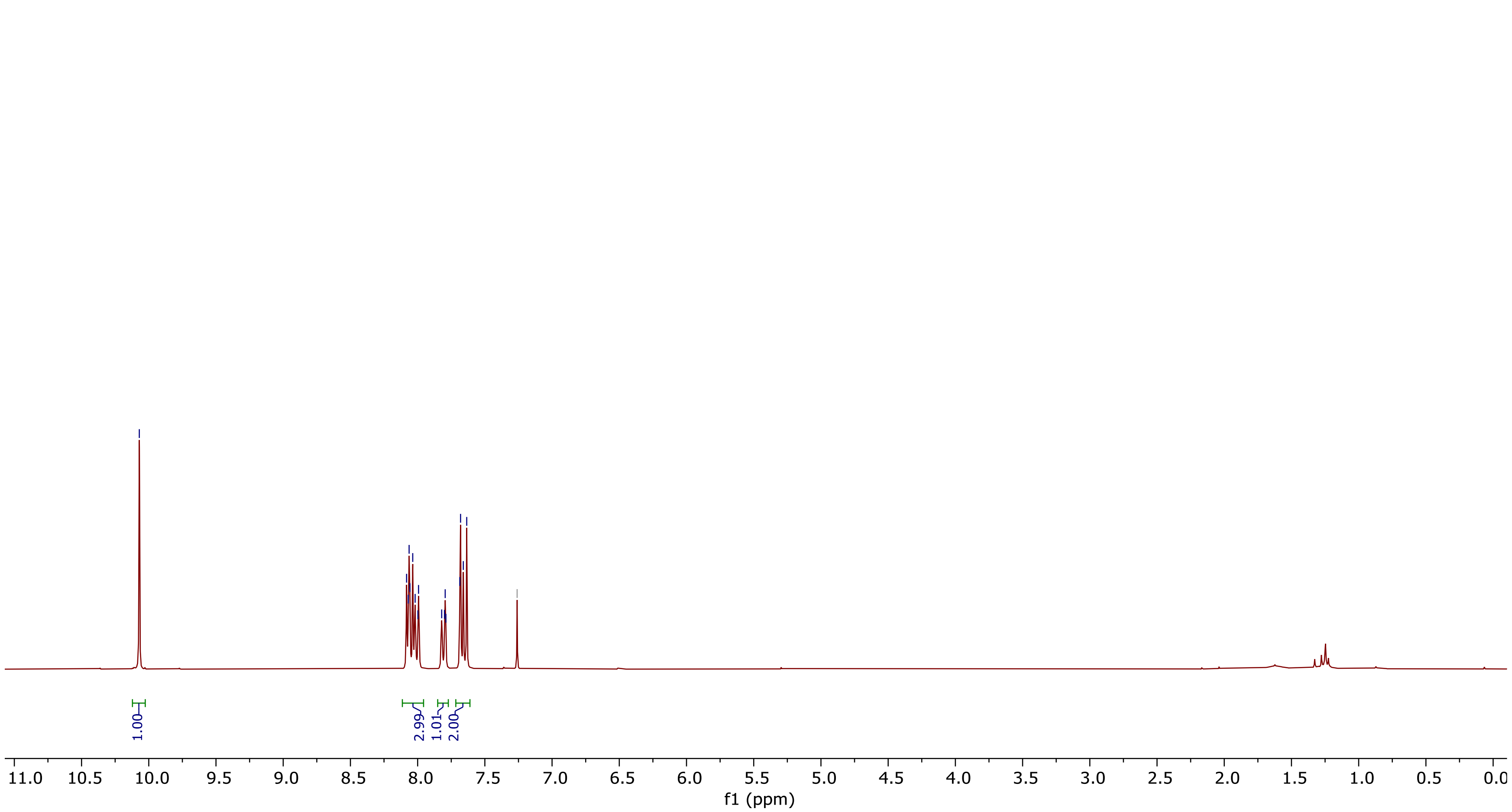

^{13}C NMR (75 MHz, $CDCl_3$) of **50**

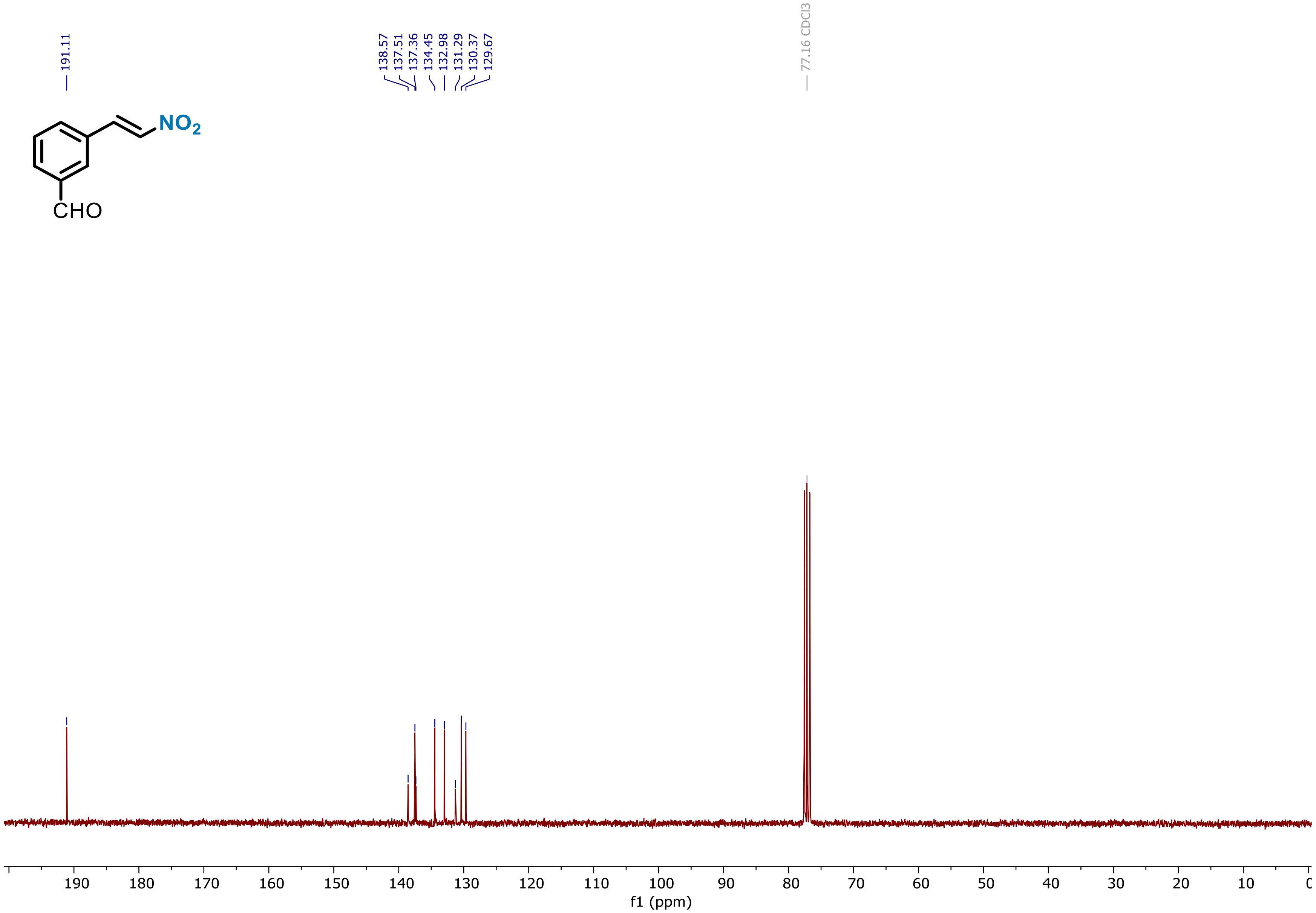

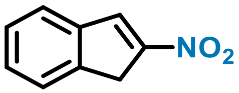

^1H NMR (300 MHz, $CDCl_3$) of **51**

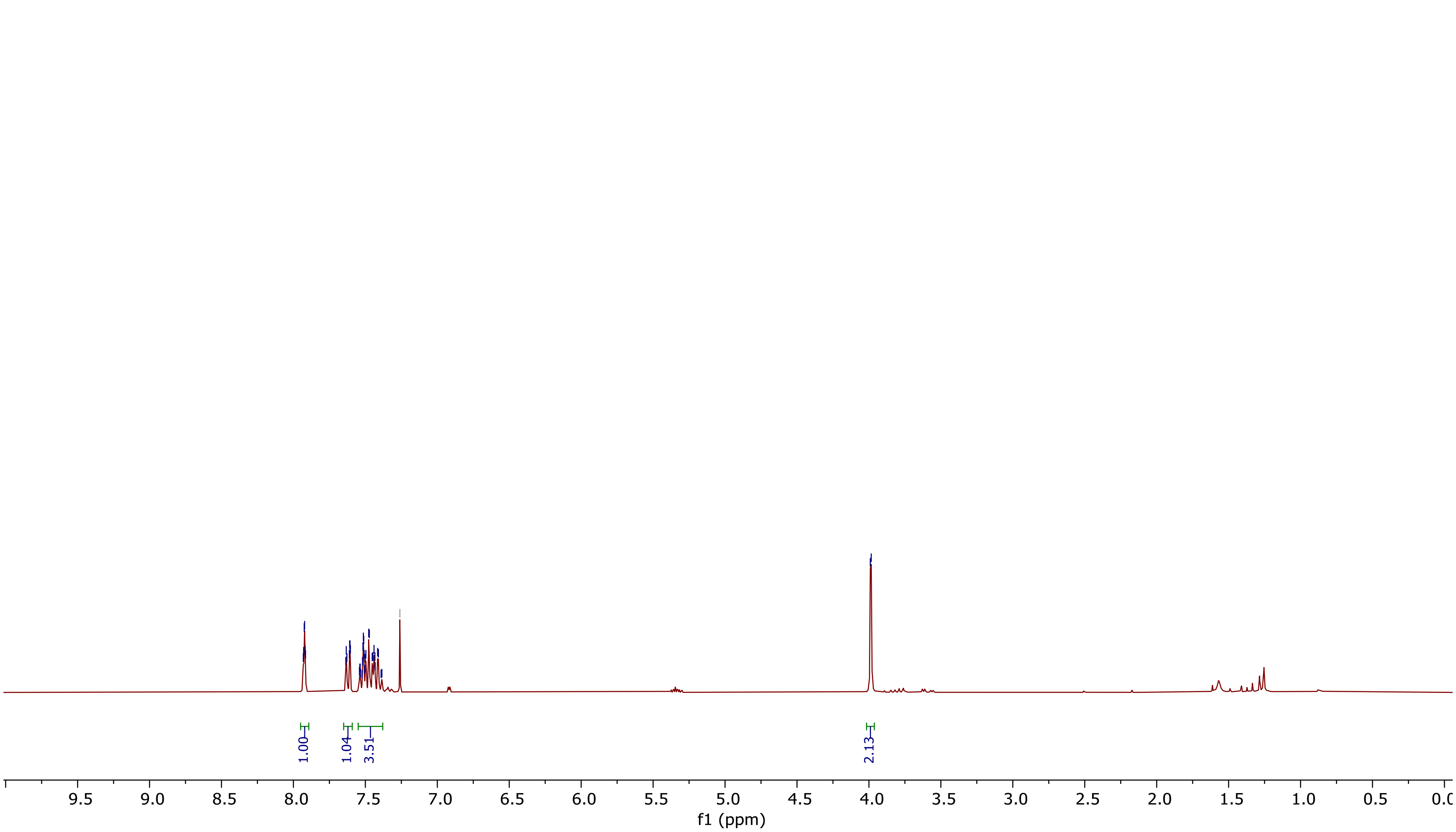

^{13}C NMR (75 MHz, $CDCl_3$) of **51**

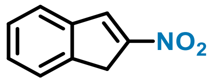

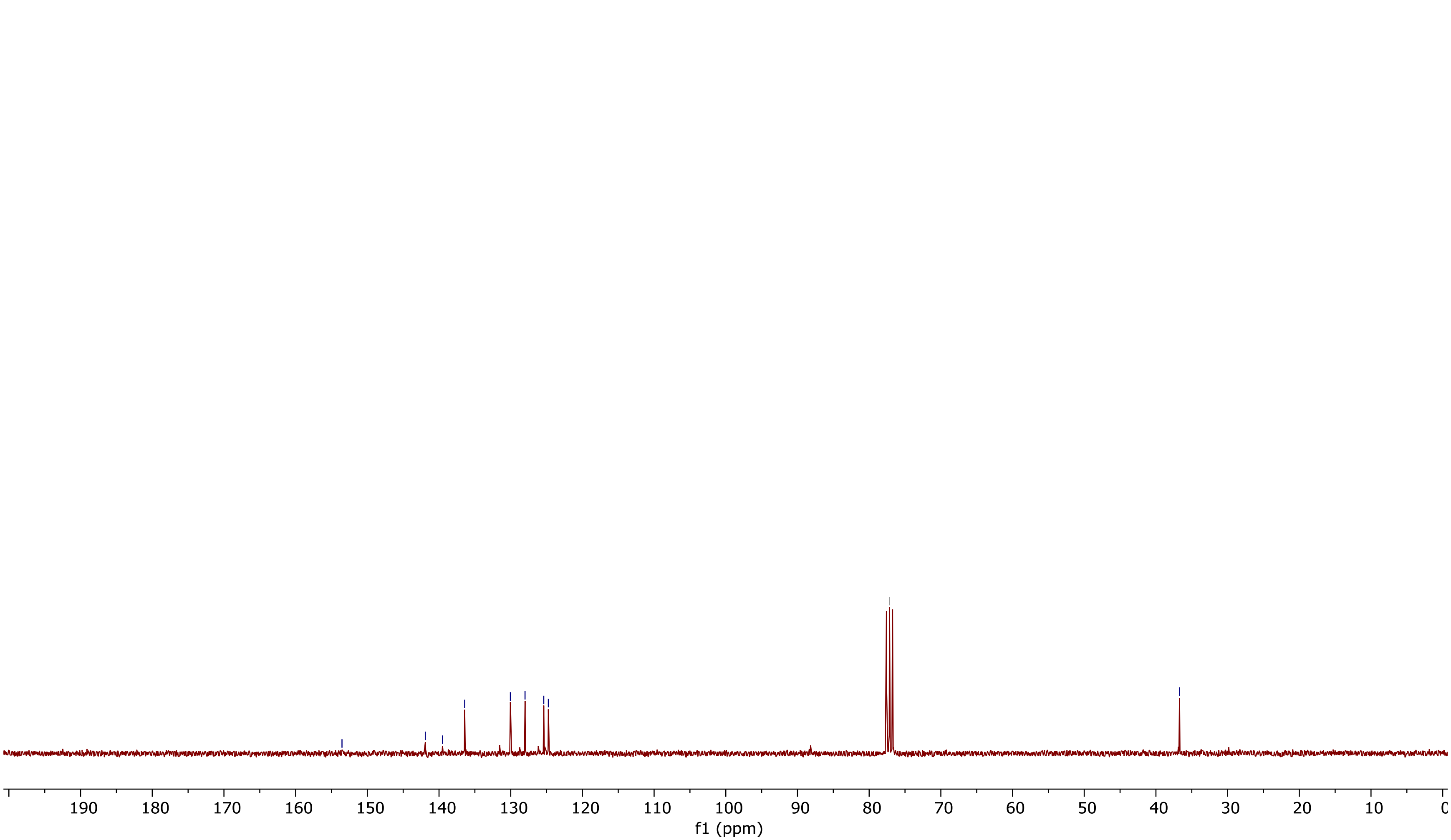

^{1}H NMR (300 MHz, $CDCl_3$) of **52**

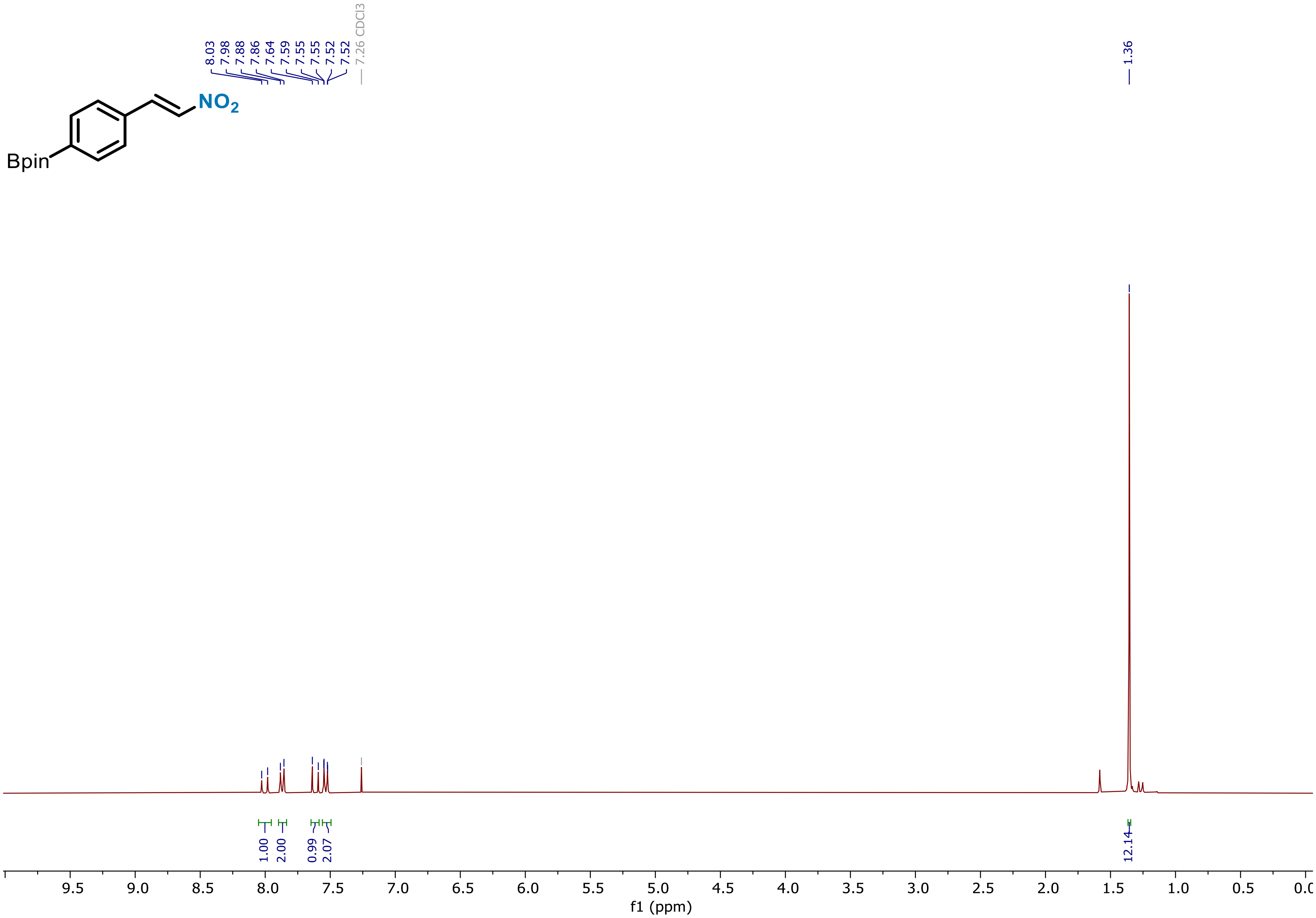

^{13}C NMR (75 MHz, $CDCl_3$) of **52**

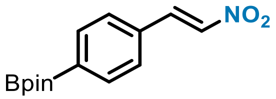

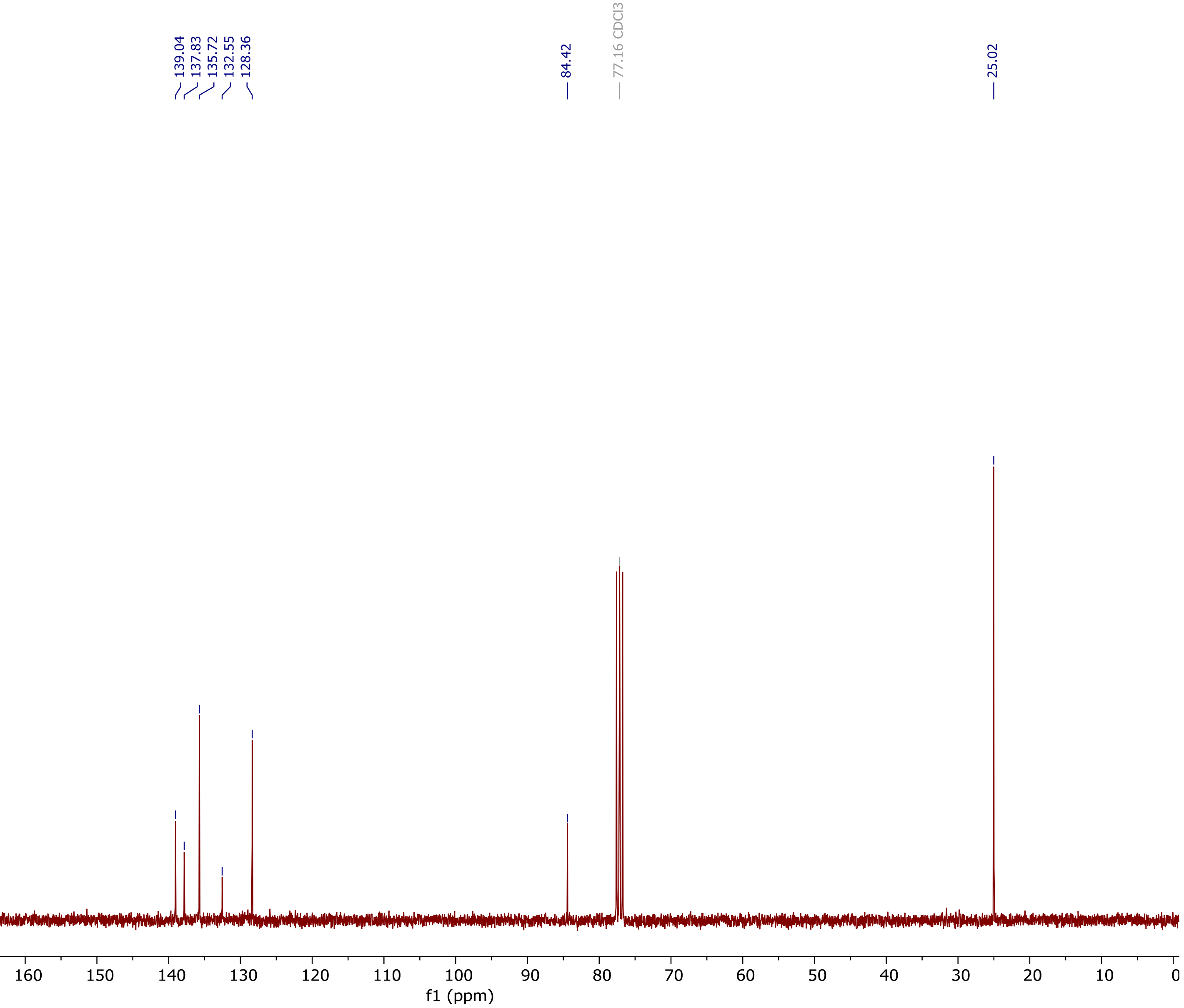

^{1}H NMR (300 MHz, $CDCl_3$) of **53**

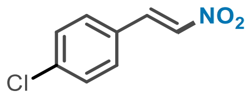

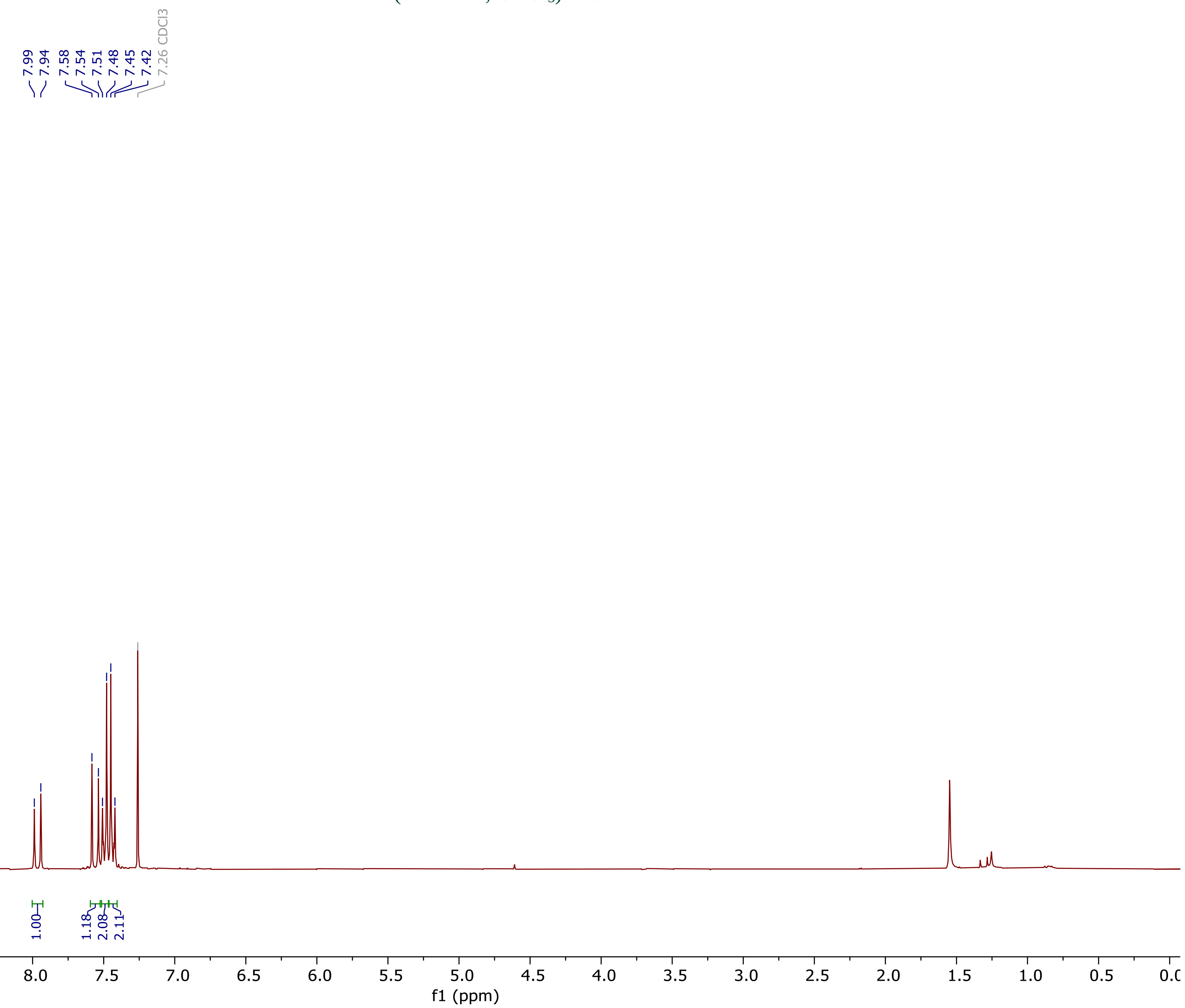

^{1}H NMR (300 MHz, $CDCl_3$) of **54**

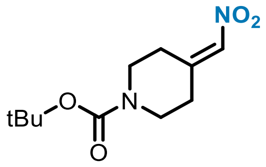

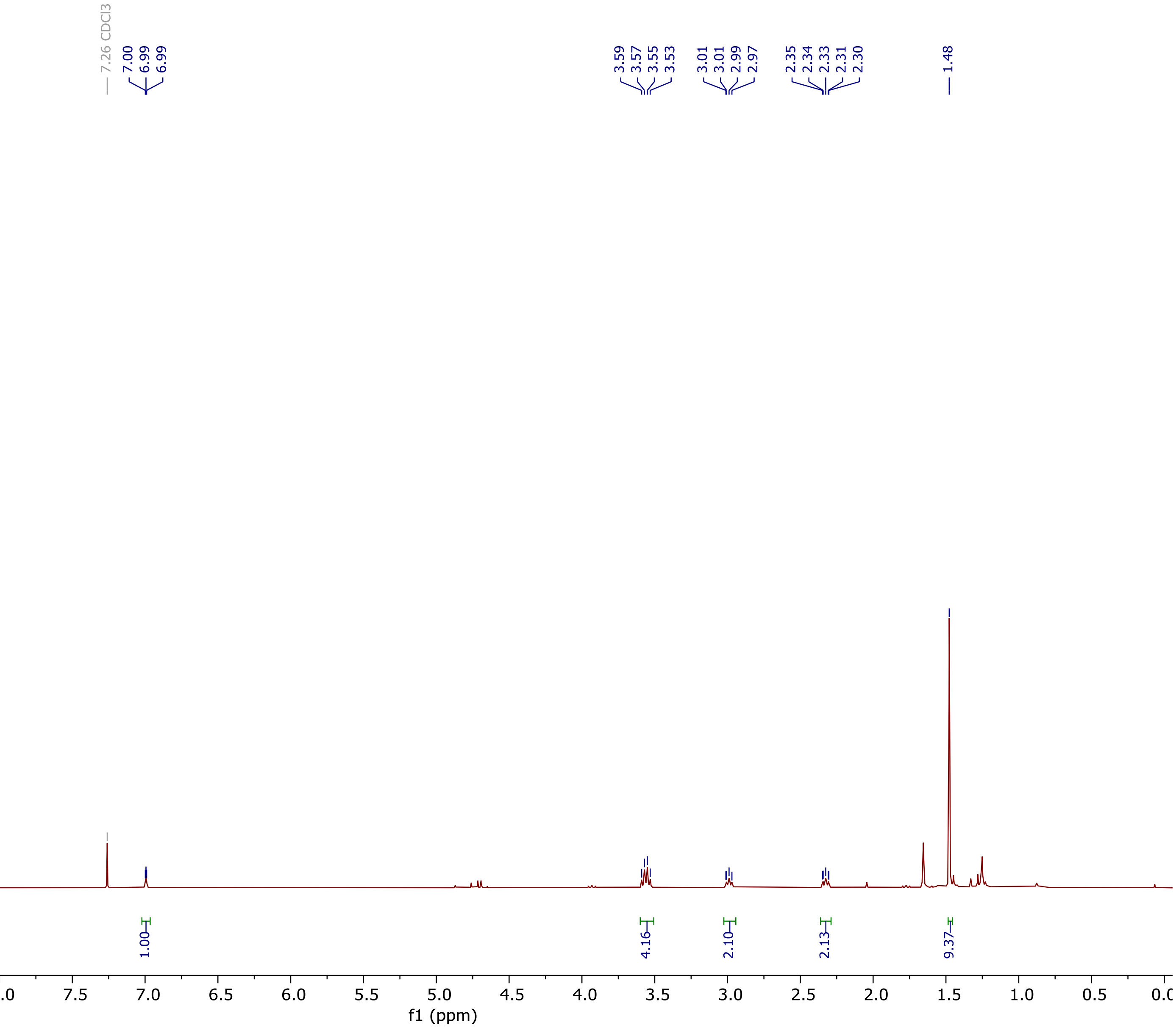

^{13}C NMR (75 MHz, $CDCl_3$) of **54**

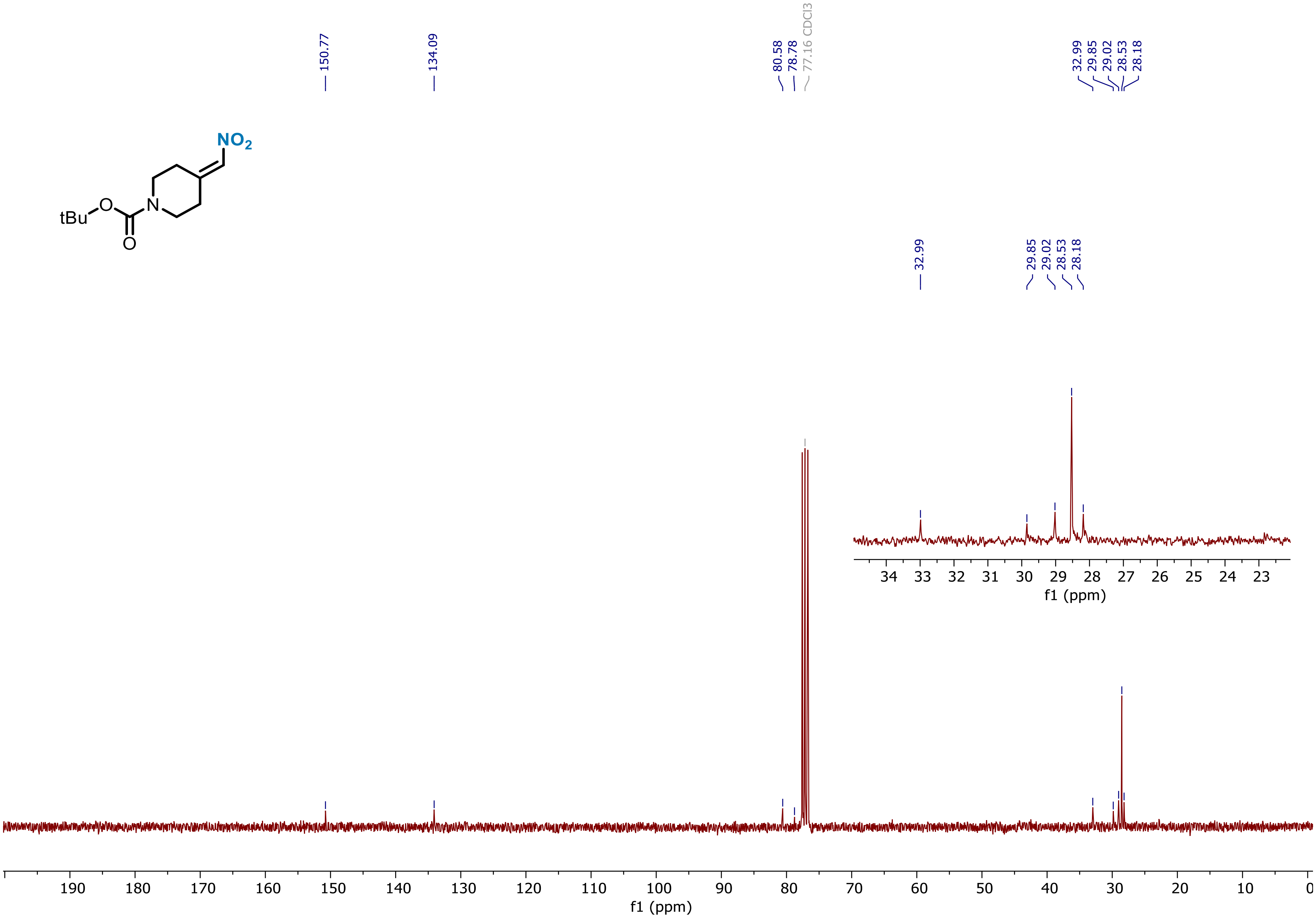

^{1}H NMR (300 MHz, $CDCl_3$) of **55**

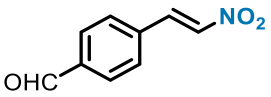

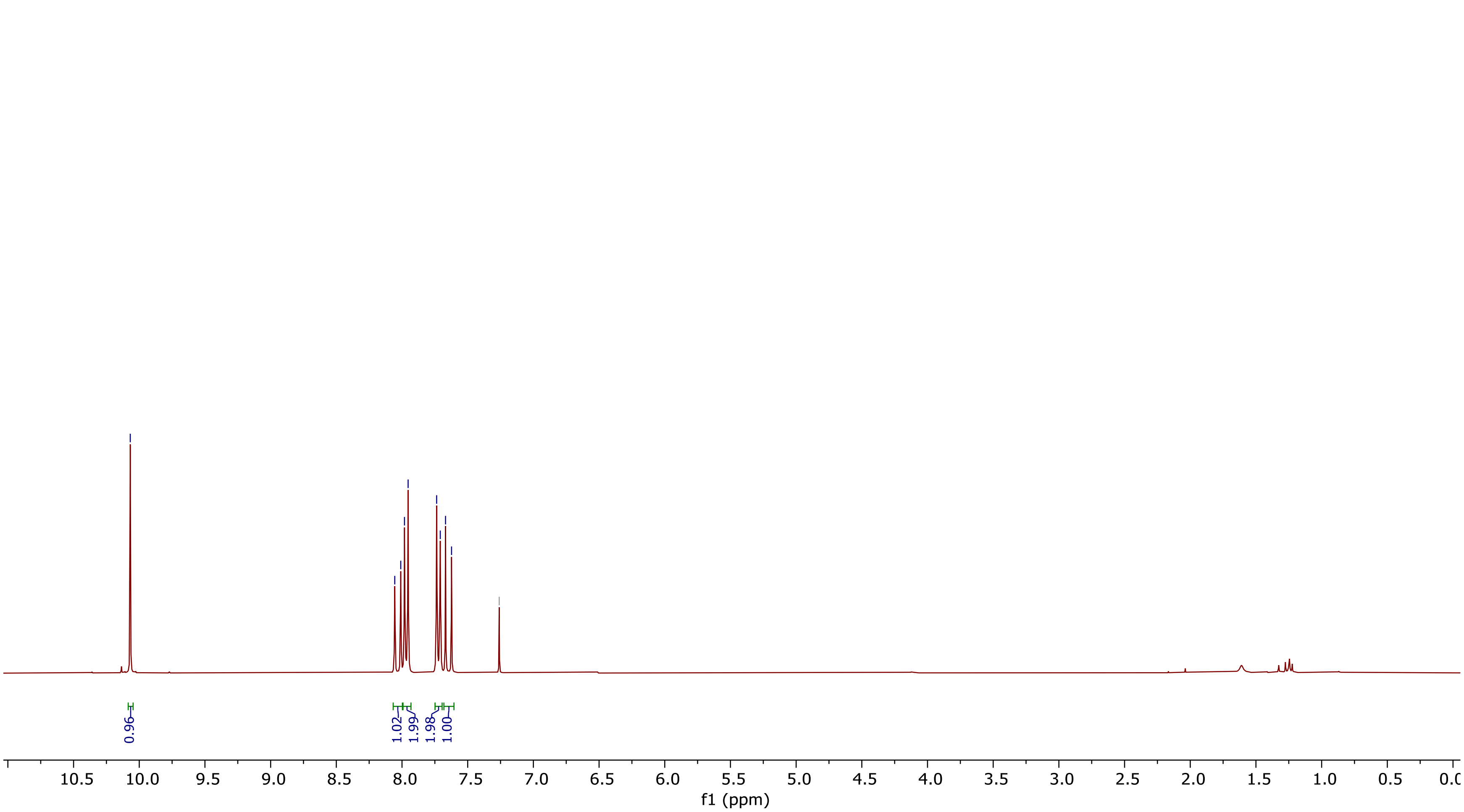

^{13}C NMR (75 MHz, $CDCl_3$) of **55**

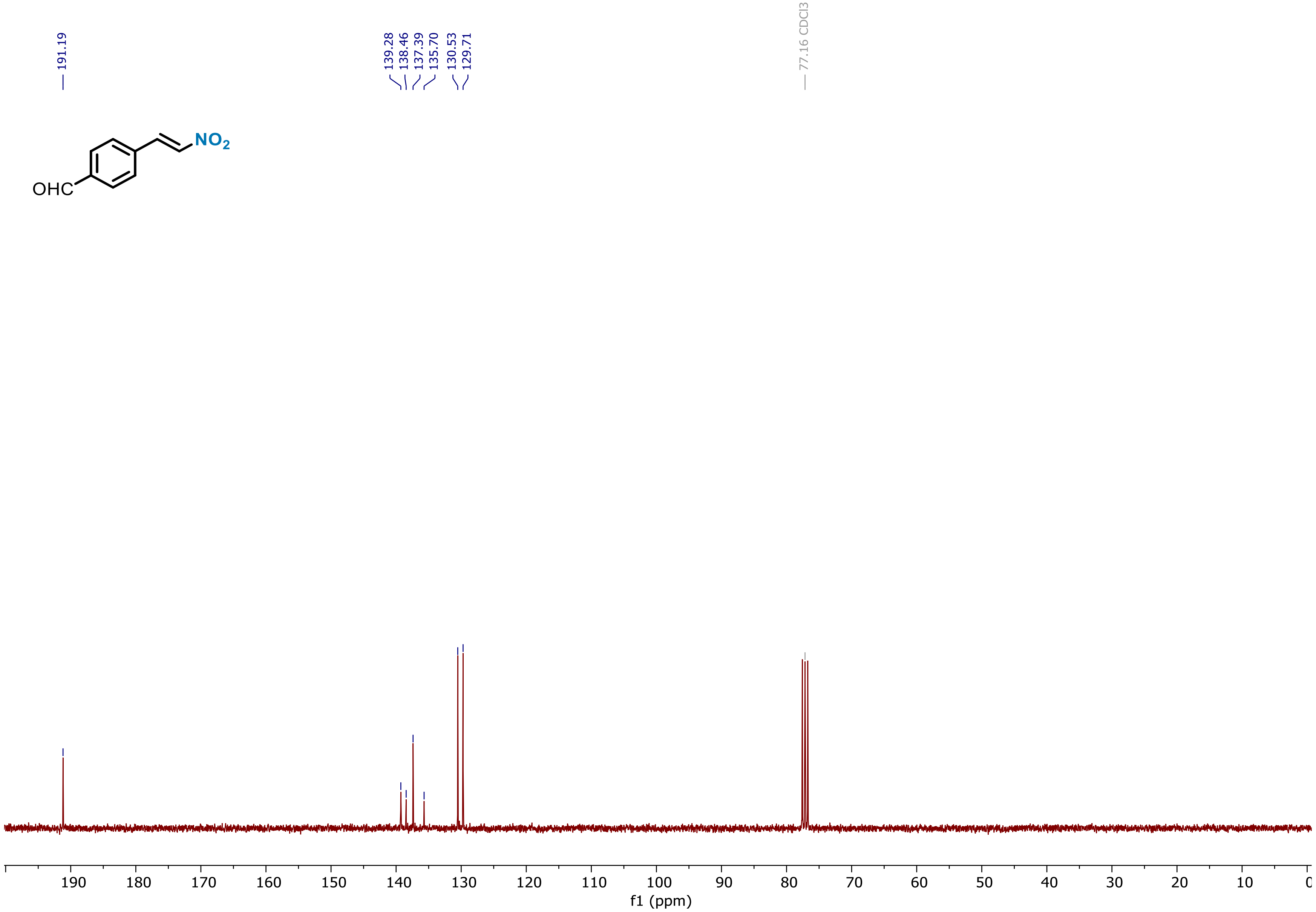

^{1}H NMR (300 MHz, $CDCl_3$) of **56**

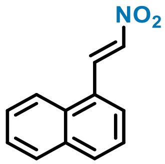

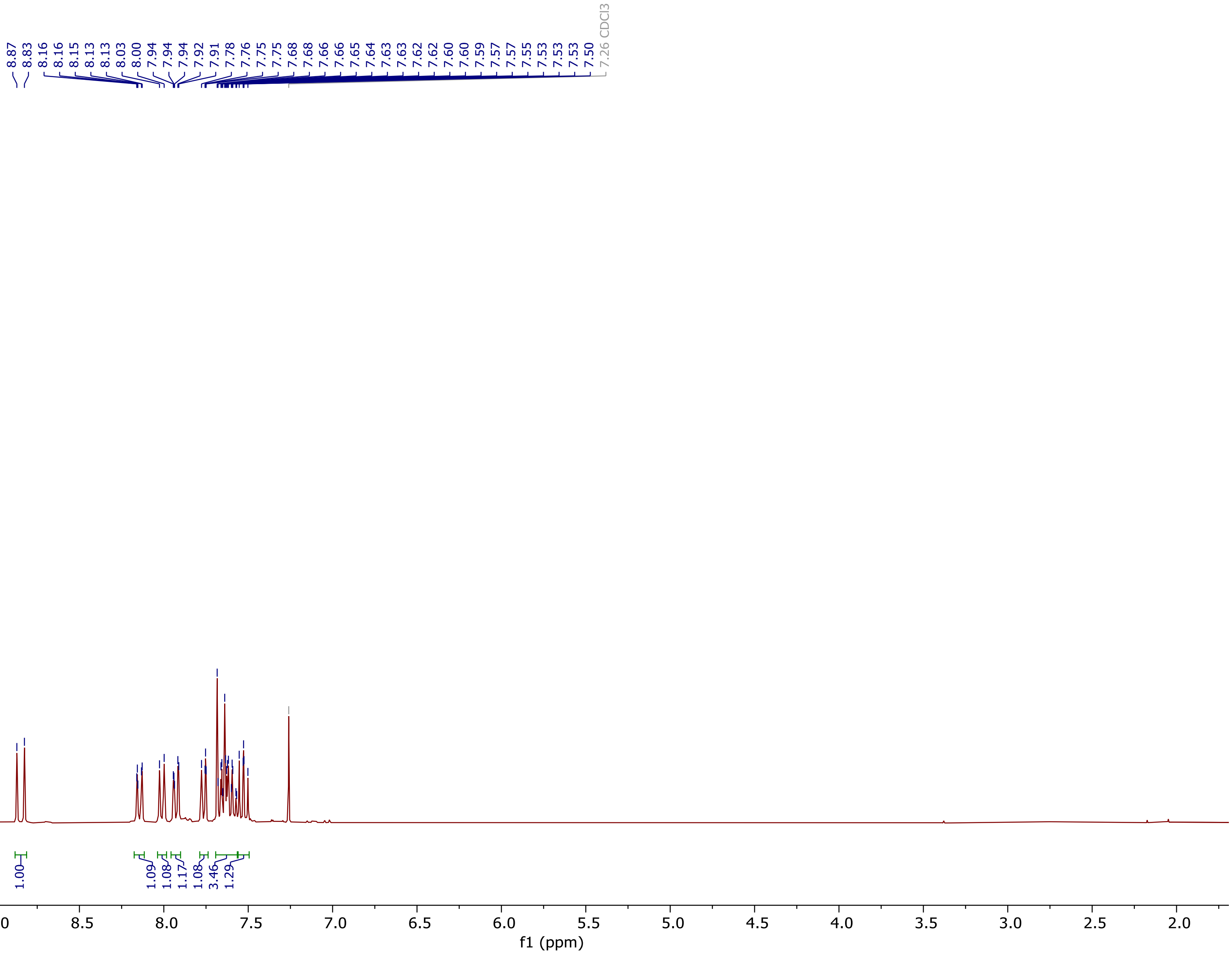

^{13}C NMR (75 MHz, $CDCl_3$) of **56**

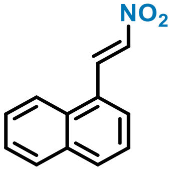

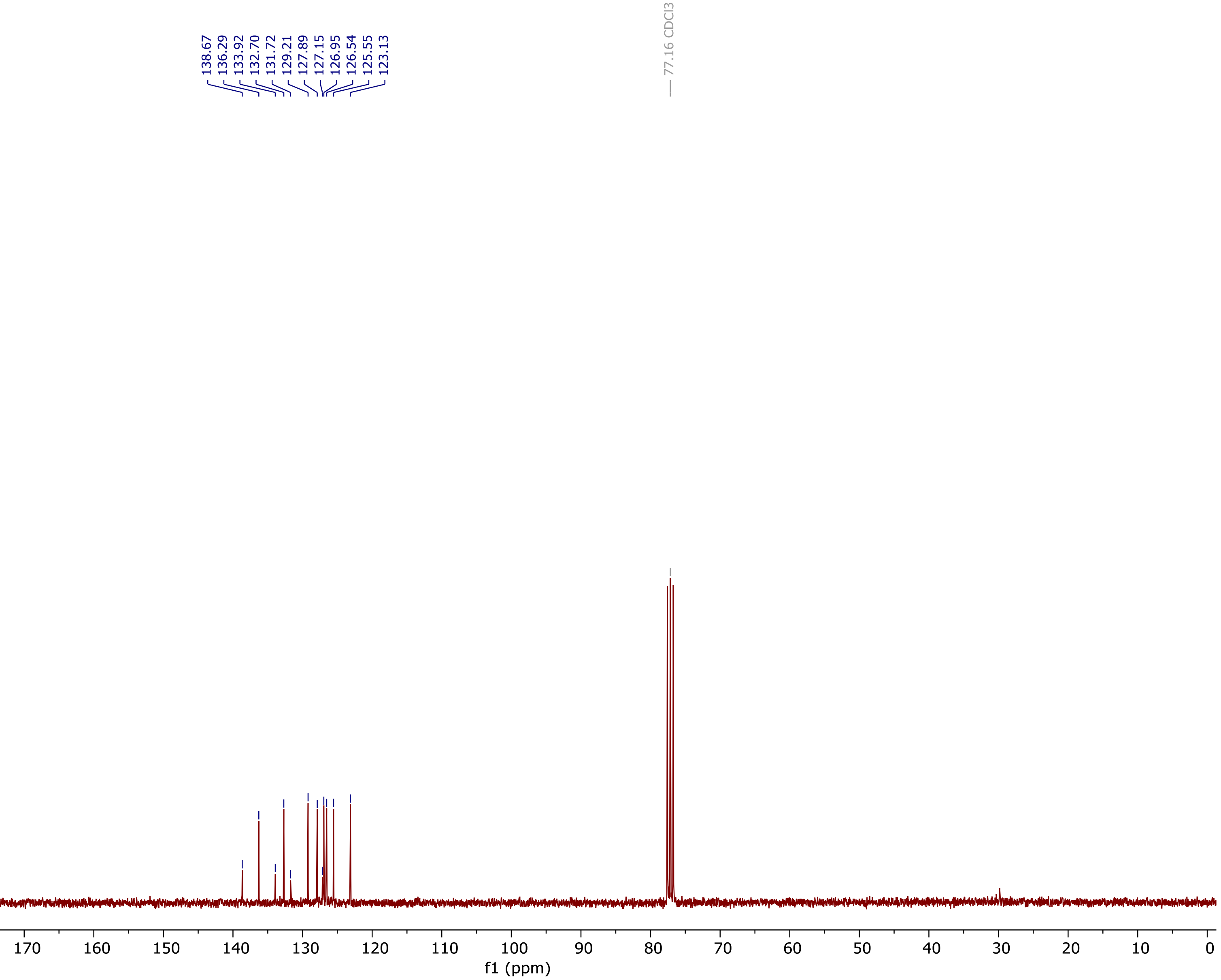

^{1}H NMR (300 MHz, $CDCl_3$) of **57**

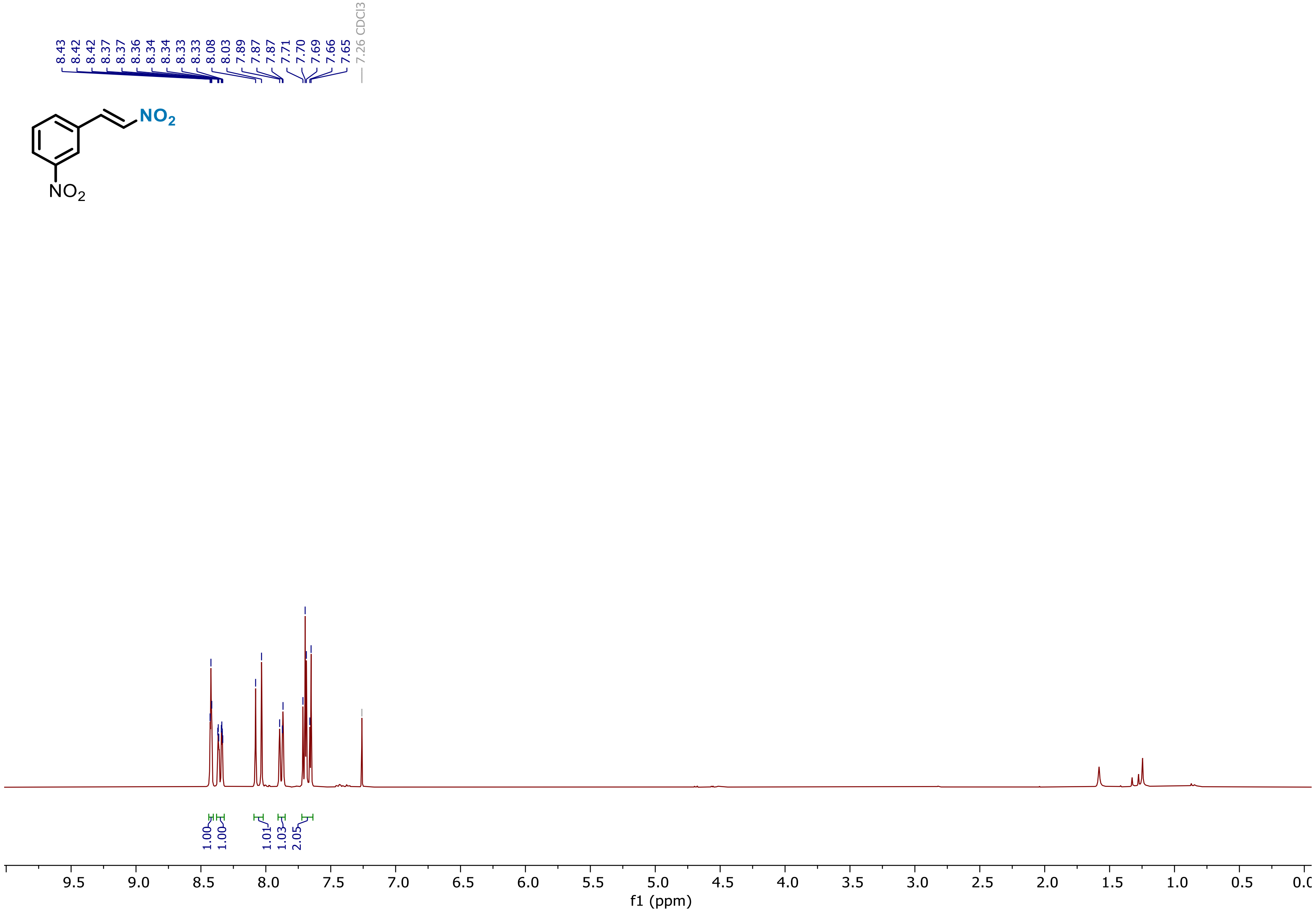

^{13}C NMR (75 MHz, $CDCl_3$) of **57**

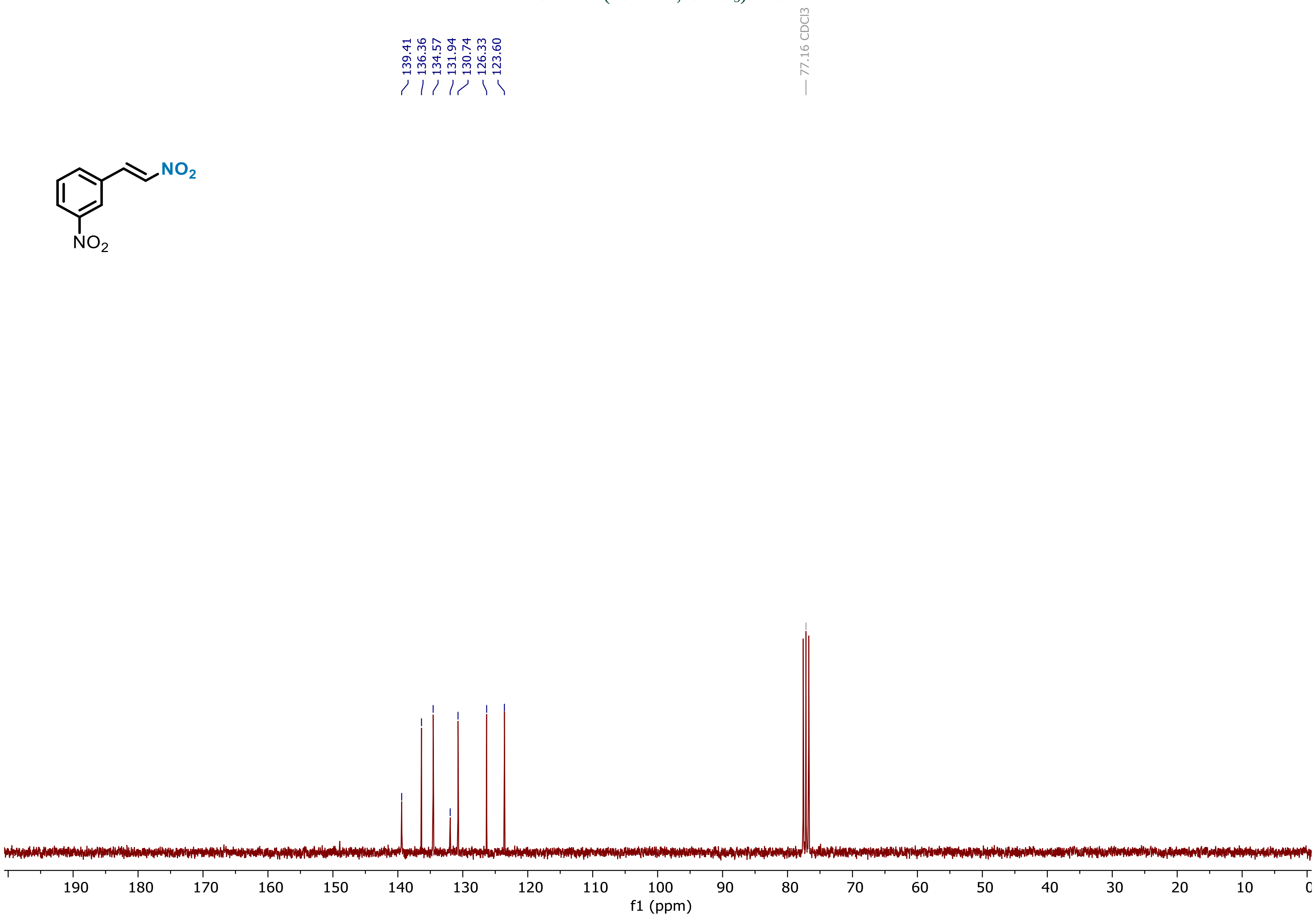

^{1}H NMR (300 MHz, $CDCl_3$) of **58**

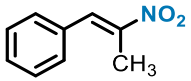

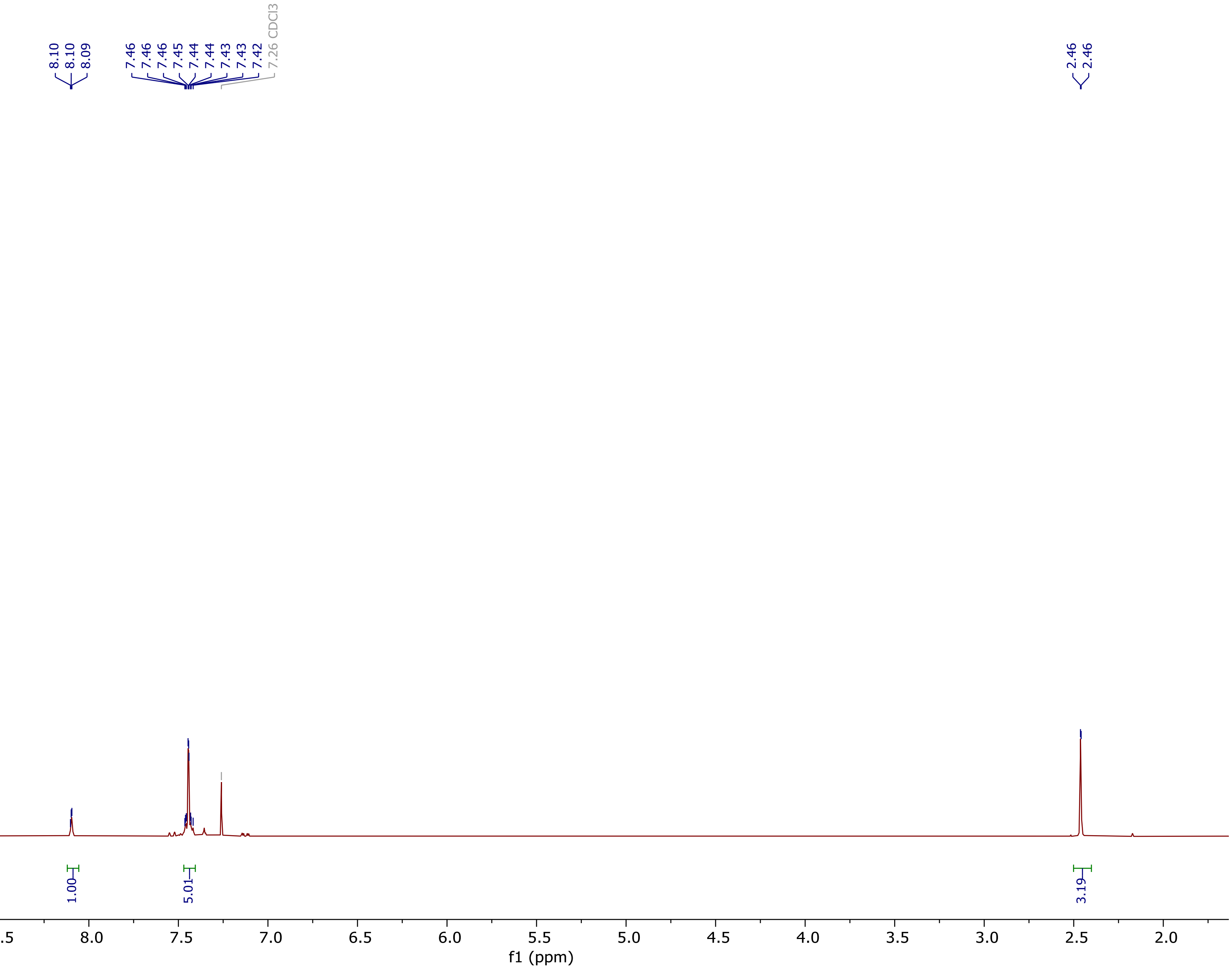

^{1}H NMR (300 MHz, $CDCl_3$) of ***E*-59**

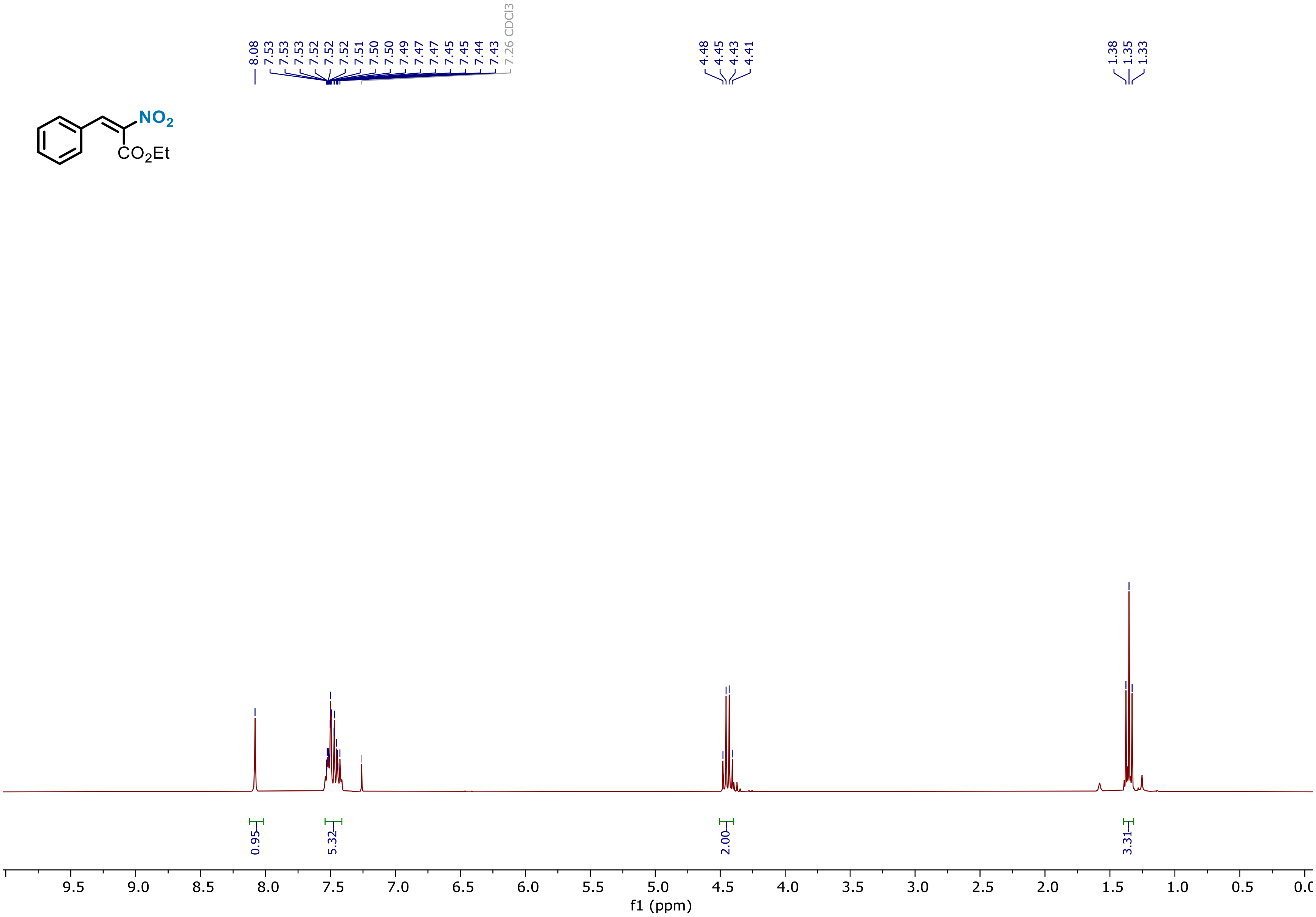

^{13}C NMR (75 MHz, $CDCl_3$) of ***E*-59**

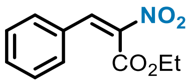

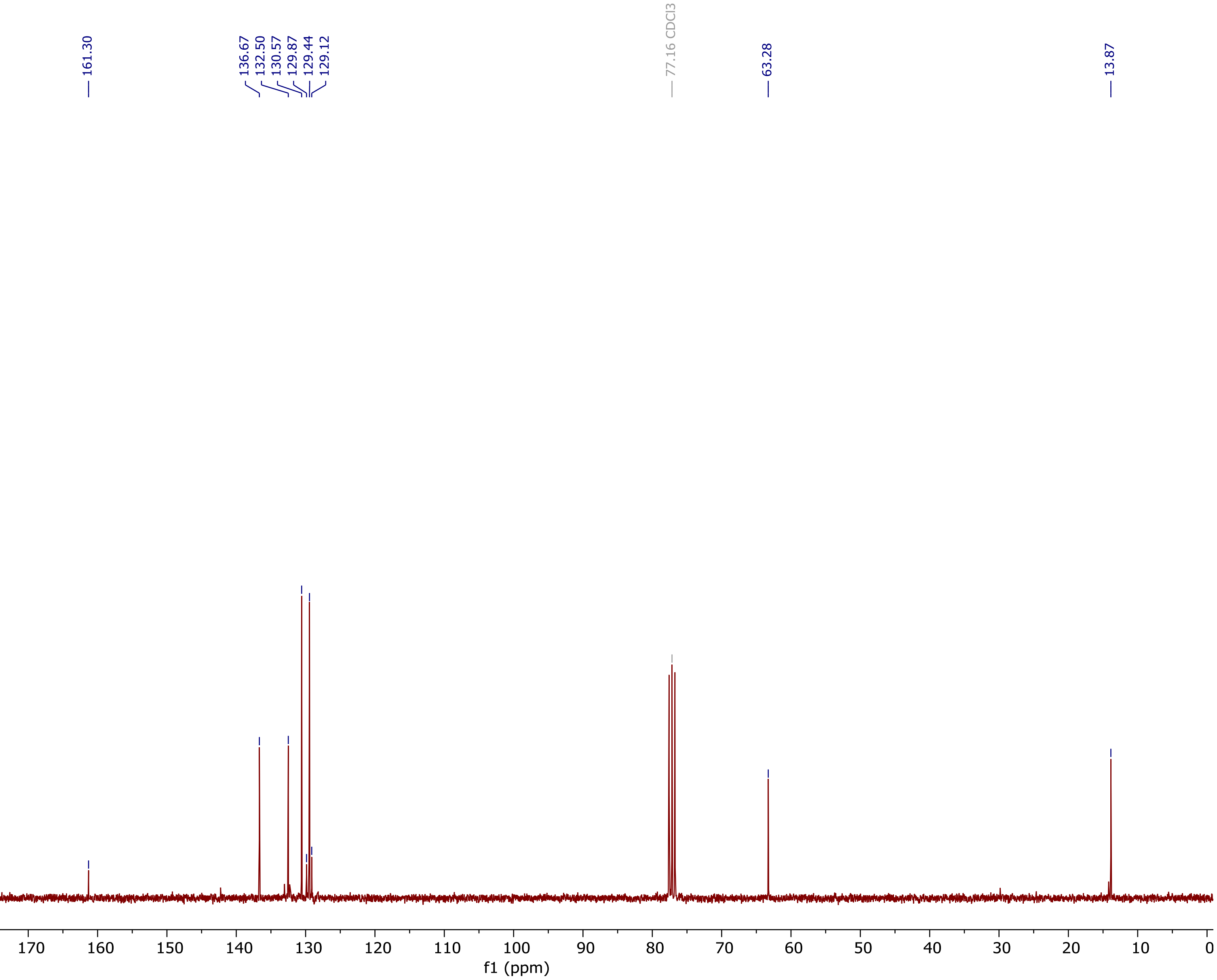

^1H NMR (300 MHz, $CDCl_3$) of ***Z*-59**

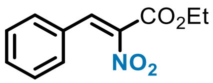

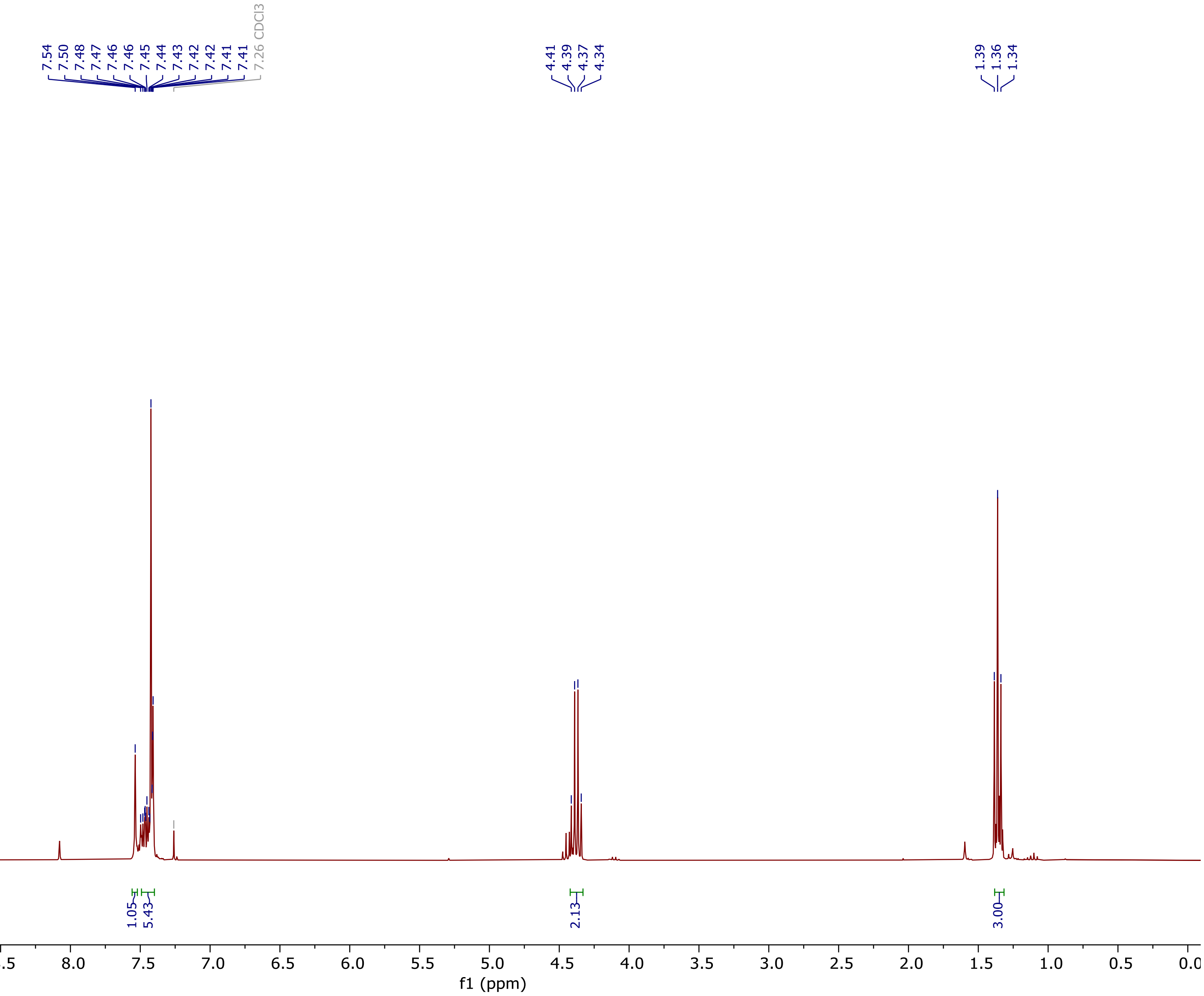

^{13}C NMR (75 MHz, $CDCl_3$) of ***Z*-59**

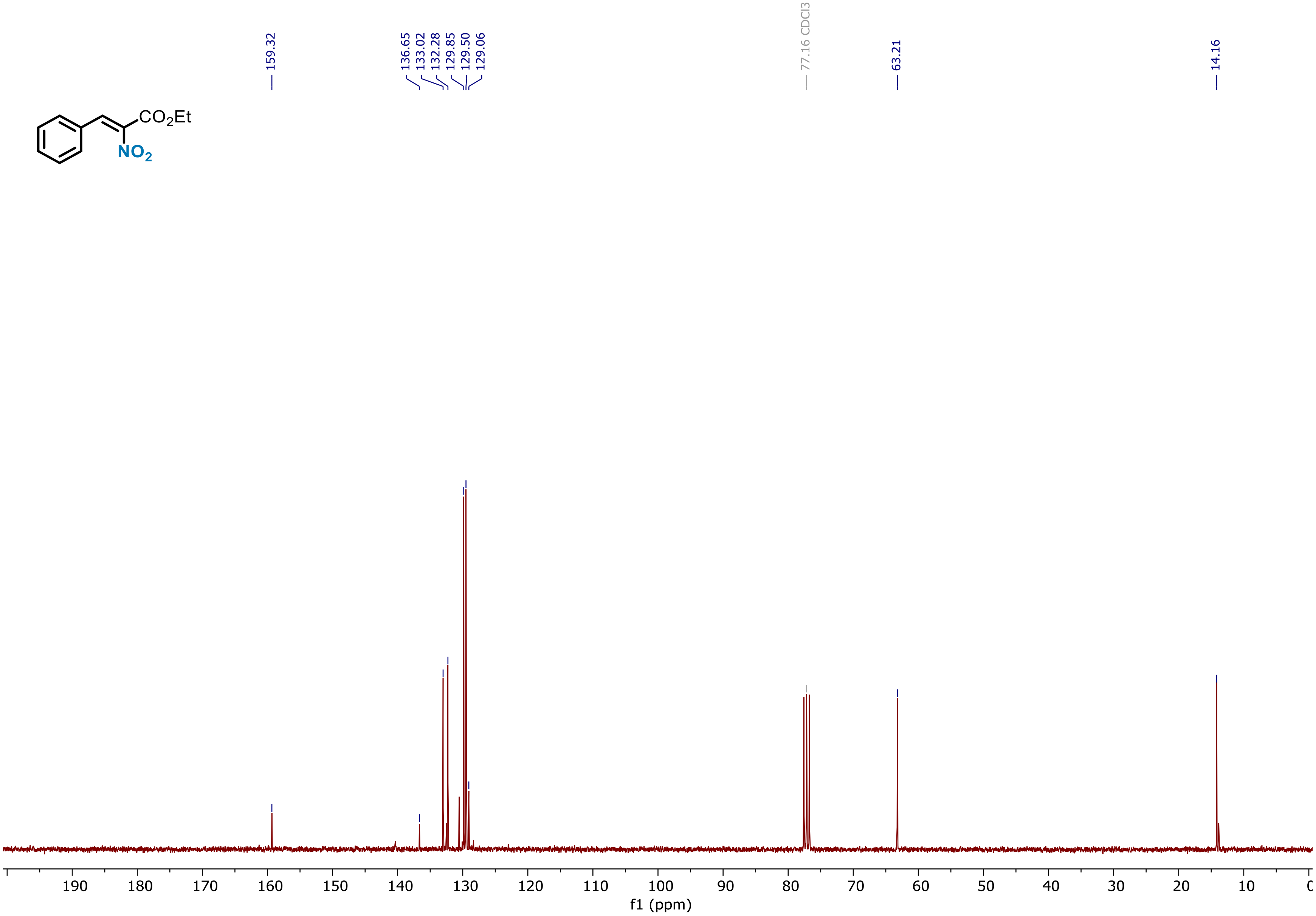

^{1}H NMR (300 MHz, $CDCl_3$) of **60**

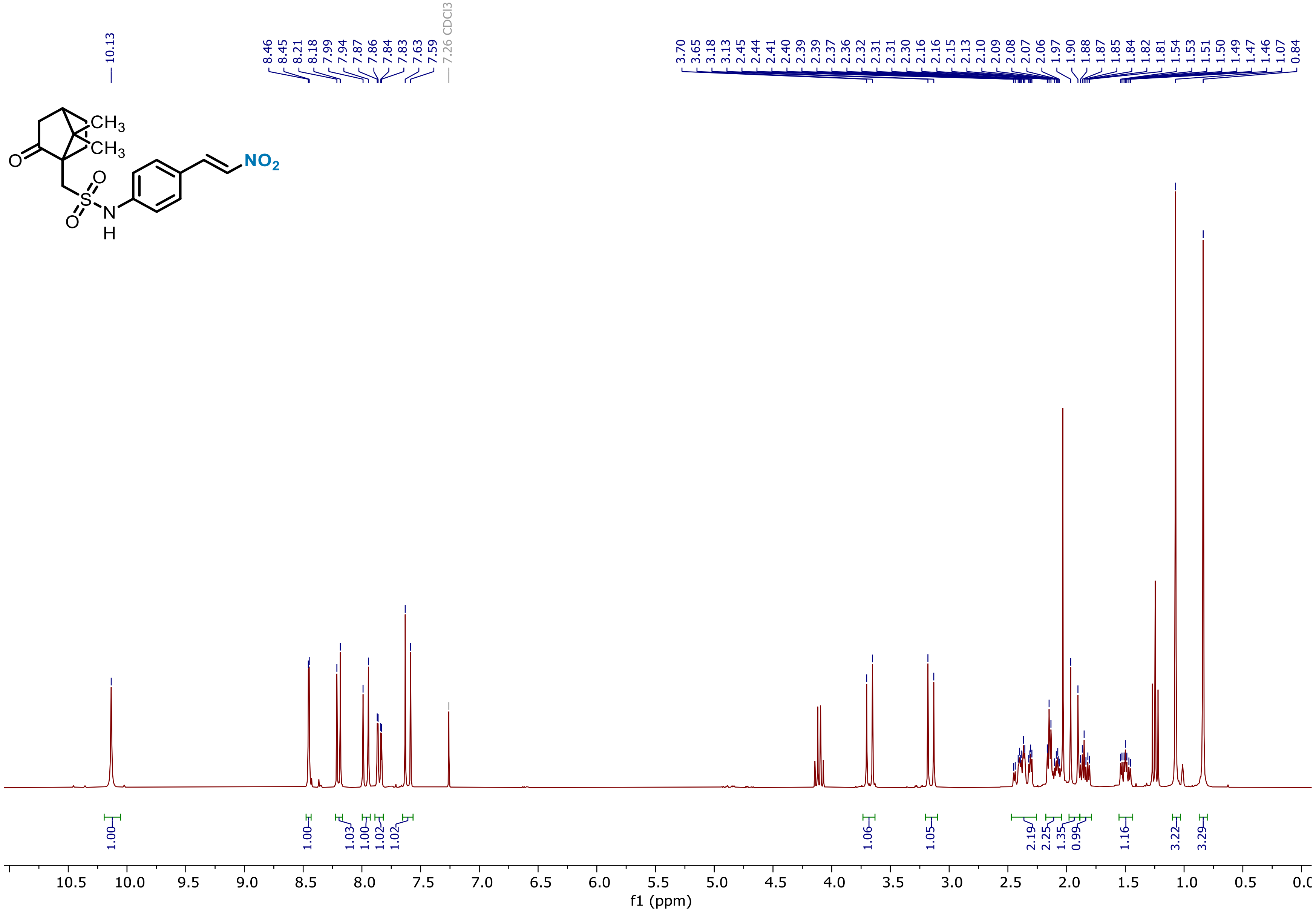

^{13}C NMR (75 MHz, $CDCl_3$) of **60**

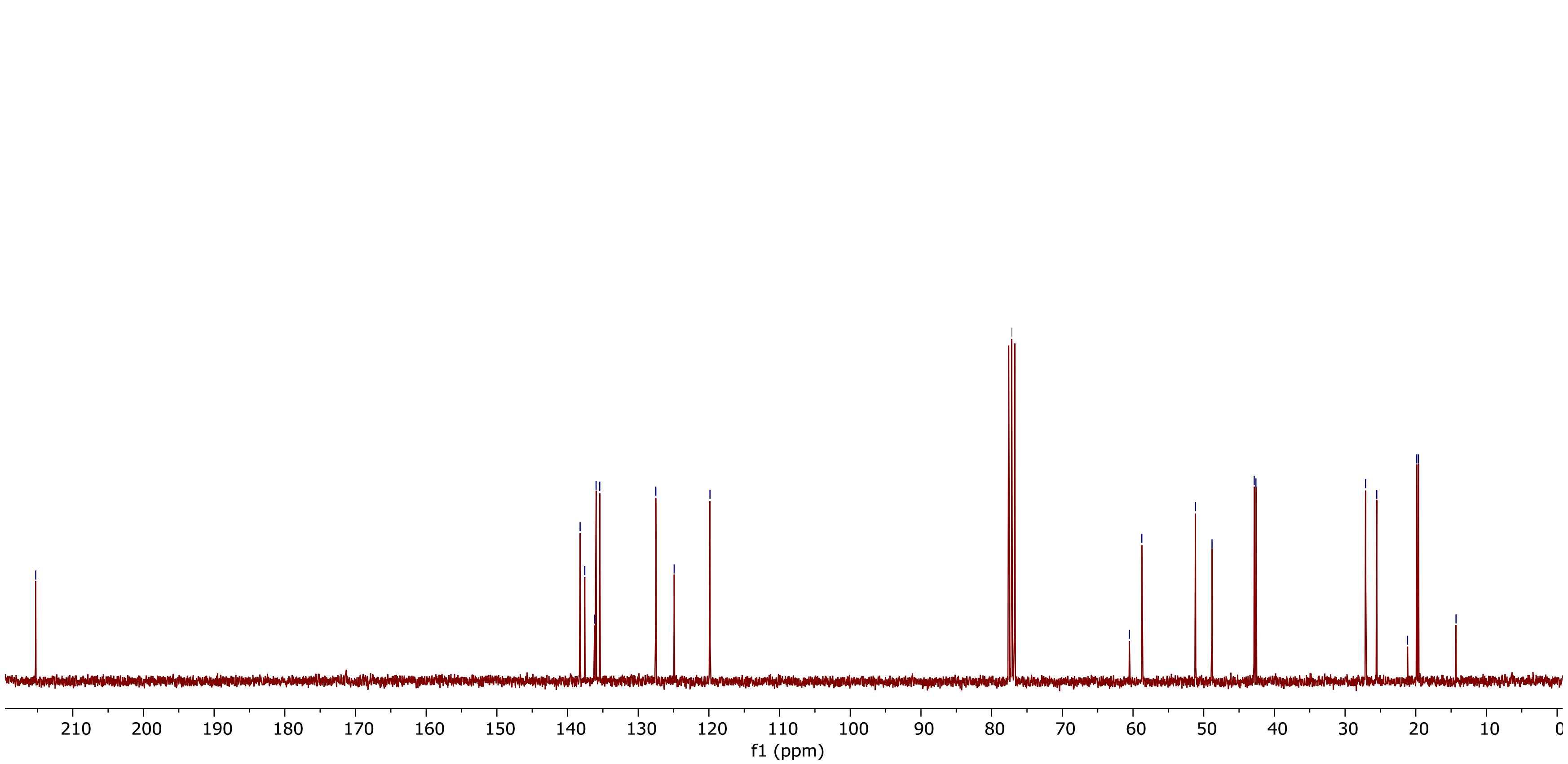

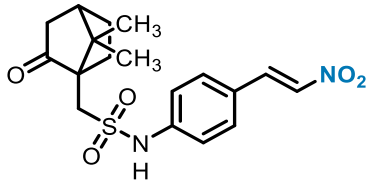

^{1}H NMR (300 MHz, $CDCl_3$) of **61**

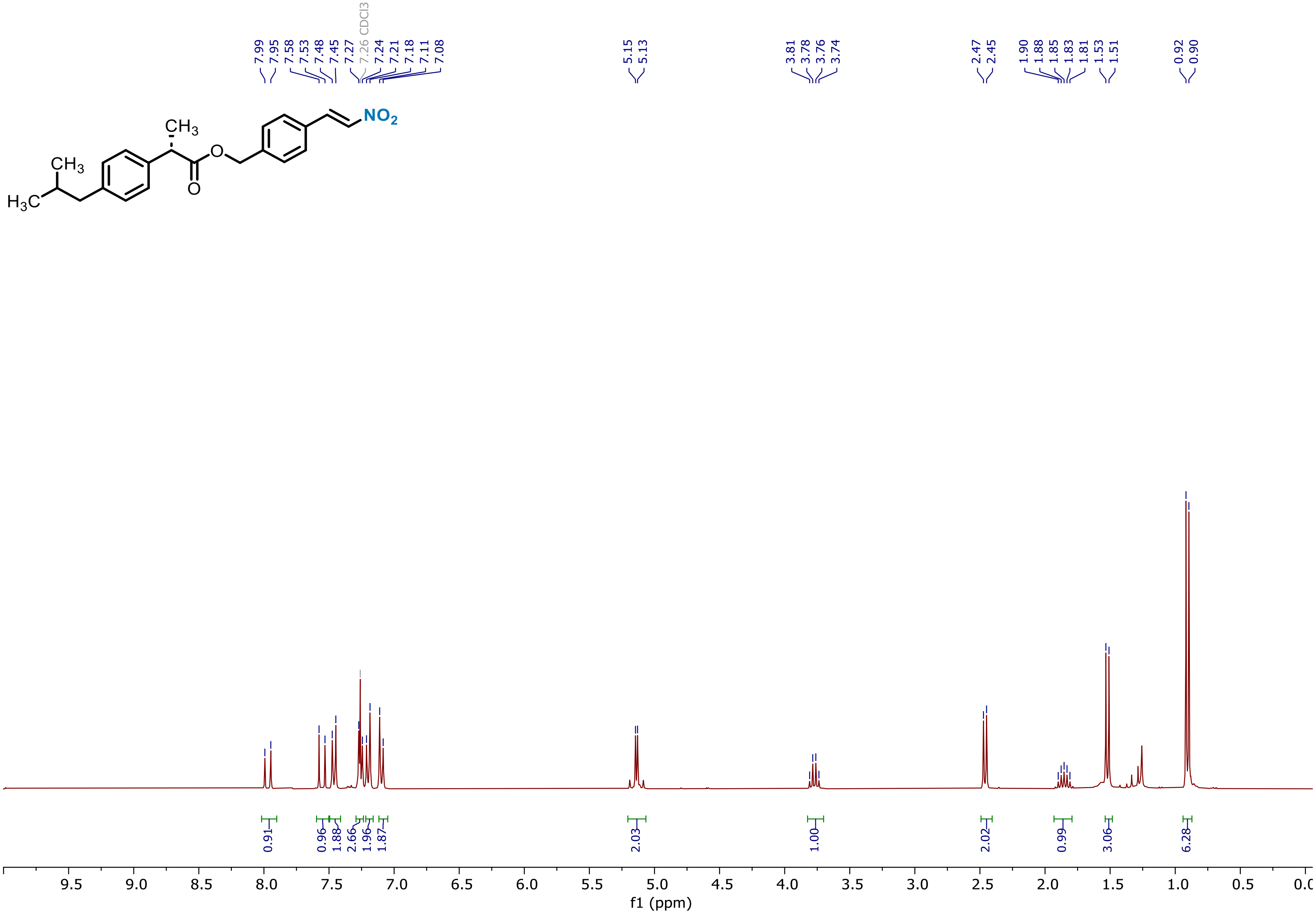

^{13}C NMR (75 MHz, $CDCl_3$) of **61**

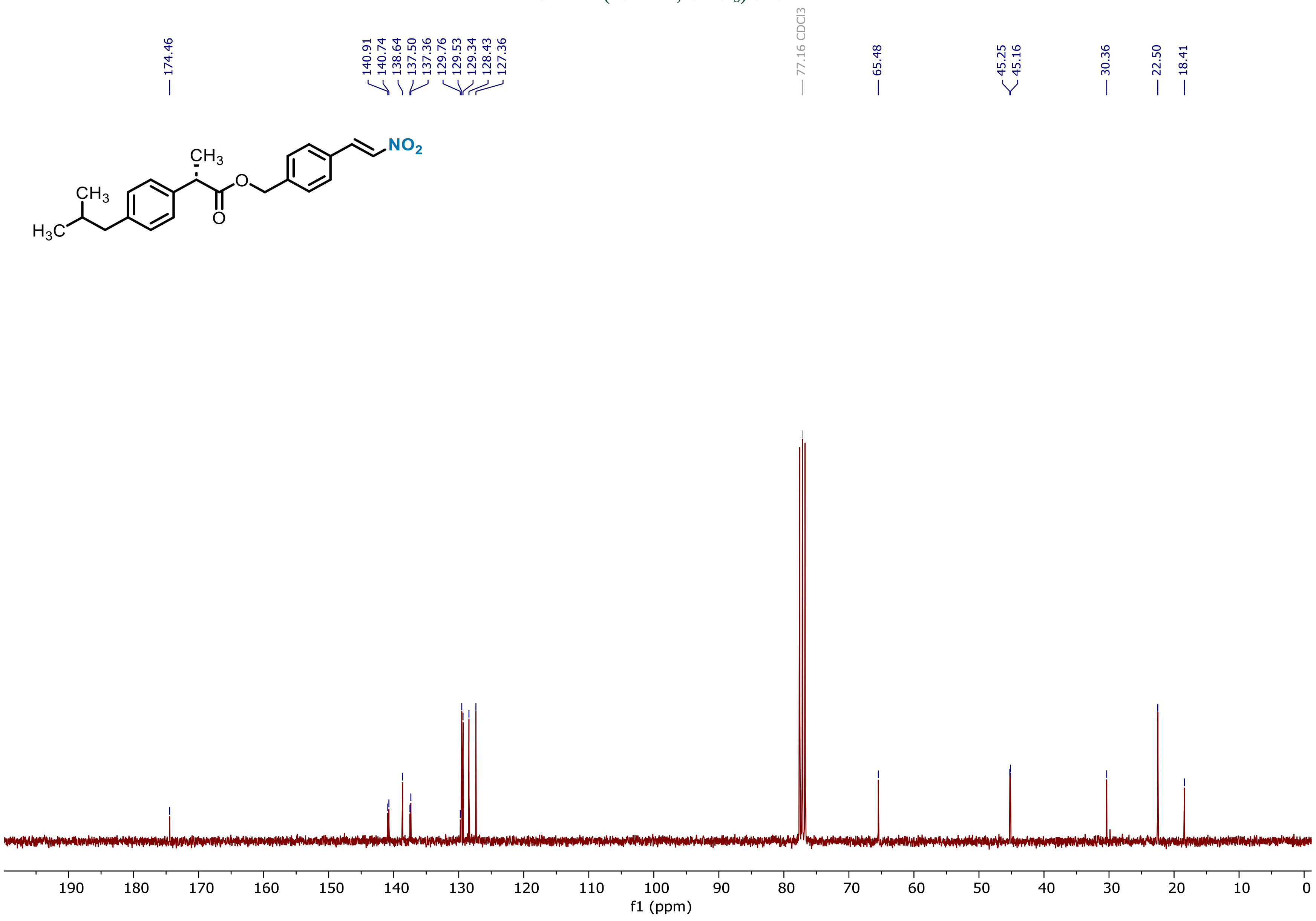

^{1}H NMR (300 MHz, $CDCl_3$) of **62**

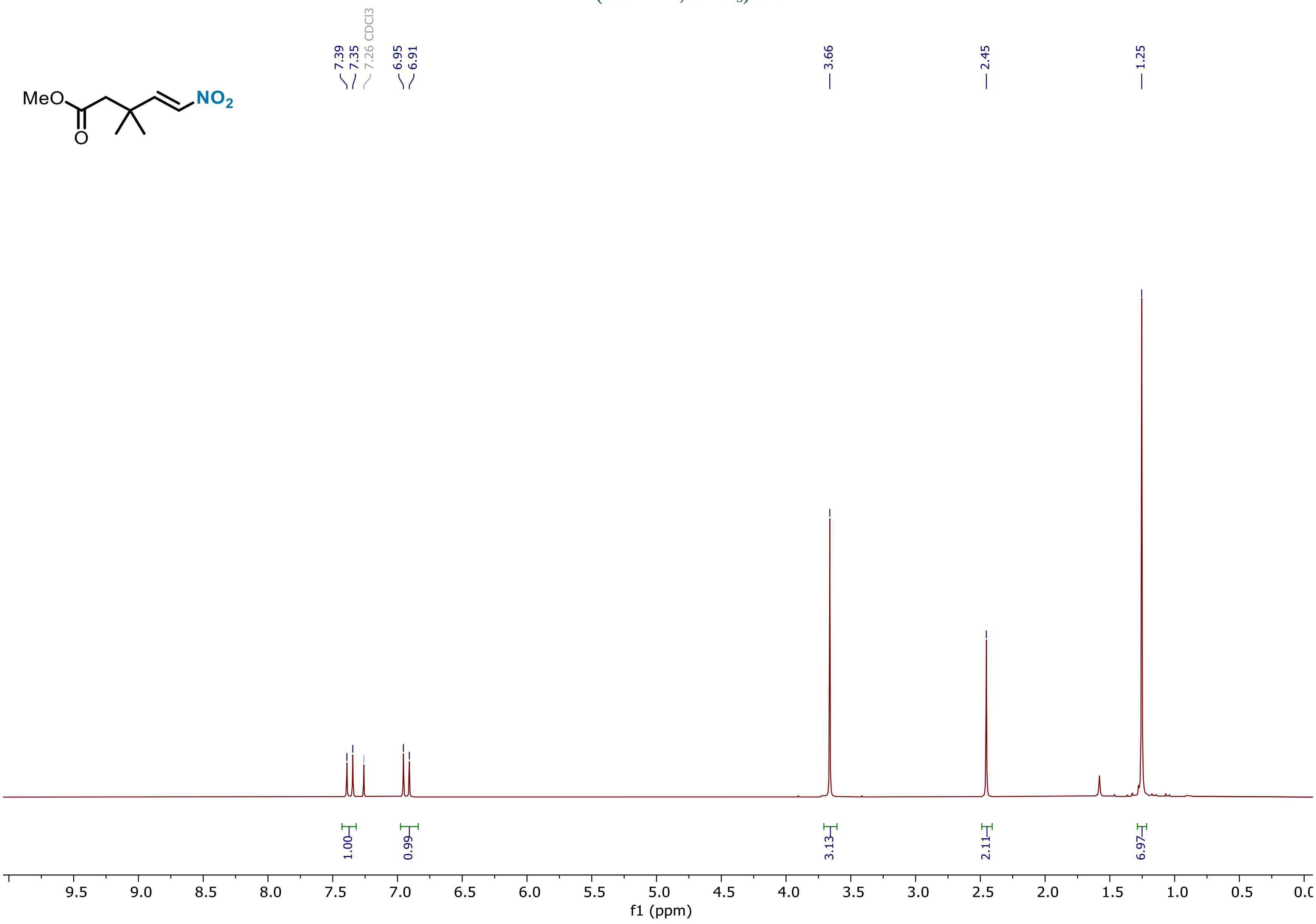

^{1}H NMR (400 MHz, $CDCl_3$) of **63**

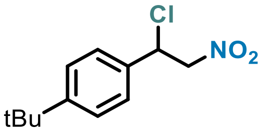

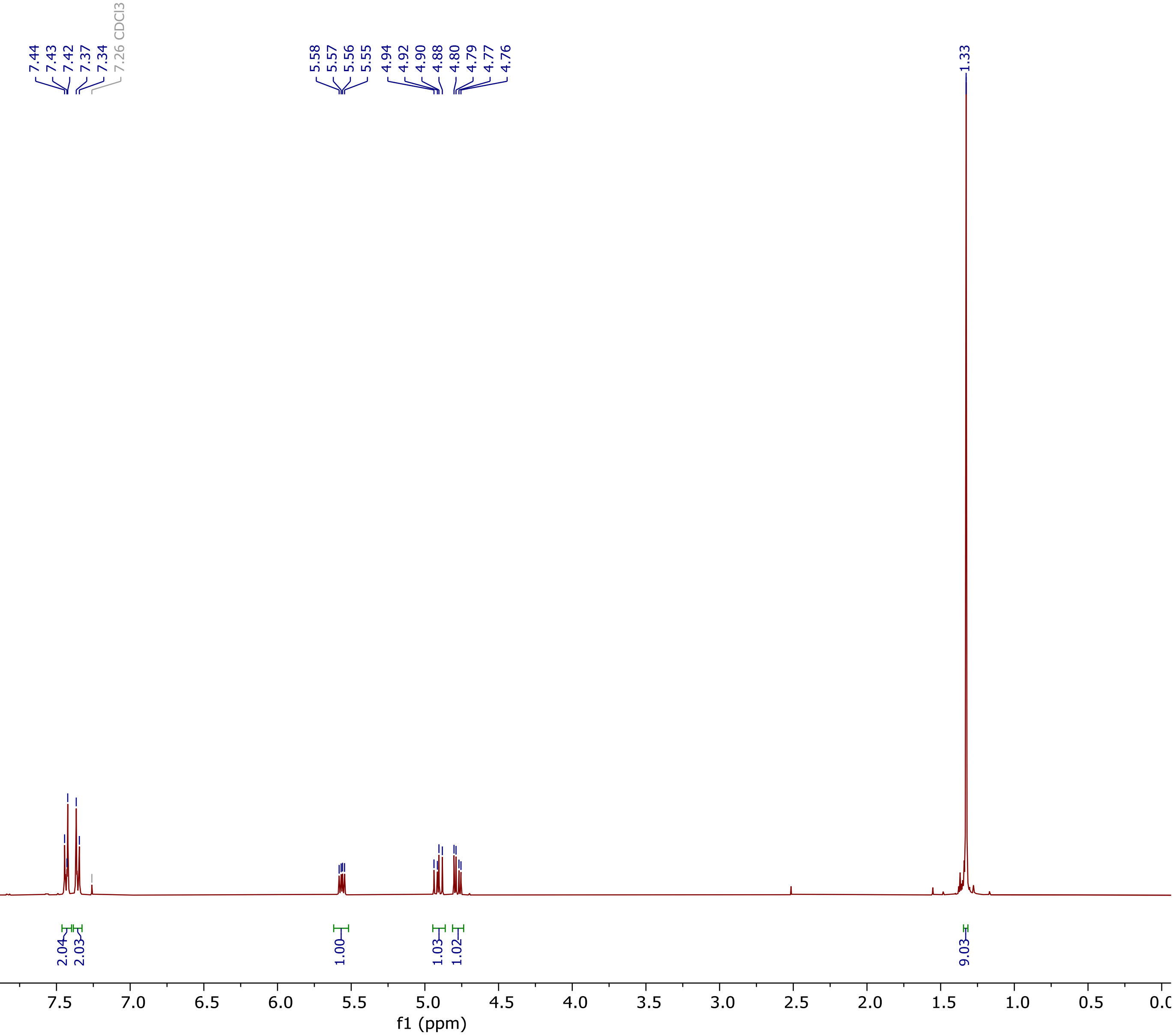

^{1}H NMR (300 MHz, $CDCl_3$) of **64**

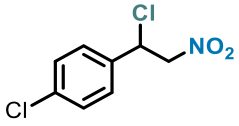

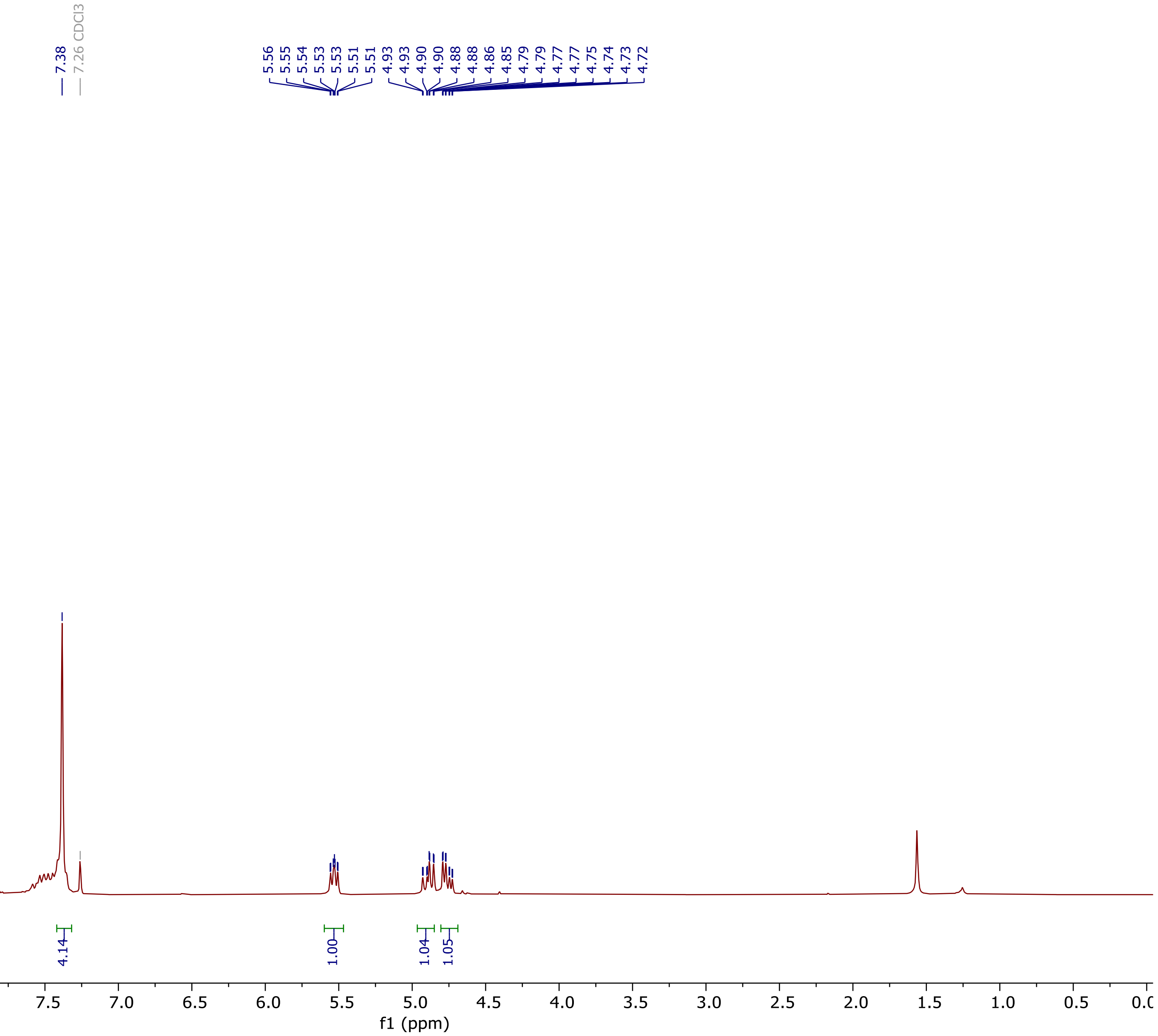

^{13}C NMR (75 MHz, $CDCl_3$) of **64**

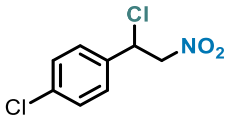

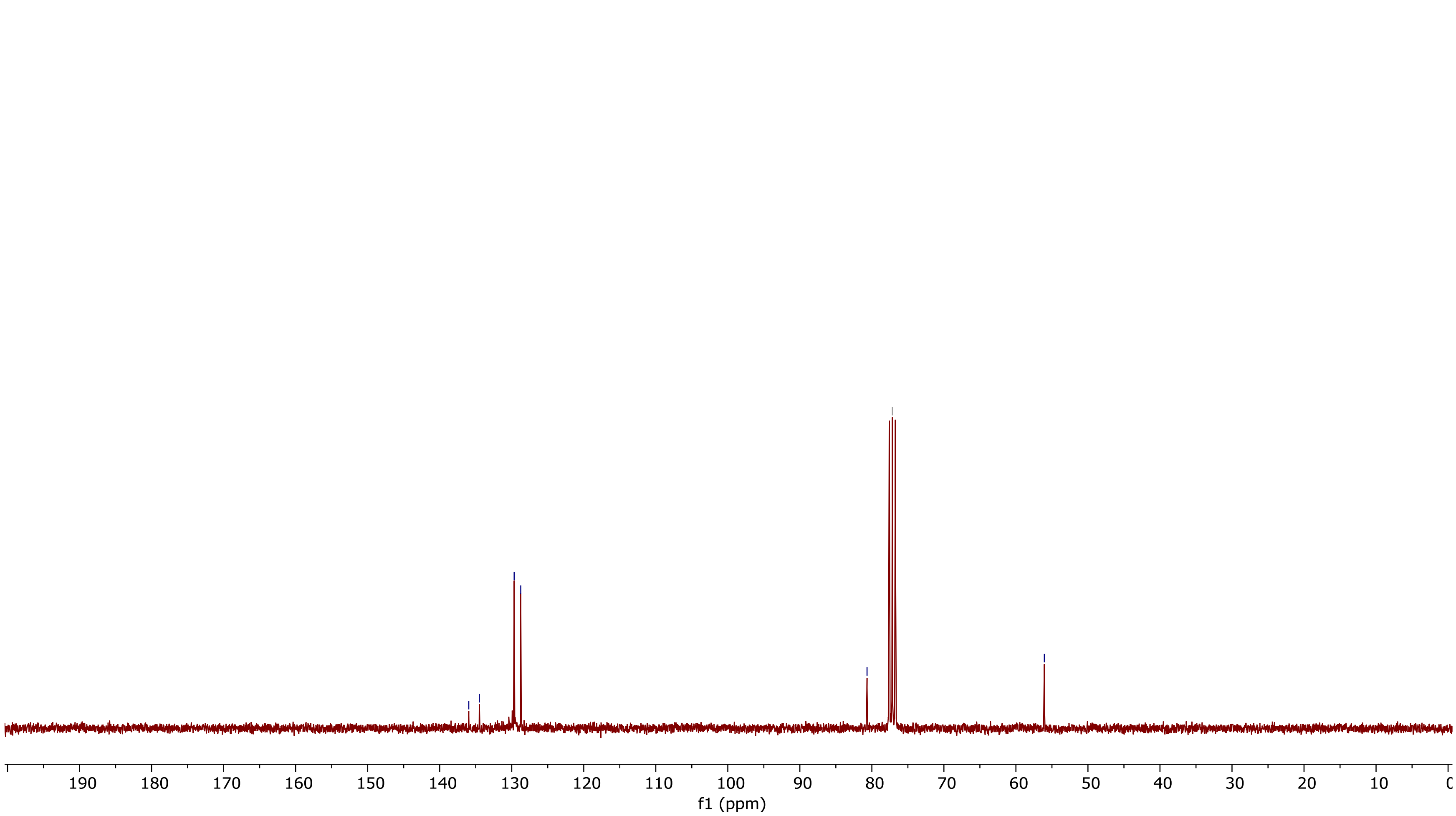

^{1}H NMR (300 MHz, $CDCl_3$) of **65**

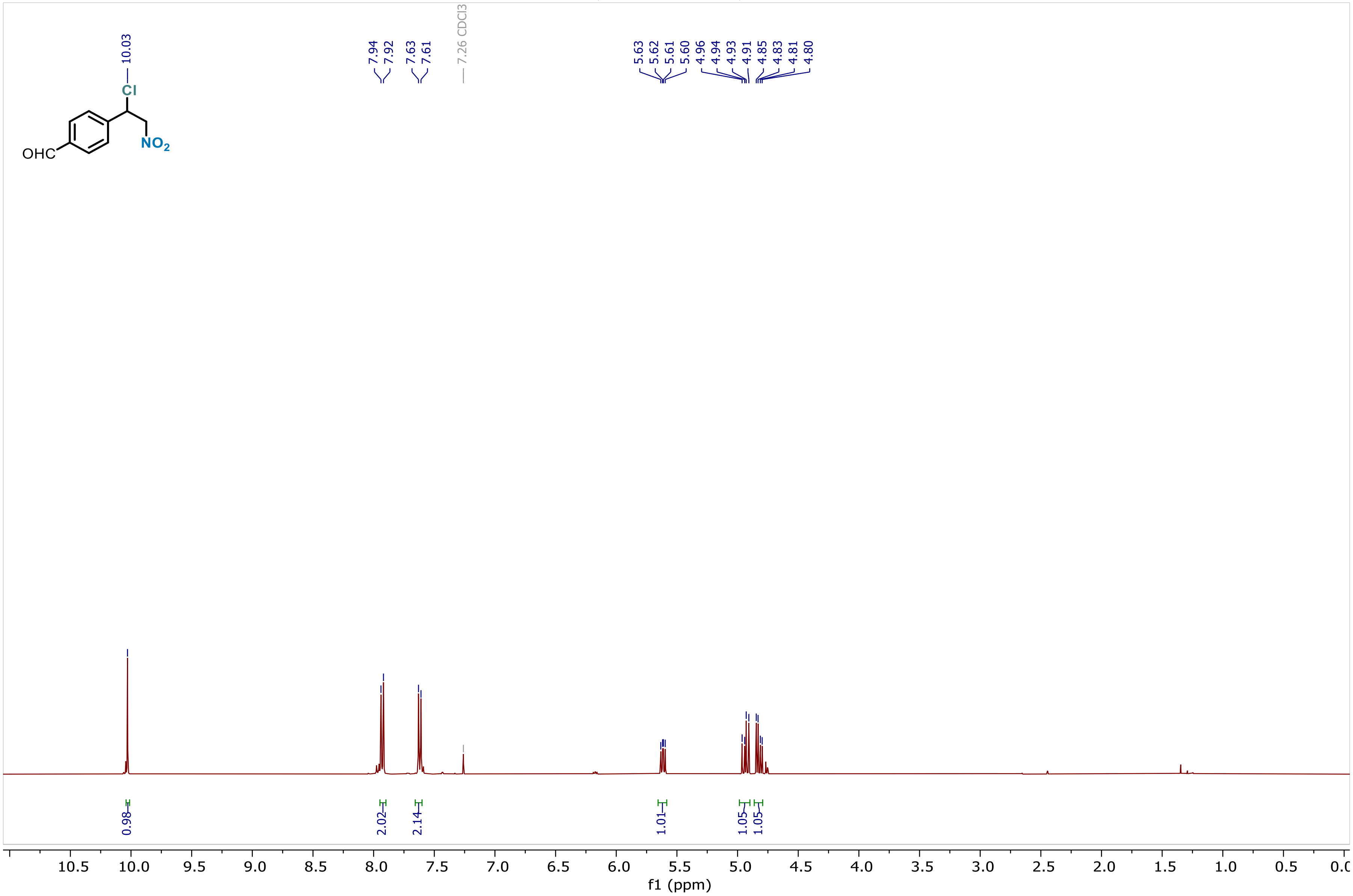

^{1}H NMR (300 MHz, $CDCl_3$) of **66**

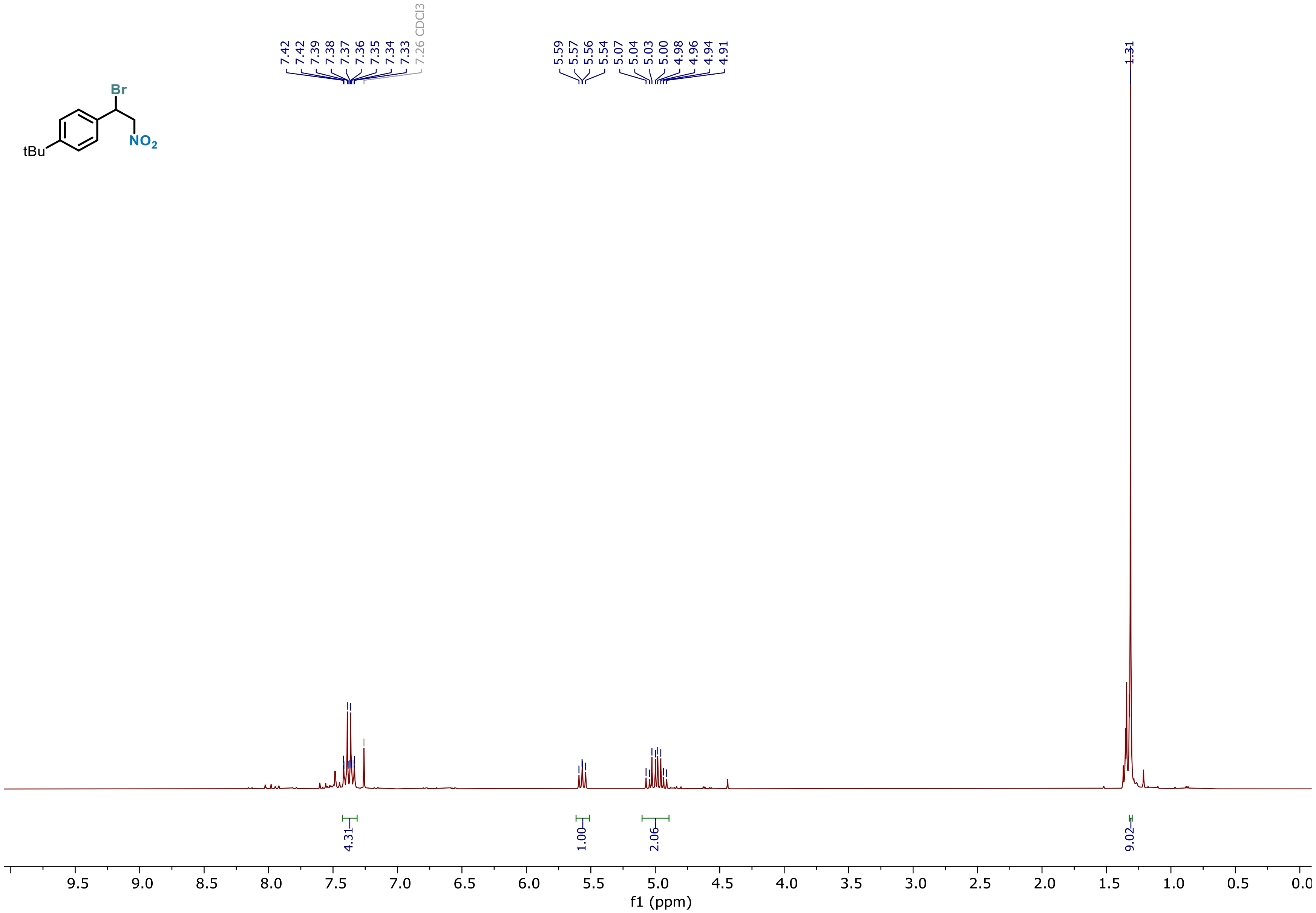

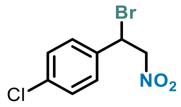

^1H NMR (300 MHz, $CDCl_3$) of **67**

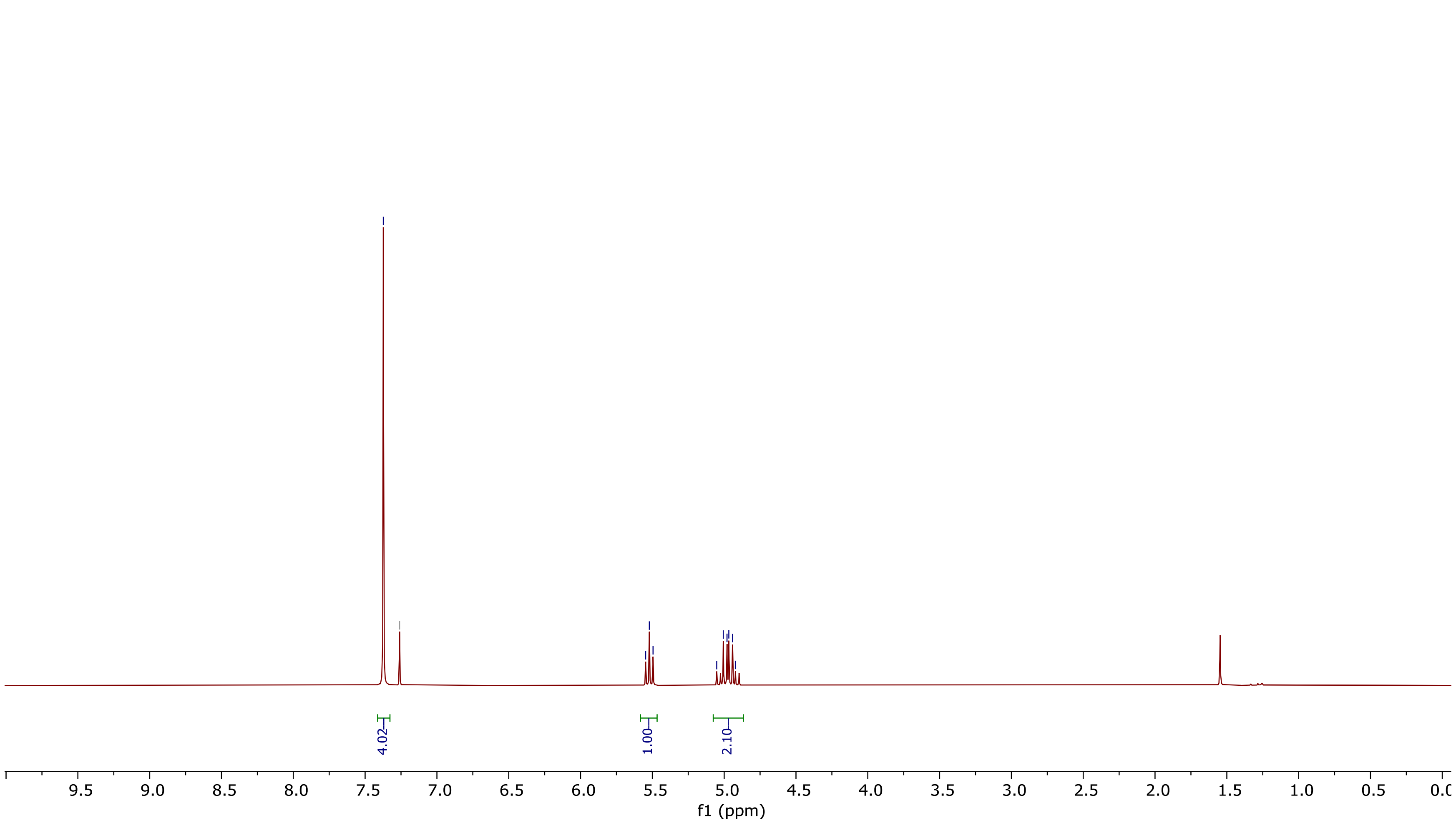

^{13}C NMR (75 MHz, $CDCl_3$) of **67**

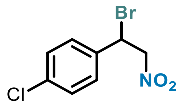

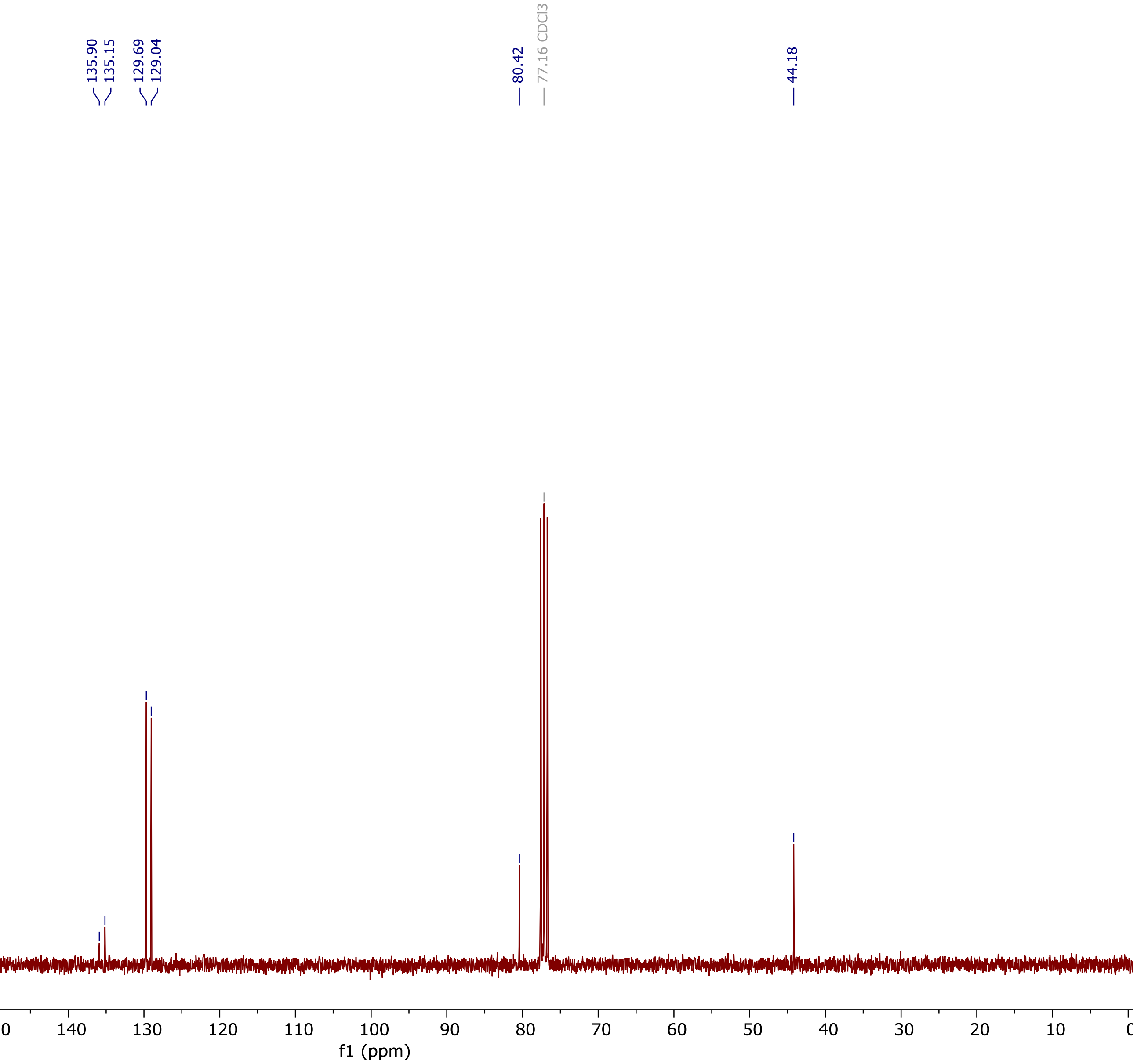

^{1}H NMR (300 MHz, $CDCl_3$) of **68**

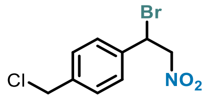

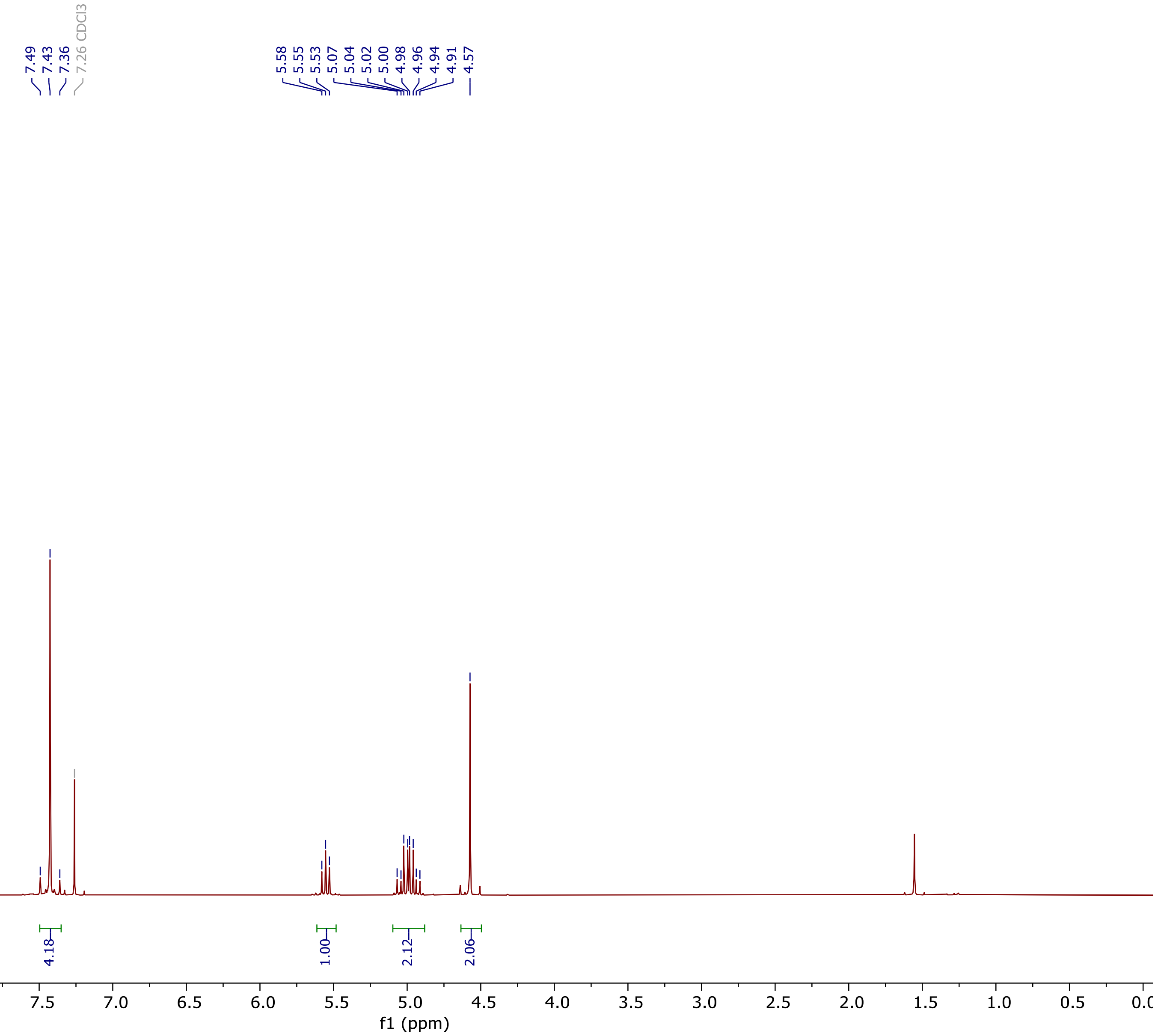

^{1}H NMR (300 MHz, $CDCl_3$) of **69** (unstable, yield is based on NMR purity of isolated compound)

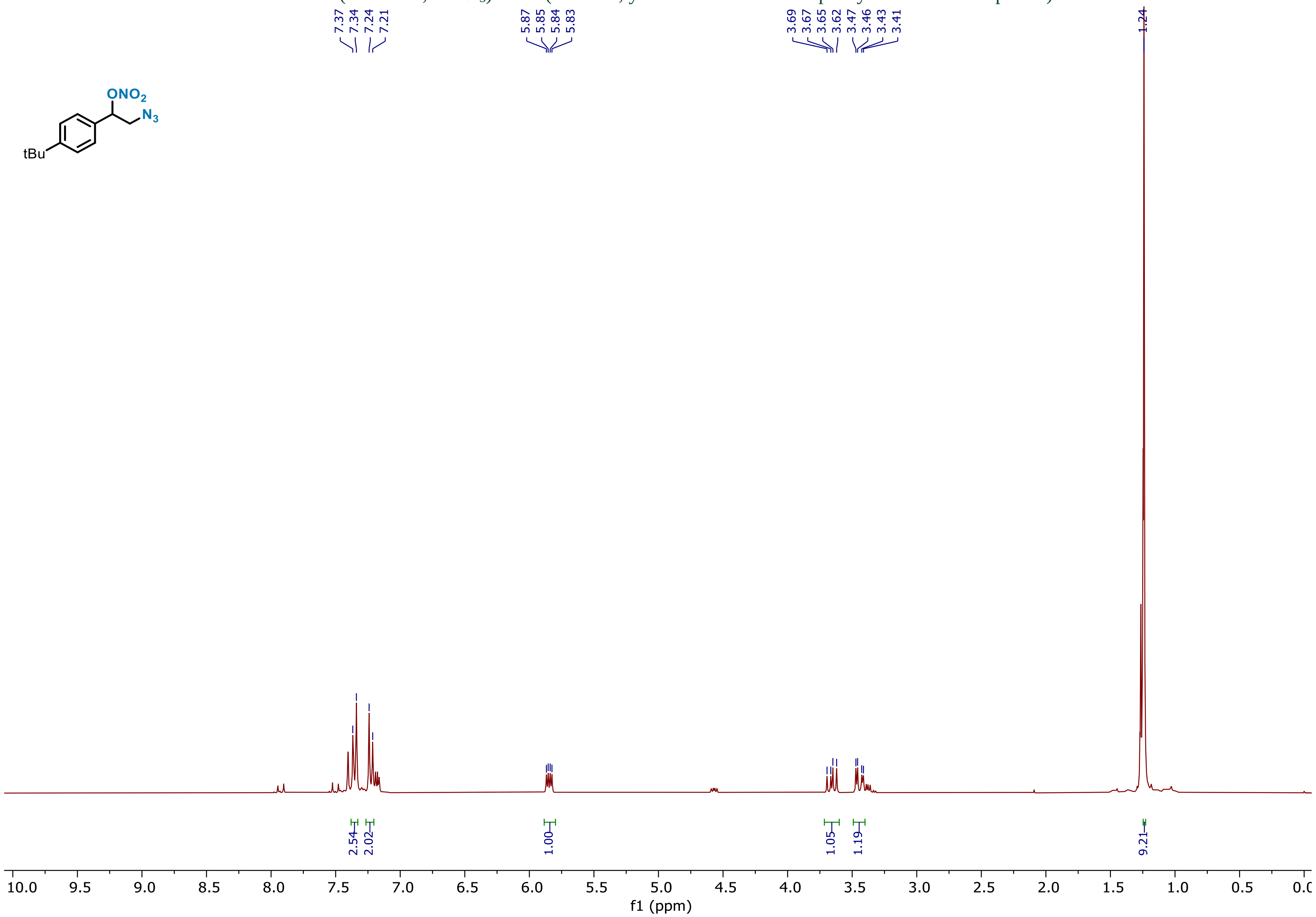

^{13}C NMR (75 MHz, $CDCl_3$) of **69**

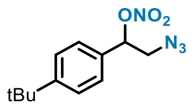

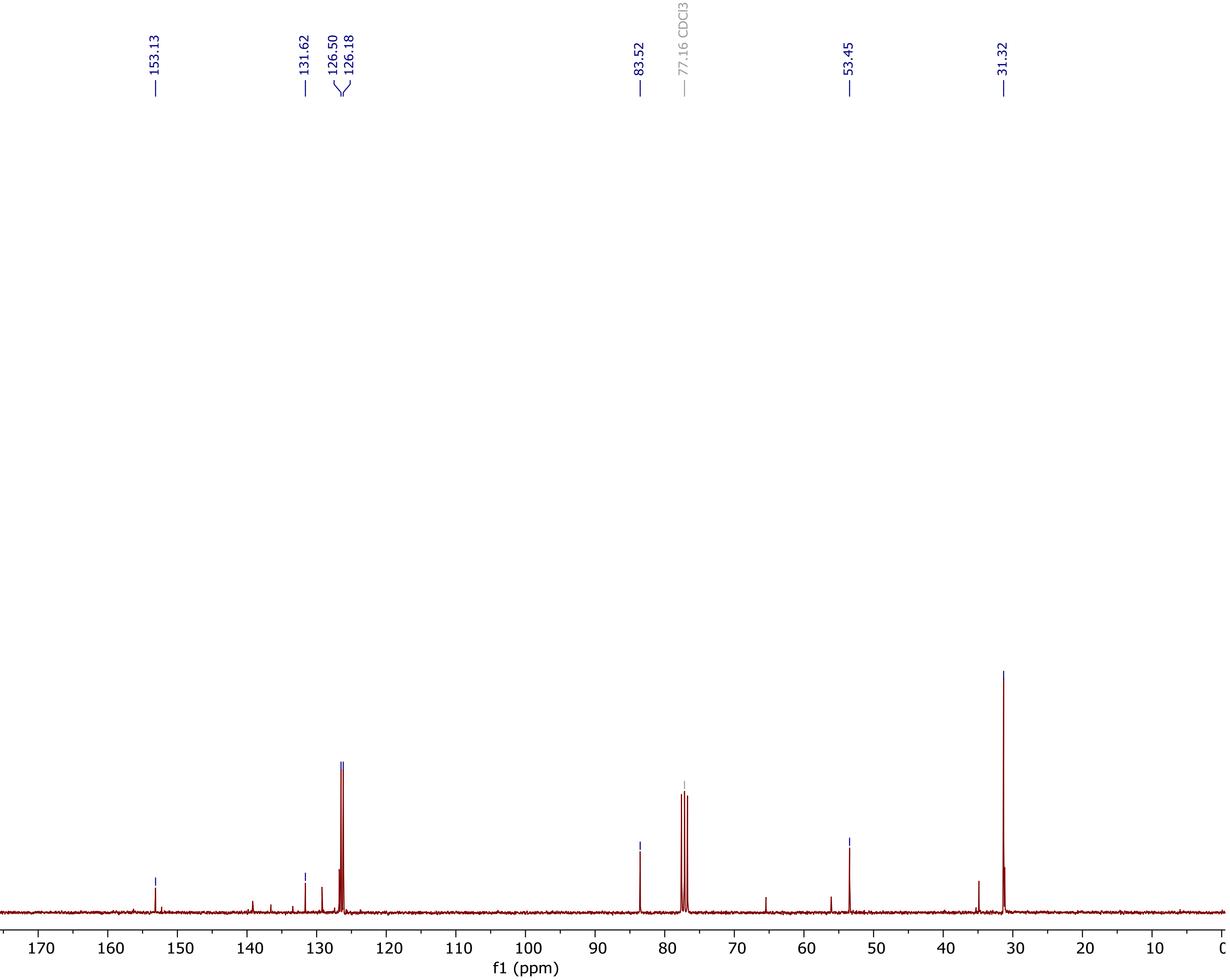

^{1}H NMR (300 MHz, MeOH-d_4) of **70**

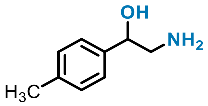

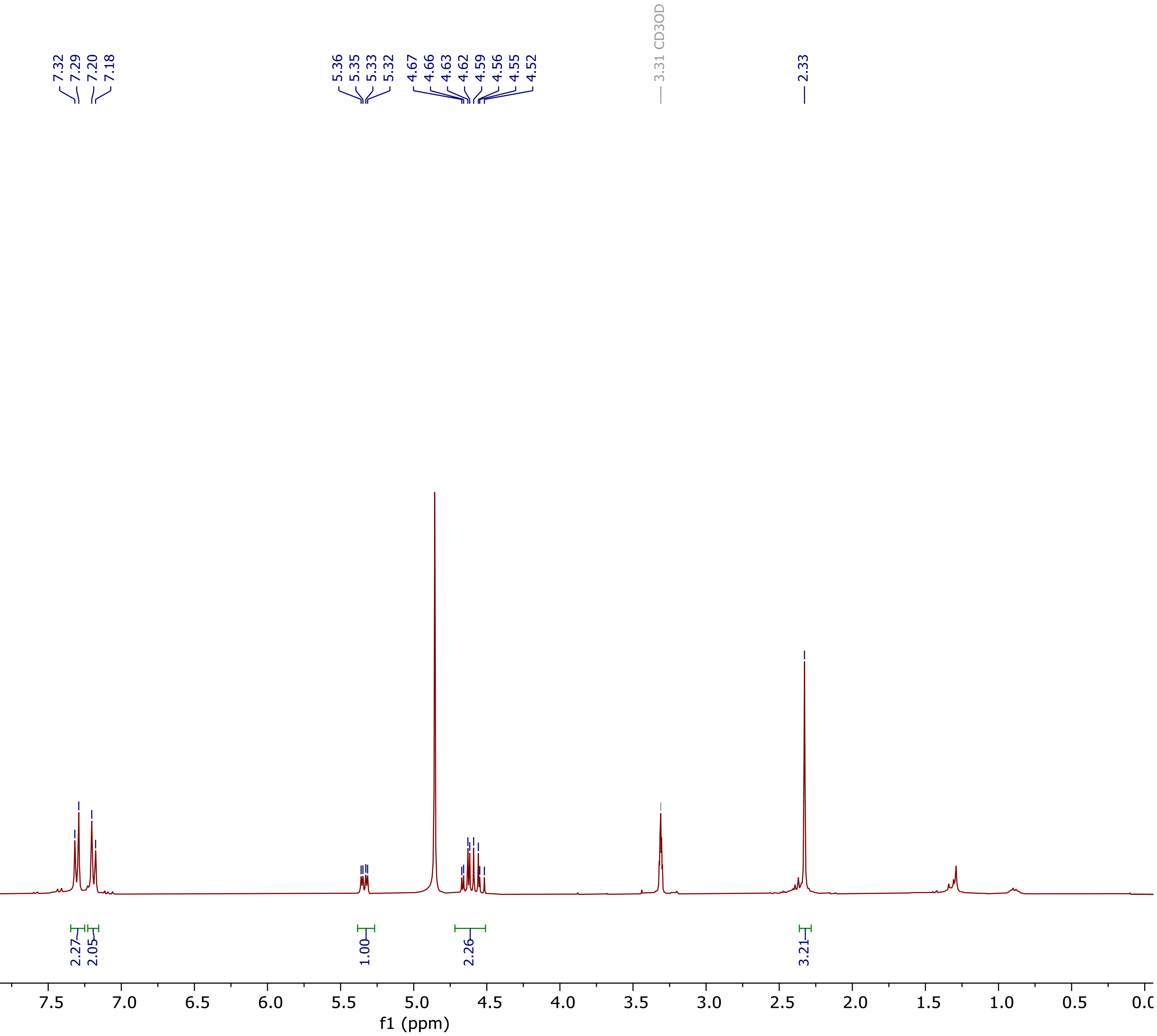

^{13}C NMR (75 MHz, MeOH-d_4) of **70**

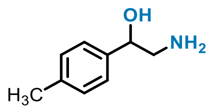

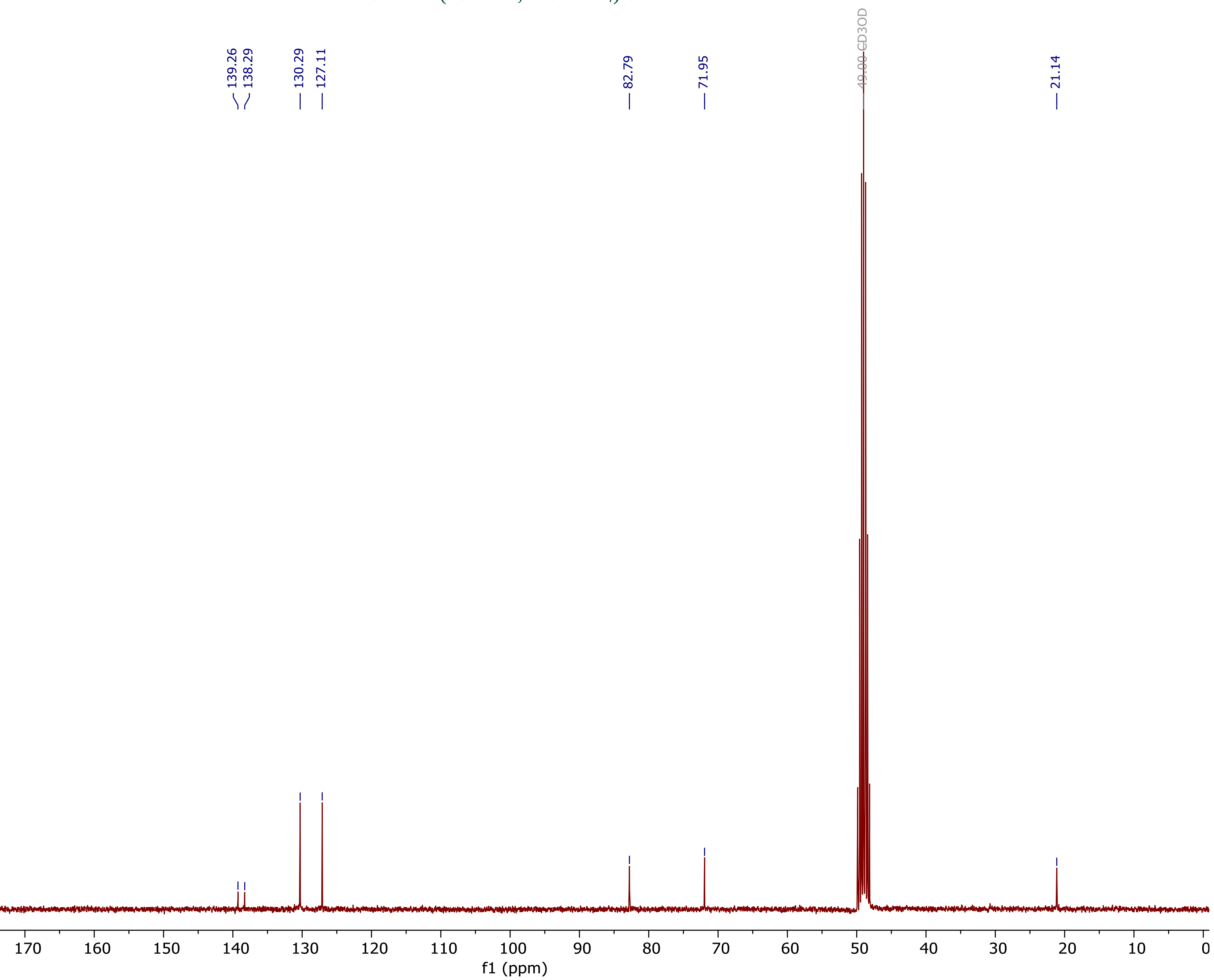

^{1}H NMR (300 MHz, $CDCl_3$) of **71**

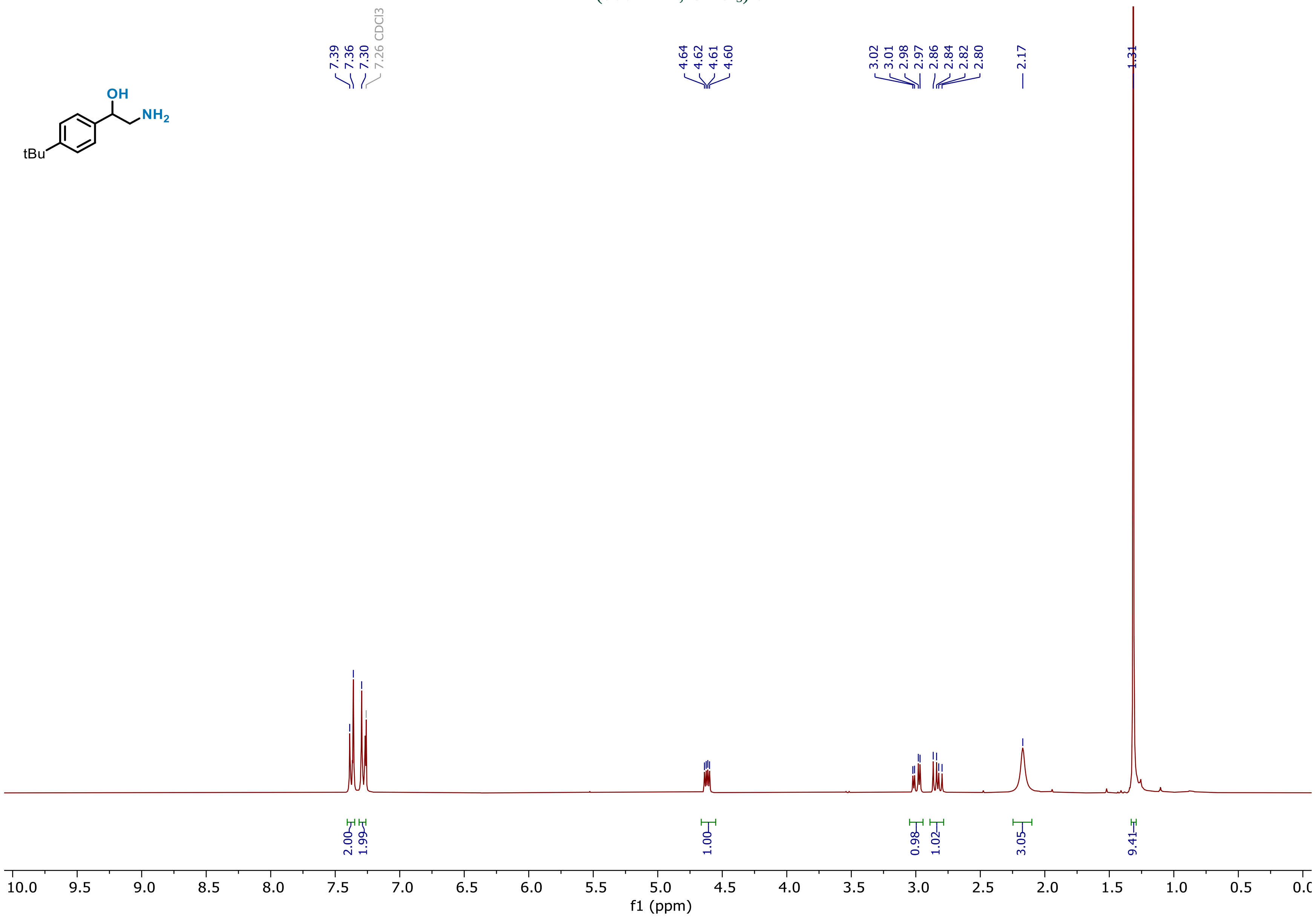

^{13}C NMR (75 MHz, $CDCl_3$) of **71**

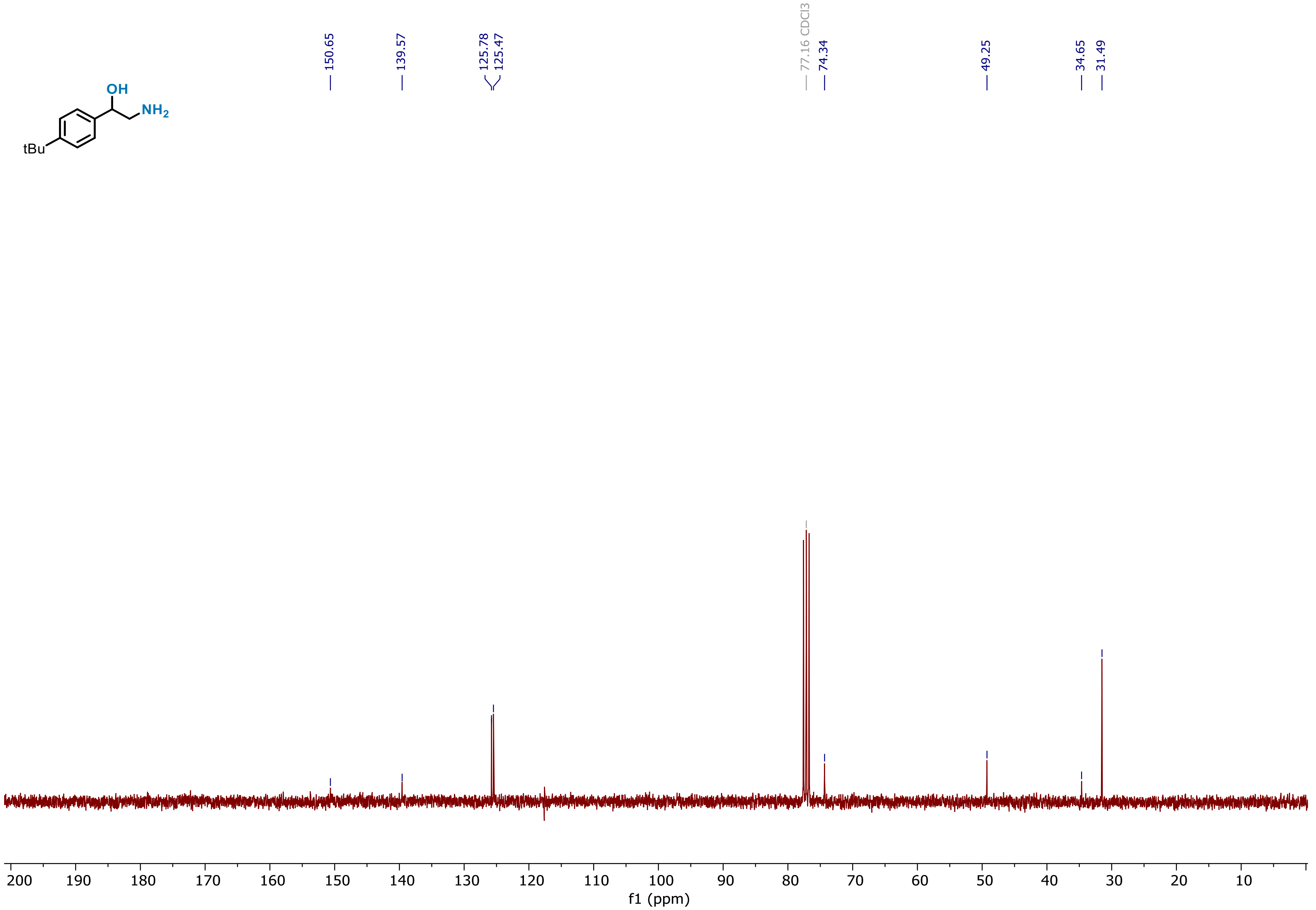

^{1}H NMR (300 MHz, $CDCl_3$) of **72**

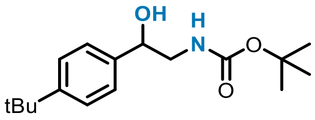

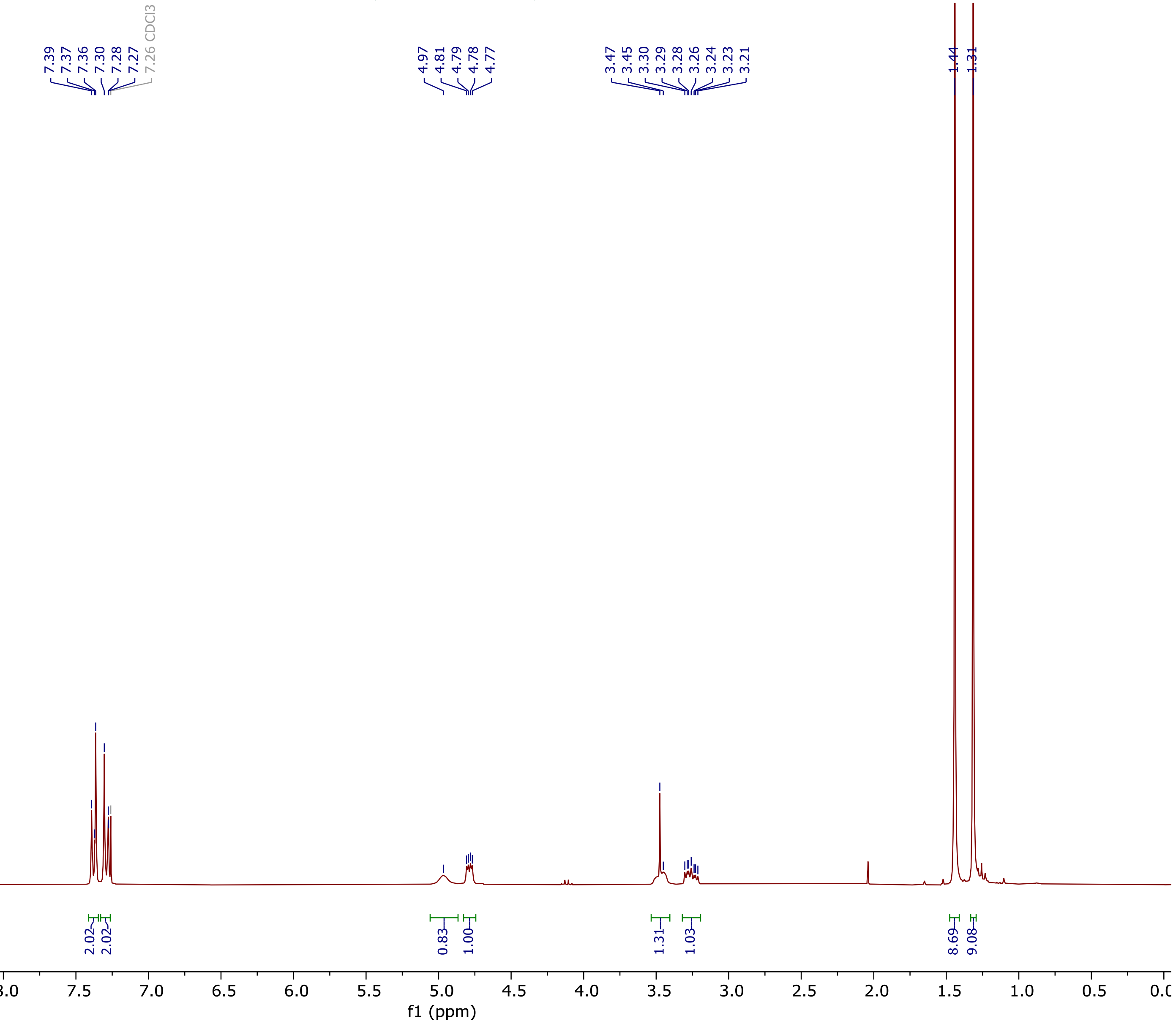

^{13}C NMR (75 MHz, $CDCl_3$) of **72**

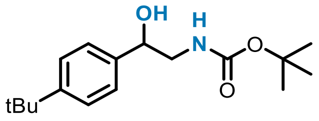

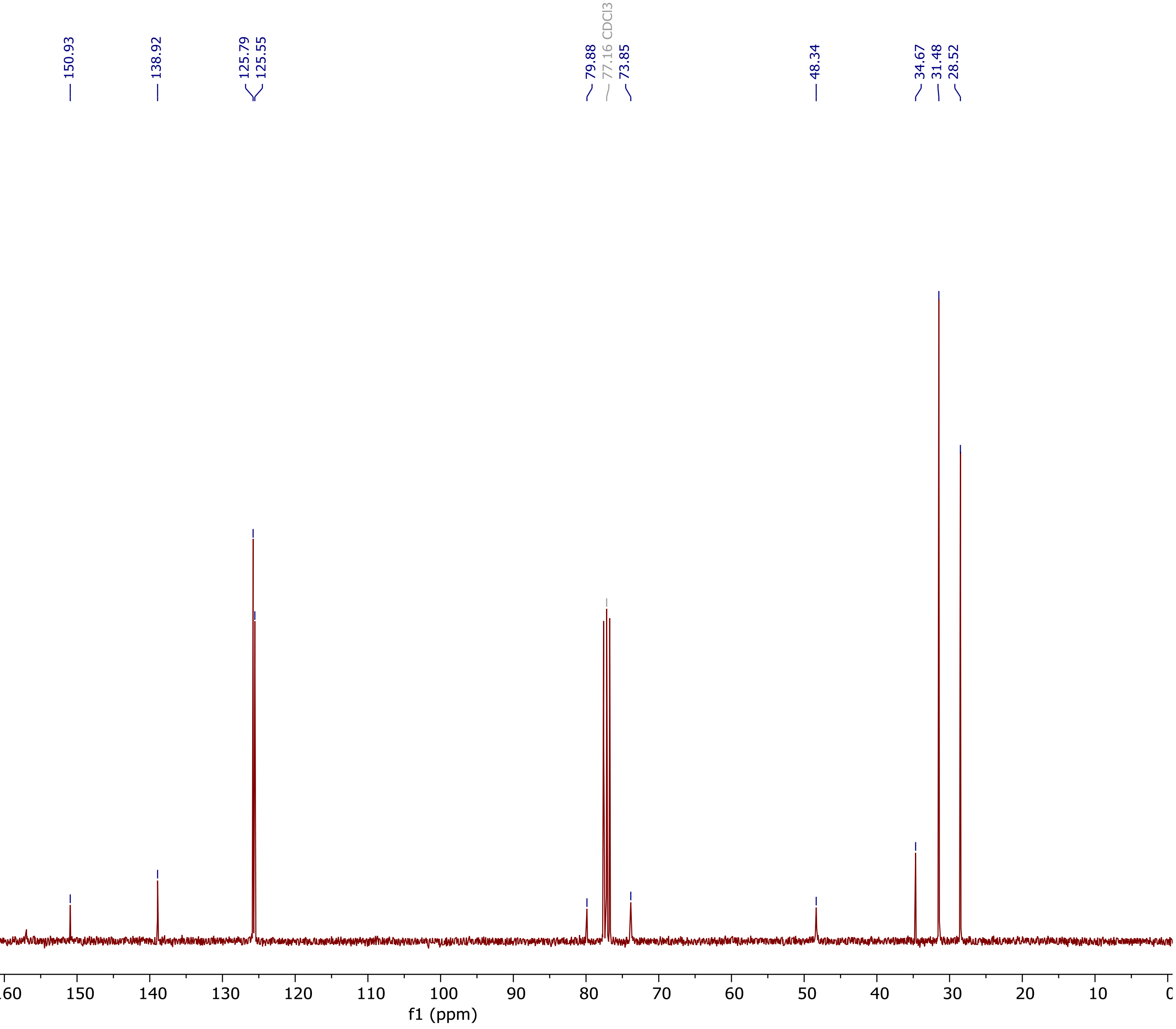

^{1}H NMR (300 MHz, $CDCl_3$) of **73**

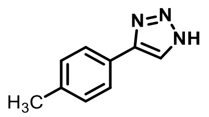

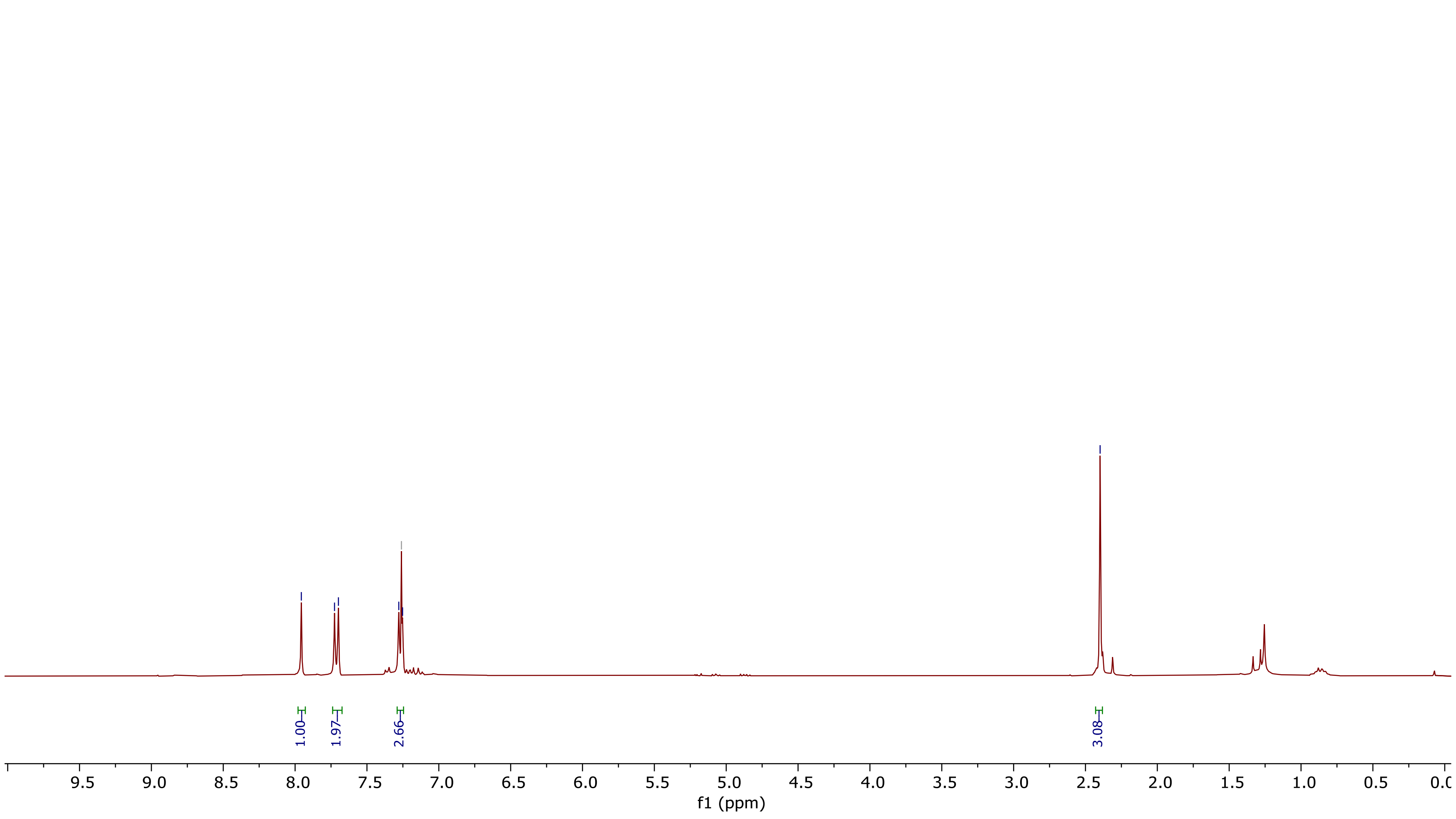

^{1}H NMR (300 MHz, DMSO-d_6) of **74**

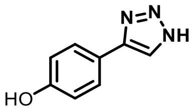

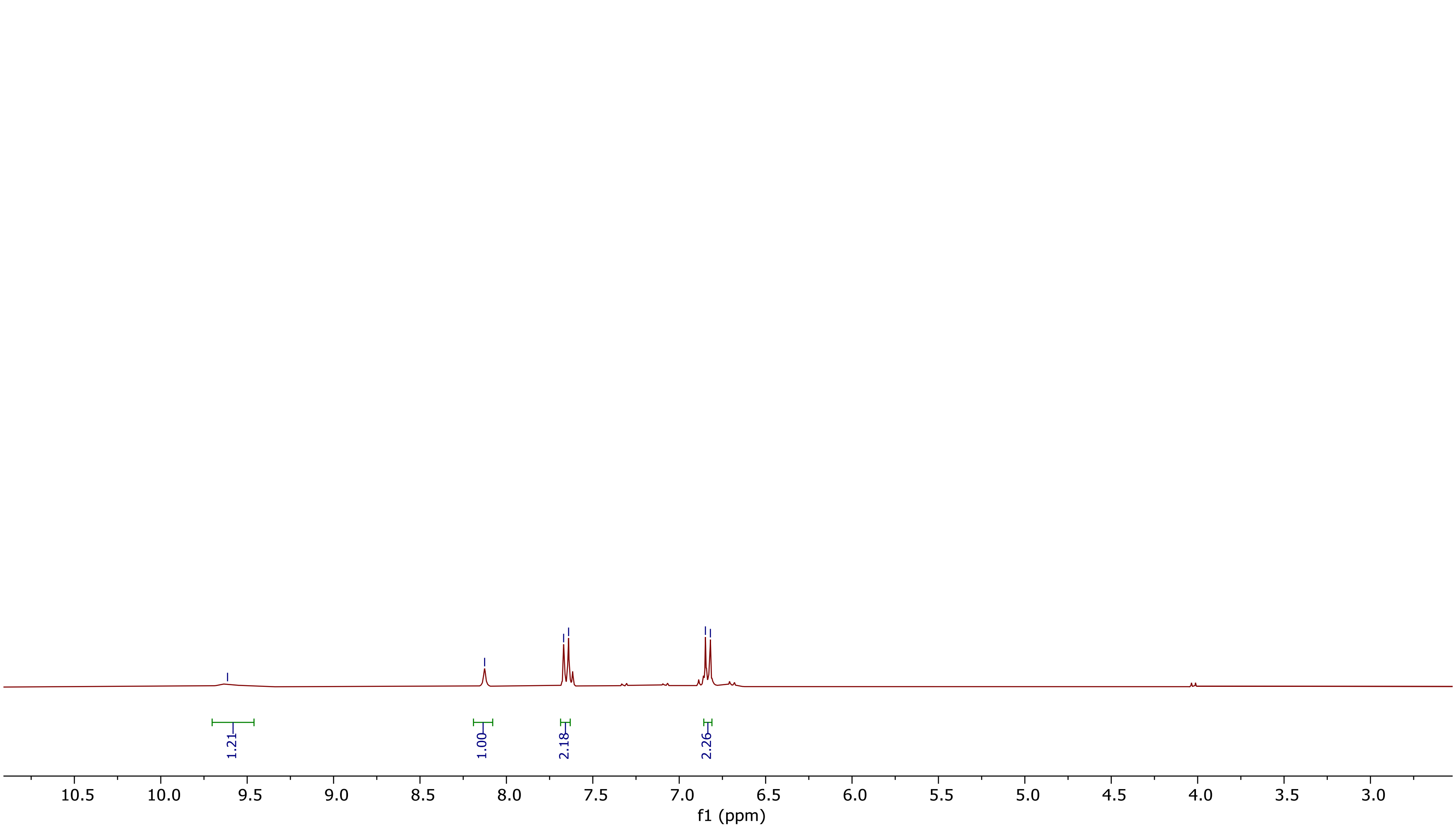

^{1}H NMR (300 MHz, $CDCl_3$) of **75**

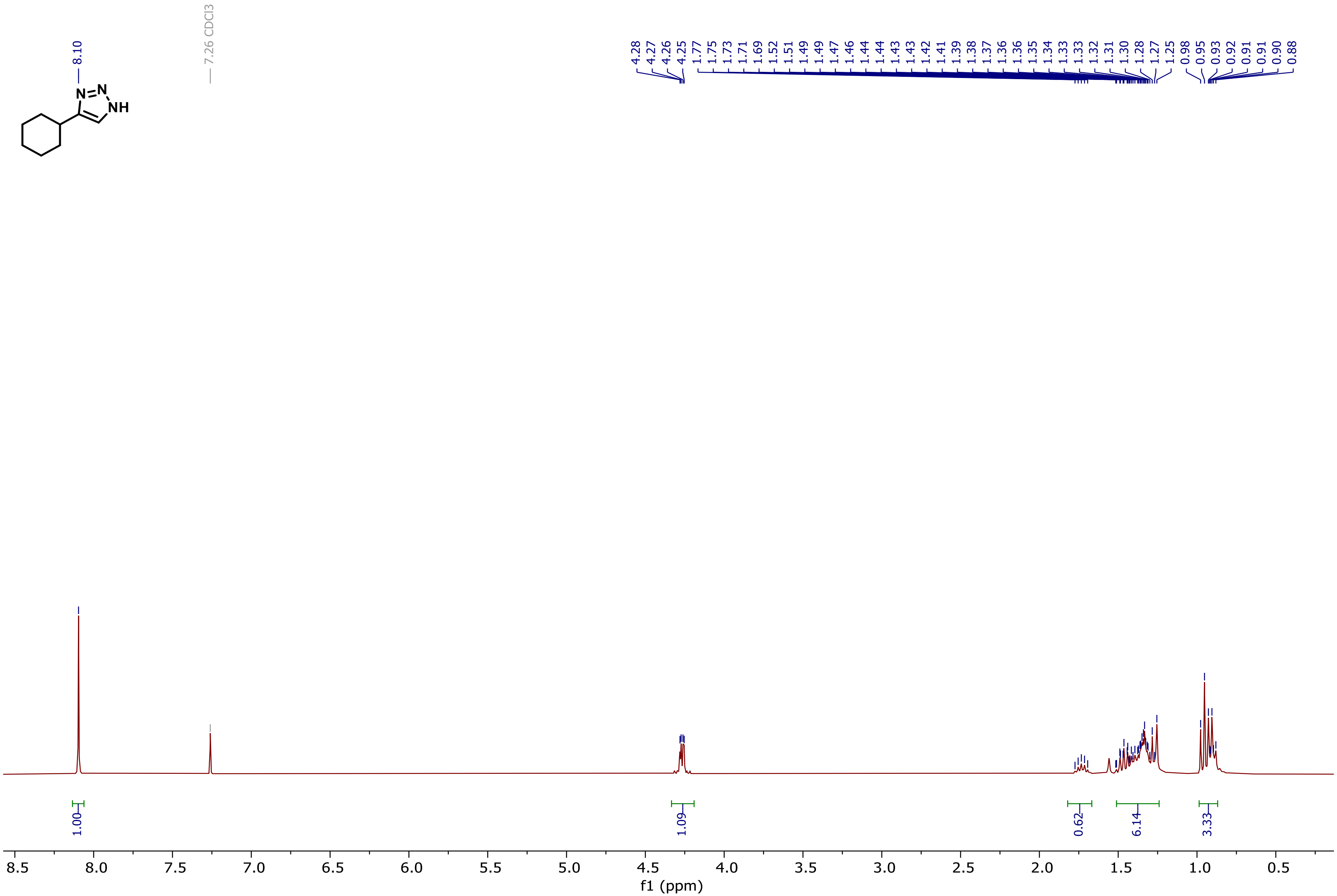

17. References

[1] S. Patra, I. Mosiagin, R. Giri, T. Nauser, D. Katayev, *Angew. Chem.Int. Ed*. **2023**, *62*, e2023005.
[2] K. Zhang, B. Jelier, A. Passera, G. Jeschke, D. Katayev, *Chem. Eur. J*. **2019**, *25*, 12929–12939.
[3] B. M. Trost, N. R. Schmuff, *J. Am. Chem. Soc*. **1985**, *107*, 396–405.
[4] W. Wang, M. K. Brown, *Angew. Chem.Int. Ed*. **2023**, *62*, e2023056.
[5] S. N. Alektiar, Z. K. Wickens, *J. Am. Chem. Soc*. **2021**, *143*, 13022–13028.
[6] D. Uraguchi, K. Oyaizu, T. Ooi, *Chem. Eur. J*. **2012**, *18*, 8306–8309.
[7] T. Taniguchi, A.Yajima, H. Ishibashi, *Adv. Synth. Catal*. **2011**, *353*, 2643–2647.
[8] S. Phae-nok, C. Kuhakarn, P. Leowanawat, V. Reutrakul , D. Soorukram, *Synlett* **2022**, *33*, 1323–1328.
[9] C. Czekelius, E. M. Carreira, *Org. Lett.* **2004**, *6*, 4575–4577.
[10] S. Maity, T. Naveen, U. Sharma, D. Maiti, *Org. Lett*. **2013**, *15*, 3384–3387.
[11] W. Chen, H. Fang, K. Xie, M. Oestreich, *Chem. Eur. J.* **2020**, *26*, 15126–15129.
[12] T. Naveen, S. Maity, U. Sharma, D. Maiti, *J. Org. Chem*. **2013**, *78*, 5949–5954.
[13] J. P. Das, P. Sinha, S. Roy, *Org. Lett*. **2002**, *4*, 3055–3058.
[14] A. V. Buevich, Y. Wu, T.-M. Chan, A. Stamford, *Tetrahedron Lett*. **2008**, *49*, 2132–2135.
[15] B. M. Trost, Y. Wang, *Angew. Chem. Int. Ed*. **2018**, *57*, 11025–11029.
[16] S. Patra, R. Giri, D. Katayev, *ACS Catal*. **2023**, *13*, 16136–16147.
[17] J. H. Schrittwieser, F. Coccia, S. Kara, B. Grischek, W. Kroutil, N. d'Alessandrod, F. Hollmann, *Green Chem*. **2013**, *15*, 3318–3331.
[18] L. Legnani, B. Morandi, *Angew. Chem. Int. Ed*. **2016**, *55*, 2248–2251.
[19] S. Kamijo, Z. Huo, T. Jin, C. Kanazawa, Y. Yamamoto, *J. Org. Chem*. **2005**, *70*, 6389–6397.
[20] R. Jiang, H. B. Sun, S. Li, K. Zhan, J. Zhou, L. Liu, K. Zhang, Q. Liang, Z. Chen, *Synth. Commun*. **2018**, *48*, 2652–2662.
[21] P. R. Clark, G. D. Williams, J. F. Hayes, N. C. O. Tomkinson, *Angew. Chem. Int. Ed.* **2020**, *59*, 6740–6744.
